# Supplementary material for: Primary care and cancer: an analysis of the impact and inequalities of the COVID-19 pandemic on patient pathways
Source: BMJ Open. 2022 Mar 23;12(3):e059374. doi: 10.1136/bmjopen-2021-059374 (PMC8948073; doi:10.1136/bmjopen-2021-059374)

# Annex 1: NCRAS data equity pack, technical notes

CADEAS and NCRAS have produced two equity data packs presenting the latest national data on the number of urgent suspected two-week wait referrals and first definitive treatments for cancer. These data packs are produced on the basis of the Cancer Wait Times data, with analysis from Hospital Episode Statistics (HES) and other sources outlined in their technical notes.

"Any differences between treatment volumes in the published official statistics and the volumes presented in this pack are because:

- Data was extracted from the CWT system at a slightly different time.
- Data included here is based on England residents only.

Additional logic has been applied to remove treatments where some of the information required for this equity analysis is missing or there are potential data quality issues, for example cases with a mismatch between the suspected cancer referral type and sex (eg. gynaecological cancer treatments for men, testicular cancer treatments for women), and suspected cancer referral type and age (eg. suspected children's cancer for patients aged 20 and over)." – NCRAS Cancer data equity pack technical notes, final tab within the downloaded spreadsheet. Available under "Links to data": http://www.ncin.org.uk/local_cancer_intelligence/cadeas as at 26/01/2022

# Annex 2: Consultation definition

CPRD Aurum data dictionary sets out the structure of the data. Within the consultation file there are two variables one can use to identify whether a primary care contact, rather than an administrative note ("EMIS® consultation source identifier" and "Consultation source code identifier").

The EMIS consultation source identifier is the primary variable used. We include the following observations of this variable:

Acute visit, Casualty attendance, Clinic, Emergency appointment, Emergency consultation, Enterprise consultation, Face to face consultation, Follow-up/routine visit, Gp surgery, Home visit, Home visit note, Main surgery, Nursing home, Nursing home visit note, Online services message, Other, Residential home, Residential home visit note, Same day appointment, Surgery consultation, Telephone encounter, Urgent consultation, Walk-in centre, Walk-in clinic

We also include instances where EMIS consultation source identifier is "awaiting review" and the Consultation source code identifier is in the following list:

Consultation, visit, seen in gp unit, seen in private clinic, seen in rapid access clinic at gp surgery, seen in urgent care centre, online communication.

We then further exclude records on the basis of the category of staff responsible for the record. The "Job category" variable from the staff file, linked by the consultation id is used. We only include as a consultation records filled out by GPs, doctors, nurses and other health care professionals as defined in CPRD's numerical codes listed below:

GP – 4, 5, 15, 24, 31, 181, 183

Dr – 1, 41, 91, 116, 119, 121, 126, 173, 177, 197

Nurse – 8, 9, 27, 33, 47, 48, 50, 55, 59, 60, 61, 111

Other healthcare professional - 2, 3, 6, 7, 10:14, 16, 17, 34:37, 42, 43, 52, 54, 58, 62:65, 68, 72, 73, 77, 80, 82, 83, 86:89, 94, 95, 97, 100:102, 105, 106, 112:114, 118, 122, 125, 127, 131, 135, 136, 138, 141, 142, 145, 148, 149, 154, 156, 158, 168, 185, 186, 188, 189, 204, 208

In Table A2.1 we present the CPRD Aurum Staff Category list.

In Table A2.2 we present the total number of consultations identified from 01 January 2016 to 31 January 2021, the count with each combination of staff category, "EMIS® consultation source identifier" and "Consultation source code identifier" in Table A2.2 we show the count of records that were excluded, highlighting those that were excluded on the basis of staff category, not the consultation file description variables.

Table A2.1: CPRD Aurum Staff Job Categories

1 Consultant
2 Hospital Practitioner
3 Clinical Medical Officer
4 General Medical Practitioner
5 Salaried General Practitioner
6 Midwife - Sister/Charge Nurse
7 Midwife
8 Community Practitioner
9 Community Nurse
10 Chiropodist/Podiatrist
11 Dietitian
12 Pharmacist
13 Clinical Psychologist
14 Health Care Support Worker
15 Associate Practitioner - General Practitioner
16 Counsellor
17 Phlebotomist
18 Clerical Worker
19 Manager
20 Analyst
21 System Administrator
22 Desktop Support Administrator
23 System Worker
24 GP Registrar
25 Medical Student
26 Other Community Health Service - Admin Clerk
27 Specialist Nurse Practitioner
28 Receptionist
29 Secretary
30 Medical Secretary
31 Sessional GP
32 Clinical Application Administrator
33 Nurse Consultant
34 Physiotherapist
35 Specialist Practitioner
36 Healthcare Assistant
37 Medical Technical Officer - Pharmacy
38 Health Records Administrator
39 Helpdesk Administrator
40 Appointments Clerk
41 Senior House Officer
42 Social Worker
43 Trainee Practitioner
44 Network Technician
45 Clinical Coder
46 Medical Records Clerk
47 Staff Nurse
48 Enrolled Nurse
49 Multi Therapist
50 Nursery Nurse
51 Helper/Assistant
52 Community Mental Health Nurse
53 Senior Administrator
54 Technician - Healthcare Scientists
55 Associate Practitioner - Nurse
56 Senior Manager
57 Community Administrator
58 Associate Specialist
59 Student Practice Nurse
60 Nurse Manager
61 Sister/Charge Nurse
62 Psychotherapist
63 Osteopath
64 Social Care Support Worker
65 Assistant Psychologist
66 Officer
67 Technician - Admin & Clerical
68 Psychiatrist
69 Health Records Clerk
70 Desktop Support Technician
71 Dispenser
72 Clinical Assistant
73 Practitioner
74 Information Officer
75 Network Administrator
76 Chaplain
77 Student Physiotherapist
78 Paramedic Specialist Practitioner
79 Clinical Team Manager
80 Physiotherapist Specialist Practitioner
81 Helpdesk Technician
82 Radiographer
83 Other Community Health Service
84 Call Operator
85 Community Worker (children)
86 Paramedic Consultant
87 Associate Practitioner
88 Modern Matron
89 Asst. Clinical Medical Officer
90 Community Team Manager
91 Specialist Registrar
92 Chiropodist/Podiatrist Manager
93 Radiographer - Therapeutic, Manager
94 Optometrist
95 Assistant Practitioner
96 Community Learning Disabilities Nurse
97 Technician - Additional Clinical Services
98 Student Health Visitor
99 Interpreter
100 Medical Technical Officer

101 Midwife - Specialist Practitioner
102 Occupational Therapist
103 Chief Executive
104 Audit Manager
105 Paramedic
106 Physiotherapist Consultant
107 Availability Monitor
108 Medical Laboratory Assistant
109 Gateway Worker
110 Medical Records Manager
111 Student Nurse - Adult Branch
112 Audiologist
113 Radiographer - Diagnostic
114 Therapist
115 Student District Nurse
116 House Officer - Post Registration
117 Speech & Language Therapist
118 Dietitian Specialist Practitioner
119 Trust Grade Doctor - SHO level
120 Director of Public Health
121 Staff Grade
122 Patient Welfare Officer
123 Occupational Therapy Specialist Practitioner
124 Technician - PS&T
125 Chiropodist/Podiatrist Consultant
126 Trust Grade Doctor - Career Grade level
127 Student Community Practitioner
128 Healthcare Scientist
129 Waiting List Clerk
130 Clinical Director
131 Pre-reg Pharmacist
132 Mental Health Act Administrator
133 Ward Clerk
134 Support, Time, Recovery Worker
135 Art Therapist Specialist Practitioner
136 Physiotherapist Manager
137 Healthcare Cadet
138 Dietitian Consultant
139 Orthoptist Manager
140 Social work assistant (mental health)
141 Chiropodist/Podiatrist Specialist Practitioner
142 Student Technician
143 Complaints Investigator
144 Trainee Scientist
145 Radiographer - Diagnostic, Manager
146 Social services care manager (mental health)
147 Dietitian Manager
148 Midwife - Consultant
149 Art Therapist Consultant
150 Paramedic Manager
151 Finance Director
152 Senior social worker (adults)
153 Student Midwife
154 Radiologist
155 Ward Manager
156 Midwife - Manager
157 Waiting List Manager
158 Radiographer - Diagnostic, Specialist Practitioner
159 Biomedical Scientist
160 Board Level Director
161 Non Executive Director
162 Nursing Cadet
163 Porter
164 Social services care manager (adults)
165 Student Psychotherapist
166 Orthoptist
167 Clinical Director - Medical
168 Approved Social Worker
169 Student Community Mental Health Nurse
170 Other Executive Director
171 Student Orthoptist
172 Childcare Co-ordinator
173 House Officer - Pre Registration
174 SODP
175 Outpatient Manager
176 Medical Director
177 Trust Grade Doctor - Specialist Registrar level
178 Senior Clinical Medical Officer
179 Consultant Healthcare Scientist
180 Reporting Radiographer
181 Locum GP
182 Researcher
183 Assistant GP
184 Special salary scale in Public Health Medicine
185 Advanced Practitioner
186 Health Visitor
187 Dental Assistant Clinical Director
188 Other Community Health Service - Social Care Worker
189 Physician Assistant
190 Deputising Doctor
191 Student Occupational Health Nurse
192 Senior social worker (mental health)
193 Regional Dental Officer
194 Trainer
195 Cytoscreener
196 Chair
197 Trust Grade Doctor - House Officer level
198 Art Therapist
199 Multi Therapist Specialist Practitioner
200 Drama Therapist
201 Healthcare Science Assistant
202 Social work assistant (adults)
203 Social work team manager (adults)

204 Intermediate Care worker
205 Student Occupational Therapist
206 Student Dietitian
207 Healthcare Science Associate
208 Child Protection worker
209 Professor
210 General Dental Practitioner
211 Student School Nurse
212 Occupational Therapist Consultant
213 Intermediate Care staff
214 Home help
215 Art, Music & Drama Student
216 Specialist Healthcare Scientist
217 Social Services information manager

Table A2.2: Number of observations by EMIS® consultation source identifier, Consultation source code identifier and Staff Job Category, with an indicator for whether it was included as a consultation: Include: 1 = Include, 0 = Exclude, "Excl. job title" = Excluded on the basis of job title.

| Include | Staff Job Category | Consultation source code identifier | EMIS consultation source identifier | Count |
|---|---|---|---|---|
| 1 | gp | gp surgery | gp surgery | 3,119,080 |
| 1 | nurse | gp surgery | gp surgery | 1,692,606 |
| 1 | gp | telephone consultation | telephone consultation | 1,471,946 |
| 1 | other care provider | gp surgery | gp surgery | 977,067 |
| 1 | nurse | telephone consultation | telephone consultation | 196,665 |
| 1 | gp | telephone call to a patient | telephone call to a patient | 108,025 |
| 1 | gp | home visit note | home visit note | 83,600 |
| 1 | other care provider | telephone consultation | telephone consultation | 81,980 |
| 1 | nurse | telephone call to a patient | telephone call to a patient | 32,423 |
| 1 | gp | face to face consultation | face to face consultation | 25,435 |
| 1 | nurse | home visit note | home visit note | 24,174 |
| 1 | other care provider | telephone call to a patient | telephone call to a patient | 23,194 |
| 1 | gp | gp surgery | surgery consultation | 22,756 |

| | | | | |
|---|---|---|---|---|
| 1 | gp | nursing home visit note | nursing home visit note | 21,357 |
| 1 | nurse | face to face consultation | face to face consultation | 17,580 |
| 1 | gp | enterprise consultation | enterprise consultation | 14,904 |
| 1 | gp | telephone call from a patient | telephone call from a patient | 13,062 |
| 1 | gp | routine consultation | surgery consultation | 11,668 |
| 1 | other care provider | home visit note | home visit note | 10,853 |
| 1 | gp | provision of general practitioner intermediate care | gp surgery | 10,441 |
| 1 | gp | emergency consultation | emergency consultation | 10,351 |
| 1 | gp | residential home visit note | residential home visit note | 9,579 |
| 1 | other care provider | face to face consultation | face to face consultation | 9,350 |
| 1 | gp | emergency appointment | emergency appointment | 8,687 |
| 1 | gp | urgent consultation | urgent consultation | 8,155 |
| 1 | gp | walk-in clinic | walk-in clinic | 7,908 |
| 1 | dr | gp surgery | gp surgery | 7,654 |
| 1 | gp | other note | other | 7,520 |
| 1 | gp | face to face consultation | surgery consultation | 6,932 |
| 1 | nurse | gp surgery | surgery consultation | 5,318 |
| 1 | gp | seen in gp unit | surgery consultation | 4,687 |
| 1 | gp | consultation via video conference | awaiting review | 4,653 |
| 1 | nurse | enterprise consultation | enterprise consultation | 4,460 |
| 1 | other care provider | provision of general practitioner intermediate care | gp surgery | 4,369 |
| 1 | gp | clinic note | surgery consultation | 3,823 |
| 1 | nurse | residential home visit note | residential home visit note | 3,612 |
| 1 | nurse | clinic note | clinic | 3,585 |
| 1 | nurse | nursing home visit note | nursing home visit note | 3,528 |
| 1 | nurse | face to face consultation | surgery consultation | 3,442 |
| 1 | gp | online communication | awaiting review | 3,410 |
| 1 | other care provider | other note | other | 3,406 |
| 1 | other care provider | seen in gp unit | gp surgery | 2,781 |
| 1 | gp | e-mail consultation | awaiting review | 2,523 |
| 1 | nurse | other note | other | 2,449 |
| 1 | other care provider | gp surgery | surgery consultation | 2,334 |
| 1 | other care provider | enterprise consultation | enterprise consultation | 2,318 |
| 1 | other care provider | telephone call from a patient | telephone call from a patient | 2,211 |
| 1 | nurse | telephone call from a patient | telephone call from a patient | 2,183 |
| 1 | gp | routine consultation | awaiting review | 2,117 |
| 1 | nurse | emergency appointment | emergency appointment | 2,041 |
| 1 | gp | home visit note | home visit | 2,021 |
| 1 | gp | seen in gp unit | gp surgery | 1,896 |
| 1 | nurse | provision of general practitioner intermediate care | gp surgery | 1,762 |

| | | | | |
|---|---|---|---|---|
| 1 | other care provider | clinic note | clinic | 1,699 |
| 1 | nurse | clinic note | surgery consultation | 1,628 |
| 1 | gp | clinic note | clinic | 1,623 |
| 1 | nurse | routine consultation | surgery consultation | 1,578 |
| 1 | nurse | seen in gp unit | surgery consultation | 1,426 |
| 1 | nurse | walk-in clinic | walk-in clinic | 1,412 |
| 1 | nurse | gp surgery | clinic | 1,355 |
| 1 | other care provider | routine consultation | other | 1,303 |
| 1 | other care provider | clinic note | surgery consultation | 1,297 |
| 1 | gp | face to face consultation | emergency consultation | 1,292 |
| 1 | other care provider | walk-in clinic | walk-in clinic | 1,216 |
| 1 | gp | telephone encounter | telephone encounter | 1,184 |
| 1 | gp | online communication | online services message | 1,139 |
| 1 | gp | other consultation medium used | awaiting review | 1,134 |
| 1 | other care provider | residential home visit note | residential home visit note | 1,113 |
| 1 | nurse | seen in gp unit | gp surgery | 1,103 |
| 1 | other care provider | nursing home visit note | nursing home visit note | 1,081 |
| 1 | other care provider | face to face consultation | surgery consultation | 1,045 |
| 1 | other care provider | seen in gp unit | surgery consultation | 1,043 |
| 1 | nurse | emergency consultation | emergency consultation | 1,024 |
| 1 | nurse | urgent consultation | urgent consultation | 959 |
| 1 | gp | extended hours consultation | awaiting review | 924 |
| 1 | gp | routine consultation | other | 922 |
| 1 | gp | home visit note | other | 835 |
| 1 | gp | gp surgery | face to face consultation | 808 |
| 1 | other care provider | gp surgery | clinic | 746 |
| 1 | other care provider | routine consultation | surgery consultation | 738 |
| 1 | gp | consultation via multimedia | awaiting review | 734 |
| 1 | gp | face to face consultation with relative/carer | awaiting review | 669 |
| 1 | nurse | e-mail consultation | awaiting review | 638 |
| 1 | nurse | routine consultation | awaiting review | 574 |
| 1 | nurse | consultation via video conference | awaiting review | 505 |
| 1 | nurse | routine consultation | other | 478 |
| 1 | other care provider | online communication | awaiting review | 473 |
| 1 | nurse | same day appointment | same day appointment | 468 |
| 1 | gp | face to face consultation | gp surgery | 461 |
| 1 | gp | same day appointment | same day appointment | 457 |
| 1 | nurse | gp surgery | face to face consultation | 455 |
| 1 | gp | telephone encounter | telephone consultation | 429 |
| 1 | nurse | face to face consultation | emergency consultation | 420 |

| | | | | |
|---|---|---|---|---|
| 1 | gp | group consultation | awaiting review | 402 |
| 1 | nurse | home visit note | other | 402 |
| 1 | nurse | seen in urgent care centre | awaiting review | 344 |
| 1 | gp | face to face consultation | emergency appointment | 330 |
| 1 | gp | seen in urgent care centre | awaiting review | 322 |
| 1 | nurse | telephone encounter | telephone encounter | 314 |
| 1 | other care provider | consultation via video conference | awaiting review | 288 |
| 1 | gp | telephone consultation | telephone call to a patient | 287 |
| 1 | dr | telephone consultation | telephone consultation | 279 |
| 1 | nurse | online communication | awaiting review | 279 |
| 1 | other care provider | face to face consultation | awaiting review | 272 |
| 1 | other care provider | home visit note | home visit | 262 |
| 1 | other care provider | routine consultation | awaiting review | 260 |
| 1 | nurse | home visit note | home visit | 246 |
| 1 | gp | consultation via sms text message | awaiting review | 244 |
| 1 | other care provider | online communication | online services message | 241 |
| 1 | other care provider | emergency consultation | emergency consultation | 235 |
| 1 | other care provider | emergency appointment | emergency appointment | 231 |
| 1 | gp | telephone encounter | telephone call to a patient | 226 |
| 1 | gp | residential home visit note | residential home | 225 |
| 1 | gp | face to face consultation | awaiting review | 224 |
| 1 | nurse | face to face consultation | gp surgery | 221 |
| 1 | other care provider | gp surgery | face to face consultation | 209 |
| 1 | nurse | extended hours consultation | awaiting review | 186 |
| 1 | gp | seen in rapid access clinic at gp surgery | awaiting review | 182 |
| 1 | gp | school visit note | awaiting review | 181 |
| 1 | other care provider | telephone consultation | telephone call to a patient | 179 |
| 1 | nurse | face to face consultation | awaiting review | 166 |
| 1 | gp | home visit note | acute visit | 153 |
| 1 | nurse | online communication | online services message | 145 |
| 1 | other care provider | group consultation | awaiting review | 133 |
| 1 | gp | seen in gp unit | awaiting review | 129 |
| 1 | dr | clinic note | surgery consultation | 125 |
| 1 | other care provider | telephone encounter | telephone encounter | 119 |
| 1 | other care provider | extended hours consultation | awaiting review | 116 |
| 1 | nurse | face to face consultation with relative/carer | awaiting review | 115 |
| 1 | nurse | seen in gp unit | awaiting review | 109 |
| 1 | gp | night visit note | awaiting review | 108 |

| | | | | |
|---|---|---|---|---|
| 1 | nurse | school visit note | awaiting review | 107 |
| 1 | nurse | group consultation | awaiting review | 102 |
| 1 | gp | other consultation medium used | other | 99 |
| 1 | gp | children's home visit note | awaiting review | 95 |
| 1 | gp | telephone consultation | telephone call from a patient | 94 |
| 1 | gp | administration note | other | 91 |
| 1 | nurse | telephone encounter | telephone call to a patient | 86 |
| 1 | other care provider | e-mail consultation | awaiting review | 71 |
| 1 | nurse | consultation via multimedia | awaiting review | 65 |
| 1 | gp | gp surgery | clinic | 63 |
| 1 | nurse | telephone consultation | telephone call to a patient | 63 |
| 1 | other care provider | face to face consultation with relative/carer | awaiting review | 62 |
| 1 | gp | consultation via telemedicine web camera | awaiting review | 61 |
| 1 | dr | telephone call to a patient | telephone call to a patient | 60 |
| 1 | nurse | laboratory result | clinic | 55 |
| 1 | other care provider | other consultation medium used | awaiting review | 53 |
| 1 | nurse | other consultation medium used | awaiting review | 51 |
| 1 | nurse | district nurse visit | awaiting review | 48 |
| 1 | gp | clinic note | gp surgery | 47 |
| 1 | other care provider | clinic note | gp surgery | 45 |
| 1 | other care provider | urgent consultation | urgent consultation | 42 |
| 1 | other care provider | home visit note | other | 41 |
| 1 | other care provider | laboratory result | clinic | 40 |
| 1 | gp | laboratory result | acute visit | 39 |
| 1 | dr | home visit note | home visit note | 38 |
| 1 | other care provider | telephone encounter | telephone call to a patient | 38 |
| 1 | nurse | telephone encounter | telephone consultation | 33 |
| 1 | other care provider | consultation via sms text message | awaiting review | 28 |
| 1 | gp | walk-in clinic | walk-in centre | 27 |
| 1 | nurse | walk-in clinic | walk-in centre | 27 |
| 1 | other care provider | walk-in clinic | clinic | 27 |
| 1 | nurse | children's home visit note | awaiting review | 25 |
| 1 | gp | gp surgery | main surgery | 24 |
| 1 | nurse | residential home visit note | residential home | 22 |
| 1 | other care provider | seen in urgent care centre | awaiting review | 22 |
| 1 | nurse | night visit note | awaiting review | 20 |
| 1 | other care provider | children's home visit note | awaiting review | 19 |
| 1 | other care provider | consultation via multimedia | awaiting review | 19 |

| | | | | |
|---|---|---|---|---|
| 1 | gp | home visit note | nursing home visit note | 17 |
| 1 | gp | nursing home visit note | nursing home | 17 |
| 1 | gp | residential home visit note | nursing home | 15 |
| 1 | other care provider | face to face consultation | gp surgery | 14 |
| 1 | other care provider | night visit note | awaiting review | 14 |
| 1 | other care provider | other consultation medium used | other | 14 |
| 1 | nurse | clinic note | gp surgery | 13 |
| 1 | nurse | walk-in clinic | clinic | 13 |
| 1 | nurse | home visit note | acute visit | 10 |
| 1 | nurse | consultation via sms text message | awaiting review | < 10 |
| 1 | nurse | consultation via telemedicine web camera | awaiting review | < 10 |
| 1 | dr | consultation via video conference | awaiting review | < 10 |
| 1 | dr | nursing home visit note | nursing home visit note | < 10 |
| 1 | other care provider | home visit note | nursing home visit note | < 10 |
| 1 | dr | other note | other | < 10 |
| 1 | gp | telephone encounter | telephone call from a patient | < 10 |
| 1 | gp | twilight visit note | awaiting review | < 10 |
| 1 | other care provider | laboratory result | acute visit | < 10 |
| 1 | dr | face to face consultation | face to face consultation | < 10 |
| 1 | gp | home visit note | follow-up/routine visit | < 10 |
| 1 | gp | other consultation medium used | casualty attendance | < 10 |
| 1 | other care provider | home visit note | residential home visit note | < 10 |
| 1 | other care provider | other note | gp surgery | < 10 |
| 1 | gp | home visit note | awaiting review | < 10 |
| 1 | gp | non-consultation medication data | awaiting review | < 10 |
| 1 | gp | remote consultation | awaiting review | < 10 |
| 1 | gp | third party consultation | casualty attendance | < 10 |
| 1 | nurse | home visit note | follow-up/routine visit | < 10 |
| 1 | nurse | telephone encounter | telephone call from a patient | < 10 |
| 1 | nurse | third party consultation | casualty attendance | < 10 |
| 1 | dr | enterprise consultation | enterprise consultation | < 10 |
| 1 | dr | telephone call from a patient | telephone call from a patient | < 10 |
| 1 | gp | district nurse visit | awaiting review | < 10 |
| 1 | gp | e-mail received from patient | acute visit | < 10 |
| 1 | gp | hospital outpatient report | casualty attendance | < 10 |
| 1 | gp | joint consultation | awaiting review | < 10 |
| 1 | gp | pharmacy consultation | awaiting review | < 10 |
| 1 | gp | telephone call to a patient | telephone consultation | < 10 |
| 1 | nurse | administration note | other | < 10 |
| 1 | nurse | face to face consultation | emergency appointment | < 10 |
| 1 | other care provider | consultation via telemedicine web camera | awaiting review | < 10 |

| | | | | |
|---|---|---|---|---|
| 1 | other care provider | face to face consultation | emergency appointment | < 10 |
| 1 | other care provider | face to face consultation | emergency consultation | < 10 |
| 1 | other care provider | home visit note | acute visit | < 10 |
| 1 | other care provider | pharmacy consultation | awaiting review | < 10 |
| 1 | other care provider | residential home visit note | residential home | < 10 |
| 1 | other care provider | telephone encounter | telephone consultation | < 10 |
| 1 | dr | group consultation | awaiting review | < 10 |
| 1 | dr | home visit note | acute visit | < 10 |
| 1 | gp | clinic note | follow-up/routine visit | < 10 |
| 1 | gp | emergency consultation | casualty attendance | < 10 |
| 1 | gp | home visit note | nursing home | < 10 |
| 1 | nurse | seen in rapid access clinic at gp surgery | awaiting review | < 10 |
| 1 | nurse | telephone consultation | telephone call from a patient | < 10 |
| 1 | nurse | twilight visit note | awaiting review | < 10 |
| 1 | other care provider | administration note | other | < 10 |
| 1 | other care provider | children's home visit note | clinic | < 10 |
| 1 | other care provider | home visit note | awaiting review | < 10 |
| 1 | other care provider | twilight visit note | awaiting review | < 10 |
| excl. job cat | | gp surgery | gp surgery | 875,291 |
| excl. job cat | | other note | other | 31,332 |
| excl. job cat | | telephone consultation | telephone consultation | 29,455 |
| excl. job cat | | online communication | online services message | 14,055 |
| excl. job cat | | telephone call to a patient | telephone call to a patient | 13,935 |
| excl. job cat | | provision of general practitioner intermediate care | gp surgery | 9,791 |
| excl. job cat | | telephone call from a patient | telephone call from a patient | 7,098 |
| excl. job cat | | seen in gp unit | gp surgery | 6,240 |
| excl. job cat | | home visit note | home visit note | 4,776 |
| excl. job cat | | routine consultation | other | 4,248 |
| excl. job cat | | gp surgery | surgery consultation | 3,047 |
| excl. job cat | | home visit note | other | 2,664 |
| excl. job cat | | face to face consultation | face to face consultation | 2,071 |
| excl. job cat | | face to face consultation | surgery consultation | 1,177 |
| excl. job cat | | online communication | awaiting review | 1,098 |
| excl. job cat | | nursing home visit note | nursing home visit note | 708 |
| excl. job cat | | routine consultation | surgery consultation | 509 |
| excl. job cat | | consultation via video conference | awaiting review | 494 |
| excl. job cat | | routine consultation | awaiting review | 453 |
| excl. job cat | | enterprise consultation | enterprise consultation | 398 |
| excl. job cat | | administration note | other | 376 |
| excl. job cat | | telephone encounter | telephone encounter | 376 |

| | | | | |
|---|---|---|---|---|
| excl. job cat | | home visit note | awaiting review | 371 |
| excl. job cat | | clinic note | clinic | 337 |
| excl. job cat | | clinic note | surgery consultation | 329 |
| excl. job cat | | residential home visit note | residential home visit note | 327 |
| excl. job cat | | face to face consultation | gp surgery | 305 |
| excl. job cat | | face to face consultation | awaiting review | 293 |
| excl. job cat | | home visit note | home visit | 250 |
| excl. job cat | | urgent consultation | urgent consultation | 240 |
| excl. job cat | | walk-in clinic | walk-in clinic | 196 |
| excl. job cat | | group consultation | awaiting review | 170 |
| excl. job cat | | seen in gp unit | surgery consultation | 148 |
| excl. job cat | | e-mail consultation | awaiting review | 145 |
| excl. job cat | | emergency consultation | emergency consultation | 121 |
| excl. job cat | | face to face consultation with relative/carer | awaiting review | 116 |
| excl. job cat | | consultation via multimedia | awaiting review | 75 |
| excl. job cat | | seen in gp unit | awaiting review | 75 |
| excl. job cat | | children's home visit note | awaiting review | 63 |
| excl. job cat | | other note | gp surgery | 54 |
| excl. job cat | | other consultation medium used | other | 44 |
| excl. job cat | | other consultation medium used | awaiting review | 42 |
| excl. job cat | | extended hours consultation | awaiting review | 37 |
| excl. job cat | | gp surgery | face to face consultation | 36 |
| excl. job cat | | emergency appointment | emergency appointment | 35 |
| excl. job cat | | gp surgery | clinic | 32 |
| excl. job cat | | face to face consultation | emergency consultation | 25 |
| excl. job cat | | residential home visit note | residential home | 23 |
| excl. job cat | | telephone encounter | telephone call to a patient | 23 |
| excl. job cat | | night visit note | awaiting review | 14 |
| excl. job cat | | home visit note | acute visit | 13 |
| excl. job cat | | walk-in clinic | walk-in centre | 11 |
| excl. job cat | | district nurse visit | awaiting review | < 10 |
| excl. job cat | | seen in urgent care centre | awaiting review | < 10 |
| excl. job cat | | twilight visit note | awaiting review | < 10 |
| excl. job cat | | clinic note | gp surgery | < 10 |
| excl. job cat | | laboratory result | acute visit | < 10 |
| excl. job cat | | telephone consultation | telephone call to a patient | < 10 |
| excl. job cat | | walk-in clinic | clinic | < 10 |
| excl. job cat | | telephone encounter | telephone call from a patient | < 10 |
| excl. job cat | | emergency consultation | casualty attendance | < 10 |
| excl. job cat | | pharmacy consultation | awaiting review | < 10 |
| excl. job cat | | third party consultation | casualty attendance | < 10 |
| excl. job cat | | case conference | gp surgery | < 10 |
| excl. job cat | | emergency consultation | awaiting review | < 10 |
| excl. job cat | | gp surgery | main surgery | < 10 |
| excl. job cat | | home visit note | follow-up/routine visit | < 10 |
| excl. job cat | | non-consultation medication data | casualty attendance | < 10 |
| excl. job cat | | nursing home visit note | nursing home | < 10 |

| | | | | |
|---|---|---|---|---|
| excl. job cat | | remote consultation | awaiting review | < 10 |
| excl. job cat | | same day appointment | same day appointment | < 10 |
| excl. job cat | | school visit note | awaiting review | < 10 |
| excl. job cat | | telephone consultation | awaiting review | < 10 |
| excl. job cat | | telephone encounter | telephone consultation | < 10 |
| excl. job cat | | third party consultation | awaiting review | < 10 |
| 0 | gp | externally entered note | externally entered | 3,467,397 |
| 0 | | scanned document | docman | 3,183,781 |
| 0 | | administration note | administration note | 968,767 |
| 0 | | | | 737,843 |
| 0 | | scanned document | scanned document | 727,269 |
| 0 | gp | administration note | administration note | 725,612 |
| 0 | | inbound document | inbound document | 402,647 |
| 0 | | awaiting clinical code migration to emis web | awaiting review | 385,598 |
| 0 | nurse | externally entered note | externally entered | 303,830 |
| 0 | gp | awaiting clinical code migration to emis web | awaiting review | 261,627 |
| 0 | | administration note | administration | 256,604 |
| 0 | other care provider | scanned document | docman | 255,022 |
| 0 | | externally entered note | externally entered | 252,167 |
| 0 | gp | | | 208,462 |
| 0 | gp | outbound referral | outbound referral | 197,534 |
| 0 | gp | awaiting clinical code migration to emis web | gp surgery | 162,048 |
| 0 | other care provider | administration note | administration note | 160,918 |
| 0 | | awaiting clinical code migration to emis web | third party consultation | 147,374 |
| 0 | gp | scanned document | scanned document | 145,355 |
| 0 | | awaiting clinical code migration to emis web | gp surgery | 127,041 |
| 0 | gp | awaiting clinical code migration to emis web | results recording | 122,118 |
| 0 | | awaiting clinical code migration to emis web | other | 121,401 |
| 0 | gp | awaiting clinical code migration to emis web | surgery consultation | 107,304 |
| 0 | | awaiting clinical code migration to emis web | | 104,693 |
| 0 | gp | inbound document | inbound document | 102,534 |
| 0 | nurse | awaiting clinical code migration to emis web | awaiting review | 94,158 |
| 0 | other care provider | awaiting clinical code migration to emis web | gp surgery | 86,398 |
| 0 | other care provider | awaiting clinical code migration to emis web | awaiting review | 85,745 |
| 0 | nurse | administration note | administration note | 84,051 |
| 0 | gp | awaiting clinical code migration to emis web | | 77,585 |
| 0 | nurse | awaiting clinical code migration to emis web | gp surgery | 75,552 |

| | | | | |
|---|---|---|---|---|
| 0 | other care provider | | | 66,366 |
| 0 | other care provider | externally entered note | externally entered | 62,810 |
| 0 | | externally entered note | externally entered note | 61,848 |
| 0 | | awaiting clinical code migration to emis web | repeat issue | 61,683 |
| 0 | nurse | | | 58,019 |
| 0 | gp | telephone triage encounter | telephone triage encounter | 50,662 |
| 0 | | third party consultation | third party consultation | 44,609 |
| 0 | other care provider | scanned document | scanned document | 38,341 |
| 0 | nurse | awaiting clinical code migration to emis web | | 35,376 |
| 0 | nurse | awaiting clinical code migration to emis web | surgery consultation | 27,699 |
| 0 | gp | administration note | administration | 27,390 |
| 0 | gp | awaiting clinical code migration to emis web | telephone consultation | 26,214 |
| 0 | gp | awaiting clinical code migration to emis web | other | 26,204 |
| 0 | | awaiting clinical code migration to emis web | scanned document | 25,627 |
| 0 | | awaiting clinical code migration to emis web | administration note | 24,458 |
| 0 | | gp surgery | awaiting review | 24,337 |
| 0 | | awaiting clinical code migration to emis web | touchscreen | 24,311 |
| 0 | gp | third party consultation | third party consultation | 23,952 |
| 0 | | awaiting clinical code migration to emis web | patientchase insert | 22,515 |
| 0 | gp | laboratory result | results recording | 22,066 |
| 0 | gp | externally entered note | externally entered note | 20,864 |
| 0 | gp | awaiting clinical code migration to emis web | administration note | 20,729 |
| 0 | other care provider | inbound document | inbound document | 20,159 |
| 0 | gp | telephone call to relative/carer | telephone call to relative/carer | 19,234 |
| 0 | other care provider | awaiting clinical code migration to emis web | | 17,081 |
| 0 | | awaiting clinical code migration to emis web | surgery consultation | 15,972 |
| 0 | nurse | awaiting clinical code migration to emis web | clinic | 15,892 |
| 0 | gp | other note | other note | 15,816 |
| 0 | gp | gp surgery | awaiting review | 15,810 |
| 0 | gp | discussion with colleague | discussion with colleague | 12,973 |
| 0 | gp | awaiting clinical code migration to emis web | telephone call to a patient | 11,782 |
| 0 | | repeat prescription | repeat issue | 11,542 |
| 0 | other care provider | administration note | administration | 11,037 |
| 0 | nurse | awaiting clinical code migration to emis web | other | 11,008 |

| | | | | |
|---|---|---|---|---|
| 0 | | hospital outpatient report | hospital outpatient report | 10,033 |
| 0 | nurse | inbound document | inbound document | 9,304 |
| 0 | nurse | scanned document | scanned document | 7,878 |
| 0 | other care provider | awaiting clinical code migration to emis web | repeat issue | 7,745 |
| 0 | gp | awaiting clinical code migration to emis web | outbound referral | 7,402 |
| 0 | other care provider | externally entered note | externally entered note | 6,736 |
| 0 | gp | telephone call from relative/carer | telephone call from relative/carer | 6,626 |
| 0 | other care provider | awaiting clinical code migration to emis web | surgery consultation | 6,514 |
| 0 | | awaiting clinical code migration to emis web | inbound document | 6,495 |
| 0 | nurse | gp surgery | awaiting review | 6,475 |
| 0 | other care provider | awaiting clinical code migration to emis web | other | 6,315 |
| 0 | nurse | administration note | administration | 6,285 |
| 0 | | outbound referral | outbound referral | 6,194 |
| 0 | nurse | telephone triage encounter | telephone triage encounter | 6,074 |
| 0 | gp | clinic note | clinic note | 5,834 |
| 0 | gp | awaiting clinical code migration to emis web | repeat issue | 5,803 |
| 0 | gp | non-consultation data | non-consultation data | 5,552 |
| 0 | other care provider | third party consultation | third party consultation | 5,505 |
| 0 | other care provider | gp surgery | awaiting review | 5,332 |
| 0 | gp | awaiting clinical code migration to emis web | telephone call from a patient | 5,282 |
| 0 | nurse | externally entered note | externally entered note | 5,251 |
| 0 | gp | face to face consultation | triage | 5,196 |
| 0 | nurse | telephone call to relative/carer | telephone call to relative/carer | 5,191 |
| 0 | | awaiting clinical code migration to emis web | mail to patient | 5,178 |
| 0 | | non-consultation data | non-consultation data | 4,933 |
| 0 | gp | e-mail received from patient | e-mail received from patient | 4,877 |
| 0 | nurse | outbound referral | outbound referral | 4,787 |
| 0 | | medication requested | repeat issue | 4,639 |
| 0 | nurse | third party consultation | third party consultation | 4,605 |
| 0 | nurse | awaiting clinical code migration to emis web | results recording | 4,405 |
| 0 | gp | awaiting clinical code migration to emis web | scanned document | 4,384 |
| 0 | | other note | other note | 4,375 |
| 0 | | awaiting clinical code migration to emis web | mjog | 4,221 |
| 0 | other care provider | other note | other note | 4,071 |
| 0 | | mail to patient | mail to patient | 3,924 |
| 0 | other care provider | awaiting clinical code migration to emis web | clinic | 3,859 |
| 0 | | e-mail received from patient | e-mail received from patient | 3,632 |

| | | | | |
|---|---|---|---|---|
| 0 | | awaiting clinical code migration to emis web | out of hours, non practice | 3,607 |
| 0 | other care provider | clinic note | clinic note | 3,371 |
| 0 | gp | awaiting clinical code migration to emis web | home visit note | 3,370 |
| 0 | gp | hospital outpatient report | hospital outpatient report | 3,359 |
| 0 | nurse | nurse telephone triage | nurse telephone triage | 3,274 |
| 0 | nurse | clinic note | clinic note | 3,252 |
| 0 | gp | awaiting clinical code migration to emis web | clinic | 3,245 |
| 0 | other care provider | awaiting clinical code migration to emis web | administration note | 3,044 |
| 0 | gp | awaiting clinical code migration to emis web | third party consultation | 3,038 |
| 0 | other care provider | awaiting clinical code migration to emis web | medicine management | 2,829 |
| 0 | other care provider | telephone call to relative/carer | telephone call to relative/carer | 2,807 |
| 0 | nurse | awaiting clinical code migration to emis web | telephone consultation | 2,782 |
| 0 | | awaiting clinical code migration to emis web | clinic | 2,775 |
| 0 | | laboratory result | laboratory result | 2,727 |
| 0 | gp | awaiting clinical code migration to emis web | inbound document | 2,605 |
| 0 | | telephone call from relative/carer | telephone call from relative/carer | 2,594 |
| 0 | | clinic note | clinic note | 2,579 |
| 0 | gp | laboratory result | laboratory result | 2,511 |
| 0 | other care provider | repeat prescription | repeat issue | 2,511 |
| 0 | | awaiting clinical code migration to emis web | home of patient | 2,454 |
| 0 | gp | awaiting clinical code migration to emis web | out of hours, non practice | 2,365 |
| 0 | gp | awaiting clinical code migration to emis web | nhs direct report | 2,334 |
| 0 | | awaiting clinical code migration to emis web | results recording | 2,185 |
| 0 | dr | third party consultation | third party consultation | 2,146 |
| 0 | other care provider | awaiting clinical code migration to emis web | third party consultation | 2,095 |
| 0 | nurse | awaiting clinical code migration to emis web | administration note | 2,075 |
| 0 | | administration note | patientchase insert | 1,855 |
| 0 | | clinic note | awaiting review | 1,790 |
| 0 | | hospital inpatient report | hospital inpatient report | 1,760 |
| 0 | | e-mail received from patient | docman | 1,753 |
| 0 | nurse | other note | other note | 1,708 |
| 0 | nurse | non-consultation data | non-consultation data | 1,681 |
| 0 | gp | awaiting clinical code migration to emis web | referral letter | 1,660 |
| 0 | | laboratory result | results recording | 1,624 |

| | | | | |
|---|---|---|---|---|
| 0 | | awaiting clinical code migration to emis web | non-consultation data | 1,537 |
| 0 | gp | awaiting clinical code migration to emis web | medicine management | 1,510 |
| 0 | nurse | laboratory result | results recording | 1,464 |
| 0 | nurse | telephone call from relative/carer | telephone call from relative/carer | 1,401 |
| 0 | | administration note | scanned document | 1,373 |
| 0 | | telephone triage encounter | telephone triage encounter | 1,353 |
| 0 | gp | medication requested | awaiting review | 1,347 |
| 0 | nurse | discussion with colleague | discussion with colleague | 1,329 |
| 0 | gp | awaiting clinical code migration to emis web | telephone triage encounter | 1,313 |
| 0 | nurse | awaiting clinical code migration to emis web | telephone call to a patient | 1,243 |
| 0 | other care provider | non-consultation data | non-consultation data | 1,239 |
| 0 | gp | case conference | awaiting review | 1,225 |
| 0 | gp | awaiting clinical code migration to emis web | externally entered note | 1,206 |
| 0 | | telephone call to relative/carer | telephone call to relative/carer | 1,191 |
| 0 | nurse | awaiting clinical code migration to emis web | third party consultation | 1,170 |
| 0 | gp | e-mail sent to patient | awaiting review | 1,162 |
| 0 | dr | administration note | administration | 1,159 |
| 0 | | other note | | 1,156 |
| 0 | | administration note | | 1,120 |
| 0 | dr | externally entered note | externally entered | 1,113 |
| 0 | gp | awaiting clinical code migration to emis web | face to face consultation | 1,112 |
| 0 | other care provider | telephone triage encounter | telephone triage encounter | 1,107 |
| 0 | nurse | laboratory result | laboratory result | 1,064 |
| 0 | other care provider | outbound referral | outbound referral | 1,052 |
| 0 | gp | clinic note | awaiting review | 1,030 |
| 0 | other care provider | mail to patient | patientchase insert | 1,019 |
| 0 | gp | repeat prescription | awaiting review | 1,003 |
| 0 | gp | awaiting clinical code migration to emis web | data transferred from other system | 983 |
| 0 | | awaiting clinical code migration to emis web | non-consultation medication data | 980 |
| 0 | gp | awaiting clinical code migration to emis web | nursing home visit note | 971 |
| 0 | gp | repeat prescription | repeat issue | 964 |
| 0 | | awaiting clinical code migration to emis web | telephone call to a patient | 901 |
| 0 | dr | | | 885 |
| 0 | nurse | awaiting clinical code migration to emis web | face to face consultation | 853 |
| 0 | other care provider | hospital outpatient report | hospital outpatient report | 828 |
| 0 | | clinic note | community clinic | 823 |

| | | | | |
|---|---|---|---|---|
| 0 | | awaiting clinical code migration to emis web | gp2gp import | 822 |
| 0 | | awaiting clinical code migration to emis web | letter from outpatients | 809 |
| 0 | other care provider | mail to patient | mail to patient | 801 |
| 0 | other care provider | telephone call from relative/carer | telephone call from relative/carer | 789 |
| 0 | | awaiting clinical code migration to emis web | externally entered note | 769 |
| 0 | | awaiting clinical code migration to emis web | referral letter | 751 |
| 0 | other care provider | awaiting clinical code migration to emis web | inbound document | 719 |
| 0 | | e-mail sent to patient | awaiting review | 711 |
| 0 | gp | hospital inpatient report | hospital inpatient report | 710 |
| 0 | nurse | awaiting clinical code migration to emis web | repeat issue | 694 |
| 0 | gp | awaiting clinical code migration to emis web | acute visit | 693 |
| 0 | gp | awaiting clinical code migration to emis web | telephone call to relative/carer | 680 |
| 0 | | non-consultation medication data | repeat issue | 679 |
| 0 | nurse | awaiting clinical code migration to emis web | home visit note | 672 |
| 0 | other care provider | discussion with colleague | discussion with colleague | 665 |
| 0 | | administration note | docman | 665 |
| 0 | gp | other note | | 661 |
| 0 | | awaiting clinical code migration to emis web | telephone consultation | 656 |
| 0 | other care provider | awaiting clinical code migration to emis web | scanned document | 642 |
| 0 | | ooh report | third party consultation | 637 |
| 0 | gp | scanned document | externally entered | 619 |
| 0 | gp | medication requested | repeat issue | 606 |
| 0 | nurse | awaiting clinical code migration to emis web | telephone triage encounter | 602 |
| 0 | other care provider | awaiting clinical code migration to emis web | telephone call to a patient | 541 |
| 0 | other care provider | awaiting clinical code migration to emis web | face to face consultation | 534 |
| 0 | dr | administration note | administration note | 525 |
| 0 | gp | discussion with other professional | awaiting review | 514 |
| 0 | gp | administration note | awaiting review | 509 |
| 0 | other care provider | awaiting clinical code migration to emis web | mail to patient | 507 |
| 0 | | ooh report | awaiting review | 504 |
| 0 | dr | awaiting clinical code migration to emis web | gp surgery | 501 |
| 0 | | walk-in clinic | | 495 |
| 0 | | administration note | inbound document | 493 |
| 0 | | routine consultation | repeat issue | 488 |

| | | | | |
|---|---|---|---|---|
| 0 | | awaiting clinical code migration to emis web | telephone call from a patient | 486 |
| 0 | gp | ooh report | out of hours, non practice | 478 |
| 0 | gp | mail from patient | mail from patient | 473 |
| 0 | | other note | awaiting review | 472 |
| 0 | gp | awaiting clinical code migration to emis web | enterprise consultation | 458 |
| 0 | other care provider | repeat prescription | awaiting review | 455 |
| 0 | gp | awaiting clinical code migration to emis web | discussion with colleague | 451 |
| 0 | | awaiting clinical code migration to emis web | outbound referral | 450 |
| 0 | gp | awaiting clinical code migration to emis web | nursing home | 447 |
| 0 | dr | awaiting clinical code migration to emis web | awaiting review | 444 |
| 0 | nurse | e-mail received from patient | e-mail received from patient | 443 |
| 0 | | awaiting clinical code migration to emis web | data transferred from other system | 442 |
| 0 | nurse | ooh report | awaiting review | 439 |
| 0 | | administration note | awaiting review | 436 |
| 0 | other care provider | clinic note | awaiting review | 431 |
| 0 | gp | administration note | | 415 |
| 0 | gp | administration note | scanned document | 414 |
| 0 | gp | awaiting clinical code migration to emis web | urgent consultation | 404 |
| 0 | other care provider | awaiting clinical code migration to emis web | externally entered note | 385 |
| 0 | | awaiting clinical code migration to emis web | school | 384 |
| 0 | nurse | hospital outpatient report | hospital outpatient report | 380 |
| 0 | | mail to patient | patientchase insert | 377 |
| 0 | | awaiting clinical code migration to emis web | telephone encounter | 376 |
| 0 | gp | administration note | results recording | 372 |
| 0 | | discussion with colleague | discussion with colleague | 368 |
| 0 | other care provider | awaiting clinical code migration to emis web | results recording | 365 |
| 0 | | case conference | awaiting review | 361 |
| 0 | other care provider | awaiting clinical code migration to emis web | telephone consultation | 350 |
| 0 | other care provider | awaiting clinical code migration to emis web | patientchase insert | 345 |
| 0 | | sms text message sent to patient | patientchase insert | 345 |
| 0 | nurse | mail to patient | mail to patient | 342 |
| 0 | | awaiting clinical code migration to emis web | hospital outpatient report | 336 |
| 0 | | gp surgery | | 315 |
| 0 | gp | ooh report | nhs direct report | 314 |
| 0 | gp | administration note | referral letter | 313 |
| 0 | dr | awaiting clinical code migration to emis web | | 310 |

| | | | | |
|---|---|---|---|---|
| 0 | gp | awaiting clinical code migration to emis web | telephone call from relative/carer | 310 |
| 0 | gp | awaiting clinical code migration to emis web | non-consultation data | 309 |
| 0 | nurse | awaiting clinical code migration to emis web | telephone call from a patient | 309 |
| 0 | other care provider | awaiting clinical code migration to emis web | non-consultation data | 306 |
| 0 | other care provider | e-mail received from patient | e-mail received from patient | 292 |
| 0 | other care provider | awaiting clinical code migration to emis web | non-consultation medication data | 289 |
| 0 | nurse | clinic note | awaiting review | 286 |
| 0 | dr | scanned document | scanned document | 285 |
| 0 | | ooh report | out of hours, non practice | 283 |
| 0 | other care provider | laboratory result | laboratory result | 282 |
| 0 | gp | awaiting clinical code migration to emis web | discharge details | 281 |
| 0 | | home visit note | home of patient | 277 |
| 0 | nurse | awaiting clinical code migration to emis web | inbound document | 276 |
| 0 | other care provider | hospital inpatient report | hospital inpatient report | 275 |
| 0 | gp | mail to patient | mail to patient | 270 |
| 0 | gp | awaiting clinical code migration to emis web | residential home visit note | 266 |
| 0 | other care provider | administration note | awaiting review | 265 |
| 0 | | awaiting clinical code migration to emis web | acute visit | 263 |
| 0 | | walk-in clinic | awaiting review | 260 |
| 0 | dr | hospital outpatient report | hospital outpatient report | 256 |
| 0 | gp | awaiting clinical code migration to emis web | mail to patient | 249 |
| 0 | | administration note | mjog | 248 |
| 0 | | mail from patient | mail from patient | 248 |
| 0 | gp | awaiting clinical code migration to emis web | letter from outpatients | 247 |
| 0 | | awaiting clinical code migration to emis web | discharge details | 244 |
| 0 | nurse | awaiting clinical code migration to emis web | mail to patient | 243 |
| 0 | other care provider | awaiting clinical code migration to emis web | home visit note | 236 |
| 0 | gp | home visit note | results recording | 235 |
| 0 | dr | inbound document | inbound document | 234 |
| 0 | gp | awaiting clinical code migration to emis web | other note | 230 |
| 0 | gp | awaiting clinical code migration to emis web | patientchase insert | 229 |
| 0 | dr | awaiting clinical code migration to emis web | administration note | 225 |
| 0 | gp | awaiting clinical code migration to emis web | open door surgery | 222 |

| | | | | |
|---|---|---|---|---|
| 0 | | awaiting clinical code migration to emis web | nhs direct report | 222 |
| 0 | other care provider | awaiting clinical code migration to emis web | touchscreen | 216 |
| 0 | gp | scanned document | docman | 213 |
| 0 | nurse | awaiting clinical code migration to emis web | nursing home visit note | 210 |
| 0 | nurse | administration note | | 209 |
| 0 | nurse | awaiting clinical code migration to emis web | externally entered note | 209 |
| 0 | | awaiting clinical code migration to emis web | laboratory result | 199 |
| 0 | | hospital outpatient report | hospital | 197 |
| 0 | other care provider | non-consultation medication data | repeat issue | 196 |
| 0 | other care provider | awaiting clinical code migration to emis web | referral letter | 195 |
| 0 | nurse | e-mail sent to patient | awaiting review | 191 |
| 0 | nurse | awaiting clinical code migration to emis web | referral letter | 190 |
| 0 | dr | awaiting clinical code migration to emis web | telephone consultation | 186 |
| 0 | nurse | case conference | awaiting review | 185 |
| 0 | other care provider | awaiting clinical code migration to emis web | data transferred from other system | 184 |
| 0 | gp | multidisciplinary team meeting without patient | awaiting review | 179 |
| 0 | gp | awaiting clinical code migration to emis web | clinic note | 176 |
| 0 | other care provider | awaiting clinical code migration to emis web | mjog | 175 |
| 0 | | awaiting clinical code migration to emis web | hospital inpatient report | 168 |
| 0 | | awaiting clinical code migration to emis web | home visit note | 164 |
| 0 | | awaiting clinical code migration to emis web | online services message | 163 |
| 0 | other care provider | administration note | | 162 |
| 0 | nurse | awaiting clinical code migration to emis web | outbound referral | 159 |
| 0 | other care provider | ooh report | awaiting review | 156 |
| 0 | gp | awaiting clinical code migration to emis web | non-consultation medication data | 152 |
| 0 | nurse | awaiting clinical code migration to emis web | telephone call to relative/carer | 152 |
| 0 | other care provider | other note | awaiting review | 149 |
| 0 | dr | awaiting clinical code migration to emis web | surgery consultation | 148 |
| 0 | nurse | awaiting clinical code migration to emis web | scanned document | 146 |
| 0 | gp | administration note | repeat issue | 145 |
| 0 | | awaiting clinical code migration to emis web | open door surgery | 144 |

| | | | | |
|---|---|---|---|---|
| 0 | other care provider | awaiting clinical code migration to emis web | clinic note | 140 |
| 0 | | inbound referral | awaiting review | 138 |
| 0 | | awaiting clinical code migration to emis web | clinic note | 133 |
| 0 | | awaiting clinical code migration to emis web | face to face consultation | 129 |
| 0 | other care provider | non-consultation medication data | non-consultation medication data | 124 |
| 0 | nurse | awaiting clinical code migration to emis web | residential home visit note | 122 |
| 0 | nurse | awaiting clinical code migration to emis web | data transferred from other system | 121 |
| 0 | other care provider | e-mail sent to patient | awaiting review | 121 |
| 0 | gp | administration note | outbound referral | 118 |
| 0 | nurse | other note | | 117 |
| 0 | nurse | awaiting clinical code migration to emis web | nurse telephone triage | 116 |
| 0 | | awaiting clinical code migration to emis web | accident & emergency | 116 |
| 0 | gp | other consultation medium used | data transferred from other system | 109 |
| 0 | | non-consultation medication data | non-consultation medication data | 108 |
| 0 | nurse | face to face consultation | triage | 104 |
| 0 | other care provider | awaiting clinical code migration to emis web | acute visit | 102 |
| 0 | nurse | awaiting clinical code migration to emis web | telephone call from relative/carer | 100 |
| 0 | | extended hours consultation | out of hours, non practice | 100 |
| 0 | nurse | mail from patient | mail from patient | 98 |
| 0 | other care provider | hospital outpatient report | hospital | 97 |
| 0 | dr | awaiting clinical code migration to emis web | nursing home visit note | 96 |
| 0 | | nurse telephone triage | nurse telephone triage | 94 |
| 0 | nurse | hospital inpatient report | hospital inpatient report | 93 |
| 0 | other care provider | case conference | awaiting review | 92 |
| 0 | gp | provision of general practitioner intermediate care | awaiting review | 91 |
| 0 | gp | walk-in clinic | | 91 |
| 0 | | hospital outpatient report | letter from outpatients | 91 |
| 0 | other care provider | other note | | 89 |
| 0 | gp | administration note | other note | 87 |
| 0 | other care provider | medication requested | awaiting review | 85 |
| 0 | dr | laboratory result | results recording | 84 |
| 0 | | awaiting clinical code migration to emis web | mail from patient | 84 |
| 0 | | awaiting clinical code migration to emis web | health centre | 80 |
| 0 | gp | administration note | inbound document | 76 |
| 0 | gp | other note | awaiting review | 76 |

| | | | | |
|---|---|---|---|---|
| 0 | nurse | walk-in clinic | | 76 |
| 0 | | other note | non-consultation medication data | 76 |
| 0 | other care provider | awaiting clinical code migration to emis web | nhs direct report | 73 |
| 0 | | administration note | repeat issue | 73 |
| 0 | gp | ooh report | awaiting review | 70 |
| 0 | gp | weekly care home ward round | awaiting review | 70 |
| 0 | | awaiting clinical code migration to emis web | medicine management | 70 |
| 0 | other care provider | awaiting clinical code migration to emis web | open door surgery | 69 |
| 0 | other care provider | awaiting clinical code migration to emis web | telephone encounter | 69 |
| 0 | | medication requested | awaiting review | 68 |
| 0 | other care provider | awaiting clinical code migration to emis web | telephone call to relative/carer | 65 |
| 0 | dr | awaiting clinical code migration to emis web | repeat issue | 64 |
| 0 | gp | non-consultation medication data | repeat issue | 64 |
| 0 | other care provider | awaiting clinical code migration to emis web | telephone call from a patient | 64 |
| 0 | gp | clinic note | nhs direct report | 62 |
| 0 | gp | non-consultation medication data | medicine management | 61 |
| 0 | other care provider | awaiting clinical code migration to emis web | letter from outpatients | 61 |
| 0 | gp | hospital outpatient report | letter from outpatients | 60 |
| 0 | gp | non-consultation data | data transferred from other system | 57 |
| 0 | nurse | awaiting clinical code migration to emis web | enterprise consultation | 57 |
| 0 | gp | outbound referral | referral letter | 55 |
| 0 | other care provider | repeat prescription | medicine management | 55 |
| 0 | nurse | seen in influenza vaccination clinic | awaiting review | 54 |
| 0 | | administration note | results recording | 54 |
| 0 | | awaiting clinical code migration to emis web | telephone call from relative/carer | 54 |
| 0 | gp | awaiting clinical code migration to emis web | hospital outpatient report | 53 |
| 0 | gp | gp surgery | | 53 |
| 0 | gp | third party consultation | out of hours, non practice | 51 |
| 0 | | awaiting clinical code migration to emis web | telephone call to relative/carer | 51 |
| 0 | | clinic note | out of hours, non practice | 51 |
| 0 | | face to face consultation | primary care centre | 51 |
| 0 | gp | awaiting clinical code migration to emis web | laboratory result | 50 |
| 0 | nurse | non-consultation medication data | repeat issue | 48 |
| 0 | | awaiting clinical code migration to emis web | other note | 48 |
| 0 | | face to face consultation | triage | 48 |
| 0 | gp | progress report | nhs direct report | 47 |
| 0 | other care provider | ooh report | nhs direct report | 47 |

| | | | | |
|---|---|---|---|---|
| 0 | nurse | awaiting clinical code migration to emis web | non-consultation data | 46 |
| 0 | | awaiting clinical code migration to emis web | nursing home | 46 |
| 0 | gp | awaiting clinical code migration to emis web | mail from patient | 45 |
| 0 | nurse | awaiting clinical code migration to emis web | discussion with colleague | 45 |
| 0 | | awaiting clinical code migration to emis web | e-mail received from patient | 45 |
| 0 | nurse | awaiting clinical code migration to emis web | medicine management | 43 |
| 0 | nurse | medication requested | repeat issue | 42 |
| 0 | dr | mail to patient | mail to patient | 40 |
| 0 | gp | awaiting clinical code migration to emis web | encompass message | 39 |
| 0 | gp | other note | data transferred from other system | 39 |
| 0 | nurse | awaiting clinical code migration to emis web | clinic note | 39 |
| 0 | nurse | awaiting clinical code migration to emis web | laboratory result | 39 |
| 0 | other care provider | awaiting clinical code migration to emis web | other note | 39 |
| 0 | other care provider | medication requested | repeat issue | 38 |
| 0 | gp | administration note | non-consultation data | 37 |
| 0 | other care provider | mail from patient | mail from patient | 37 |
| 0 | dr | awaiting clinical code migration to emis web | results recording | 36 |
| 0 | gp | awaiting clinical code migration to emis web | e-mail received from patient | 36 |
| 0 | nurse | awaiting clinical code migration to emis web | walk-in centre | 36 |
| 0 | other care provider | awaiting clinical code migration to emis web | gp2gp import | 36 |
| 0 | | administration note | mail to patient | 36 |
| 0 | gp | awaiting clinical code migration to emis web | home of patient | 35 |
| 0 | gp | awaiting clinical code migration to emis web | casualty attendance | 34 |
| 0 | gp | inbound document | letter from outpatients | 34 |
| 0 | other care provider | awaiting clinical code migration to emis web | outbound referral | 34 |
| 0 | other care provider | extended hours consultation | out of hours, non practice | 34 |
| 0 | gp | awaiting clinical code migration to emis web | telephone encounter | 33 |
| 0 | gp | seen in hospital ward | awaiting review | 33 |
| 0 | | other note | non-consultation data | 33 |
| 0 | nurse | other note | awaiting review | 32 |
| 0 | other care provider | awaiting clinical code migration to emis web | discussion with colleague | 32 |
| 0 | | discussion with other professional | awaiting review | 32 |

| | | | | |
|---|---|---|---|---|
| 0 | | multidisciplinary team meeting without patient | awaiting review | 32 |
| 0 | | children's home visit note | | 31 |
| 0 | other care provider | laboratory result | results recording | 30 |
| 0 | | awaiting clinical code migration to emis web | casualty attendance | 30 |
| 0 | | extended hours consultation | nhs direct report | 30 |
| 0 | nurse | administration note | awaiting review | 29 |
| 0 | nurse | awaiting clinical code migration to emis web | letter from outpatients | 29 |
| 0 | nurse | awaiting clinical code migration to emis web | open door surgery | 29 |
| 0 | nurse | discussion with other professional | awaiting review | 29 |
| 0 | nurse | ooh report | out of hours, non practice | 29 |
| 0 | | face to face consultation | treatment room | 29 |
| 0 | gp | email received from carer | awaiting review | 28 |
| 0 | other care provider | awaiting clinical code migration to emis web | telephone triage encounter | 28 |
| 0 | | administration note | touchscreen | 28 |
| 0 | nurse | awaiting clinical code migration to emis web | encompass message | 27 |
| 0 | nurse | awaiting clinical code migration to emis web | nursing home | 27 |
| 0 | | third party consultation | out of hours, non practice | 27 |
| 0 | gp | awaiting clinical code migration to emis web | follow-up/routine visit | 26 |
| 0 | other care provider | emergency consultation | accident & emergency | 26 |
| 0 | gp | hospital outpatient report | nhs direct report | 25 |
| 0 | other care provider | awaiting clinical code migration to emis web | telephone call from relative/carer | 23 |
| 0 | other care provider | outbound referral | referral letter | 23 |
| 0 | | externally entered note | scanned document | 23 |
| 0 | nurse | multidisciplinary team meeting without patient | awaiting review | 22 |
| 0 | | other consultation medium used | data transferred from other system | 22 |
| 0 | dr | awaiting clinical code migration to emis web | outbound referral | 21 |
| 0 | gp | seen in influenza vaccination clinic | awaiting review | 21 |
| 0 | nurse | awaiting clinical code migration to emis web | other note | 21 |
| 0 | | ooh report | nhs direct report | 21 |
| 0 | dr | awaiting clinical code migration to emis web | inbound document | 20 |
| 0 | gp | awaiting clinical code migration to emis web | day case report | 20 |
| 0 | other care provider | seen by general practitioner with special interest in ear nose and throat disorders | data transferred from other system | 20 |
| 0 | | emergency consultation | accident & emergency | 20 |
| 0 | gp | awaiting clinical code migration to emis web | online services message | 19 |

| | | | | |
|---|---|---|---|---|
| 0 | gp | hospital outpatient report | third party consultation | 19 |
| 0 | nurse | repeat prescription | awaiting review | 19 |
| 0 | | awaiting clinical code migration to emis web | walk in centre | 19 |
| 0 | dr | hospital inpatient report | hospital inpatient report | 18 |
| 0 | other care provider | awaiting clinical code migration to emis web | residential home visit note | 18 |
| 0 | other care provider | walk-in clinic | | 18 |
| 0 | | awaiting clinical code migration to emis web | residential home visit note | 18 |
| 0 | | awaiting clinical code migration to emis web | telephone triage encounter | 18 |
| 0 | dr | outbound referral | outbound referral | 17 |
| 0 | gp | ooh report | third party consultation | 17 |
| 0 | gp | other consultation medium used | nhs direct report | 17 |
| 0 | gp | radiology result | awaiting review | 17 |
| 0 | gp | telephone consultation | telephone call from relative/carer | 17 |
| 0 | other care provider | awaiting clinical code migration to emis web | enterprise consultation | 17 |
| 0 | other care provider | walk-in clinic | walk in centre | 17 |
| 0 | | administration note | referral letter | 17 |
| 0 | dr | other note | other note | 16 |
| 0 | other care provider | awaiting clinical code migration to emis web | nursing home visit note | 16 |
| 0 | dr | medication requested | repeat issue | 15 |
| 0 | other care provider | administration note | inbound document | 15 |
| 0 | other care provider | awaiting clinical code migration to emis web | out of hours, non practice | 15 |
| 0 | | non-consultation data | data transferred from other system | 15 |
| 0 | other care provider | face to face consultation | triage | 15 |
| 0 | dr | awaiting clinical code migration to emis web | telephone call to a patient | 14 |
| 0 | dr | externally entered note | externally entered note | 14 |
| 0 | gp | hospital inpatient report | discharge details | 14 |
| 0 | gp | seen by general practitioner with special interest in ear nose and throat disorders | data transferred from other system | 14 |
| 0 | other care provider | administration note | scanned document | 14 |
| 0 | other care provider | discussion with other professional | awaiting review | 14 |
| 0 | | awaiting clinical code migration to emis web | out of hours, practice | 14 |
| 0 | | externally entered note | | 14 |
| 0 | dr | telephone call to relative/carer | telephone call to relative/carer | 13 |
| 0 | nurse | awaiting clinical code migration to emis web | nhs direct report | 13 |
| 0 | nurse | awaiting clinical code migration to emis web | out of hours, non practice | 13 |
| 0 | | hospital inpatient note | awaiting review | 13 |

| | | | | |
|---|---|---|---|---|
| 0 | | repeat prescription | awaiting review | 13 |
| 0 | gp | other note | encompass message | 12 |
| 0 | nurse | awaiting clinical code migration to emis web | hospital outpatient report | 12 |
| 0 | other care provider | awaiting clinical code migration to emis web | accident & emergency | 12 |
| 0 | nurse | other note | encompass message | 11 |
| 0 | nurse | repeat prescription | repeat issue | 11 |
| 0 | other care provider | seen in influenza vaccination clinic | awaiting review | 11 |
| 0 | | awaiting clinical code migration to emis web | discussion with colleague | 11 |
| 0 | | awaiting clinical code migration to emis web | hospital outpatient consultation | 11 |
| 0 | | awaiting clinical code migration to emis web | walk-in clinic | 11 |
| 0 | | non-consultation medication data | medicine management | 11 |
| 0 | | provision of general practitioner intermediate care | awaiting review | 10 |
| 0 | dr | clinic note | awaiting review | < 10 |
| 0 | other care provider | ooh report | out of hours centre | < 10 |
| 0 | | administration note | non-consultation data | < 10 |
| 0 | | clinic note | nhs direct report | < 10 |
| 0 | | telephone follow-up | awaiting review | < 10 |
| 0 | gp | administration note | clinic note | < 10 |
| 0 | gp | progress report | awaiting review | < 10 |
| 0 | nurse | awaiting clinical code migration to emis web | acute visit | < 10 |
| 0 | other care provider | multidisciplinary team meeting without patient | awaiting review | < 10 |
| 0 | | administration note | other note | < 10 |
| 0 | dr | awaiting clinical code migration to emis web | home visit note | < 10 |
| 0 | gp | hospital inpatient note | day case report | < 10 |
| 0 | gp | inbound document | discharge details | < 10 |
| 0 | nurse | awaiting clinical code migration to emis web | follow-up/routine visit | < 10 |
| 0 | other care provider | awaiting clinical code migration to emis web | discharge details | < 10 |
| 0 | | awaiting clinical code migration to emis web | enterprise consultation | < 10 |
| 0 | | awaiting clinical code migration to emis web | nursing home visit note | < 10 |
| 0 | | email received from carer | awaiting review | < 10 |
| 0 | | ooh report | out of hours, practice | < 10 |
| 0 | | other note | data transferred from other system | < 10 |
| 0 | | outbound referral | referral letter | < 10 |
| 0 | | radiology result | awaiting review | < 10 |
| 0 | dr | gp surgery | awaiting review | < 10 |
| 0 | gp | routine consultation | repeat issue | < 10 |
| 0 | nurse | email received from carer | awaiting review | < 10 |
| 0 | nurse | non-consultation data | data transferred from other system | < 10 |

| | | | | |
|---|---|---|---|---|
| 0 | nurse | scanned document | docman | < 10 |
| 0 | other care provider | awaiting clinical code migration to emis web | home of patient | < 10 |
| 0 | other care provider | hospital outpatient report | letter from outpatients | < 10 |
| 0 | dr | discussion with colleague | discussion with colleague | < 10 |
| 0 | gp | administration note | discussion with colleague | < 10 |
| 0 | gp | awaiting clinical code migration to emis web | out of hours, practice | < 10 |
| 0 | gp | clinic note | out of hours, non practice | < 10 |
| 0 | gp | ooh report | out of hours, practice | < 10 |
| 0 | gp | seen in diabetic clinic | awaiting review | < 10 |
| 0 | gp | sms text message sent to patient | awaiting review | < 10 |
| 0 | nurse | administration note | discussion with colleague | < 10 |
| 0 | other care provider | awaiting clinical code migration to emis web | nursing home | < 10 |
| 0 | other care provider | awaiting clinical code migration to emis web | online services message | < 10 |
| 0 | | awaiting clinical code migration to emis web | bulk operation | < 10 |
| 0 | | non-consultation data | touchscreen | < 10 |
| 0 | dr | awaiting clinical code migration to emis web | hospital outpatient report | < 10 |
| 0 | dr | awaiting clinical code migration to emis web | other | < 10 |
| 0 | dr | awaiting clinical code migration to emis web | third party consultation | < 10 |
| 0 | dr | clinic note | clinic note | < 10 |
| 0 | dr | non-consultation data | non-consultation data | < 10 |
| 0 | gp | awaiting clinical code migration to emis web | hospital inpatient report | < 10 |
| 0 | gp | clinic note | community clinic | < 10 |
| 0 | nurse | administration note | non-consultation data | < 10 |
| 0 | nurse | awaiting clinical code migration to emis web | non-consultation medication data | < 10 |
| 0 | other care provider | externally entered note | data transferred from other system | < 10 |
| 0 | other care provider | gp surgery | | < 10 |
| 0 | other care provider | other note | referral letter | < 10 |
| 0 | | awaiting clinical code migration to emis web | nurseries/playgroup | < 10 |
| 0 | | hospital outpatient report | third party consultation | < 10 |
| 0 | gp | nurse telephone triage | nurse telephone triage | < 10 |
| 0 | dr | awaiting clinical code migration to emis web | discussion with colleague | < 10 |
| 0 | dr | awaiting clinical code migration to emis web | medicine management | < 10 |
| 0 | dr | awaiting clinical code migration to emis web | residential home visit note | < 10 |
| 0 | dr | e-mail received from patient | e-mail received from patient | < 10 |
| 0 | dr | telephone call from relative/carer | telephone call from relative/carer | < 10 |

| | | | | |
|---|---|---|---|---|
| 0 | gp | awaiting clinical code migration to emis web | walk-in centre | < 10 |
| 0 | gp | extended hours consultation | out of hours, non practice | < 10 |
| 0 | gp | externally entered note | | < 10 |
| 0 | gp | other consultation medium used | other note | < 10 |
| 0 | gp | telephone consultation | telephone call to relative/carer | < 10 |
| 0 | nurse | awaiting clinical code migration to emis web | telephone encounter | < 10 |
| 0 | nurse | medication requested | awaiting review | < 10 |
| 0 | nurse | telephone follow-up | awaiting review | < 10 |
| 0 | other care provider | awaiting clinical code migration to emis web | e-mail received from patient | < 10 |
| 0 | other care provider | awaiting clinical code migration to emis web | mail from patient | < 10 |
| 0 | other care provider | awaiting clinical code migration to emis web | out of hours, practice | < 10 |
| 0 | other care provider | other note | data transferred from other system | < 10 |
| 0 | | awaiting clinical code migration to emis web | day case report | < 10 |
| 0 | | awaiting clinical code migration to emis web | encompass message | < 10 |
| 0 | | e-mail encounter to carer | awaiting review | < 10 |
| 0 | | walk-in clinic | walk in centre | < 10 |
| 0 | dr | awaiting clinical code migration to emis web | non-consultation data | < 10 |
| 0 | dr | awaiting clinical code migration to emis web | other note | < 10 |
| 0 | dr | e-mail sent to patient | awaiting review | < 10 |
| 0 | gp | email received from third party | awaiting review | < 10 |
| 0 | gp | hospital inpatient note | awaiting review | < 10 |
| 0 | gp | night visit note | night visit | < 10 |
| 0 | gp | other note | third party consultation | < 10 |
| 0 | gp | telephone encounter | telephone call to relative/carer | < 10 |
| 0 | gp | telephone follow-up | awaiting review | < 10 |
| 0 | nurse | administration note | referral letter | < 10 |
| 0 | nurse | awaiting clinical code migration to emis web | urgent consultation | < 10 |
| 0 | nurse | seen in asthma clinic | awaiting review | < 10 |
| 0 | nurse | seen in hospital ward | awaiting review | < 10 |
| 0 | nurse | telephone consultation | telephone call to relative/carer | < 10 |
| 0 | other care provider | administration note | other note | < 10 |
| 0 | other care provider | administration note | referral letter | < 10 |
| 0 | other care provider | administration note | repeat issue | < 10 |
| 0 | other care provider | administration note | results recording | < 10 |
| 0 | other care provider | awaiting clinical code migration to emis web | casualty attendance | < 10 |
| 0 | other care provider | awaiting clinical code migration to emis web | diabetic clinic | < 10 |

| | | | | |
|---|---|---|---|---|
| 0 | other care provider | awaiting clinical code migration to emis web | encompass message | < 10 |
| 0 | other care provider | clinic note | nhs direct report | < 10 |
| 0 | other care provider | seen in diabetic clinic | awaiting review | < 10 |
| 0 | | awaiting clinical code migration to emis web | other report | < 10 |
| 0 | | child in need meeting | awaiting review | < 10 |
| 0 | | hospital inpatient report | hospital inpatient | < 10 |
| 0 | | progress report | nhs direct report | < 10 |
| 0 | | repeat prescription | non-consultation medication data | < 10 |
| 0 | | weekly care home ward round | awaiting review | < 10 |
| 0 | dr | awaiting clinical code migration to emis web | non-consultation medication data | < 10 |
| 0 | dr | awaiting clinical code migration to emis web | telephone call from a patient | < 10 |
| 0 | dr | awaiting clinical code migration to emis web | telephone call from relative/carer | < 10 |
| 0 | dr | awaiting clinical code migration to emis web | telephone call to relative/carer | < 10 |
| 0 | gp | awaiting clinical code migration to emis web | night visit , practice | < 10 |
| 0 | gp | awaiting clinical code migration to emis web | walk-in clinic | < 10 |
| 0 | gp | non-consultation medication data | non-consultation medication data | < 10 |
| 0 | gp | other consultation medium used | referral letter | < 10 |
| 0 | gp | other note | non-consultation data | < 10 |
| 0 | gp | other note | non-consultation medication data | < 10 |
| 0 | gp | seen in baby clinic | awaiting review | < 10 |
| 0 | gp | telephone encounter | telephone call from relative/carer | < 10 |
| 0 | nurse | administration note | clinic note | < 10 |
| 0 | nurse | administration note | laboratory result | < 10 |
| 0 | nurse | awaiting clinical code migration to emis web | casualty attendance | < 10 |
| 0 | nurse | awaiting clinical code migration to emis web | patientchase insert | < 10 |
| 0 | nurse | clinic note | community clinic | < 10 |
| 0 | nurse | extended hours consultation | out of hours, non practice | < 10 |
| 0 | nurse | externally entered note | data transferred from other system | < 10 |
| 0 | nurse | first attendance face to face | awaiting review | < 10 |
| 0 | nurse | hospital inpatient note | awaiting review | < 10 |
| 0 | nurse | ooh report | out of hours, practice | < 10 |
| 0 | nurse | other consultation medium used | data transferred from other system | < 10 |
| 0 | nurse | other note | data transferred from other system | < 10 |
| 0 | nurse | other note | referral letter | < 10 |
| 0 | nurse | outbound referral | referral letter | < 10 |
| 0 | nurse | patient initiated enc. nos | awaiting review | < 10 |
| 0 | nurse | telephone consultation | telephone call from relative/carer | < 10 |
| 0 | nurse | telephone encounter | telephone call to relative/carer | < 10 |
| 0 | nurse | weekly care home ward round | awaiting review | < 10 |

| | | | | |
|---|---|---|---|---|
| 0 | other care provider | administration note | mail to patient | < 10 |
| 0 | other care provider | awaiting clinical code migration to emis web | hospital inpatient report | < 10 |
| 0 | other care provider | awaiting clinical code migration to emis web | hospital outpatient report | < 10 |
| 0 | other care provider | awaiting clinical code migration to emis web | laboratory result | < 10 |
| 0 | other care provider | clinic note | community clinic | < 10 |
| 0 | other care provider | first attendance face to face | awaiting review | < 10 |
| 0 | other care provider | inbound referral | awaiting review | < 10 |
| 0 | other care provider | non-consultation data | data transferred from other system | < 10 |
| 0 | other care provider | ooh report | out of hours, non practice | < 10 |
| 0 | other care provider | telephone encounter | awaiting review | < 10 |
| 0 | | awaiting clinical code migration to emis web | follow-up/routine visit | < 10 |
| 0 | | awaiting clinical code migration to emis web | letter | < 10 |
| 0 | | awaiting clinical code migration to emis web | minor injuries unit | < 10 |
| 0 | | awaiting clinical code migration to emis web | secretary | < 10 |
| 0 | | email received from third party | awaiting review | < 10 |
| 0 | | gp surgery | non-consultation data | < 10 |
| 0 | | hospital inpatient report | awaiting review | < 10 |
| 0 | | hospital inpatient report | discharge details | < 10 |
| 0 | | multidisciplinary team meeting with patient | awaiting review | < 10 |
| 0 | | night visit note | night visit, local rota | < 10 |
| 0 | | provision of general practitioner intermediate care | gp2gp import | < 10 |
| 0 | | telephone triage encounter | nhs direct report | < 10 |
| 0 | | third party consultation | third party | < 10 |
| 0 | other care provider | nurse telephone triage | nurse telephone triage | < 10 |

## Annex 3: *NHS targets for cancer wait times*

| | | | Operational standard |
|---|---|---|---|
| Maximum two weeks from | Receipt of urgent referral for suspected cancer to first outpatient attendance | | 93% |
| | Receipt of referral of any patient with breast symptoms (where cancer not suspected) to first hospital assessment | | 93% |
| Maximum 28 days from | Receipt of two week wait referral for suspected cancer, receipt of urgent referral from a cancer screening programme (breast, bowel, cervical), and receipt of two week wait referral of any patient with breast symptoms (where cancer not suspected), to the date the patient is informed of a diagnosis or ruling out of cancer | | 75% |
| Maximum one month (31 days) from: | Decision to treat to first definitive treatment | | 96% |
| | Decision to treat/earliest clinically appropriate date to start of second or subsequent treatment(s) for all cancer patients including those diagnosed with a recurrence where the subsequent treatment is: | surgery | 94% |
| | | drug treatment | 98% |
| | | radiotherapy | 94% |
| Maximum two months (62 days) from: | Urgent referral for suspected cancer to first treatment (62-day classic) | | 85% |
| | Urgent referral from a NHS Cancer Screening Programme (breast, cervical or bowel) for suspected cancer to first treatment | | 90% |

## Annex 4: Comparing Cancer Wait Times counts for referrals and first treatments with the NCRAS data equity pack

Table A4.1: Comparing Cancer Wait Times counts for referrals and first treatments with the NCRAS data equity pack

| | 01 Apr 2020 - 31 Jan 2021 | | | | | |
|---|---|---|---|---|---|---|
| | First treatments for new cancer | | | Urgent cancer referrals | | |
| | Observed | Expected | Percentage change (95% CI) | Observed | Expected | Percentage change (95% CI) |
| Cancer wait times data | | | | | | |
| All | 224,323 | 267,946 | -16.3% (-16.6, -15.9) | 1,673,775 | 2,071,967 | -19.2% (-19.3, -19.1) |
| NCRAS data equity pack | | | | | | |
| All | 219,410 | 254,436 | -13.8% (-14.3, -13.3) | 1,658,309 | 1,984,489 | -16.4% (-16.6, -16.3) |
| Breast | 30,488 | 40,530 | -24.8% (-25.9, -23.6) | 337,582 | 363,139 | -7% (-7.5, -6.6) |
| Gynaecological | 11,281 | 12,344 | -8.6% (-10.9, -6.2) | 158,723 | 176,985 | -10.3% (-10.9, -9.7) |
| Head and Neck | 8,892 | 9,901 | -10.2% (-12.7, -7.6) | 163,668 | 189,837 | -13.8% (-14.4, -13.2) |
| Lower GI | 23,507 | 27,056 | -13.1% (-14.6, -11.6) | 302,369 | 366,677 | -17.5% (-17.9, -17.1) |
| Lung | 24,796 | 27,409 | -9.5% (-11.1, -8.0) | 33,830 | 53,641 | -36.9% (-37.8, -36.1) |
| Skin | 40,977 | 43,475 | -5.7% (-7.0, -4.5) | 338,172 | 429,802 | -21.3% (-21.7, -21.0) |
| Upper GI | 17,059 | 17,586 | -3% (-5.0, -0.9) | 141,720 | 163,013 | -13.1% (-13.7, -12.4) |
| Urological | 37,970 | 50,056 | -24.1% (-25.2, -23.1) | 134,389 | 184,642 | -27.2% (-27.7, -26.7) |
| All other | 24,441 | 26,080 | -6.3% (-7.9, -4.6) | 47,856 | 56,753 | -15.7% (-16.7, -14.6) |

# Annex 5: Patient demographics and person-time and total numbers of observed activity in CPRD Aurum sample

Table A5.1: Patient demographics in CPRD Aurum sample as at 22 March 2020

| | Patient count as at 22 March 2020 | |
|---|---|---|
| | n | % |
| All | 375,501 | |
| Female | 187,509 | 49.9% |
| Male | 187,992 | 50.1% |
| Under 11 | 39,611 | 10.5% |
| 11 to 19 | 43,406 | 11.6% |
| 20 to 49 | 157,962 | 42.1% |
| 50 to 69 | 87,482 | 23.3% |
| 70 and older | 47,040 | 12.5% |
| IMD Quintile - 1 | 78,759 | 21.0% |
| IMD Quintile - 2 | 73,046 | 19.5% |
| IMD Quintile - 3 | 71,840 | 19.1% |
| IMD Quintile - 4 | 77,545 | 20.7% |
| IMD Quintile - 5 | 74,020 | 19.7% |
| IMD not recorded | 291 | 0.1% |

Table A5.2: Person time (weeks) and total primary care activity analysed - CPRD Aurum

| | 3 January 2016 - 21March 2020* | | | | 22 March 2020 - 30 January 2021 | | | |
|---|---|---|---|---|---|---|---|---|
| | Patient-time (weeks) | 100,000 person-months | Activity count | Observed rate per 100,000 person-months | Patient-time (weeks) | 100,000 person-months | Activity count | Observed rate per 100,000 person-months |
| Consultations excl flu vaccinations | 78,868,977 | 181.44 | 6,912,079 | 38,095 | 16,701,707 | 38.19 | 1,306,206 | 34,201 |
| Routine Referrals | 78,868,977 | 181.44 | 312,422 | 1,722 | 16,701,707 | 38.19 | 40,744 | 1,067 |
| 2 Week Wait Referrals | 78,868,977 | 181.44 | 38,905 | 214 | 16,701,707 | 38.19 | 10,235 | 268 |

**The pre-pandemic period consists of 220 weeks or 51.61 months, the post pandemic period is 45 weeks or 10.29 months*

## Annex 6: Observed vs expected appointment and cancer diagnosis counts from CWT data from 01 Jan 2019 (per person-month)

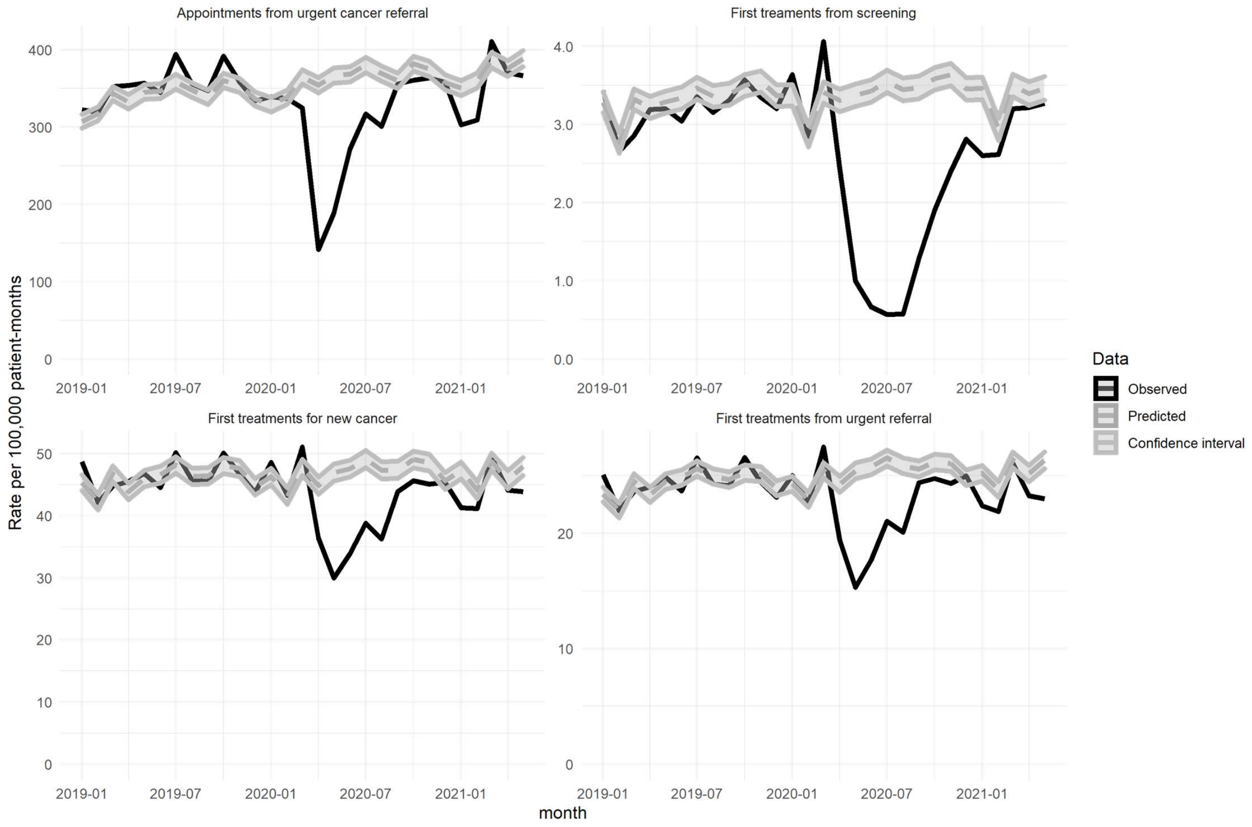

## Annex 7: Observed vs expected consultations by IMD quintile

*Figure A7: Observed vs expected consultations per person per week by IMD quintile, age-standardised (01 Jan 2019 – 30 Jan 2021)*

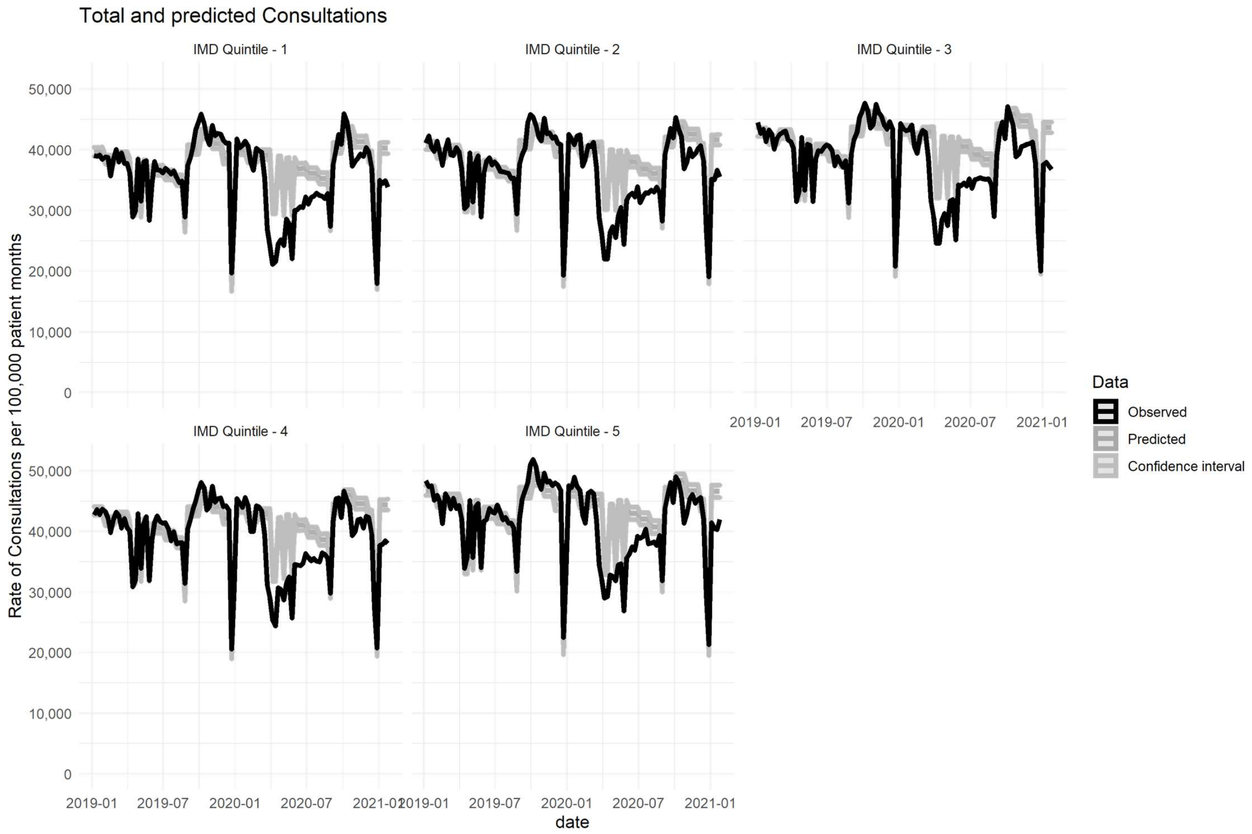

Supplement: Supplementary data [file bmjopen-2021-059374supp001.pdf]
