# Supplementary material for: Primary care and cancer: an analysis of the impact and inequalities of the COVID-19 pandemic on patient pathways
Source: BMJ Open. 2022 Mar 23;12(3):e059374. doi: 10.1136/bmjopen-2021-059374 (PMC8948073; doi:10.1136/bmjopen-2021-059374)
Supplement: Author's manuscript [file bmjopen-2021-059374.draft_revisions.pdf]

BMJ Open

BMJ Open is committed to open peer review. As part of this commitment we make the peer review history of every article we publish publicly available.

When an article is published we post the peer reviewers' comments and the authors' responses online. We also post the versions of the paper that were used during peer review. These are the versions that the peer review comments apply to.

The versions of the paper that follow are the versions that were submitted during the peer review process. They are not the versions of record or the final published versions. They should not be cited or distributed as the published version of this manuscript.

BMJ Open is an open access journal and the full, final, typeset and author-corrected version of record of the manuscript is available on our site with no access controls, subscription charges or pay-per-view fees (http://bmjopen.bmj.com).

If you have any questions on BMJ Open's open peer review process please email info.bmjopen@bmj.com

BMJ Open

Primary Care and Cancer: an analysis of the impact and inequalities of the COVID-19 pandemic on patient pathways

Journal:	*BMJ Open*
Manuscript ID	bmjopen-2021-059374
Article Type:	Original research
Date Submitted by the Author:	18-Nov-2021

Complete List of Authors:	Watt, Toby; The Health Foundation, REAL Centre<br>Sullivan, Richard; King's College London<br>Aggarwal, Ajay; London School of Hygiene and Tropical Medicine Faculty of Public Health and Policy, Health Services Research & Policy

Keywords:	ONCOLOGY, PRIMARY CARE, COVID-19

SCHOLARONE™ Manuscripts

I, the Submitting Author has the right to grant and does grant on behalf of all authors of the Work (as defined in the below author licence), an exclusive licence and/or a non-exclusive licence for contributions from authors who are: i) UK Crown employees; ii) where BMJ has agreed a CC-BY licence shall apply, and/or iii) in accordance with the terms applicable for US Federal Government officers or employees acting as part of their official duties; on a worldwide, perpetual, irrevocable, royalty-free basis to BMJ Publishing Group Ltd ("BMJ") its licensees and where the relevant Journal is co-owned by BMJ to the co-owners of the Journal, to publish the Work in this journal and any other BMJ products and to exploit all rights, as set out in our licence.

The Submitting Author accepts and understands that any supply made under these terms is made by BMJ to the Submitting Author unless you are acting as an employee on behalf of your employer or a postgraduate student of an affiliated institution which is paying any applicable article publishing charge ("APC") for Open Access articles. Where the Submitting Author wishes to make the Work available on an Open Access basis (and intends to pay the relevant APC), the terms of reuse of such Open Access shall be governed by a Creative Commons licence – details of these licences and which Creative Commons licence will apply to this Work are set out in our licence referred to above.

Other than as permitted in any relevant BMJ Author's Self Archiving Policies, I confirm this Work has not been accepted for publication elsewhere, is not being considered for publication elsewhere and does not duplicate material already published. I confirm all authors consent to publication of this Work and authorise the granting of this licence.

For peer review only

Primary Care and Cancer: an analysis of the impact and inequalities of the COVID-19 pandemic on patient pathways

Authors: Toby Watt[1,2], Richard Sullivan[3] and Ajay Aggarwal[4]

1. The Health Foundation, 8 Salisbury Sq., London, UK,
2. Department of Public Health, Environments and Society, London School of Hygiene and Tropical Medicine, 15-17 Tavistock Place, London, UK
3, School of Cancer and Pharmaceutical Sciences, King's College London, London, UK; Institute of Cancer Policy, King's College London, London, UK; Department of
Oncology, Guy's and St Thomas' NHS Foundation Trust, London, UK.
4. Department of Health Services Research and Policy, London School of Hygiene & Tropical Medicine, London, UK; Institute of Cancer Policy, King's College London,
London, UK; Department of Oncology, Guy's and St Thomas' NHS Foundation Trust, London, UK.

Corresponding author: Toby Watt, toby.watt@health.org.uk

Word count: 3,995

For peer review only

Abstract

Objectives

We explore the routes to cancer diagnosis to further undertanding of the inequality in the reduction in detection of new cancers since the start of the pandemic. We use different data sets to assess stages in the cancer pathway: primary care data for primary care consultations, routine and urgent referrals and published analysis of cancer registry data for appointments and first treatments.

Setting

Primary and cancer care

Participants

In this study we combine multiple data sets to perform a population-based cohort study on different areas of the cancer pathway. For primary care analysis, we use a random sample of 500,000 patients from the Clinical Practice Research Datalink (CPRD). Post-referral we perform a secondary data analysis on the Cancer Wait Times data and the NCRAS COVID-19 data equity pack.

Outcome measures:

Primary care: consultation, urgent cancer referral and routine referral rates, then appointments following an urgent cancer referral, and first treatments for new cancer, for all and by quintile of patient's local area Index of Multiple Deprivation.

Results

Primary care contacts and urgent cancer referrals fell by 12.4% (-12.6, -12.3) and 20.2% (-22.3, -18.1) respectively, while routine referrals have not recovered to pre-pandemic levels. Reductions in first treatments for newly diagnosed cancers are down 16.3% (-16.6, -15.9). The reduction in the number of two week wait referrals and first treatments for all cancer has been largest for those living in poorer areas, despite having a smaller reduction in primary care contact.

Conclusions

Our results further evidence the strain on primary care and the presence of the inverse care law, and the dire need to address the inequalities so sharply brought into focus by the pandemic. We need to address the disconnect between the importance we place on the role of primary care and the resources we devote to it.

Key Words: Oncology, Primary Care, COVID-19

Strengths and Limitations

- This study uses multiple data sets to analyse a complex multidisciplinary cancer pathway. Due to data limitations, we innovate in drawing from different data sets to analyse the impact of the pandemic on each stage of the route to diagnosis.
- We use an extremely rich primary care data set containing patient level primary care activity linked to patients' local area socioeconomic indicator.
- Our relatively small (500,000) patient sample is collected from a nationally representative sample of primary care registered patients. The data produces results that closely mirror the rates of consultation and urgent cancer referral per patient (and their reduction) produced in publicly available national data sets.

For peer review only

INTRODUCTION

The COVID-19 pandemic has had a profound impact on UK's health system. Each part of the UK's National Health Service has been impacted in different ways, and we are still feeling many of the consequences of both the COVID-19 pandemic and the public health measures put in place to manage it (non-pharmaceutical interventions; NPI). Cancer is one of the most complicated diseases that the UK health system must manage, being responsible for over one in four UK deaths in 2019. Cancer outcomes are acutely sensitive to changes in social determinants, patient pathways and service provision. Delays in both diagnosis and treatment have significant impacts on patient outcomes(1,2). Pandemic related diagnostic delays, lack of capacity and downstream stage progression (to more advanced disease) are already being seen(3). In addition, the impact of the pandemic needs to be seen in the context of an already overstretched UK cancer care system pre-pandemic that was 'burning hot' even in normal times(4).

Primary care sits at the heart of the cancer patient pathway and is the most crucial interface for early diagnosis and referral to hospital-based care, in addition to their wider support of cancer patient undergoing and post treatment. As models of cancer care have involved in light of both technical advances and an ageing co-morbid population primary care has become an increasingly important aspect of integrated cancer care and an expansion of GP roles in cancer care(5). On average 22.5% of patients diagnosed with cancer come via primary care, but this reflects wide site-specific variation from as little as 8.3% of breast cancer to 42% for bladder cancer(6) (Data table presented in Annex 1).

It is important to reflect that prior to the start of the COVID-19 pandemic primary care had seen significant declines in overall resourcing relative to the funding of the rest of the NHS and compared to growing levels of disease burden that is managed in primary care. In addition, there is growing evidence that primary care has been under greater pressure in more deprived areas, with higher levels of staff turnover(7), higher levels of complex multimorbidity(8), higher numbers of consultations(9) and lower levels of funding and fewer GPs per capita once levels of ill health are taken into account(10). These pressures on primary care, and a desire to correct them, have been recognised in the NHS Long Term Plan(11).

Thus, to understand the COVID-19's impact on primary care and the downstream impact on cancer outcomes we need to see that the pandemic arrived when the system that was already struggling to cope. Prior to COVID-19, the central role of primary care as agents of change in reducing inequalities had been the subject of much debate yet could do little in the face of political avoidance of health equity(12). Primary care had become a mirror on inequalities but also subject to significant pressures from these growing inequalities that had put practices in deprived populations under significant stress. Yet despite this, equity-oriented primary care reform in England in the mid-to-late 2000s may have helped to reduce socioeconomic inequality in health(13).

[Box]

COVID-19 was officially declared a pandemic by the WHO on 11 March 2020, and the UK announced its first full lockdown on 23rd March. In the following months UK NPI were eased, schools reopened in phases, non-essential shops reopened and in August the population were encouraged to eat out. Some restrictions were re-imposed in September and October, on the 5th of November 2020 a second brief national locked lasted until 2nd December. On the 6th of January a third national lockdown was introduced(14).

[End box]

It is now clear that the UK experience of the pandemic was one of the worst in the world, both in terms of excess mortality (both COVID-19 and non-COVID-19) and the impact of NPI (lockdowns) on both the ability of health services to continue provide care and the impact of messaging (stay at home) on patients' timely presentation for care(15). However, the overwhelming focus of impact studies on cancer care has been on hospital-based services, including diagnostics. Given primary care's central role in pathways to diagnosis and integrated cancer care, including survivorship there has been little insight around how overall changes in consultation rates impacted both routine and two week wait referrals as well as how this varied both in terms of site-specific cancers and as a consequence of socio-economic

For peer review only

inequalities. In this study we provide the first detailed analysis of primary care activity in relation to cancer patients during the pandemic with a particular focus on inequalities.

METHODS

Study design, data sources and participants

We perform a population-based cohort study using the following three separate sources.

Primary care data – CPRD Aurum

Primary care electronic health records were obtained from the Clinical Practice Research Datalink (CPRD) database. We included patient records from 01/01/2016 to 31/01/2021. Pre-pandemic data were included to establish long-term trends and patterns of seasonality in primary care use and referrals to secondary care. Similar to recent analysis of the covid pandemic(16), our analysis focusses on comparing observed levels of activity to the expected from March 2020.

The CPRD Aurum database contains anonymised patient data from approximately 7% of the UK population and is broadly representative in terms of age, sex, and ethnicity(17).

The period of eligibility for study inclusion, which starts on the latest of the study start date (01/01/2016) or the patient's registration to their practice. A patient's period of eligibility ends on the earliest of leaving their practice, the end of data collection from their practice or their death. Primary care records from CPRD were linked to the deciled index of multiple deprivation (IMD) from 2015[i](18) of each patient's lower super output area (LSOA). 500,000 patients were randomly sampled from the CPRD population who were eligible for linkage within the defined study period. The patient records include information on consultations, patient demographic information, diagnoses, medication prescriptions, and referrals to secondary care.

Cancer wait times data

Cancer waiting times (CWT) measure performance against the NHS Constitution Standards, as well as a number of other metrics. These measures are used by local and national organisations to monitor the timely delivery of services to patients, they are published quarterly by NHS Digital[ii].

Cancer diagnosis by socioeconomic status - NCRAS Cancer data equity pack

Data on cancer diagnosis by socioeconomic group was drawn from CADEAS and NCRAS which have two equity data packs presenting the latest national data on the number of urgent suspected two-week wait referrals and first definitive treatments for cancer(19). These data packs are produced based on the CWT data, with analysis from Hospital Episode Statistics (HES) and other sources outlined in their technical notes.

Outcomes

CPRD Aurum

We define consultations in CPRD data by a set of rules developed based on two variables in the consultations file[iii] ("EMIS® consultation source identifier" and "Consultation source code identifier"), combined with the type of staff that filled in the record. This method is an adaptation of the approach taken by Carey et al 2012 for CPRD Gold data (Annex 2).

Using the observation file in CPRD Aurum, we were also able to identify where patients had influenza vaccinations. We look to exclude flu vaccines from our analysis on the basis that the programme was expanded in 2020/21 to achieve

---

[i] https://www.gov.uk/government/statistics/english-indices-of-deprivation-2015
[ii] https://www.england.nhs.uk/statistics/statistical-work-areas/cancer-waiting-times/
[iii] https://cprd.com/primary-care

For peer review only - http://bmjopen.bmj.com/site/about/guidelines.xhtml

maximum uptake[iv]. To help with the comparability of consultations in the two periods, we removed primary care appointments that included a flu vaccine.

We were able to identify vaccinations in CPRD Aurum which identifies observations related to vaccination. We then performed a string search for influenza, including notes that clearly referred to a health care professional administering a vaccine. A list of excluded terms is presented in Annex 3 with a summary of consultations including and excluding influenza vaccinations.

Referrals in CPRD are categorized into routine and "urgent cancer". Referrals from the 'referral file' are linked to patients, no additional data cleaning steps were taken in the analysis of referrals.

Cancer Wait Times

The CWT data present monthly counts of patients who have been recorded as having been through each stage of the cancer diagnosis pathway that is held to a performance target (Annex 4).

We include in our analysis: the number of patients that receive a first appointment following an urgent referral, the total number of patients receiving first treatments following a cancer diagnosis and the number of patients receiving first treatments following an urgent cancer referral from primary care.

Cancer diagnosis by socioeconomic status - NCRAS Cancer data equity pack

The data show monthly counts, from January 2018 to January 2021 inclusive, broken down by tumour type and patient factors: deprivation, age, sex and ethnicity. The analysis presents the working day adjusted proportion compared to pre-pandemic levels.

Patient and public involvement

No patient involved

Data analysis

CPRD & CWT

For both our primary care data and the CWT data we perform a linear regression of each outcome over time to estimate expected values for the post-pandemic period (end of March 2020 to end of January 2021), based on predicted values from the data pre pandemic:

- in the case of CPRD Aurum outcomes this is weekly data from 01 January 2016 to 15 March 2020,
- for CWT outcomes this is monthly data from October 2009 to March 2020.

To account for seasonality and time trends we include months as a categorical variable and time as a continuous variable, the approach taken by Carr et al.(16). In the case of weekly primary care data we observe large dips in activity in weeks that include bank holidays and include a categorical variable on the basis of the number of bank holidays in each week (in the winter holidays in England there is always one week with two bank holidays).

When analysing primary care consultation rates by socioeconomics we adjust for population age. We do so when calculating the consultation rates by IMD quintile and weighting the sample according to the European Standard Population[v] (ESP).

NCRAS equity data

[iv] https://www.england.nhs.uk/wp-content/uploads/2020/05/Letter_AnnualFlu_2020-21_20200805.pdf
[v] https://www.causesofdeath.org/docs/standard.pdf

For peer review only - http://bmjopen.bmj.com/site/about/guidelines.xhtml

The analysis presented in the equity data pack compares new instances of first treatments in months during the pandemic (01 April 2020 – 31 Jan 2021) compared with the same months in 2019/20. The analysis includes a 95% confidence interval for the changes, based on rate ratios under an assumption that the population is the same in the pre-COVID-19 baseline and COVID-19 months. This is calculated using the exact method described in Breslow & Day 1987, pp 93-95(20). The NCRAS equity data pack shows the high levels of heterogeneity in the impact of the COVID-19 pandemic on different tumour locations[vi]. Our presentation of these data follows the same method but presents the cumulative difference for the period from April 2020 to the end of January compared with the previous 12 months.

RESULTS

Overall impact of the pandemic

Before the COVID-19 pandemic and the associated NPI, there was an average of 0.086 primary care consultations per person-week (03/03/2020 – 29/02/2020). This equates to 254 million appointments in primary care nationally. Primary care consultations (Figure 1 a) dropped rapidly to a low of 0.062 appointments per person-week in the week following 29/03/2020, this was 66.2% lower than the predicted rate. Rates slowly recovered over the next 22 weeks and by the 31/08/2020 were up to 99% of the baseline. In total there were 18.7 million fewer primary care consultations in the English NHS during this period. Primary care consultations again fell to below 90% of predicted levels in the third wave NPI starting on 06/01/2021, by the end of that month there were a further 8.2 million fewer consultations than expected. Between the start of the first NPI in March 2020 and the end of January 2021 there were 27.0 million fewer appointments than expected.

In the year prior to the pandemic, the average rate of urgent cancer (two week wait) referral was 0.00073 per person per week, equating to an estimated 2.1 million for the NHS in England (March 2019 to March 2020). Following the first NPI, urgent cancer referrals from primary care (Figure 1 b) fell to a nadir of 0.00023 by 29/03/2020 (29.7% of the predicted level). Urgent cancer referrals did not return to pre-pandemic baseline until the week following 23/08/2020 equating to 303,016 estimated lost urgent cancer referrals over this period. There was a second fall in urgent cancer referrals from primary care in the winter to below 90% of the baseline following the third lockdown (around 0.00065 referrals per person-week). This resulted in a further estimated 91,705 fewer urgent cancer referrals than expected. Between the start of the first NPI in March 2020 and the end of January 2021 there were 394,721 fewer urgent cancer referrals than expected.

Routine referrals however have shown a different trajectory in that their rates did not recover to pre-pandemic levels (Figure 1 c). As a share of predicted levels routine referrals had the greatest fall, dropping to around 16% of predicted rates for three weeks from the 05/04/2020. From then to the end of January the closest it came to predicted levels was 80.3% in the week flowing 13/09/2020. In January 2021 it had fallen back down to 60-70% of predicted rates. In the year before March 2020 there were an average of 0.0042 routine referrals per person-week from primary care, equivalent to an estimated 12.2 million for the NHS in England. Between the start of the first NPI in March 2020 and the end of January 2021 there were 4.3 million fewer routine referrals than expected.

[vi] The NCRAS data equity pack is different in its count and analysis of "all tumours" compared with the Cancer Wait Times Data, this is because the data are slightly different (cleaned and analysed by NCRAS). Results of our analysis with each data set are compared in Annex 7.

Table 1a: Observed post COVID-19 primary care activity (CPRD Aurum) 15 March 2020 – 30 January 2021

	Observed rate	Expected rate	Percentage reduction (95% CI)	Estimated # missing from England population, n
Event rate per 100,000 person months				
Consultations excl flu vaccinations	31,996	36,517	12.4% (12.3, 12.6)	26,887,014
Routine Referrals	1,046	1,772	41.1% (40.4, 41.8)	4,317,710
2 Week Wait Referrals	268.0	335.8	20.2% (18.1, 22.3)	394,721

Table 1b: Observed post COVID-19 cancer diagnostic activity (Cancer Wait Times), 01 April 2020 - 31 January 2021

	Observed rate	Expected rate	Percentage reduction (95% CI)	Estimated # missing from England population, n
Event rate per 100,000 person months				
First consultant appointments following urgent referral from primary care	296.0	366.4	19.2% (19.1, 19.3)	398,192
Incidence rate per 100,000 person months				
First treatments for new cancer from the urgent primary care referral pathway	21.4	25.5	16.1% (15.5, 16.8)	23,270
First treatments for new cancer from the national screening pathway	1.6	3.5	53.2% (52.0, 54.3)	10,444
First treatments for new cancer	39.7	47.4	16.3% (15.9, 16.6)	43,623

Table 1a summarises the missing appointments and referrals for the post-pandemic period. Since the start of the pandemic in March we have observed consultations rates (excluding flu vaccine appointments) that are 12.4% (C.I. 12.3%, 12.6%) lower than predicted by previous data: equivalent to 27 million appointments when adjusted to match the whole England population between 02/03/2020 and 30/01/2021. The number of referrals to secondary care per consultation has also fallen, with urgent cancer referrals 20.2% (C.I. 18.1%, 22.3%) and routine referrals 41.1% (C.I. 40.4%,41.8%) lower than expected: this is an estimated 394,721 and 4.3 million missing urgent cancer and routine referrals respectively.

The knock-on effect of the reductions in patients' primary care appointments and referrals can be observed in the national CWT data. The number of first appointments with a cancer specialist following an urgent cancer referral has fallen by approximately the same amount as estimated for the referrals themselves: 19.2% (C.I. 19.1%, 19.3%). The number of cancer first treatments (following a diagnosis and decision to treat) was 16.3% (C.I. 15.9%, 16.6%) lower than expected, or 43,623 missing first treatments from 01/04/2020 - 31/01/2021[vii] (graphs of observed compared with expected are presented in Annex 6).

Urgent cancer referrals by site specific cancer from 01/04/2020 until 31/01/2021 showed significant heterogeneity from moderate reductions in urgent referrals for suspected breast (7.0% C.I. 6.6%, 7.5%) and gynaecological cancers (10.3% C.I. 9.7%, 10.9%), to 36.9% (C.I. 36.1%, 37.8%) and 27.2% (C.I. 26.7%, 27.7%) reduction in urgent referrals for lung and urological cancers, respectively (Figure 2). To show how pathway delays interface with reductions in cancer referrals we also examined reductions in first treatments for the same site-specific cancers over this period (Figure 2). Breast and urological cancers observed the greatest reduction in new first treatments: Breast fell by 24.8% (C.I. 23.6%, 25.9%) which equates to 10,042 missing treatments and urological by 24.1% (C.I. 23.2%, 25.2%) which equates to 12,086 missing treatments. Taken together these data reflect substantial delays in both diagnostic and treatment phases of the patient pathway.

[vii] Dates for the CWT and NCRAS analysis do not line up with the CPRD analysis because the latter is conducted weekly, not monthly.

For peer review only

Inequalities in cancer diagnosis outcomes in the pandemic

There are inequalities in primary care use in England, with the people who live in the poorest areas have higher rates of consultation than those in richer areas once we adjust for age. The most deprived quintile was expected to have 41,702 consultations per 100,000 person-months, 15% more than the least deprived.

Table 2: Observed post COVID-19 primary care activity (CPRD Aurum) by IMD quintile, actual and age-standardised

	22 Mar 2020 - 30 Jan 2021 (Weekly)		
	Observed rate	Expected rate	Percentage reduction (95% CI)
Consultations per 100,000 person-months			
IMD Quintile - 1 (least deprived)	32,015	37,030	13.5% (13.2, 13.9)
IMD Quintile - 2	32,490	37,308	12.9% (12.6, 13.3)
IMD Quintile - 3	33,479	38,707	13.5% (13.2, 13.9)
IMD Quintile - 4	32,143	36,573	12.1% (11.8, 12.5)
IMD Quintile - 5 (most deprived)	33,361	37,062	10.0% (9.6, 10.3)
Consultations per 100,000 person-months (Age-standardised*)			
IMD Quintile - 1 (least deprived)	31,216	36,141	13.6% (13.3, 14.0)
IMD Quintile - 2	32,244	37,160	13.2% (12.9, 13.6)
IMD Quintile - 3	33,886	39,382	14% (13.6, 14.3)
IMD Quintile - 4	34,683	39,677	12.6% (12.3, 12.9)
IMD Quintile - 5 (most deprived)	37,487	41,702	10.1% (9.8, 10.4)

**Age-standardisation is performed according to the European Standard Population (ESP)*

For peer review only

The reduction of consultations over the period 22/03/2020-30/01/2021 was smallest for those in most deprived areas. Their reduction in consultations for the non-age-standardised figures was 10.0% (C.I. 9.6%, 10.3%), while for the least deprived the reduction was 13.5% (C.I. 13.2%, 13.9%). Weekly levels of age-standardised consultations per person-week by IMD quintile are presented in Annex 8.

Despite a smaller reduction in primary care contacts, we observe the largest reduction in both urgent cancer referrals and first treatments for cancer for patients living in the most deprived areas. The NCRAS data equity pack presents the number of urgent cancer referrals and first cancer treatments by IMD quintile[viii]. Figure 3 shows the reduction in urgent cancer referrals and first treatments for newly diagnosed cancer by IMD quintile.

There was a greater percentage reduction in urgent cancer referrals for those living in the most deprived areas in England experienced a 17.6% (C.I. 17.2%, 18.0%) reduction between 01/03/2020 and 31/01/2021 compared with the same period 12 months before, while referrals for the least deprived quintile fell by proportionately less: 15.3% (C.I. 14.9%, 15.6%). This equates to a reduction of 61,469 referrals for the most deprived and 62,601 or the least: without adjusting for age, the most deprive quintile had a smaller proportion of the pre-pandemic urgent cancer referrals, with 349,640 referrals compared to 410,293 for the least deprived quintile from April 2019 to January 2020.

At the same time, rates of new treatment for cancer for the people living in the most deprived 20% of England experienced a 15.8% (C.I. 14.6%, 17.0%) reduction between 01/03/2020 and 31/01/2021 compared with the same

---

[viii] They do not age-standardise their results

period 12 months before (6,607 missing first treatments). The reduction for the least deprived was 12.6% (C.I. 11.5%, 13.7%) which equates to 6,880 missing first treatments.

Despite having more access to primary care for patients in more deprived areas (10.1% reduction for most deprived compared to 13.6% for the least deprived), urgent cancer referrals and newly diagnosed cancers have been disrupted by the pandemic more for people living in poorer areas.

DISCUSSION

The coronavirus SARS-CoV-2 (COVID-19) pandemic has had a profound impact on the management of patients with cancer(21). The first national lockdown in March 2020 created a ripple of non-pharmaceutical interventions, including 'stay at home' orders, diminished healthcare service provision and redistribution of healthcare to COVID-19 related care that has had a profound impact on cancer services(1,22). Whilst it was already known that there had been a substantial reduction in the number of overall cancer-related referrals(23,24) the quantification of this had been missing. Our findings, that primary care consultations in English NHS fell by 12.4% between January 2020 and January 2021 with urgent cancer referrals even more suppressed (20.2%), reflect how profound the pathway disruptions were for cancer patients. Furthermore, many cancers are picked up through the course of routine referrals from general practice for non-specific symptoms. The drop in routine referrals that we found - 4.3 million – over this period will inevitably translate into late-stage presentation and a substantial reduction in outcomes. This will include wider economic costs due to more expensive (late stage) treatment and productivity losses due to morbidity and premature mortality. However, the trajectory of the declines reflect not just changes to national policy in terms of NPI, but also knock-on effects around public behaviour, primary care staffing, downstream reductions in diagnostics and an overall increase in friction across all cancer pathways and systems.

This reduction in cancer pathways through primary care needs to be put in the context of wider disruptions. The suspension of national cancer screening programmes meant that circa 2 million people were not screened for cancer through national programs(26,27). Moreover, delays in cancer diagnoses and treatments have consistently been associated with poorer outcomes(1,2). The COVID-19 pandemic has also exacerbated the worst 62-day cancer waiting time targets in the last decade where 1 of 4 patients urgently referred from primary care between April 2020 and January 2021 did not receive treatment within 62 days(24).

In our analysis of urgent cancer referrals by site in relation to reductions seen in first treatments significant differences were seen. Urological cancers (testis, renal and urothelial) have been particularly impacted with greater than 25% decrease both in urgent referrals and first treatments. This suggests that outcomes will be particularly impacted in this group. Lung, skin and lower GI (colon and rectal) also experienced significant declines in urgent referrals. Breast cancer was the least impacted of all in terms of urgent referrals, but experienced a 25% reduction in first treatments, indicating that cancer's reliance on screening programmes, which have suffered yet more than urgent referrals as a result of the pandemic(24). Although it is likely that some cancer patients have already been 'lost' to the system i.e. died of COVID-19 or other non-COVID19 comorbidities, a significant number will now present with later stage disease.

Our findings also reflect socioeconomic inequalities with more profound decrease in urgent cancer referrals and first treatments for the most deprived populations, despite relatively better preservation of consultation rates. This is unexpected and extremely worrying, indicating greater disruption to the diagnostic pathway for patients living in more deprived areas, whose cancer outcomes were typically worse than their less deprived counterparts pre-pandemic(28,29).

Limitations

This study uses multiple data sets to analyse a complex and disjointed pathway. We include a primary care data set that uses a relatively small (500,000) patient sample. However, the CPRD data produces results that closely mirror the rates of consultation per patient (and their reduction) produced in NHS Digital's appointments data(25). In addition, the estimated reduction in urgent cancer referrals is close to those presented in the NCRAS's analysis of their cancer registry

For peer review only

data (Table 1). It is not yet possible to link these data on a patient basis due to delays in data access and once possible further research would be illuminating.

For peer review only

Conclusions

Our data reflects a disruption to a complex interaction of several systemic issues that place a great deal of impetus on the role of primary care in ensuring early diagnosis of cancer. Primary care were under strain pre-pandemic, with low levels of investment creating problems(30). Particularly areas of high deprivation general practice is under-funded and under-doctored relative to need(7,8,10).

Early cancer diagnosis requires concordance of each participant and mechanism - including patients' awareness and ability to present with cancer symptoms, the ability of GPs to detect and urgently refer possible cancer cases and sufficient diagnostic capacity (in terms of both workforce and equipment) to enable swift referrals and minimise delays to diagnosis and treatment.

Every one of these nodes on the pathway to early diagnosis has been affected by the pandemic and the government response. There are also new potential barriers to the pathway that have resulted, including decreases in health seeking behaviour, many appointments being conducted remotely, changes in routine referral guidelines, changes in the capacity of acute care. However, further work is required as there is currently little understanding and even less evidence about how much each disruption is ultimately impacting cancer pathways.

The impact of the pandemic on cancer shown here is concerning. However, what is more concerning is the unequal and inequitable impact on those worst off. Cancer as a disease area *"magnifies what we know to be true about the totality of the health care system. It exposes all its strengths and weaknesses"*(31). Our results further evidence the strain on primary care and the presence of the inverse care law(32), and the dire need to address the inequalities so sharply brought into focus by the pandemic. We need to address the disconnect between the importance we place on the role of primary care and the resources we devote to it.

Figure legends:

Figure 1: Observed vs expected primary care activity, 01 Jan 2019 – 30 January 2021 (per person-week) (CPRD Aurum data) Panels: a. Consultations (excluding flu vaccinations), b. Urgent cancer (2 week wait) referrals from primary care, c. Routine referrals from primary care

Figure 2: Change in observed vs expected first treatments for new cancer and urgent cancer referrals by tumour location from NCRAS Cancer equity data pack (%, 01 Apr 2020 to 31 Jan 2021)

Figure 3: Percentage difference in observed and expected urgent cancer referrals and first treatments for cancer by IMD quintile (01 Apr 2020 – 31 Jan 2021)

Contributors: Data cleaning and analysis was conducted by TW. TW conceived the study with input from the coauthors and wrote the first draft. All the authors provided critical scholarly feedback on the manuscript. All the co-authors approved of the final version of the manuscript. TW and RS are the guarantors. The corresponding author attests that all listed authors meet authorship criteria and that no others meeting the criteria have been omitted.

Competing interests: None

Funding: This publication is funded through the UK Research and Innovation GCRF grant ES/P010962/1.

Data Availability statement:

The primary care activity data may be obtained from a third party and are not publicly available. We used de-identified primary care data from the Clinical Practice Research Datalink (CPRD). For more information please visit: https://www.cprd.com/Data-access, enquiries can be emailed to enquiries@cprd.gov.uk. Scientific approval for this study was given by the CPRD Independent Scientific Advisory Committee (ISAC). The study was approved by the Independent Scientific Advisory Committee for CPRD research (20_143).

For peer review only

Other data sources are available in a public, open access repository: Cancer Wait Times at https://www.england.nhs.uk/statistics/statistical-work-areas/cancer-waiting-times/ and the NCRAS Cancer data equity pack is available at http://www.ncin.org.uk/local_cancer_intelligence/cadeas.

References

1. Maringe C, Spicer J, Morris M, Purushotham A, Nolte E, Sullivan R, et al. The impact of the COVID-19 pandemic on cancer deaths due to delays in diagnosis in England, UK: a national, population-based, modelling study. Lancet Oncol. 2020;21(8).
2. Hanna TP, King WD, Thibodeau S, Jalink M, Paulin GA, Harvey-Jones E, et al. Mortality due to cancer treatment delay: systematic review and meta-analysis. BMJ. 2020;371.
3. Purushotham A, Roberts G, Haire K, Dodkins J, Harvey-Jones E, Han L, et al. The impact of national non-pharmaceutical interventions ('lockdowns') on the presentation of cancer patients. Ecancermedicalscience. 2021;15.
4. Health at a Glance: Europe 2020 [Internet]. OECD; 2020 [cited 2021 Oct 14]. (Health at a Glance: Europe). Available from: https://www.oecd-ilibrary.org/social-issues-migration-health/health-at-a-glance-europe-2020_82129230-en
5. Meiklejohn JA, Mimery A, Martin JH, Bailie R, Garvey G, Walpole ET, et al. The role of the GP in follow-up cancer care: a systematic literature review. Vol. 10, Journal of Cancer Survivorship. 2016.
6. PHE. Routes to Diagnosis [Internet]. National Cancer Registration and Analysis Service. [cited 2021 Oct 14]. Available from: http://www.ncin.org.uk/publications/routes_to_diagnosis
7. Parisi R, Lau YS, Bower P, Checkland K, Rubery J, Sutton M, et al. Rates of turnover among general practitioners: A retrospective study of all English general practices between 2007 and 2019. Vol. 11, BMJ Open. 2021.
8. Head A, Fleming K, Kypridemos C, Schofield P, Pearson-Stuttard J, O'Flaherty M. Inequalities in incident and prevalent multimorbidity in England, 2004–19: a population-based, descriptive study. Lancet Heal Longev. 2021;2(8).
9. DHSC, ONS. Direct and Indirect health impacts of COVID-19 in England [Internet]. London; 2021 Sep [cited 2021 Oct 14]. Available from: https://assets.publishing.service.gov.uk/government/uploads/system/uploads/attachment_data/file/1018698/S1373_Direct_and_Indirect_Health_Impacts_of_C19_Detailed_Paper_.pdf
10. Fisher R, Dunn P, Asaria M, Thorlby R. Level or not? Comparing general practice in areas of high and low socioeconomic deprivation in England. London; 2020 Sep.
11. NHS. NHS Long Term Plan Summary. NHS Improv. 2019;
12. Moscrop A. Health inequalities in primary care: Time to face justice. Vol. 62, British Journal of General Practice. 2012.
13. Cookson R, Mondor L, Asaria M, Kringos DS, Klazinga NS, Wodchis WP. Primary care and health inequality: Difference-in-difference study comparing England and Ontario. PLoS One. 2017;12(11).
14. IFG. Timeline of UK coronavirus lockdowns, March 2020 to March 2021 [Internet]. Institute for Government . 2021 [cited 2021 Oct 14]. Available from: https://www.instituteforgovernment.org.uk/sites/default/files/timeline-lockdown-web.pdf
15. Health and Social Care Committee & Science and Technology Committee. Coronavirus: lessons learned to date [Internet]. London; 2021 Oct [cited 2021 Oct 14]. Available from: https://committees.parliament.uk/work/657/coronavirus-lessons-learnt/news/157991/coronavirus-lessons-

For peer review only

learned-to-date-report-published/

16. Carr MJ, Steeg S, Webb RT, Kapur N, Chew-Graham CA, Abel KM, et al. Effects of the COVID-19 pandemic on primary care-recorded mental illness and self-harm episodes in the UK: a population-based cohort study. Lancet Public Heal. 2021;6(2).

17. Herrett E, Gallagher AM, Bhaskaran K, Forbes H, Mathur R, Staa T van, et al. Data Resource Profile: Clinical Practice Research Datalink (CPRD). Int J Epidemiol. 2015;44(3).

18. Padmanabhan S, Carty L, Cameron E, Ghosh RE, Williams R, Strongman H. Approach to record linkage of primary care data from Clinical Practice Research Datalink to other health-related patient data: overview and implications. Eur J Epidemiol. 2019;34(1).

19. NHS, PHE. The Cancer Alliance Data, Evaluation and Analysis Service [Internet]. National Cancer Registration and Analysis Service: Local Cancer Intelligence. 2021 [cited 2021 Oct 14]. Available from: http://www.ncin.org.uk/local_cancer_intelligence/cadeas#covid-19

20. Breslow NE, Day NE. Statistical methods in cancer research. Volume II--The design and analysis of cohort studies. IARC scientific publications. 1987.

21. Raymond E, Thieblemont C, Alran S, Faivre S. Impact of the COVID-19 Outbreak on the Management of Patients with Cancer. Vol. 15, Targeted Oncology. 2020.

22. UK Government. Staying at home and away from others (social distancing) [Internet]. Cabinet Office. 2020 [cited 2021 Oct 29]. Available from: https://www.gov.uk/government/publications/full-guidance-on-staying-at-home-and-away-from-others

23. Morris EJA, Goldacre R, Spata E, Mafham M, Finan PJ, Shelton J, et al. Impact of the COVID-19 pandemic on the detection and management of colorectal cancer in England: a population-based study. Lancet Gastroenterol Hepatol. 2021;6(3).

24. Cancer Waiting Times Team: NHS England and NHS Improvement. Cancer Wait Times Statistics [Internet]. NHS England. 2021 [cited 2021 Oct 25]. Available from: https://www.england.nhs.uk/statistics/statistical-work-areas/cancer-waiting-times/

25. NHS. Appointments in General Practice - September 2021 [Internet]. NHS Digital. 2021 [cited 2021 Nov 16]. Available from: https://digital.nhs.uk/data-and-information/publications/statistical/appointments-in-general-practice

26. Sud A, Torr B, Jones ME, Broggio J, Scott S, Loveday C, et al. Effect of delays in the 2-week-wait cancer referral pathway during the COVID-19 pandemic on cancer survival in the UK: a modelling study. Lancet Oncol. 2020;21(8).

27. Greenwood E, Swanton C. Consequences of COVID-19 for cancer care — a CRUK perspective. Vol. 18, Nature Reviews Clinical Oncology. 2021.

28. Coleman MP, Rachet B, Woods LM, Mitry E, Riga M, Cooper N, et al. Trends and socioeconomic inequalities in cancer survival in England and Wales up to 2001. Br J Cancer. 2004;90(7).

29. Cancer Research UK. Cancer incidence for all cancers combined: cancer incidence by deprivation [Internet]. 2020 [cited 2021 Oct 14]. Available from: https://www.cancerresearchuk.org/health-professional/cancer-statistics/incidence/all-cancers-combined#heading-Three

30. Roland M, Everington S. Tackling the crisis in general practice. BMJ. 2016;

31. Levit LA, Balogh EP, Nass SJ, Ganz PA. Delivering high-quality cancer care: Charting a new course for a system in crisis. Delivering High-Quality Cancer Care: Charting a New Course for a System in Crisis. 2014.

32. Tudor Hart J. THE INVERSE CARE LAW. Lancet. 1971;297(7696).

For peer review only

Key messages

- Primary care is key part of the pathway for early cancer diagnosis through both routine and 2ww referrals
- Cancer diagnosis rates have experienced a sustained fall since the start of the COVID-19 pandemic and introduction of non-pharmaceutical interventions (NPIs) 'lockdowns'.
- The fall in urgent cancer referral is larger than the fall in primary care contacts, implying that the content of consultations has shifted away from potential cancer diagnosis.
- Despite having a smaller reduction in primary care contact through the pandemic, patients living in poorer areas have had larger reductions in urgent cancer referrals and first treatments for new cancer.
- Government, patients and primary care staff must work together to catch up on missing diagnosis.
- Resilience in primary care is key for the cancer diagnosis pathway and must be developed for future disruptions, particularly in poorer areas where care is more complex.

For peer review only

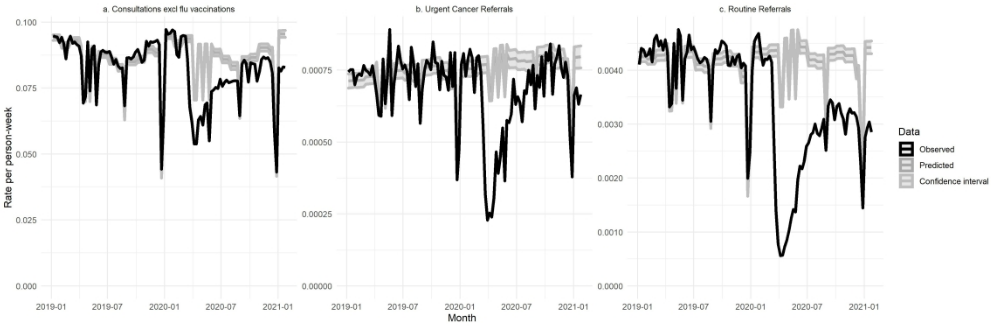

Figure 1: Observed vs expected primary care activity, 01 Jan 2019 – 30 January 2021 (per person-week) (CPRD Aurum data) Panels: a. Consultations (excluding flu vaccinations), b. Urgent cancer (2 week wait) referrals from primary care, c. Routine referrals from primary care

381x127mm (300 x 300 DPI)

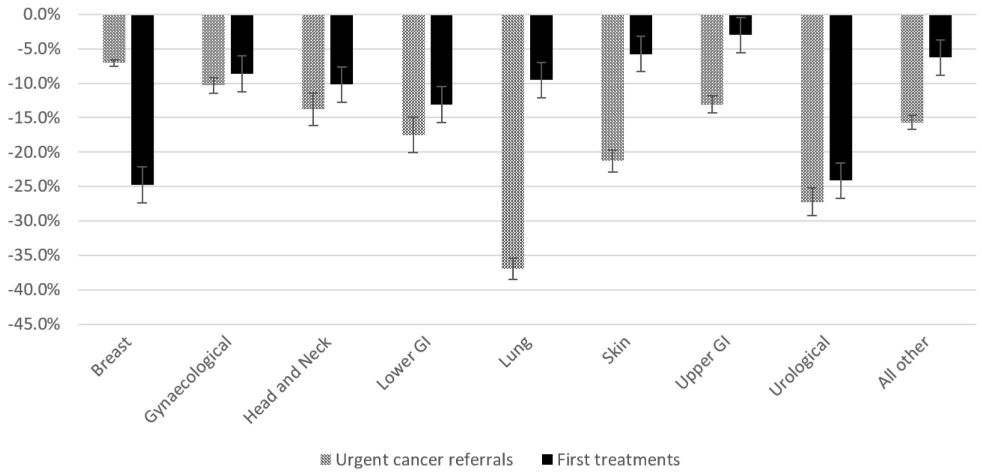

Figure 2: Change in observed vs expected first treatments for new cancer and urgent cancer referrals by tumour location from NCRAS Cancer equity data pack (%, 01 Apr 2020 to 31 Jan 2021)

720x351mm (47 x 47 DPI)

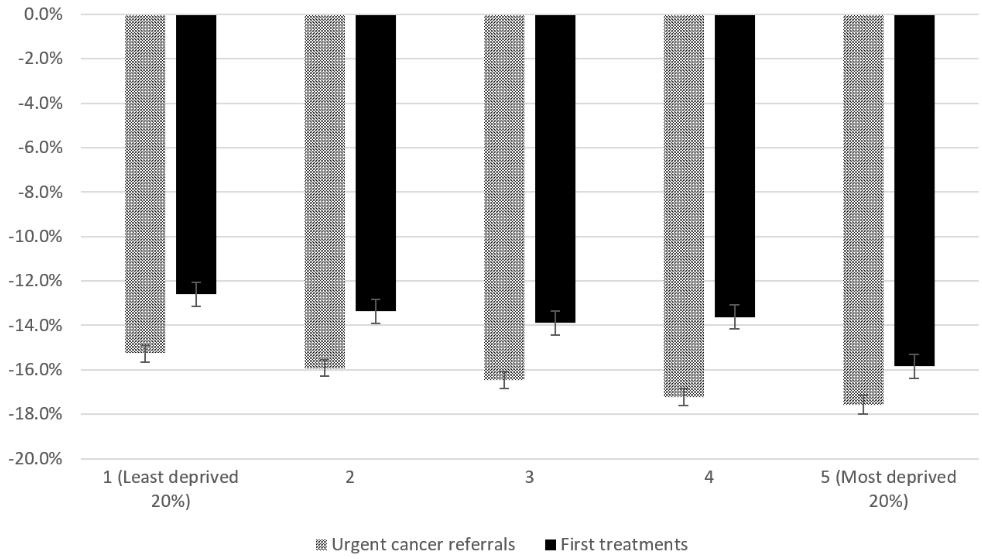

Figure 3: Percentage difference in observed and expected urgent cancer referrals and first treatments for cancer by IMD quintile (01 Apr 2020 – 31 Jan 2021)

698x403mm (47 x 47 DPI)

Annex 1: NCRAS Analysis of routes to diagnosis

Table 1 NCRAS analysis of route to diagnosis in 2017

	Count	Screen	TWW	GP referral	Other outpatient	Inpatient elective	Emergency	DCO	Unknown
All (excl. NMSC)	305,682	6.1%	38.6%	22.5%	9.0%	1.8%	18.8%	0.1%	3.1%
Testis	1,854		61.8%	13.6%	9.2%	2.3%	9.9%	0.0%	3.2%
Uterus	7,862		58.5%	24.8%	5.6%	0.7%	7.4%	0.1%	2.9%
Prostate	41,201		53.2%	28.2%	7.0%	1.3%	6.8%	0.1%	3.4%
Breast cancer	53,123	34.1%	48.0%	8.3%	2.7%	0.2%	3.4%	0.1%	3.2%
Oesophagus	7,569		46.3%	18.1%	6.8%	8.5%	18.1%	0.1%	2.1%
Head and neck	12,865		46.1%	28.1%	14.7%	1.4%	6.8%	0.1%	2.8%
Colorectal	34,825	9.9%	35.8%	20.3%	7.0%	3.1%	21.5%	0.1%	2.3%
Stomach	5,142		32.5%	21.6%	7.1%	6.3%	30.0%	0.1%	2.4%
Bladder	17,056		30.2%	42.0%	12.4%	1.6%	12.0%	0.1%	1.6%
Kidney	9,298		30.1%	28.9%	16.4%	1.7%	19.7%	0.1%	3.1%
Lung	38,906		26.9%	23.2%	14.1%	1.6%	31.5%	0.1%	2.7%
Pancreas	8,829		21.7%	21.2%	9.8%	2.5%	41.9%	0.3%	2.7%
Liver	4,975		17.2%	25.6%	12.7%	1.8%	37.6%	0.3%	4.7%
Haematological	7,377		14.5%	32.2%	8.4%	2.6%	35.1%	0.1%	7.2%
Cervix	23,569	39.6%	2.7%	33.6%	8.0%	3.0%	1.9%	0.0%	11.3%
Brain	5,116		1.5%	22.1%	24.4%	2.6%	45.7%	0.1%	3.7%

Annex 2: Consultation definition

CPRD Aurum data dictionary sets out the structure of the data. Within the consultation file there are two variables one can use to identify whether a primary care contact, rather than an administrative note ("EMIS® consultation source identifier" and "Consultation source code identifier").

The EMIS consultation source identifier is the primary variable used. We include the following observations of this variable:

Acute visit, Casualty attendance, Clinic, Emergency appointment, Emergency consultation, Enterprise consultation, Face to face consultation, Follow-up/routine visit, Gp surgery, Home visit, Home visit note, Main surgery, Nursing home, Nursing home visit note, Online services message, Other, Residential home, Residential home visit note, Same day appointment, Surgery consultation, Telephone encounter, Urgent consultation, Walk-in centre, Walk-in clinic

We also include instances where EMIS consultation source identifier is "awaiting review" and the Consultation source code identifier is in the following list:

Consultation, visit, seen in gp unit, seen in private clinic, seen in rapid access clinic at gp surgery, seen in urgent care centre, online communication.

We then further exclude records on the basis of the category of staff responsible for the record. The “Job category” variable from the staff file, linked by the consultation id is used. We only include as a consultation records filled out by GPs, doctors, nurses and other health care professionals as defined in CPRD’s numerical codes listed below:

GP – 4, 5, 15, 24, 31, 181, 183

Dr – 1, 41, 91, 116, 119, 121, 126, 173, 177, 197

Nurse – 8, 9, 27, 33, 47, 48, 50, 55, 59, 60, 61, 111

Other healthcare professional - 2, 3, 6, 7, 10:14, 16, 17, 34:37, 42, 43, 52, 54, 58, 62:65, 68, 72, 73, 77, 80, 82, 83, 86:89, 94, 95, 97, 100:102, 105, 106, 112:114, 118, 122, 125, 127, 131, 135, 136, 138, 141, 142, 145, 148, 149, 154, 156, 158, 168, 185, 186, 188, 189, 204, 208

In Table A2.1 we present the CPRD Aurum Staff Category list.

In Table A2.2 we present the total number of consultations identified from 01 January 2016 to 31 January 2021, the count with each combination of staff category, “EMIS® consultation source identifier” and “Consultation source code identifier” in table y we show the count of records that were excluded, highlighting those that were excluded on the basis of staff category, not the consultation file description variables.

Table A2.1: CPRD Aurum Staff Job Categories

1 Consultant
2 Hospital Practitioner
3 Clinical Medical Officer
4 General Medical Practitioner
5 Salaried General Practitioner
6 Midwife - Sister/Charge Nurse
7 Midwife
8 Community Practitioner
9 Community Nurse
10 Chiropodist/Podiatrist
11 Dietitian
12 Pharmacist
13 Clinical Psychologist
14 Health Care Support Worker
15 Associate Practitioner - General Practitioner
16 Counsellor
17 Phlebotomist
18 Clerical Worker
19 Manager
20 Analyst
21 System Administrator
22 Desktop Support Administrator
23 System Worker
24 GP Registrar
25 Medical Student
26 Other Community Health Service - Admin Clerk
27 Specialist Nurse Practitioner
28 Receptionist
29 Secretary
30 Medical Secretary
31 Sessional GP
32 Clinical Application Administrator
33 Nurse Consultant
34 Physiotherapist
51 Helper/Assistant
52 Community Mental Health Nurse
53 Senior Administrator
54 Technician - Healthcare Scientists
55 Associate Practitioner - Nurse
56 Senior Manager
57 Community Administrator
58 Associate Specialist
59 Student Practice Nurse
60 Nurse Manager
61 Sister/Charge Nurse
62 Psychotherapist
63 Osteopath
64 Social Care Support Worker
65 Assistant Psychologist
66 Officer
67 Technician - Admin & Clerical
68 Psychiatrist
69 Health Records Clerk
70 Desktop Support Technician
71 Dispenser
72 Clinical Assistant
73 Practitioner
74 Information Officer
75 Network Administrator
76 Chaplain
77 Student Physiotherapist
78 Paramedic Specialist Practitioner
79 Clinical Team Manager
80 Physiotherapist Specialist Practitioner
81 Helpdesk Technician
82 Radiographer
83 Other Community Health Service
84 Call Operator

For peer review only

35 Specialist Practitioner
36 Healthcare Assistant
37 Medical Technical Officer - Pharmacy
38 Health Records Administrator
39 Helpdesk Administrator
40 Appointments Clerk
41 Senior House Officer
42 Social Worker
43 Trainee Practitioner
44 Network Technician
45 Clinical Coder
46 Medical Records Clerk
47 Staff Nurse
48 Enrolled Nurse
49 Multi Therapist
50 Nursery Nurse
85 Community Worker (children)
86 Paramedic Consultant
87 Associate Practitioner
88 Modern Matron
89 Asst. Clinical Medical Officer
90 Community Team Manager
91 Specialist Registrar
92 Chiropodist/Podiatrist Manager
93 Radiographer - Therapeutic, Manager
94 Optometrist
95 Assistant Practitioner
96 Community Learning Disabilities Nurse
97 Technician - Additional Clinical Services
98 Student Health Visitor
99 Interpreter
100 Medical Technical Officer

For peer review only

101 Midwife - Specialist Practitioner
102 Occupational Therapist
103 Chief Executive
104 Audit Manager
105 Paramedic
106 Physiotherapist Consultant
107 Availability Monitor
108 Medical Laboratory Assistant
109 Gateway Worker
110 Medical Records Manager
111 Student Nurse - Adult Branch
112 Audiologist
113 Radiographer - Diagnostic
114 Therapist
115 Student District Nurse
116 House Officer - Post Registration
117 Speech & Language Therapist
118 Dietitian Specialist Practitioner
119 Trust Grade Doctor - SHO level
120 Director of Public Health
121 Staff Grade
122 Patient Welfare Officer
123 Occupational Therapy Specialist Practitioner
124 Technician - PS&T
125 Chiropodist/Podiatrist Consultant
126 Trust Grade Doctor - Career Grade level
127 Student Community Practitioner
128 Healthcare Scientist
129 Waiting List Clerk
130 Clinical Director
131 Pre-reg Pharmacist
132 Mental Health Act Administrator
133 Ward Clerk
134 Support, Time, Recovery Worker
135 Art Therapist Specialist Practitioner
136 Physiotherapist Manager
137 Healthcare Cadet
138 Dietitian Consultant
139 Orthoptist Manager
140 Social work assistant (mental health)
141 Chiropodist/Podiatrist Specialist Practitioner
142 Student Technician
143 Complaints Investigator
144 Trainee Scientist
145 Radiographer - Diagnostic, Manager
146 Social services care manager (mental health)
147 Dietitian Manager
148 Midwife - Consultant
149 Art Therapist Consultant
150 Paramedic Manager
151 Finance Director
152 Senior social worker (adults)
153 Student Midwife
154 Radiologist
155 Ward Manager
156 Midwife - Manager
157 Waiting List Manager
158 Radiographer - Diagnostic, Specialist Practitioner
159 Biomedical Scientist
160 Board Level Director
161 Non Executive Director
162 Nursing Cadet
163 Porter
164 Social services care manager (adults)
165 Student Psychotherapist
166 Orthoptist
167 Clinical Director - Medical
168 Approved Social Worker
169 Student Community Mental Health Nurse
170 Other Executive Director
171 Student Orthoptist
172 Childcare Co-ordinator
173 House Officer - Pre Registration
174 SODP
175 Outpatient Manager
176 Medical Director
177 Trust Grade Doctor - Specialist Registrar level
178 Senior Clinical Medical Officer
179 Consultant Healthcare Scientist
180 Reporting Radiographer
181 Locum GP
182 Researcher
183 Assistant GP
184 Special salary scale in Public Health Medicine
185 Advanced Practitioner
186 Health Visitor
187 Dental Assistant Clinical Director
188 Other Community Health Service - Social Care Worker
189 Physician Assistant
190 Deputising Doctor
191 Student Occupational Health Nurse
192 Senior social worker (mental health)
193 Regional Dental Officer
194 Trainer
195 Cytoscreener
196 Chair
197 Trust Grade Doctor - House Officer level
198 Art Therapist
199 Multi Therapist Specialist Practitioner
200 Drama Therapist
201 Healthcare Science Assistant
202 Social work assistant (adults)
203 Social work team manager (adults)

For peer review only

204 Intermediate Care worker
205 Student Occupational Therapist
206 Student Dietitian
207 Healthcare Science Associate
208 Child Protection worker
209 Professor
210 General Dental Practitioner
211 Student School Nurse
212 Occupational Therapist Consultant
213 Intermediate Care staff
214 Home help
215 Art, Music & Drama Student
216 Specialist Healthcare Scientist
217 Social Services information manager

For peer review only

Table A2.2: Number of observations by EMIS® consultation source identifier, Consultation source code identifier and Staff Job Category, with an indicator for whether it was included as a consultation: Include: 1 = Include, 0 = Exclude, "Excl. job title" = Excluded on the basis of job title.

Include	Staff Job Category	Consultation source code identifier	EMIS consultation source identifier	Count
1	gp	gp surgery	gp surgery	3,119,080
1	nurse	gp surgery	gp surgery	1,692,606
1	gp	telephone consultation	telephone consultation	1,471,946
1	other care provider	gp surgery	gp surgery	977,067
1	nurse	telephone consultation	telephone consultation	196,665
1	gp	telephone call to a patient	telephone call to a patient	108,025
1	gp	home visit note	home visit note	83,600
1	other care provider	telephone consultation	telephone consultation	81,980
1	nurse	telephone call to a patient	telephone call to a patient	32,423
1	gp	face to face consultation	face to face consultation	25,435
1	nurse	home visit note	home visit note	24,174
1	other care provider	telephone call to a patient	telephone call to a patient	23,194
1	gp	gp surgery	surgery consultation	22,756

1	gp	nursing home visit note	nursing home visit note	21,357
1	nurse	face to face consultation	face to face consultation	17,580
1	gp	enterprise consultation	enterprise consultation	14,904
1	gp	telephone call from a patient	telephone call from a patient	13,062
1	gp	routine consultation	surgery consultation	11,668
1	other care provider	home visit note	home visit note	10,853
1	gp	provision of general practitioner intermediate care	gp surgery	10,441
1	gp	emergency consultation	emergency consultation	10,351
1	gp	residential home visit note	residential home visit note	9,579
1	other care provider	face to face consultation	face to face consultation	9,350
1	gp	emergency appointment	emergency appointment	8,687
1	gp	urgent consultation	urgent consultation	8,155
1	gp	walk-in clinic	walk-in clinic	7,908
1	dr	gp surgery	gp surgery	7,654
1	gp	other note	other	7,520
1	gp	face to face consultation	surgery consultation	6,932
1	nurse	gp surgery	surgery consultation	5,318
1	gp	seen in gp unit	surgery consultation	4,687
1	gp	consultation via video conference	awaiting review	4,653
1	nurse	enterprise consultation	enterprise consultation	4,460
1	other care provider	provision of general practitioner intermediate care	gp surgery	4,369
1	gp	clinic note	surgery consultation	3,823
1	nurse	residential home visit note	residential home visit note	3,612
1	nurse	clinic note	clinic	3,585
1	nurse	nursing home visit note	nursing home visit note	3,528
1	nurse	face to face consultation	surgery consultation	3,442
1	gp	online communication	awaiting review	3,410
1	other care provider	other note	other	3,406
1	other care provider	seen in gp unit	gp surgery	2,781
1	gp	e-mail consultation	awaiting review	2,523
1	nurse	other note	other	2,449
1	other care provider	gp surgery	surgery consultation	2,334
1	other care provider	enterprise consultation	enterprise consultation	2,318
1	other care provider	telephone call from a patient	telephone call from a patient	2,211
1	nurse	telephone call from a patient	telephone call from a patient	2,183
1	gp	routine consultation	awaiting review	2,117
1	nurse	emergency appointment	emergency appointment	2,041
1	gp	home visit note	home visit	2,021
1	gp	seen in gp unit	gp surgery	1,896
1	nurse	provision of general practitioner intermediate care	gp surgery	1,762

For peer review only

1	other care provider	clinic note	clinic	1,699
1	nurse	clinic note	surgery consultation	1,628
1	gp	clinic note	clinic	1,623
1	nurse	routine consultation	surgery consultation	1,578
1	nurse	seen in gp unit	surgery consultation	1,426
1	nurse	walk-in clinic	walk-in clinic	1,412
1	nurse	gp surgery	clinic	1,355
1	other care provider	routine consultation	other	1,303
1	other care provider	clinic note	surgery consultation	1,297
1	gp	face to face consultation	emergency consultation	1,292
1	other care provider	walk-in clinic	walk-in clinic	1,216
1	gp	telephone encounter	telephone encounter	1,184
1	gp	online communication	online services message	1,139
1	gp	other consultation medium used	awaiting review	1,134
1	other care provider	residential home visit note	residential home visit note	1,113
1	nurse	seen in gp unit	gp surgery	1,103
1	other care provider	nursing home visit note	nursing home visit note	1,081
1	other care provider	face to face consultation	surgery consultation	1,045
1	other care provider	seen in gp unit	surgery consultation	1,043
1	nurse	emergency consultation	emergency consultation	1,024
1	nurse	urgent consultation	urgent consultation	959
1	gp	extended hours consultation	awaiting review	924
1	gp	routine consultation	other	922
1	gp	home visit note	other	835
1	gp	gp surgery	face to face consultation	808
1	other care provider	gp surgery	clinic	746
1	other care provider	routine consultation	surgery consultation	738
1	gp	consultation via multimedia	awaiting review	734
1	gp	face to face consultation with relative/carer	awaiting review	669
1	nurse	e-mail consultation	awaiting review	638
1	nurse	routine consultation	awaiting review	574
1	nurse	consultation via video conference	awaiting review	505
1	nurse	routine consultation	other	478
1	other care provider	online communication	awaiting review	473
1	nurse	same day appointment	same day appointment	468
1	gp	face to face consultation	gp surgery	461
1	gp	same day appointment	same day appointment	457
1	nurse	gp surgery	face to face consultation	455
1	gp	telephone encounter	telephone consultation	429
1	nurse	face to face consultation	emergency consultation	420

For peer review only

1	gp	group consultation	awaiting review	402
1	nurse	home visit note	other	402
1	nurse	seen in urgent care centre	awaiting review	344
1	gp	face to face consultation	emergency appointment	330
1	gp	seen in urgent care centre	awaiting review	322
1	nurse	telephone encounter	telephone encounter	314
1	other care provider	consultation via video conference	awaiting review	288
1	gp	telephone consultation	telephone call to a patient	287
1	dr	telephone consultation	telephone consultation	279
1	nurse	online communication	awaiting review	279
1	other care provider	face to face consultation	awaiting review	272
1	other care provider	home visit note	home visit	262
1	other care provider	routine consultation	awaiting review	260
1	nurse	home visit note	home visit	246
1	gp	consultation via sms text message	awaiting review	244
1	other care provider	online communication	online services message	241
1	other care provider	emergency consultation	emergency consultation	235
1	other care provider	emergency appointment	emergency appointment	231
1	gp	telephone encounter	telephone call to a patient	226
1	gp	residential home visit note	residential home	225
1	gp	face to face consultation	awaiting review	224
1	nurse	face to face consultation	gp surgery	221
1	other care provider	gp surgery	face to face consultation	209
1	nurse	extended hours consultation	awaiting review	186
1	gp	seen in rapid access clinic at gp surgery	awaiting review	182
1	gp	school visit note	awaiting review	181
1	other care provider	telephone consultation	telephone call to a patient	179
1	nurse	face to face consultation	awaiting review	166
1	gp	home visit note	acute visit	153
1	nurse	online communication	online services message	145
1	other care provider	group consultation	awaiting review	133
1	gp	seen in gp unit	awaiting review	129
1	dr	clinic note	surgery consultation	125
1	other care provider	telephone encounter	telephone encounter	119
1	other care provider	extended hours consultation	awaiting review	116
1	nurse	face to face consultation with relative/carer	awaiting review	115
1	nurse	seen in gp unit	awaiting review	109
1	gp	night visit note	awaiting review	108

For peer review only

1	nurse	school visit note	awaiting review	107
1	nurse	group consultation	awaiting review	102
1	gp	other consultation medium used	other	99
1	gp	children's home visit note	awaiting review	95
1	gp	telephone consultation	telephone call from a patient	94
1	gp	administration note	other	91
1	nurse	telephone encounter	telephone call to a patient	86
1	other care provider	e-mail consultation	awaiting review	71
1	nurse	consultation via multimedia	awaiting review	65
1	gp	gp surgery	clinic	63
1	nurse	telephone consultation	telephone call to a patient	63
1	other care provider	face to face consultation with relative/carer	awaiting review	62
1	gp	consultation via telemedicine web camera	awaiting review	61
1	dr	telephone call to a patient	telephone call to a patient	60
1	nurse	laboratory result	clinic	55
1	other care provider	other consultation medium used	awaiting review	53
1	nurse	other consultation medium used	awaiting review	51
1	nurse	district nurse visit	awaiting review	48
1	gp	clinic note	gp surgery	47
1	other care provider	clinic note	gp surgery	45
1	other care provider	urgent consultation	urgent consultation	42
1	other care provider	home visit note	other	41
1	other care provider	laboratory result	clinic	40
1	gp	laboratory result	acute visit	39
1	dr	home visit note	home visit note	38
1	other care provider	telephone encounter	telephone call to a patient	38
1	nurse	telephone encounter	telephone consultation	33
1	other care provider	consultation via sms text message	awaiting review	28
1	gp	walk-in clinic	walk-in centre	27
1	nurse	walk-in clinic	walk-in centre	27
1	other care provider	walk-in clinic	clinic	27
1	nurse	children's home visit note	awaiting review	25
1	gp	gp surgery	main surgery	24
1	nurse	residential home visit note	residential home	22
1	other care provider	seen in urgent care centre	awaiting review	22
1	nurse	night visit note	awaiting review	20
1	other care provider	children's home visit note	awaiting review	19
1	other care provider	consultation via multimedia	awaiting review	19

For peer review only

1	gp	home visit note	nursing home visit note	17
1	gp	nursing home visit note	nursing home	17
1	gp	residential home visit note	nursing home	15
1	other care provider	face to face consultation	gp surgery	14
1	other care provider	night visit note	awaiting review	14
1	other care provider	other consultation medium used	other	14
1	nurse	clinic note	gp surgery	13
1	nurse	walk-in clinic	clinic	13
1	nurse	home visit note	acute visit	10
1	nurse	consultation via sms text message	awaiting review	< 10
1	nurse	consultation via telemedicine web camera	awaiting review	< 10
1	dr	consultation via video conference	awaiting review	< 10
1	dr	nursing home visit note	nursing home visit note	< 10
1	other care provider	home visit note	nursing home visit note	< 10
1	dr	other note	other	< 10
1	gp	telephone encounter	telephone call from a patient	< 10
1	gp	twilight visit note	awaiting review	< 10
1	other care provider	laboratory result	acute visit	< 10
1	dr	face to face consultation	face to face consultation	< 10
1	gp	home visit note	follow-up/routine visit	< 10
1	gp	other consultation medium used	casualty attendance	< 10
1	other care provider	home visit note	residential home visit note	< 10
1	other care provider	other note	gp surgery	< 10
1	gp	home visit note	awaiting review	< 10
1	gp	non-consultation medication data	awaiting review	< 10
1	gp	remote consultation	awaiting review	< 10
1	gp	third party consultation	casualty attendance	< 10
1	nurse	home visit note	follow-up/routine visit	< 10
1	nurse	telephone encounter	telephone call from a patient	< 10
1	nurse	third party consultation	casualty attendance	< 10
1	dr	enterprise consultation	enterprise consultation	< 10
1	dr	telephone call from a patient	telephone call from a patient	< 10
1	gp	district nurse visit	awaiting review	< 10
1	gp	e-mail received from patient	acute visit	< 10
1	gp	hospital outpatient report	casualty attendance	< 10
1	gp	joint consultation	awaiting review	< 10
1	gp	pharmacy consultation	awaiting review	< 10
1	gp	telephone call to a patient	telephone consultation	< 10
1	nurse	administration note	other	< 10
1	nurse	face to face consultation	emergency appointment	< 10
1	other care provider	consultation via telemedicine web camera	awaiting review	< 10

For peer review only

1	other care provider	face to face consultation	emergency appointment	< 10
1	other care provider	face to face consultation	emergency consultation	< 10
1	other care provider	home visit note	acute visit	< 10
1	other care provider	pharmacy consultation	awaiting review	< 10
1	other care provider	residential home visit note	residential home	< 10
1	other care provider	telephone encounter	telephone consultation	< 10
1	dr	group consultation	awaiting review	< 10
1	dr	home visit note	acute visit	< 10
1	gp	clinic note	follow-up/routine visit	< 10
1	gp	emergency consultation	casualty attendance	< 10
1	gp	home visit note	nursing home	< 10
1	nurse	seen in rapid access clinic at gp surgery	awaiting review	< 10
1	nurse	telephone consultation	telephone call from a patient	< 10
1	nurse	twilight visit note	awaiting review	< 10
1	other care provider	administration note	other	< 10
1	other care provider	children's home visit note	clinic	< 10
1	other care provider	home visit note	awaiting review	< 10
1	other care provider	twilight visit note	awaiting review	< 10
excl. job cat		gp surgery	gp surgery	875,291
excl. job cat		other note	other	31,332
excl. job cat		telephone consultation	telephone consultation	29,455
excl. job cat		online communication	online services message	14,055
excl. job cat		telephone call to a patient	telephone call to a patient	13,935
excl. job cat		provision of general practitioner intermediate care	gp surgery	9,791
excl. job cat		telephone call from a patient	telephone call from a patient	7,098
excl. job cat		seen in gp unit	gp surgery	6,240
excl. job cat		home visit note	home visit note	4,776
excl. job cat		routine consultation	other	4,248
excl. job cat		gp surgery	surgery consultation	3,047
excl. job cat		home visit note	other	2,664
excl. job cat		face to face consultation	face to face consultation	2,071
excl. job cat		face to face consultation	surgery consultation	1,177
excl. job cat		online communication	awaiting review	1,098
excl. job cat		nursing home visit note	nursing home visit note	708
excl. job cat		routine consultation	surgery consultation	509
excl. job cat		consultation via video conference	awaiting review	494
excl. job cat		routine consultation	awaiting review	453
excl. job cat		enterprise consultation	enterprise consultation	398
excl. job cat		administration note	other	376
excl. job cat		telephone encounter	telephone encounter	376

For peer review only

excl. job cat		home visit note	awaiting review	371
excl. job cat		clinic note	clinic	337
excl. job cat		clinic note	surgery consultation	329
excl. job cat		residential home visit note	residential home visit note	327
excl. job cat		face to face consultation	gp surgery	305
excl. job cat		face to face consultation	awaiting review	293
excl. job cat		home visit note	home visit	250
excl. job cat		urgent consultation	urgent consultation	240
excl. job cat		walk-in clinic	walk-in clinic	196
excl. job cat		group consultation	awaiting review	170
excl. job cat		seen in gp unit	surgery consultation	148
excl. job cat		e-mail consultation	awaiting review	145
excl. job cat		emergency consultation	emergency consultation	121
excl. job cat		face to face consultation with relative/carer	awaiting review	116
excl. job cat		consultation via multimedia	awaiting review	75
excl. job cat		seen in gp unit	awaiting review	75
excl. job cat		children's home visit note	awaiting review	63
excl. job cat		other note	gp surgery	54
excl. job cat		other consultation medium used	other	44
excl. job cat		other consultation medium used	awaiting review	42
excl. job cat		extended hours consultation	awaiting review	37
excl. job cat		gp surgery	face to face consultation	36
excl. job cat		emergency appointment	emergency appointment	35
excl. job cat		gp surgery	clinic	32
excl. job cat		face to face consultation	emergency consultation	25
excl. job cat		residential home visit note	residential home	23
excl. job cat		telephone encounter	telephone call to a patient	23
excl. job cat		night visit note	awaiting review	14
excl. job cat		home visit note	acute visit	13
excl. job cat		walk-in clinic	walk-in centre	11
excl. job cat		district nurse visit	awaiting review	< 10
excl. job cat		seen in urgent care centre	awaiting review	< 10
excl. job cat		twilight visit note	awaiting review	< 10
excl. job cat		clinic note	gp surgery	< 10
excl. job cat		laboratory result	acute visit	< 10
excl. job cat		telephone consultation	telephone call to a patient	< 10
excl. job cat		walk-in clinic	clinic	< 10
excl. job cat		telephone encounter	telephone call from a patient	< 10
excl. job cat		emergency consultation	casualty attendance	< 10
excl. job cat		pharmacy consultation	awaiting review	< 10
excl. job cat		third party consultation	casualty attendance	< 10
excl. job cat		case conference	gp surgery	< 10
excl. job cat		emergency consultation	awaiting review	< 10
excl. job cat		gp surgery	main surgery	< 10
excl. job cat		home visit note	follow-up/routine visit	< 10
excl. job cat		non-consultation medication data	casualty attendance	< 10
excl. job cat		nursing home visit note	nursing home	< 10

For peer review only

excl. job cat		remote consultation	awaiting review	< 10
excl. job cat		same day appointment	same day appointment	< 10
excl. job cat		school visit note	awaiting review	< 10
excl. job cat		telephone consultation	awaiting review	< 10
excl. job cat		telephone encounter	telephone consultation	< 10
excl. job cat		third party consultation	awaiting review	< 10
0	gp	externally entered note	externally entered	3,467,397
0		scanned document	docman	3,183,781
0		administration note	administration note	968,767
0				737,843
0		scanned document	scanned document	727,269
0	gp	administration note	administration note	725,612
0		inbound document	inbound document	402,647
0		awaiting clinical code migration to emis web	awaiting review	385,598
0	nurse	externally entered note	externally entered	303,830
0	gp	awaiting clinical code migration to emis web	awaiting review	261,627
0		administration note	administration	256,604
0	other care provider	scanned document	docman	255,022
0		externally entered note	externally entered	252,167
0	gp			208,462
0	gp	outbound referral	outbound referral	197,534
0	gp	awaiting clinical code migration to emis web	gp surgery	162,048
0	other care provider	administration note	administration note	160,918
0		awaiting clinical code migration to emis web	third party consultation	147,374
0	gp	scanned document	scanned document	145,355
0		awaiting clinical code migration to emis web	gp surgery	127,041
0	gp	awaiting clinical code migration to emis web	results recording	122,118
0		awaiting clinical code migration to emis web	other	121,401
0	gp	awaiting clinical code migration to emis web	surgery consultation	107,304
0		awaiting clinical code migration to emis web		104,693
0	gp	inbound document	inbound document	102,534
0	nurse	awaiting clinical code migration to emis web	awaiting review	94,158
0	other care provider	awaiting clinical code migration to emis web	gp surgery	86,398
0	other care provider	awaiting clinical code migration to emis web	awaiting review	85,745
0	nurse	administration note	administration note	84,051
0	gp	awaiting clinical code migration to emis web		77,585
0	nurse	awaiting clinical code migration to emis web	gp surgery	75,552

For peer review only

0	other care provider			66,366
0	other care provider	externally entered note	externally entered	62,810
0		externally entered note	externally entered note	61,848
0		awaiting clinical code migration to emis web	repeat issue	61,683
0	nurse			58,019
0	gp	telephone triage encounter	telephone triage encounter	50,662
0		third party consultation	third party consultation	44,609
0	other care provider	scanned document	scanned document	38,341
0	nurse	awaiting clinical code migration to emis web		35,376
0	nurse	awaiting clinical code migration to emis web	surgery consultation	27,699
0	gp	administration note	administration	27,390
0	gp	awaiting clinical code migration to emis web	telephone consultation	26,214
0	gp	awaiting clinical code migration to emis web	other	26,204
0		awaiting clinical code migration to emis web	scanned document	25,627
0		awaiting clinical code migration to emis web	administration note	24,458
0		gp surgery	awaiting review	24,337
0		awaiting clinical code migration to emis web	touchscreen	24,311
0	gp	third party consultation	third party consultation	23,952
0		awaiting clinical code migration to emis web	patientchase insert	22,515
0	gp	laboratory result	results recording	22,066
0	gp	externally entered note	externally entered note	20,864
0	gp	awaiting clinical code migration to emis web	administration note	20,729
0	other care provider	inbound document	inbound document	20,159
0	gp	telephone call to relative/carer	telephone call to relative/carer	19,234
0	other care provider	awaiting clinical code migration to emis web		17,081
0		awaiting clinical code migration to emis web	surgery consultation	15,972
0	nurse	awaiting clinical code migration to emis web	clinic	15,892
0	gp	other note	other note	15,816
0	gp	gp surgery	awaiting review	15,810
0	gp	discussion with colleague	discussion with colleague	12,973
0	gp	awaiting clinical code migration to emis web	telephone call to a patient	11,782
0		repeat prescription	repeat issue	11,542
0	other care provider	administration note	administration	11,037
0	nurse	awaiting clinical code migration to emis web	other	11,008

For peer review only

0		hospital outpatient report	hospital outpatient report	10,033
0	nurse	inbound document	inbound document	9,304
0	nurse	scanned document	scanned document	7,878
0	other care provider	awaiting clinical code migration to emis web	repeat issue	7,745
0	gp	awaiting clinical code migration to emis web	outbound referral	7,402
0	other care provider	externally entered note	externally entered note	6,736
0	gp	telephone call from relative/carer	telephone call from relative/carer	6,626
0	other care provider	awaiting clinical code migration to emis web	surgery consultation	6,514
0		awaiting clinical code migration to emis web	inbound document	6,495
0	nurse	gp surgery	awaiting review	6,475
0	other care provider	awaiting clinical code migration to emis web	other	6,315
0	nurse	administration note	administration	6,285
0		outbound referral	outbound referral	6,194
0	nurse	telephone triage encounter	telephone triage encounter	6,074
0	gp	clinic note	clinic note	5,834
0	gp	awaiting clinical code migration to emis web	repeat issue	5,803
0	gp	non-consultation data	non-consultation data	5,552
0	other care provider	third party consultation	third party consultation	5,505
0	other care provider	gp surgery	awaiting review	5,332
0	gp	awaiting clinical code migration to emis web	telephone call from a patient	5,282
0	nurse	externally entered note	externally entered note	5,251
0	gp	face to face consultation	triage	5,196
0	nurse	telephone call to relative/carer	telephone call to relative/carer	5,191
0		awaiting clinical code migration to emis web	mail to patient	5,178
0		non-consultation data	non-consultation data	4,933
0	gp	e-mail received from patient	e-mail received from patient	4,877
0	nurse	outbound referral	outbound referral	4,787
0		medication requested	repeat issue	4,639
0	nurse	third party consultation	third party consultation	4,605
0	nurse	awaiting clinical code migration to emis web	results recording	4,405
0	gp	awaiting clinical code migration to emis web	scanned document	4,384
0		other note	other note	4,375
0		awaiting clinical code migration to emis web	mjog	4,221
0	other care provider	other note	other note	4,071
0		mail to patient	mail to patient	3,924
0	other care provider	awaiting clinical code migration to emis web	clinic	3,859
0		e-mail received from patient	e-mail received from patient	3,632

For peer review only

0		awaiting clinical code migration to emis web	out of hours, non practice	3,607
0	other care provider	clinic note	clinic note	3,371
0	gp	awaiting clinical code migration to emis web	home visit note	3,370
0	gp	hospital outpatient report	hospital outpatient report	3,359
0	nurse	nurse telephone triage	nurse telephone triage	3,274
0	nurse	clinic note	clinic note	3,252
0	gp	awaiting clinical code migration to emis web	clinic	3,245
0	other care provider	awaiting clinical code migration to emis web	administration note	3,044
0	gp	awaiting clinical code migration to emis web	third party consultation	3,038
0	other care provider	awaiting clinical code migration to emis web	medicine management	2,829
0	other care provider	telephone call to relative/carer	telephone call to relative/carer	2,807
0	nurse	awaiting clinical code migration to emis web	telephone consultation	2,782
0		awaiting clinical code migration to emis web	clinic	2,775
0		laboratory result	laboratory result	2,727
0	gp	awaiting clinical code migration to emis web	inbound document	2,605
0		telephone call from relative/carer	telephone call from relative/carer	2,594
0		clinic note	clinic note	2,579
0	gp	laboratory result	laboratory result	2,511
0	other care provider	repeat prescription	repeat issue	2,511
0		awaiting clinical code migration to emis web	home of patient	2,454
0	gp	awaiting clinical code migration to emis web	out of hours, non practice	2,365
0	gp	awaiting clinical code migration to emis web	nhs direct report	2,334
0		awaiting clinical code migration to emis web	results recording	2,185
0	dr	third party consultation	third party consultation	2,146
0	other care provider	awaiting clinical code migration to emis web	third party consultation	2,095
0	nurse	awaiting clinical code migration to emis web	administration note	2,075
0		administration note	patientchase insert	1,855
0		clinic note	awaiting review	1,790
0		hospital inpatient report	hospital inpatient report	1,760
0		e-mail received from patient	docman	1,753
0	nurse	other note	other note	1,708
0	nurse	non-consultation data	non-consultation data	1,681
0	gp	awaiting clinical code migration to emis web	referral letter	1,660
0		laboratory result	results recording	1,624

For peer review only

0		awaiting clinical code migration to emis web	non-consultation data	1,537
0	gp	awaiting clinical code migration to emis web	medicine management	1,510
0	nurse	laboratory result	results recording	1,464
0	nurse	telephone call from relative/carer	telephone call from relative/carer	1,401
0		administration note	scanned document	1,373
0		telephone triage encounter	telephone triage encounter	1,353
0	gp	medication requested	awaiting review	1,347
0	nurse	discussion with colleague	discussion with colleague	1,329
0	gp	awaiting clinical code migration to emis web	telephone triage encounter	1,313
0	nurse	awaiting clinical code migration to emis web	telephone call to a patient	1,243
0	other care provider	non-consultation data	non-consultation data	1,239
0	gp	case conference	awaiting review	1,225
0	gp	awaiting clinical code migration to emis web	externally entered note	1,206
0		telephone call to relative/carer	telephone call to relative/carer	1,191
0	nurse	awaiting clinical code migration to emis web	third party consultation	1,170
0	gp	e-mail sent to patient	awaiting review	1,162
0	dr	administration note	administration	1,159
0		other note		1,156
0		administration note		1,120
0	dr	externally entered note	externally entered	1,113
0	gp	awaiting clinical code migration to emis web	face to face consultation	1,112
0	other care provider	telephone triage encounter	telephone triage encounter	1,107
0	nurse	laboratory result	laboratory result	1,064
0	other care provider	outbound referral	outbound referral	1,052
0	gp	clinic note	awaiting review	1,030
0	other care provider	mail to patient	patientchase insert	1,019
0	gp	repeat prescription	awaiting review	1,003
0	gp	awaiting clinical code migration to emis web	data transferred from other system	983
0		awaiting clinical code migration to emis web	non-consultation medication data	980
0	gp	awaiting clinical code migration to emis web	nursing home visit note	971
0	gp	repeat prescription	repeat issue	964
0		awaiting clinical code migration to emis web	telephone call to a patient	901
0	dr			885
0	nurse	awaiting clinical code migration to emis web	face to face consultation	853
0	other care provider	hospital outpatient report	hospital outpatient report	828
0		clinic note	community clinic	823

For peer review only

0		awaiting clinical code migration to emis web	gp2gp import	822
0		awaiting clinical code migration to emis web	letter from outpatients	809
0	other care provider	mail to patient	mail to patient	801
0	other care provider	telephone call from relative/carer	telephone call from relative/carer	789
0		awaiting clinical code migration to emis web	externally entered note	769
0		awaiting clinical code migration to emis web	referral letter	751
0	other care provider	awaiting clinical code migration to emis web	inbound document	719
0		e-mail sent to patient	awaiting review	711
0	gp	hospital inpatient report	hospital inpatient report	710
0	nurse	awaiting clinical code migration to emis web	repeat issue	694
0	gp	awaiting clinical code migration to emis web	acute visit	693
0	gp	awaiting clinical code migration to emis web	telephone call to relative/carer	680
0		non-consultation medication data	repeat issue	679
0	nurse	awaiting clinical code migration to emis web	home visit note	672
0	other care provider	discussion with colleague	discussion with colleague	665
0		administration note	docman	665
0	gp	other note		661
0		awaiting clinical code migration to emis web	telephone consultation	656
0	other care provider	awaiting clinical code migration to emis web	scanned document	642
0		ooh report	third party consultation	637
0	gp	scanned document	externally entered	619
0	gp	medication requested	repeat issue	606
0	nurse	awaiting clinical code migration to emis web	telephone triage encounter	602
0	other care provider	awaiting clinical code migration to emis web	telephone call to a patient	541
0	other care provider	awaiting clinical code migration to emis web	face to face consultation	534
0	dr	administration note	administration note	525
0	gp	discussion with other professional	awaiting review	514
0	gp	administration note	awaiting review	509
0	other care provider	awaiting clinical code migration to emis web	mail to patient	507
0		ooh report	awaiting review	504
0	dr	awaiting clinical code migration to emis web	gp surgery	501
0		walk-in clinic		495
0		administration note	inbound document	493
0		routine consultation	repeat issue	488

For peer review only

0		awaiting clinical code migration to emis web	telephone call from a patient	486
0	gp	ooh report	out of hours, non practice	478
0	gp	mail from patient	mail from patient	473
0		other note	awaiting review	472
0	gp	awaiting clinical code migration to emis web	enterprise consultation	458
0	other care provider	repeat prescription	awaiting review	455
0	gp	awaiting clinical code migration to emis web	discussion with colleague	451
0		awaiting clinical code migration to emis web	outbound referral	450
0	gp	awaiting clinical code migration to emis web	nursing home	447
0	dr	awaiting clinical code migration to emis web	awaiting review	444
0	nurse	e-mail received from patient	e-mail received from patient	443
0		awaiting clinical code migration to emis web	data transferred from other system	442
0	nurse	ooh report	awaiting review	439
0		administration note	awaiting review	436
0	other care provider	clinic note	awaiting review	431
0	gp	administration note		415
0	gp	administration note	scanned document	414
0	gp	awaiting clinical code migration to emis web	urgent consultation	404
0	other care provider	awaiting clinical code migration to emis web	externally entered note	385
0		awaiting clinical code migration to emis web	school	384
0	nurse	hospital outpatient report	hospital outpatient report	380
0		mail to patient	patientchase insert	377
0		awaiting clinical code migration to emis web	telephone encounter	376
0	gp	administration note	results recording	372
0		discussion with colleague	discussion with colleague	368
0	other care provider	awaiting clinical code migration to emis web	results recording	365
0		case conference	awaiting review	361
0	other care provider	awaiting clinical code migration to emis web	telephone consultation	350
0	other care provider	awaiting clinical code migration to emis web	patientchase insert	345
0		sms text message sent to patient	patientchase insert	345
0	nurse	mail to patient	mail to patient	342
0		awaiting clinical code migration to emis web	hospital outpatient report	336
0		gp surgery		315
0	gp	ooh report	nhs direct report	314
0	gp	administration note	referral letter	313
0	dr	awaiting clinical code migration to emis web		310

For peer review only

0	gp	awaiting clinical code migration to emis web	telephone call from relative/carer	310
0	gp	awaiting clinical code migration to emis web	non-consultation data	309
0	nurse	awaiting clinical code migration to emis web	telephone call from a patient	309
0	other care provider	awaiting clinical code migration to emis web	non-consultation data	306
0	other care provider	e-mail received from patient	e-mail received from patient	292
0	other care provider	awaiting clinical code migration to emis web	non-consultation medication data	289
0	nurse	clinic note	awaiting review	286
0	dr	scanned document	scanned document	285
0		ooh report	out of hours, non practice	283
0	other care provider	laboratory result	laboratory result	282
0	gp	awaiting clinical code migration to emis web	discharge details	281
0		home visit note	home of patient	277
0	nurse	awaiting clinical code migration to emis web	inbound document	276
0	other care provider	hospital inpatient report	hospital inpatient report	275
0	gp	mail to patient	mail to patient	270
0	gp	awaiting clinical code migration to emis web	residential home visit note	266
0	other care provider	administration note	awaiting review	265
0		awaiting clinical code migration to emis web	acute visit	263
0		walk-in clinic	awaiting review	260
0	dr	hospital outpatient report	hospital outpatient report	256
0	gp	awaiting clinical code migration to emis web	mail to patient	249
0		administration note	mjog	248
0		mail from patient	mail from patient	248
0	gp	awaiting clinical code migration to emis web	letter from outpatients	247
0		awaiting clinical code migration to emis web	discharge details	244
0	nurse	awaiting clinical code migration to emis web	mail to patient	243
0	other care provider	awaiting clinical code migration to emis web	home visit note	236
0	gp	home visit note	results recording	235
0	dr	inbound document	inbound document	234
0	gp	awaiting clinical code migration to emis web	other note	230
0	gp	awaiting clinical code migration to emis web	patientchase insert	229
0	dr	awaiting clinical code migration to emis web	administration note	225
0	gp	awaiting clinical code migration to emis web	open door surgery	222

0		awaiting clinical code migration to emis web	nhs direct report	222
0	other care provider	awaiting clinical code migration to emis web	touchscreen	216
0	gp	scanned document	docman	213
0	nurse	awaiting clinical code migration to emis web	nursing home visit note	210
0	nurse	administration note		209
0	nurse	awaiting clinical code migration to emis web	externally entered note	209
0		awaiting clinical code migration to emis web	laboratory result	199
0		hospital outpatient report	hospital	197
0	other care provider	non-consultation medication data	repeat issue	196
0	other care provider	awaiting clinical code migration to emis web	referral letter	195
0	nurse	e-mail sent to patient	awaiting review	191
0	nurse	awaiting clinical code migration to emis web	referral letter	190
0	dr	awaiting clinical code migration to emis web	telephone consultation	186
0	nurse	case conference	awaiting review	185
0	other care provider	awaiting clinical code migration to emis web	data transferred from other system	184
0	gp	multidisciplinary team meeting without patient	awaiting review	179
0	gp	awaiting clinical code migration to emis web	clinic note	176
0	other care provider	awaiting clinical code migration to emis web	mjog	175
0		awaiting clinical code migration to emis web	hospital inpatient report	168
0		awaiting clinical code migration to emis web	home visit note	164
0		awaiting clinical code migration to emis web	online services message	163
0	other care provider	administration note		162
0	nurse	awaiting clinical code migration to emis web	outbound referral	159
0	other care provider	ooh report	awaiting review	156
0	gp	awaiting clinical code migration to emis web	non-consultation medication data	152
0	nurse	awaiting clinical code migration to emis web	telephone call to relative/carer	152
0	other care provider	other note	awaiting review	149
0	dr	awaiting clinical code migration to emis web	surgery consultation	148
0	nurse	awaiting clinical code migration to emis web	scanned document	146
0	gp	administration note	repeat issue	145
0		awaiting clinical code migration to emis web	open door surgery	144

0	other care provider	awaiting clinical code migration to emis web	clinic note	140
0		inbound referral	awaiting review	138
0		awaiting clinical code migration to emis web	clinic note	133
0		awaiting clinical code migration to emis web	face to face consultation	129
0	other care provider	non-consultation medication data	non-consultation medication data	124
0	nurse	awaiting clinical code migration to emis web	residential home visit note	122
0	nurse	awaiting clinical code migration to emis web	data transferred from other system	121
0	other care provider	e-mail sent to patient	awaiting review	121
0	gp	administration note	outbound referral	118
0	nurse	other note		117
0	nurse	awaiting clinical code migration to emis web	nurse telephone triage	116
0		awaiting clinical code migration to emis web	accident & emergency	116
0	gp	other consultation medium used	data transferred from other system	109
0		non-consultation medication data	non-consultation medication data	108
0	nurse	face to face consultation	triage	104
0	other care provider	awaiting clinical code migration to emis web	acute visit	102
0	nurse	awaiting clinical code migration to emis web	telephone call from relative/carer	100
0		extended hours consultation	out of hours, non practice	100
0	nurse	mail from patient	mail from patient	98
0	other care provider	hospital outpatient report	hospital	97
0	dr	awaiting clinical code migration to emis web	nursing home visit note	96
0		nurse telephone triage	nurse telephone triage	94
0	nurse	hospital inpatient report	hospital inpatient report	93
0	other care provider	case conference	awaiting review	92
0	gp	provision of general practitioner intermediate care	awaiting review	91
0	gp	walk-in clinic		91
0		hospital outpatient report	letter from outpatients	91
0	other care provider	other note		89
0	gp	administration note	other note	87
0	other care provider	medication requested	awaiting review	85
0	dr	laboratory result	results recording	84
0		awaiting clinical code migration to emis web	mail from patient	84
0		awaiting clinical code migration to emis web	health centre	80
0	gp	administration note	inbound document	76
0	gp	other note	awaiting review	76

For peer review only

0	nurse	walk-in clinic		76
0		other note	non-consultation medication data	76
0	other care provider	awaiting clinical code migration to emis web	nhs direct report	73
0		administration note	repeat issue	73
0	gp	ooh report	awaiting review	70
0	gp	weekly care home ward round	awaiting review	70
0		awaiting clinical code migration to emis web	medicine management	70
0	other care provider	awaiting clinical code migration to emis web	open door surgery	69
0	other care provider	awaiting clinical code migration to emis web	telephone encounter	69
0		medication requested	awaiting review	68
0	other care provider	awaiting clinical code migration to emis web	telephone call to relative/carer	65
0	dr	awaiting clinical code migration to emis web	repeat issue	64
0	gp	non-consultation medication data	repeat issue	64
0	other care provider	awaiting clinical code migration to emis web	telephone call from a patient	64
0	gp	clinic note	nhs direct report	62
0	gp	non-consultation medication data	medicine management	61
0	other care provider	awaiting clinical code migration to emis web	letter from outpatients	61
0	gp	hospital outpatient report	letter from outpatients	60
0	gp	non-consultation data	data transferred from other system	57
0	nurse	awaiting clinical code migration to emis web	enterprise consultation	57
0	gp	outbound referral	referral letter	55
0	other care provider	repeat prescription	medicine management	55
0	nurse	seen in influenza vaccination clinic	awaiting review	54
0		administration note	results recording	54
0		awaiting clinical code migration to emis web	telephone call from relative/carer	54
0	gp	awaiting clinical code migration to emis web	hospital outpatient report	53
0	gp	gp surgery		53
0	gp	third party consultation	out of hours, non practice	51
0		awaiting clinical code migration to emis web	telephone call to relative/carer	51
0		clinic note	out of hours, non practice	51
0		face to face consultation	primary care centre	51
0	gp	awaiting clinical code migration to emis web	laboratory result	50
0	nurse	non-consultation medication data	repeat issue	48
0		awaiting clinical code migration to emis web	other note	48
0		face to face consultation	triage	48
0	gp	progress report	nhs direct report	47
0	other care provider	ooh report	nhs direct report	47

0	nurse	awaiting clinical code migration to emis web	non-consultation data	46
0		awaiting clinical code migration to emis web	nursing home	46
0	gp	awaiting clinical code migration to emis web	mail from patient	45
0	nurse	awaiting clinical code migration to emis web	discussion with colleague	45
0		awaiting clinical code migration to emis web	e-mail received from patient	45
0	nurse	awaiting clinical code migration to emis web	medicine management	43
0	nurse	medication requested	repeat issue	42
0	dr	mail to patient	mail to patient	40
0	gp	awaiting clinical code migration to emis web	encompass message	39
0	gp	other note	data transferred from other system	39
0	nurse	awaiting clinical code migration to emis web	clinic note	39
0	nurse	awaiting clinical code migration to emis web	laboratory result	39
0	other care provider	awaiting clinical code migration to emis web	other note	39
0	other care provider	medication requested	repeat issue	38
0	gp	administration note	non-consultation data	37
0	other care provider	mail from patient	mail from patient	37
0	dr	awaiting clinical code migration to emis web	results recording	36
0	gp	awaiting clinical code migration to emis web	e-mail received from patient	36
0	nurse	awaiting clinical code migration to emis web	walk-in centre	36
0	other care provider	awaiting clinical code migration to emis web	gp2gp import	36
0		administration note	mail to patient	36
0	gp	awaiting clinical code migration to emis web	home of patient	35
0	gp	awaiting clinical code migration to emis web	casualty attendance	34
0	gp	inbound document	letter from outpatients	34
0	other care provider	awaiting clinical code migration to emis web	outbound referral	34
0	other care provider	extended hours consultation	out of hours, non practice	34
0	gp	awaiting clinical code migration to emis web	telephone encounter	33
0	gp	seen in hospital ward	awaiting review	33
0		other note	non-consultation data	33
0	nurse	other note	awaiting review	32
0	other care provider	awaiting clinical code migration to emis web	discussion with colleague	32
0		discussion with other professional	awaiting review	32

For peer review only

0		multidisciplinary team meeting without patient	awaiting review	32
0		children's home visit note		31
0	other care provider	laboratory result	results recording	30
0		awaiting clinical code migration to emis web	casualty attendance	30
0		extended hours consultation	nhs direct report	30
0	nurse	administration note	awaiting review	29
0	nurse	awaiting clinical code migration to emis web	letter from outpatients	29
0	nurse	awaiting clinical code migration to emis web	open door surgery	29
0	nurse	discussion with other professional	awaiting review	29
0	nurse	ooh report	out of hours, non practice	29
0		face to face consultation	treatment room	29
0	gp	email received from carer	awaiting review	28
0	other care provider	awaiting clinical code migration to emis web	telephone triage encounter	28
0		administration note	touchscreen	28
0	nurse	awaiting clinical code migration to emis web	encompass message	27
0	nurse	awaiting clinical code migration to emis web	nursing home	27
0		third party consultation	out of hours, non practice	27
0	gp	awaiting clinical code migration to emis web	follow-up/routine visit	26
0	other care provider	emergency consultation	accident & emergency	26
0	gp	hospital outpatient report	nhs direct report	25
0	other care provider	awaiting clinical code migration to emis web	telephone call from relative/carer	23
0	other care provider	outbound referral	referral letter	23
0		externally entered note	scanned document	23
0	nurse	multidisciplinary team meeting without patient	awaiting review	22
0		other consultation medium used	data transferred from other system	22
0	dr	awaiting clinical code migration to emis web	outbound referral	21
0	gp	seen in influenza vaccination clinic	awaiting review	21
0	nurse	awaiting clinical code migration to emis web	other note	21
0		ooh report	nhs direct report	21
0	dr	awaiting clinical code migration to emis web	inbound document	20
0	gp	awaiting clinical code migration to emis web	day case report	20
0	other care provider	seen by general practitioner with special interest in ear nose and throat disorders	data transferred from other system	20
0		emergency consultation	accident & emergency	20
0	gp	awaiting clinical code migration to emis web	online services message	19

For peer review only

0	gp	hospital outpatient report	third party consultation	19
0	nurse	repeat prescription	awaiting review	19
0		awaiting clinical code migration to emis web	walk in centre	19
0	dr	hospital inpatient report	hospital inpatient report	18
0	other care provider	awaiting clinical code migration to emis web	residential home visit note	18
0	other care provider	walk-in clinic		18
0		awaiting clinical code migration to emis web	residential home visit note	18
0		awaiting clinical code migration to emis web	telephone triage encounter	18
0	dr	outbound referral	outbound referral	17
0	gp	ooh report	third party consultation	17
0	gp	other consultation medium used	nhs direct report	17
0	gp	radiology result	awaiting review	17
0	gp	telephone consultation	telephone call from relative/carer	17
0	other care provider	awaiting clinical code migration to emis web	enterprise consultation	17
0	other care provider	walk-in clinic	walk in centre	17
0		administration note	referral letter	17
0	dr	other note	other note	16
0	other care provider	awaiting clinical code migration to emis web	nursing home visit note	16
0	dr	medication requested	repeat issue	15
0	other care provider	administration note	inbound document	15
0	other care provider	awaiting clinical code migration to emis web	out of hours, non practice	15
0		non-consultation data	data transferred from other system	15
0	other care provider	face to face consultation	triage	15
0	dr	awaiting clinical code migration to emis web	telephone call to a patient	14
0	dr	externally entered note	externally entered note	14
0	gp	hospital inpatient report	discharge details	14
0	gp	seen by general practitioner with special interest in ear nose and throat disorders	data transferred from other system	14
0	other care provider	administration note	scanned document	14
0	other care provider	discussion with other professional	awaiting review	14
0		awaiting clinical code migration to emis web	out of hours, practice	14
0		externally entered note		14
0	dr	telephone call to relative/carer	telephone call to relative/carer	13
0	nurse	awaiting clinical code migration to emis web	nhs direct report	13
0	nurse	awaiting clinical code migration to emis web	out of hours, non practice	13
0		hospital inpatient note	awaiting review	13

For peer review only

0		repeat prescription	awaiting review	13
0	gp	other note	encompass message	12
0	nurse	awaiting clinical code migration to emis web	hospital outpatient report	12
0	other care provider	awaiting clinical code migration to emis web	accident & emergency	12
0	nurse	other note	encompass message	11
0	nurse	repeat prescription	repeat issue	11
0	other care provider	seen in influenza vaccination clinic	awaiting review	11
0		awaiting clinical code migration to emis web	discussion with colleague	11
0		awaiting clinical code migration to emis web	hospital outpatient consultation	11
0		awaiting clinical code migration to emis web	walk-in clinic	11
0		non-consultation medication data	medicine management	11
0		provision of general practitioner intermediate care	awaiting review	10
0	dr	clinic note	awaiting review	< 10
0	other care provider	ooh report	out of hours centre	< 10
0		administration note	non-consultation data	< 10
0		clinic note	nhs direct report	< 10
0		telephone follow-up	awaiting review	< 10
0	gp	administration note	clinic note	< 10
0	gp	progress report	awaiting review	< 10
0	nurse	awaiting clinical code migration to emis web	acute visit	< 10
0	other care provider	multidisciplinary team meeting without patient	awaiting review	< 10
0		administration note	other note	< 10
0	dr	awaiting clinical code migration to emis web	home visit note	< 10
0	gp	hospital inpatient note	day case report	< 10
0	gp	inbound document	discharge details	< 10
0	nurse	awaiting clinical code migration to emis web	follow-up/routine visit	< 10
0	other care provider	awaiting clinical code migration to emis web	discharge details	< 10
0		awaiting clinical code migration to emis web	enterprise consultation	< 10
0		awaiting clinical code migration to emis web	nursing home visit note	< 10
0		email received from carer	awaiting review	< 10
0		ooh report	out of hours, practice	< 10
0		other note	data transferred from other system	< 10
0		outbound referral	referral letter	< 10
0		radiology result	awaiting review	< 10
0	dr	gp surgery	awaiting review	< 10
0	gp	routine consultation	repeat issue	< 10
0	nurse	email received from carer	awaiting review	< 10
0	nurse	non-consultation data	data transferred from other system	< 10

For peer review only

0	nurse	scanned document	docman	< 10
0	other care provider	awaiting clinical code migration to emis web	home of patient	< 10
0	other care provider	hospital outpatient report	letter from outpatients	< 10
0	dr	discussion with colleague	discussion with colleague	< 10
0	gp	administration note	discussion with colleague	< 10
0	gp	awaiting clinical code migration to emis web	out of hours, practice	< 10
0	gp	clinic note	out of hours, non practice	< 10
0	gp	ooh report	out of hours, practice	< 10
0	gp	seen in diabetic clinic	awaiting review	< 10
0	gp	sms text message sent to patient	awaiting review	< 10
0	nurse	administration note	discussion with colleague	< 10
0	other care provider	awaiting clinical code migration to emis web	nursing home	< 10
0	other care provider	awaiting clinical code migration to emis web	online services message	< 10
0		awaiting clinical code migration to emis web	bulk operation	< 10
0		non-consultation data	touchscreen	< 10
0	dr	awaiting clinical code migration to emis web	hospital outpatient report	< 10
0	dr	awaiting clinical code migration to emis web	other	< 10
0	dr	awaiting clinical code migration to emis web	third party consultation	< 10
0	dr	clinic note	clinic note	< 10
0	dr	non-consultation data	non-consultation data	< 10
0	gp	awaiting clinical code migration to emis web	hospital inpatient report	< 10
0	gp	clinic note	community clinic	< 10
0	nurse	administration note	non-consultation data	< 10
0	nurse	awaiting clinical code migration to emis web	non-consultation medication data	< 10
0	other care provider	externally entered note	data transferred from other system	< 10
0	other care provider	gp surgery		< 10
0	other care provider	other note	referral letter	< 10
0		awaiting clinical code migration to emis web	nurseries/playgroup	< 10
0		hospital outpatient report	third party consultation	< 10
0	gp	nurse telephone triage	nurse telephone triage	< 10
0	dr	awaiting clinical code migration to emis web	discussion with colleague	< 10
0	dr	awaiting clinical code migration to emis web	medicine management	< 10
0	dr	awaiting clinical code migration to emis web	residential home visit note	< 10
0	dr	e-mail received from patient	e-mail received from patient	< 10
0	dr	telephone call from relative/carer	telephone call from relative/carer	< 10

For peer review only

0	gp	awaiting clinical code migration to emis web	walk-in centre	< 10
0	gp	extended hours consultation	out of hours, non practice	< 10
0	gp	externally entered note		< 10
0	gp	other consultation medium used	other note	< 10
0	gp	telephone consultation	telephone call to relative/carer	< 10
0	nurse	awaiting clinical code migration to emis web	telephone encounter	< 10
0	nurse	medication requested	awaiting review	< 10
0	nurse	telephone follow-up	awaiting review	< 10
0	other care provider	awaiting clinical code migration to emis web	e-mail received from patient	< 10
0	other care provider	awaiting clinical code migration to emis web	mail from patient	< 10
0	other care provider	awaiting clinical code migration to emis web	out of hours, practice	< 10
0	other care provider	other note	data transferred from other system	< 10
0		awaiting clinical code migration to emis web	day case report	< 10
0		awaiting clinical code migration to emis web	encompass message	< 10
0		e-mail encounter to carer	awaiting review	< 10
0		walk-in clinic	walk in centre	< 10
0	dr	awaiting clinical code migration to emis web	non-consultation data	< 10
0	dr	awaiting clinical code migration to emis web	other note	< 10
0	dr	e-mail sent to patient	awaiting review	< 10
0	gp	email received from third party	awaiting review	< 10
0	gp	hospital inpatient note	awaiting review	< 10
0	gp	night visit note	night visit	< 10
0	gp	other note	third party consultation	< 10
0	gp	telephone encounter	telephone call to relative/carer	< 10
0	gp	telephone follow-up	awaiting review	< 10
0	nurse	administration note	referral letter	< 10
0	nurse	awaiting clinical code migration to emis web	urgent consultation	< 10
0	nurse	seen in asthma clinic	awaiting review	< 10
0	nurse	seen in hospital ward	awaiting review	< 10
0	nurse	telephone consultation	telephone call to relative/carer	< 10
0	other care provider	administration note	other note	< 10
0	other care provider	administration note	referral letter	< 10
0	other care provider	administration note	repeat issue	< 10
0	other care provider	administration note	results recording	< 10
0	other care provider	awaiting clinical code migration to emis web	casualty attendance	< 10
0	other care provider	awaiting clinical code migration to emis web	diabetic clinic	< 10

For peer review only

0	other care provider	awaiting clinical code migration to emis web	encompass message	< 10
0	other care provider	clinic note	nhs direct report	< 10
0	other care provider	seen in diabetic clinic	awaiting review	< 10
0		awaiting clinical code migration to emis web	other report	< 10
0		child in need meeting	awaiting review	< 10
0		hospital inpatient report	hospital inpatient	< 10
0		progress report	nhs direct report	< 10
0		repeat prescription	non-consultation medication data	< 10
0		weekly care home ward round	awaiting review	< 10
0	dr	awaiting clinical code migration to emis web	non-consultation medication data	< 10
0	dr	awaiting clinical code migration to emis web	telephone call from a patient	< 10
0	dr	awaiting clinical code migration to emis web	telephone call from relative/carer	< 10
0	dr	awaiting clinical code migration to emis web	telephone call to relative/carer	< 10
0	gp	awaiting clinical code migration to emis web	night visit , practice	< 10
0	gp	awaiting clinical code migration to emis web	walk-in clinic	< 10
0	gp	non-consultation medication data	non-consultation medication data	< 10
0	gp	other consultation medium used	referral letter	< 10
0	gp	other note	non-consultation data	< 10
0	gp	other note	non-consultation medication data	< 10
0	gp	seen in baby clinic	awaiting review	< 10
0	gp	telephone encounter	telephone call from relative/carer	< 10
0	nurse	administration note	clinic note	< 10
0	nurse	administration note	laboratory result	< 10
0	nurse	awaiting clinical code migration to emis web	casualty attendance	< 10
0	nurse	awaiting clinical code migration to emis web	patientchase insert	< 10
0	nurse	clinic note	community clinic	< 10
0	nurse	extended hours consultation	out of hours, non practice	< 10
0	nurse	externally entered note	data transferred from other system	< 10
0	nurse	first attendance face to face	awaiting review	< 10
0	nurse	hospital inpatient note	awaiting review	< 10
0	nurse	ooh report	out of hours, practice	< 10
0	nurse	other consultation medium used	data transferred from other system	< 10
0	nurse	other note	data transferred from other system	< 10
0	nurse	other note	referral letter	< 10
0	nurse	outbound referral	referral letter	< 10
0	nurse	patient initiated enc. nos	awaiting review	< 10
0	nurse	telephone consultation	telephone call from relative/carer	< 10
0	nurse	telephone encounter	telephone call to relative/carer	< 10
0	nurse	weekly care home ward round	awaiting review	< 10

For peer review only

0	other care provider	administration note	mail to patient	< 10
0	other care provider	awaiting clinical code migration to emis web	hospital inpatient report	< 10
0	other care provider	awaiting clinical code migration to emis web	hospital outpatient report	< 10
0	other care provider	awaiting clinical code migration to emis web	laboratory result	< 10
0	other care provider	clinic note	community clinic	< 10
0	other care provider	first attendance face to face	awaiting review	< 10
0	other care provider	inbound referral	awaiting review	< 10
0	other care provider	non-consultation data	data transferred from other system	< 10
0	other care provider	ooh report	out of hours, non practice	< 10
0	other care provider	telephone encounter	awaiting review	< 10
0		awaiting clinical code migration to emis web	follow-up/routine visit	< 10
0		awaiting clinical code migration to emis web	letter	< 10
0		awaiting clinical code migration to emis web	minor injuries unit	< 10
0		awaiting clinical code migration to emis web	secretary	< 10
0		email received from third party	awaiting review	< 10
0		gp surgery	non-consultation data	< 10
0		hospital inpatient report	awaiting review	< 10
0		hospital inpatient report	discharge details	< 10
0		multidisciplinary team meeting with patient	awaiting review	< 10
0		night visit note	night visit, local rota	< 10
0		provision of general practitioner intermediate care	gp2gp import	< 10
0		telephone triage encounter	nhs direct report	< 10
0		third party consultation	third party	< 10
0	other care provider	nurse telephone triage	nurse telephone triage	< 10

For peer review only

Annex 3: a list of terms excluded to help identify true vaccinations in the data

Figure A3.1 Consultation rates including and excluding influenza vaccine in CPRD Aurum data, 01 Jan 2019 – 30 Jan 2021

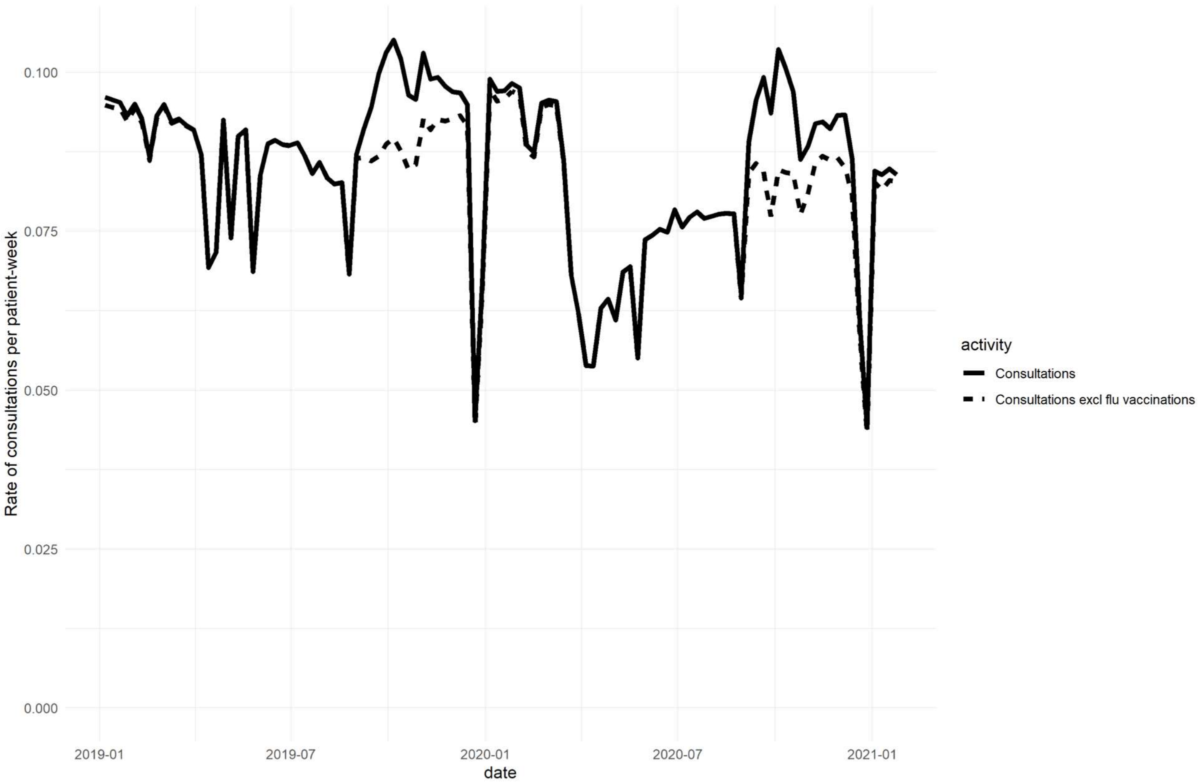

<u>A list of terms excluded to help identify flu vaccinations in the data</u>

We excluded observations in the data that the data provider identified as relating to vaccines. We then searched for the words flu and influenza in the medical term and excluded those that also contained the following words:

Letter, decline, consent, needs, request, missed, invite, needed, required, status, immunity, immune, advised, due, contact tracing, required, education, booked, up to date, did not attend, contraindicated, not indicated, invitation, history of, can be, indication, has flu vaccination at, reason for, other healthcare provider, given by pharmacist, recommend.

Annex 4: *NHS targets for cancer wait times*

			Operational standard
Maximum two weeks from	Receipt of urgent referral for suspected cancer to first outpatient attendance		93%
	Receipt of referral of any patient with breast symptoms (where cancer not suspected) to first hospital assessment		93%
Maximum 28 days from	Receipt of two week wait referral for suspected cancer, receipt of urgent referral from a cancer screening programme (breast, bowel, cervical), and receipt of two week wait referral of any patient with breast symptoms (where cancer not suspected), to the date the patient is informed of a diagnosis or ruling out of cancer		75%
Maximum one month (31 days) from:	Decision to treat to first definitive treatment		96%
	Decision to treat/earliest clinically appropriate date to start of second or subsequent treatment(s) for all cancer patients including those diagnosed with a recurrence where the subsequent treatment is:	surgery	94%
		drug treatment	98%
		radiotherapy	94%
Maximum two months (62 days) from:	Urgent referral for suspected cancer to first treatment (62-day classic)		85%
	Urgent referral from a NHS Cancer Screening Programme (breast, cervical or bowel) for suspected cancer to first treatment		90%

eview only

Annex 5: Patient demographics and person-time and total numbers of observed activity in CPRD Aurum sample

Table A5.1: Patient demographics in CPRD Aurum sample as at 22 March 2020

	Patient count as at 22 March 2020	
	n	%
All	375,501	
Female	187,509	49.9%
Male	187,992	50.1%
Under 11	39,611	10.5%
11 to 19	43,406	11.6%
20 to 49	157,962	42.1%
50 to 69	87,482	23.3%
70 and older	47,040	12.5%
IMD Quintile - 1	78,759	21.0%
IMD Quintile - 2	73,046	19.5%
IMD Quintile - 3	71,840	19.1%
IMD Quintile - 4	77,545	20.7%
IMD Quintile - 5	74,020	19.7%
IMD not recorded	291	0.1%

Table A5.2: Person time (weeks) and total primary care activity analysed - CPRD Aurum

	3 January 2016 - 21March 2020*				22 March 2020 - 29 January 2021			
	Patient-time (weeks)	100,000 person-months	Activity count	Observed rate per 100,000 person-months	Patient-time (weeks)	100,000 person-months	Activity count	Observed rate per 100,000 person-months
Consultations excl flu vaccinations	78,868,977	185.03	6,703,553	36,230	16,701,707	38.07	1,247,981	32,779
Routine Referrals	78,868,977	185.03	312,422	1,688	16,701,707	38.07	40,744	1,070
2 Week Wait Referrals	78,868,977	185.03	38,905	210.3	16,701,707	38.07	10,235	268.8

**The pre-pandemic period consists of 220 weeks and 51.61 months, the post pandemic period is 45 weeks and 10.26 months*

For peer review only

Annex 6: Observed vs expected appointment and cancer diagnosis counts from CWT data from 01 Jan 2019 (per person-month)

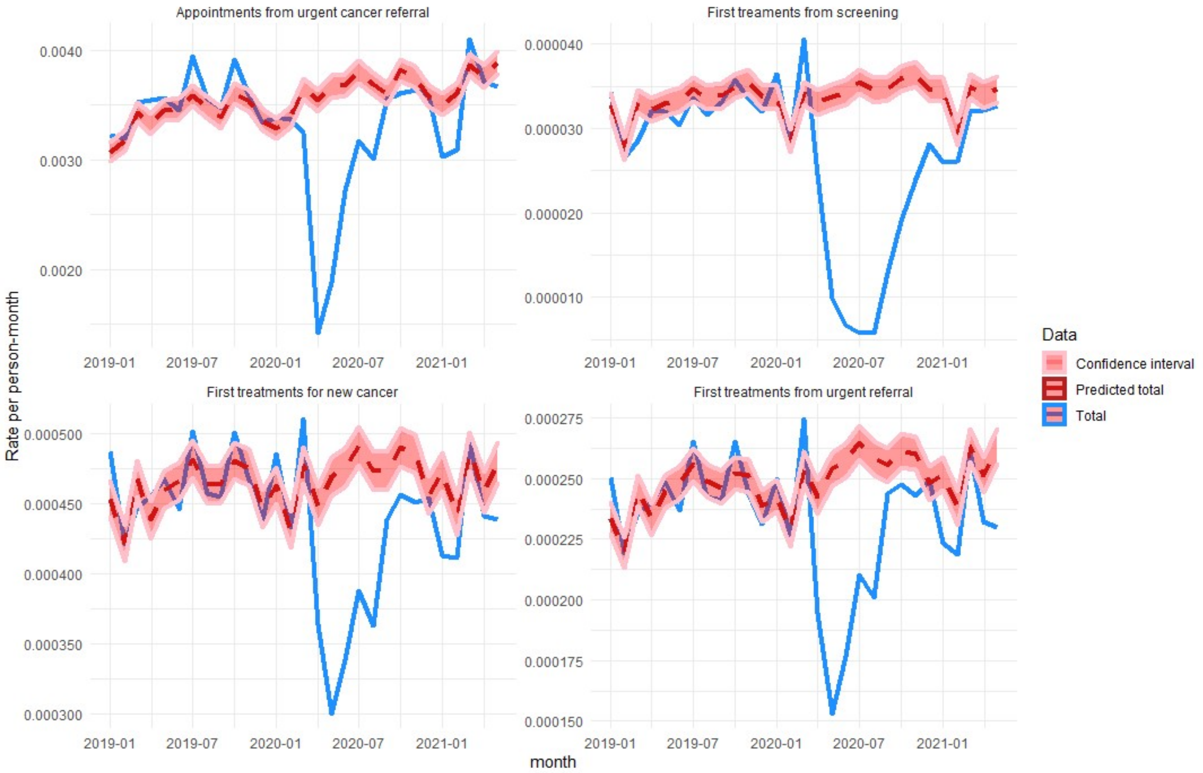

Annex 7: Comparing Cancer Wait Times counts for referrals and first treatments with the NCRAS data equity pack

CADEAS and NCRAS have produced two equity data packs presenting the latest national data on the number of urgent suspected two-week wait referrals and first definitive treatments for cancer. These data packs are produced on the basis of the Cancer Wait Times data, with analysis from Hospital Episode Statistics (HES) and other sources outlined in their technical notes.

“Any differences between treatment volumes in the published official statistics and the volumes presented in this pack are because:

- Data was extracted from the CWT system at a slightly different time.
- Data included here is based on England residents only.

Additional logic has been applied to remove treatments where some of the information required for this equity analysis is missing or there are potential data quality issues, for example cases with a mismatch between the suspected cancer referral type and sex (eg. gynaecological cancer treatments for men, testicular cancer treatments for women), and suspected cancer referral type and age (eg. suspected children's cancer for patients aged 20 and over)." – NCRAS Cancer data equity pack technical notes. Available: http://www.ncin.org.uk/local_cancer_intelligence/cadeas

Table A7.1: Comparing Cancer Wait Times counts for referrals and first treatments with the NCRAS data equity pack

	01 Apr 2020 - 31 Jan 2021					
	First treatments for new cancer			Urgent cancer referrals		
	Observed	Expected	Percentage change (95% CI)	Observed	Expected	Percentage change (95% CI)
Cancer wait times data						
All	224,323	267,946	-16.3% (-16.6, -15.9)	1,673,775	2,071,967	-19.2% (-19.3, -19.1)
NCRAS data equity pack						
All	219,410	254,436	-13.8% (-14.3, -13.3)	1,658,309	1,984,489	-16.4% (-16.6, -16.3)
Breast	30,488	40,530	-24.8% (-25.9, -23.6)	337,582	363,139	-7% (-7.5, -6.6)
Gynaecological	11,281	12,344	-8.6% (-10.9, -6.2)	158,723	176,985	-10.3% (-10.9, -9.7)
Head and Neck	8,892	9,901	-10.2% (-12.7, -7.6)	163,668	189,837	-13.8% (-14.4, -13.2)
Lower GI	23,507	27,056	-13.1% (-14.6, -11.6)	302,369	366,677	-17.5% (-17.9, -17.1)
Lung	24,796	27,409	-9.5% (-11.1, -8.0)	33,830	53,641	-36.9% (-37.8, -36.1)
Skin	40,977	43,475	-5.7% (-7.0, -4.5)	338,172	429,802	-21.3% (-21.7, -21.0)
Upper GI	17,059	17,586	-3% (-5.0, -0.9)	141,720	163,013	-13.1% (-13.7, -12.4)
Urological	37,970	50,056	-24.1% (-25.2, -23.1)	134,389	184,642	-27.2% (-27.7, -26.7)
All other	24,441	26,080	-6.3% (-7.9, -4.6)	47,856	56,753	-15.7% (-16.7, -14.6)

Annex 8: Observed vs expected consultations by IMD quintile

Figure A8: Observed vs expected consultations per person per week by IMD quintile, age-standardised (01 Jan 2019 – 30 Jan 2021)

For peer review only

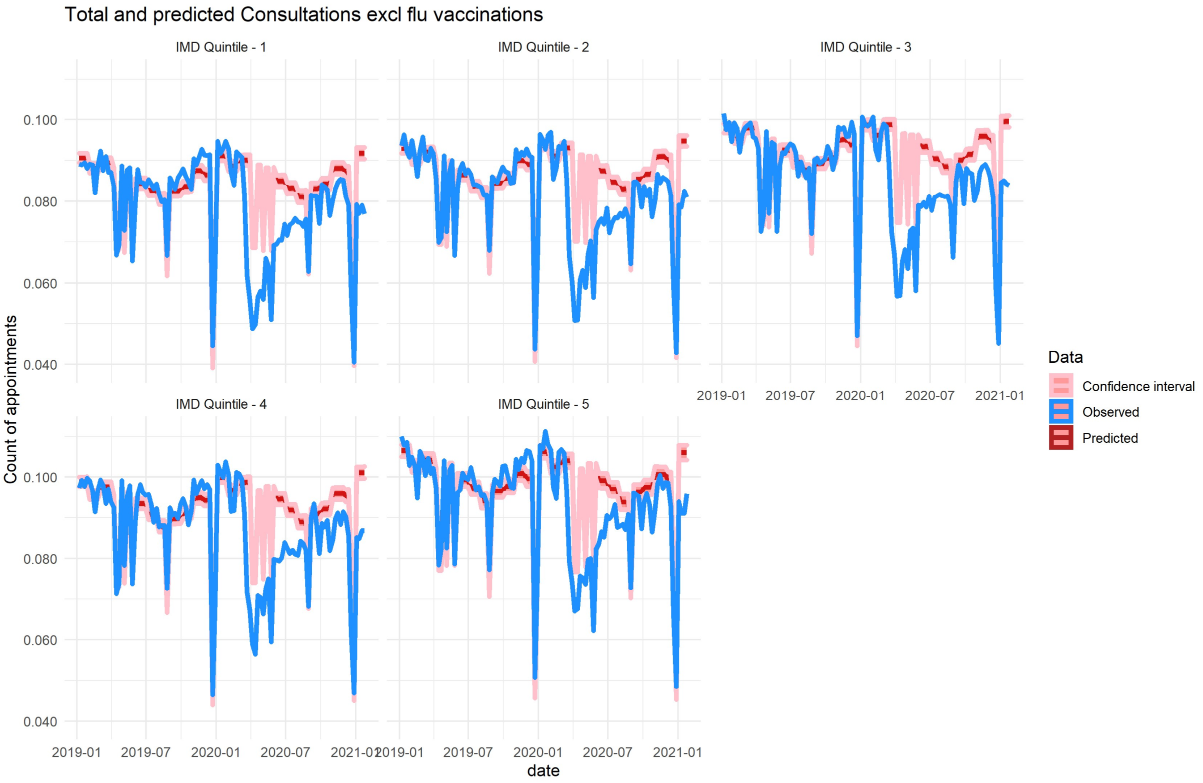

For peer review only

STROBE Statement—checklist of items that should be included in reports of observational studies

	Item No	Recommendation	Page No
**Title and abstract**	1	(*a*) Indicate the study's design with a commonly used term in the title or the abstract	2
		(*b*) Provide in the abstract an informative and balanced summary of what was done and what was found	2
**Introduction**			
Background/rationale	2	Explain the scientific background and rationale for the investigation being reported	3
Objectives	3	State specific objectives, including any prespecified hypotheses	3
**Methods**			
Study design	4	Present key elements of study design early in the paper	4
Setting	5	Describe the setting, locations, and relevant dates, including periods of recruitment, exposure, follow-up, and data collection	4
Participants	6	(*a*) *Cohort study*—Give the eligibility criteria, and the sources and methods of selection of participants. Describe methods of follow-up<br>*Case-control study*—Give the eligibility criteria, and the sources and methods of case ascertainment and control selection. Give the rationale for the choice of cases and controls<br>*Cross-sectional study*—Give the eligibility criteria, and the sources and methods of selection of participants	4
		(*b*) *Cohort study*—For matched studies, give matching criteria and number of exposed and unexposed<br>*Case-control study*—For matched studies, give matching criteria and the number of controls per case	
Variables	7	Clearly define all outcomes, exposures, predictors, potential confounders, and effect modifiers. Give diagnostic criteria, if applicable	4/5
Data sources/ measurement	8*	For each variable of interest, give sources of data and details of methods of assessment (measurement). Describe comparability of assessment methods if there is more than one group	*4/5*
Bias	9	Describe any efforts to address potential sources of bias	5/6
Study size	10	Explain how the study size was arrived at	4
Quantitative variables	11	Explain how quantitative variables were handled in the analyses. If applicable, describe which groupings were chosen and why	4-6
Statistical methods	12	(*a*) Describe all statistical methods, including those used to control for confounding	5/6
		(*b*) Describe any methods used to examine subgroups and interactions	5/6
		(*c*) Explain how missing data were addressed	4-6
		(*d*) *Cohort study*—If applicable, explain how loss to follow-up was addressed<br>*Case-control study*—If applicable, explain how matching of cases and controls was addressed<br>*Cross-sectional study*—If applicable, describe analytical methods taking account of sampling strategy	N/A
		(*e*) Describe any sensitivity analyses	4-6

Continued on next page

For peer review only - http://bmjopen.bmj.com/site/about/guidelines.xhtml

**Results**			
Participants	13*	(a) Report numbers of individuals at each stage of study—eg numbers potentially eligible, examined for eligibility, confirmed eligible, included in the study, completing follow-up, and analysed	4/5
		(b) Give reasons for non-participation at each stage	N/A
		(c) Consider use of a flow diagram	N/A
Descriptive data	14*	(a) Give characteristics of study participants (eg demographic, clinical, social) and information on exposures and potential confounders	6-8
		(b) Indicate number of participants with missing data for each variable of interest	6-8
		(c) *Cohort study*—Summarise follow-up time (eg, average and total amount)	N/A
Outcome data	15*	*Cohort study*—Report numbers of outcome events or summary measures over time	*6/7*
		Case-control study—Report numbers in each exposure category, or summary measures of exposure	*N/A*
		Cross-sectional study—Report numbers of outcome events or summary measures	*N/A*
Main results	16	(*a*) Give unadjusted estimates and, if applicable, confounder-adjusted estimates and their precision (eg, 95% confidence interval). Make clear which confounders were adjusted for and why they were included	6-8
		(*b*) Report category boundaries when continuous variables were categorized	N/A
		(*c*) If relevant, consider translating estimates of relative risk into absolute risk for a meaningful time period	N/A
Other analyses	17	Report other analyses done—eg analyses of subgroups and interactions, and sensitivity analyses	6-8
**Discussion**			
Key results	18	Summarise key results with reference to study objectives	9
Limitations	19	Discuss limitations of the study, taking into account sources of potential bias or imprecision. Discuss both direction and magnitude of any potential bias	9/10
Interpretation	20	Give a cautious overall interpretation of results considering objectives, limitations, multiplicity of analyses, results from similar studies, and other relevant evidence	10
Generalisability	21	Discuss the generalisability (external validity) of the study results	9/10
**Other information**			
Funding	22	Give the source of funding and the role of the funders for the present study and, if applicable, for the original study on which the present article is based	1

*Give information separately for cases and controls in case-control studies and, if applicable, for exposed and unexposed groups in cohort and cross-sectional studies.

Note: An Explanation and Elaboration article discusses each checklist item and gives methodological background and published examples of transparent reporting. The STROBE checklist is best used in conjunction with this article (freely available on the Web sites of PLoS Medicine at http://www.plosmedicine.org/, Annals of Internal Medicine at http://www.annals.org/, and Epidemiology at http://www.epidem.com/). Information on the STROBE Initiative is available at www.strobe-statement.org.

For peer review only

BMJ Open

Primary Care and Cancer: an analysis of the impact and inequalities of the COVID-19 pandemic on patient pathways

Journal:	*BMJ Open*
Manuscript ID	bmjopen-2021-059374.R1
Article Type:	Original research
Date Submitted by the Author:	26-Jan-2022
Complete List of Authors:	Watt, Toby; The Health Foundation, REAL Centre<br>Sullivan, Richard; King's College London<br>Aggarwal, Ajay; London School of Hygiene and Tropical Medicine Faculty of Public Health and Policy, Health Services Research & Policy
**Primary Subject Heading**:	Oncology
Secondary Subject Heading:	General practice / Family practice
Keywords:	ONCOLOGY, PRIMARY CARE, COVID-19

SCHOLARONE™
Manuscripts

I, the Submitting Author has the right to grant and does grant on behalf of all authors of the Work (as defined in the below author licence), an exclusive licence and/or a non-exclusive licence for contributions from authors who are: i) UK Crown employees; ii) where BMJ has agreed a CC-BY licence shall apply, and/or iii) in accordance with the terms applicable for US Federal Government officers or employees acting as part of their official duties; on a worldwide, perpetual, irrevocable, royalty-free basis to BMJ Publishing Group Ltd ("BMJ") its licensees and where the relevant Journal is co-owned by BMJ to the co-owners of the Journal, to publish the Work in this journal and any other BMJ products and to exploit all rights, as set out in our licence.

The Submitting Author accepts and understands that any supply made under these terms is made by BMJ to the Submitting Author unless you are acting as an employee on behalf of your employer or a postgraduate student of an affiliated institution which is paying any applicable article publishing charge ("APC") for Open Access articles. Where the Submitting Author wishes to make the Work available on an Open Access basis (and intends to pay the relevant APC), the terms of reuse of such Open Access shall be governed by a Creative Commons licence – details of these licences and which Creative Commons licence will apply to this Work are set out in our licence referred to above.

Other than as permitted in any relevant BMJ Author's Self Archiving Policies, I confirm this Work has not been accepted for publication elsewhere, is not being considered for publication elsewhere and does not duplicate material already published. I confirm all authors consent to publication of this Work and authorise the granting of this licence.

For peer review only

Primary Care and Cancer: an analysis of the impact and inequalities of the COVID-19 pandemic on patient pathways

Authors: Toby Watt[1,2], Richard Sullivan[3] and Ajay Aggarwal[4]

1. The Health Foundation, 8 Salisbury Sq., London, UK,
2. Department of Public Health, Environments and Society, London School of Hygiene and Tropical Medicine, 15-17 Tavistock Place, London, UK
3, School of Cancer and Pharmaceutical Sciences, King's College London, London, UK; Institute of Cancer Policy, King's College London, London, UK; Department of Oncology, Guy's and St Thomas' NHS Foundation Trust, London, UK.
4. Department of Health Services Research and Policy, London School of Hygiene & Tropical Medicine, London, UK; Institute of Cancer Policy, King's College London, London, UK; Department of Oncology, Guy's and St Thomas' NHS Foundation Trust, London, UK.

Corresponding author: Toby Watt, toby.watt@health.org.uk

Word count: 4,584

For peer review only

Abstract

Objectives

We explore the routes to cancer diagnosis to further undertanding of the inequality in the reduction in detection of new cancers since the start of the pandemic. We use different data sets to assess stages in the cancer pathway: primary care data for primary care consultations, routine and urgent referrals and published analysis of cancer registry data for appointments and first treatments.

Setting

Primary and cancer care

Participants

In this study we combine multiple data sets to perform a population-based cohort study on different areas of the cancer pathway. For primary care analysis, we use a random sample of 500,000 patients from the Clinical Practice Research Datalink (CPRD). Post-referral we perform a secondary data analysis on the Cancer Wait Times data and the National Cancer Registry Analysis Service (NCRAS) COVID-19 data equity pack.

Outcome measures:

Primary care: consultation, urgent cancer referral and routine referral rates, then appointments following an urgent cancer referral, and first treatments for new cancer, for all and by quintile of patient's local area Index of Multiple Deprivation.

Results

Primary care contacts and urgent cancer referrals fell by 12.4% (12.3 to 12.6; 95% CI) and 20.2% (18.1 to 22.3; 95% CI) respectively, while routine referrals have not recovered to pre-pandemic levels. Reductions in first treatments for newly diagnosed cancers are down 16.3% (15.9 to 16.6; 95% CI). The reduction in the number of two week wait referrals and first treatments for all cancer has been largest for those living in poorer areas, despite having a smaller reduction in primary care contact.

Conclusions

Our results further evidence the strain on primary care and the presence of the inverse care law, and the dire need to address the inequalities so sharply brought into focus by the pandemic. We need to address the disconnect between the importance we place on the role of primary care and the resources we devote to it.

Key Words: Oncology, Primary Care, COVID-19

Strengths and Limitations

- This study draws from multiple data sets along the complex, multidisciplinary cancer pathway.
- We use a rich primary care data set containing patient level primary care activity linked to patients' local area socioeconomic indicator.
- Our primary care patient sample is relatively small (500,000 active patients from January 2016 to January 2021), however the data produces results that closely mirror the rates of consultation and urgent cancer referral per patient produced in publicly available national data sets.

For peer review only

INTRODUCTION

The COVID-19 pandemic has had a profound impact on UK's health system. Each part of the UK's National Health Service has been impacted in different ways, and we are still feeling many of the consequences of both the COVID-19 pandemic and the public health measures put in place to manage it (non-pharmaceutical interventions; NPI). Cancer is one of the most complicated diseases that the UK health system must manage, being responsible for over one in four UK deaths in 2019. Cancer outcomes are acutely sensitive to changes in social determinants, patient pathways and service provision. Delays in both diagnosis and treatment have significant impacts on patient outcomes(1,2). Pandemic related diagnostic delays, lack of capacity and downstream stage progression (to more advanced disease) are already being seen(3). In addition, the impact of the pandemic needs to be seen in the context of an already overstretched UK cancer care system pre-pandemic that was 'burning hot' even in normal times(4).

Primary care sits at the heart of the cancer patient pathway and is the most crucial interface for early diagnosis and referral to hospital-based care, in addition to their wider support of cancer patient undergoing and post treatment. As models of cancer care have involved in light of both technical advances and an ageing co-morbid population primary care has become an increasingly important aspect of integrated cancer care and an expansion of General Practitioner (GP) roles in cancer care(5). On average 22.5% of patients diagnosed with cancer are referred to oncology diagnostic services from primary care, but this reflects wide site-specific variation from as little as 8.3% of breast cancer to 42% for bladder cancer(6).

It is important to reflect that prior to the start of the COVID-19 pandemic primary care had seen significant declines in overall resourcing relative to the funding of the rest of the NHS and compared to growing levels of disease burden that is managed in primary care. In addition, there is growing evidence that primary care has been under greater pressure in more deprived areas, with higher levels of staff turnover(7), higher levels of complex multimorbidity(8), higher numbers of consultations(9) and lower levels of funding and fewer GPs per capita once levels of ill health are taken into account(10). These pressures on primary care, and a desire to correct them, have been recognised in the NHS Long Term Plan(11).

Thus, to understand the COVID-19's impact on primary care and the downstream impact on cancer outcomes we need to see that the pandemic arrived when the system that was already struggling to cope. Prior to COVID-19, the central role of primary care as agents of change in reducing inequalities had been the subject of much debate yet could do little in the face of political avoidance of health equity(12). Primary care had become a mirror on inequalities but also subject to significant pressures from these growing inequalities that had put practices in deprived populations under significant stress. Yet despite this, equity-oriented primary care reform in England in the mid-to-late 2000s may have helped to reduce socioeconomic inequality in health(13).

[Box]

COVID-19 was officially declared a pandemic by the WHO on 11 March 2020, and the UK announced its first full lockdown on 23rd March. In the following months UK NPI were eased, schools reopened in phases, non-essential shops reopened and in August the population were encouraged to eat out. Some restrictions were re-imposed in September and October, on the 5th of November 2020 a second brief national locked lasted until 2nd December. On the 6th of January a third national lockdown was introduced(14).

[End box]

It is now clear that the UK experience of the pandemic was one of the worst in the world, both in terms of excess mortality (both COVID-19 and non-COVID-19) and the impact of NPI (lockdowns) on both the ability of health services to continue provide care and the impact of messaging (stay at home) on patients' timely presentation for care(15). However, the overwhelming focus of impact studies on cancer care has been on hospital-based services, including diagnostics. Given primary care's central role in pathways to diagnosis and integrated cancer care, including survivorship there has been little insight around how overall changes in consultation rates impacted both routine and two week wait referrals as well as how this varied both in terms of site-specific cancers and as a consequence of socio-economic

For peer review only

inequalities. This study aimed to analyse the socioeconomic inequalities in the impact of NPI measures taken in response to COVID-19 on consultations and routine and urgent cancer referrals in primary care and cancer diagnosis in secondary care.

METHODS

Study design, data sources and participants

We perform a population-based cohort study using the following three separate sources.

Primary care data – CPRD Aurum

Primary care electronic health records were obtained from the Clinical Practice Research Datalink Aurum database (henceforth CPRD). We included patient records from 01 January 2016 to 31 January 2021. Pre-pandemic data were included to establish long-term trends and patterns of seasonality in primary care use and referrals to secondary care. Similar to recent analysis of the COVID-19 pandemic(16), our analysis focusses on comparing observed levels of activity to the expected following the introduction of NPI in England in March 2020.

CPRD contains anonymised patient primary care data from approximately 7% of the UK population and is broadly representative in terms of age, sex, and ethnicity(17). The patient records include information on consultations, patient demographic information, diagnoses, medication prescriptions, and referrals to secondary care.

The period of eligibility for study inclusion, which starts on the latest of the study start date (01/01/2016) or the patient's registration to their practice. A patient's period of eligibility ends on the earliest of leaving their practice, the end of data collection from their practice or their death. Primary care records from CPRD were linked to the deciled index of multiple deprivation (IMD) from 2015[i](18) of each patient's lower layer super output area (LSOA[ii]). 500,000 patients were randomly sampled from the CPRD population in England who were eligible for linkage within the defined study period.

Cancer wait times data

Cancer waiting times (CWT) measure performance against the NHS Constitution Standards, recording the number of patients screened, referred to oncology specialists, diagnosed and treated for cancer. These measures are used by local and national organisations to monitor the timely delivery of services to patients, they are published quarterly by NHS Digital[iii].

Cancer diagnosis by socioeconomic status - NCRAS Cancer equity data

Data on cancer diagnosis by socioeconomic group was drawn from CADEAS and National Cancer Registry Analysis Service (NCRAS) which have two published data sets(19), presenting the latest national data on:

i. The number of urgent suspected two-week wait referrals[iv] and,
ii. First definitive treatments for cancer[v].

---

[i] https://www.gov.uk/government/statistics/english-indices-of-deprivation-2015
[ii] Geographic areas in England and Wales that are built from groups of contiguous Output Areas and have been automatically generated to be as consistent in population size as possible, and typically contain from four to six Output Areas. The Minimum population is 1000 and the mean is 1500. For more details visit: https://datadictionary.nhs.uk/nhs_business_definitions/lower_layer_super_output_area.html#:~:text=Lower%20Layer%20Super%20Output%20Areas,statistics%20in%20England%20and%20Wales.
[iii] https://www.england.nhs.uk/statistics/statistical-work-areas/cancer-waiting-times/
[iv] http://www.ncin.org.uk/view?rid=4346 (accessed on 24 January 2022)
[v] http://www.ncin.org.uk/view?rid=4347 (accessed on 24 January 2022)

For peer review only - http://bmjopen.bmj.com/site/about/guidelines.xhtml

These data packs are produced based on the CWT data, with analysis from Hospital Episode Statistics (HES) and other sources outlined in their technical notes (further details in Annex 1).

Study Outcomes

Primary care consultations

We define consultations in CPRD data by a set of rules developed based on two variables in the consultations file[vi] ("EMIS® consultation source identifier" and "Consultation source code identifier")[vii]. In line with the approach taken by Carey et al 2012 for CPRD Gold data, we use a combination of the consultation code and the category of the record to identify consultations (details in Annex 2).

Using the observation file in CPRD Aurum, we were also able to identify where patients had influenza vaccinations. We look to exclude flu vaccines from our analysis on the basis that the programme was expanded in 2020/21 to achieve maximum uptake[viii]. To help with the comparability of consultations in the two periods, we removed primary care appointments that included a flu vaccine.

Referrals from primary care: routine and urgent cancer

Referrals in CPRD are categorized into routine and "urgent cancer". Referrals from the 'referral file' are linked to patients, no additional data cleaning steps were taken in the analysis of referrals.

First appointment following an urgent referral

The CWT data present monthly counts of patients in England who have been recorded as receiving a first appointment following an urgent referral from primary care. The CWT data record this because the NHS have a 2-week performance target (Annex 3).

The NCRAS cancer equity data contain monthly counts in England of appointments following an urgent cancer referral broken down by tumour type and by deprivation according to patient's place of residence.

First treatment following a cancer diagnosis

The CWT data present monthly counts of patients in England who have been recorded as receiving a first treatment for a new cancer diagnosis. The CWT data record this because the NHS have a 31-day performance target (Annex 3).

The NCRAS cancer equity data contain monthly counts in England of first treatments for new cancer broken down by tumour type and by deprivation according to patient's place of residence.

Patient and public involvement

No patients involved

Data analysis

CPRD & CWT

For both CPRD and CWT we separate the data into two, before and after the introduction of the first NPI.

[vi] https://cprd.com/primary-care

[vii] These variables contain strings that categorise the patient record input and are selected by the staff member completing the record.

[viii] https://www.england.nhs.uk/wp-content/uploads/2020/05/Letter_AnnualFlu_2020-21_20200805.pdf

For peer review only

Our analysis of CPRD primary care is conducted weekly and split into two periods before and after the introduction of NPI on March 23rd 2020 (pre-NPI data is from 03 January 2016 to 21 March 2020, our post-NPI onset data is 22 March 2020 to 30 January 2021).

CWT data is reported monthly, our pre-NPI data is therefore from 01 October 2009 to 31 March 2020, our post-NPI onset period is 01 April 2020 to 31 January 2021.

We perform a linear regression of consultations, urgent and routine referrals from CPRD data and appointments following an urgent cancer referral and first treatments from CWT data over time to estimate expected values for the post-NPI onset period, based on predicted values from the data pre-NPI. To account for seasonality and time trends we include months as a categorical variable and time as a continuous variable, the approach taken by Carr et al.(16). In the case of weekly primary care data, we observe large dips in activity in weeks that include bank holidays and include a categorical variable on the basis of the number of bank holidays in each week (in the winter holidays in England there is always one week with two bank holidays). Our primary care activity rates are presented per 100,000 patient-months[ix]. When analysing primary care consultation rates by socioeconomics we adjust for population age. We do so when calculating the consultation rates by IMD quintile and weighting the sample according to the European Standard Population[x] (ESP).

NCRAS equity data

The analysis presented in the equity data pack compares new instances of first treatments in months during the pandemic (01 April 2020 – 31 Jan 2021) compared with the same months in 2019/20. The analysis includes a 95% confidence interval for the changes, based on rate ratios under an assumption that the population is the same in the pre-COVID-19 baseline and COVID-19 months. This is calculated using the exact method described in Breslow & Day 1987, pp 93-95(20). The NCRAS equity data pack shows the high levels of heterogeneity in the impact of the COVID-19 pandemic on different tumour locations. The NCRAS data equity pack is different in its count and analysis of "all tumours" compared with the Cancer Wait Times Data, this is because the data are slightly different (cleaned and analysed by NCRAS – details in Annex 1). Results of our analysis with each data set are compared in Annex 4. Our presentation of these data follows the same method but presents the cumulative difference for the period from April 2020 to the end of January compared with the previous 12 months.

RESULTS

Overall impact of the pandemic

In the calendar year of 2019, before the COVID-19 pandemic and the associated NPI, there was an average of 39,127 primary care consultations per 100,000 patient-months. This equates to 4.70 attended appointments per registered patient, or an estimated 266 million appointments in primary care nationally in 2019[xi].

Primary care consultations (Figure 1 a) dropped rapidly to a low of 26,919consultations per 100,000 patient-months in the week following 29 March 2020, this was 66.0% lower than the predicted rate. Rates slowly recovered over the next 24 weeks and by 05 September 2020 were up to 99% of the baseline. In total there were an estimated 19.7 million (19.5 to 20.0; 95% CI) fewer primary care consultations in the English NHS during this period. Primary care consultations again fell to below 90% of predicted levels in the third wave NPI starting on 06 January 2021, by the end of January 2021 there were a further 6.4 million fewer consultations than expected. Between the start of the first NPI in

---

[ix] We adjust the weekly rates per active patient in our sample to 100,000 patient-months: Weekly rate per registered patient in sample x 100,000 x (52/12)

[x] https://www.causesofdeath.org/docs/standard.pdf

[xi] For comparison the NHS national appointments data recorded 272 million attended appointments in primary care in 2019. Found here: https://digital.nhs.uk/data-and-information/publications/statistical/appointments-in-general-practice/march-2021

For peer review only - http://bmjopen.bmj.com/site/about/guidelines.xhtml

March 2020 and the end of January 2021 there were an estimated 26.1 million (25.7 to 26.5; 95% CI) fewer appointments than expected (Table 1a).

In 2019, the average rate of urgent cancer (two week wait) referral was 314 per 100,000 patient-months, equating to an estimated 2.12 million for the NHS in England. Following the first NPI, urgent cancer referrals from primary care (Figure 1 b) fell to a nadir of 86 per 100,000 patient-months by 29 March 2020 (29.7% of the predicted level). Urgent cancer referrals did not return to pre-pandemic baseline until the week following 23 August 2020 equating to 317,000 (280,000 to 356,000; 95% CI) estimated lost urgent cancer referrals over this period. There was a second fall in urgent cancer referrals from primary care in the winter to below 90% of the baseline following the third lockdown (164referrals per 100,000 patient-months in the week beginning 27 December 2021). This resulted in a further estimated 91,705 fewer urgent cancer referrals than expected. Between the start of the first NPI in March 2020 and the end of January 2021 there were 395,000 (344,000 to 446,000; 95% CI) fewer urgent cancer referrals than expected (Table 1a).

Routine referrals however have shown a different trajectory in that their rates did not recover to pre-pandemic levels (Figure 1 c). As a share of predicted levels routine referrals had the greatest fall, dropping to 16.1% of predicted rates in the week from 19 April 2020. From then to the end of January the closest it came to predicted levels was 80.3% in the week flowing 13 September 2020. For the four weeks in January 2021, it had fallen back down to 60-70% of predicted rates. In 2019 there were an average of 1,801 routine referrals per 100,000 patient-months from primary care, equivalent to an estimated 12.2 million for the NHS in England. Between the start of the first NPI in March 2020 and the end of January 2021 there were 4.33 million (4.21 to 4.46; 95% CI) fewer routine referrals than expected (Table 1a).

Patient demographics and patient-time and total numbers of observed consultations and routine and urgent referrals in our CPRD sample are presented in Annex 5.

Table 1a: Observed post COVID-19 primary care activity (CPRD Aurum) 22 March 2020 – 30 January 2021

	Observed rate	Expected rate	Percentage reduction (95% CI)	Estimated # missing from England population, to 3 significant digits (95% CI)
Event rate per 100,000 patient-months				
Consultations	34,201	38,684	11.6% (11.4, 11.7)	26,100,000 (25,700,000, 26,500,000)
Routine Referrals	1,067	1,812	41.1% (40.4, 41.8)	4,330,000 (4,210,000, 4,460,000)
2 Week Wait Referrals	268	336	20.2% (18.1, 22.3)	395,000 (344,000, 446,000)

Table 1b: Observed post COVID-19 cancer diagnostic activity (Cancer Wait Times), 01 April 2020 - 31 January 2021

	Observed rate	Expected rate	Percentage reduction (95% CI)	Estimated # missing from England population, to 3 significant digits (95% CI)
Event rate per 100,000 patient-months				
First consultant appointments following urgent referral from primary care	296	366	19.2% (19.1, 19.3)	398,000 (395,000, 401,000)
Incidence rate per 100,000 patient-months				
First treatments for new cancer from the urgent primary care referral pathway	21.4	25.5	16.1% (15.5, 16.8)	23,300 (22,200, 24,400)
First treatments for new cancer from the national screening pathway	1.63	3.47	53.2% (52, 54.3)	10,400 (10,000, 10,900)
First treatments for new cancer	39.7	47.4	16.3% (15.9, 16.6)	43,600 (42,500, 44,700)

For peer review only

Table 1a summarises the missing appointments and referrals for the post-pandemic period. Since the start of the pandemic in March we have observed consultations rates that are 11.6% (11.4 to 11.7; 95% CI) lower than predicted by previous data. The number of referrals to secondary care per consultation has also fallen, with urgent cancer referrals 20.2% (18.1 to 22.3; 95% CI) and routine referrals 41.1% (40.4 to 41.8; 95% CI) lower than expected.

The knock-on effect of the reductions in patients' primary care appointments and referrals can be observed in the national CWT data. The number of first appointments with a cancer specialist following an urgent cancer referral has fallen by approximately the same amount as estimated for the referrals themselves: 19.2% (19.1 to 19.3; 95% CI). The number of cancer first treatments (following a diagnosis and decision to treat) was 16.3% (15.9 to 16.6; 95% CI) lower than expected, or 43,600 (42,500 to 44,700; 95% CI) missing first treatments from 01 April 2020 - 31 January 2021[xii] (graphs of observed compared with expected are presented in Annex 6).

Urgent cancer referrals by site specific cancer from 01 April 2020 until 31 January 2021 showed significant heterogeneity from moderate reductions in urgent referrals for suspected breast (7.0%; 95% CI 6.6 to 7.5) and gynaecological cancers (10.3%; 95% CI 9.7 to 10.9) and greater reductions for lung (36.9%; 95% CI36.1 to 37.8) and urological (27.2%; 95% CI 26.7 to 27.7) cancers (Figure 2, further details in Annex 4, Table A4.1). To show how pathway delays interface with reductions in cancer referrals we also examined reductions in first treatments for the same site-specific cancers over this period (Figure 2). Breast and urological cancers observed the greatest reduction in new first treatments: Breast fell by 24.8% (23.6 to 25.9; 95% CI) which equates to 10,000 missing treatments and urological by 24.1% (23.2 to 25.2; 95% CI) which equates to 12,100 missing treatments. Taken together these data reflect substantial delays in both diagnostic and treatment phases of the patient pathway.

Inequalities in cancer diagnosis outcomes in the pandemic

There are inequalities in primary care use in England, with the people who live in the poorest areas have higher rates of consultation than those in richer areas once we adjust for age. The most deprived quintile was expected to have 43,184 consultations per 100,000 patient-months (Table 2), 15% more than the least deprived.

Table 2: Observed post COVID-19 primary care activity (CPRD Aurum) by IMD quintile, actual and age-standardised

	22 Mar 2020 - 30 Jan 2021 (Weekly)		
	Observed rate	Expected rate	Percentage reduction (95% CI)
Consultations per 100,000 patient-months			
IMD Quintile - 1 (least deprived)	33,813	38,601	12.4% (12.1, 12.7)
IMD Quintile - 2	34,169	38,793	11.9% (11.6, 12.3)
IMD Quintile - 3	35,069	40,127	12.6% (12.3, 12.9)
IMD Quintile - 4	33,494	37,793	11.4% (11, 11.7)
IMD Quintile - 5 (most deprived)	34,561	38,212	9.6% (9.2, 9.9)
Consultations per 100,000 patient-months (Age-standardised*)			
IMD Quintile - 1 (least deprived)	32,927	37,636	12.5% (12.2, 12.8)
IMD Quintile - 2	33,916	38,647	12.2% (11.9, 12.6)
IMD Quintile - 3	35,535	40,870	13.1% (12.7, 13.4)
IMD Quintile - 4	36,271	41,148	11.9% (11.5, 12.2)
IMD Quintile - 5 (most deprived)	38,997	43,184	9.7% (9.4, 10)

**Age-standardisation is performed according to the European Standard Population (ESP)*

The reduction of consultations over the period 22 March 2020-30 January 2021 was smallest for those in most deprived areas. Their reduction in consultations for the non-age-standardised figures was 9.6% (9.2 to 9.9), while for the least

[xii] Dates for the CWT and NCRAS analysis do not line up with the CPRD analysis because the latter is conducted weekly, not monthly.

For peer review only

deprived the reduction was 12.4% (13.2 to 13.9; 95% CI) (Table 2). Weekly levels of age-standardised consultations per 100,000 patient-months by IMD quintile are presented in Annex 7.

Despite a smaller reduction in primary care contacts, we observe the largest reduction in both urgent cancer referrals and first treatments for cancer for patients living in the most deprived areas. The NCRAS data equity pack presents the number of urgent cancer referrals and first cancer treatments by IMD quintile[xiii]. Figure 3 shows the reduction in urgent cancer referrals and first treatments for newly diagnosed cancer by IMD quintile.

There was a greater percentage reduction in urgent cancer referrals for those living in the most deprived areas in England, who experienced a 17.6% (17.2 to 18.0; 95% CI) reduction between 01 April 2020 and 31 January 2021 compared with the same period 12 months before, while referrals for the least deprived quintile fell by proportionately less: 15.3% (14.9 to 15.6; 95% CI). This equates to a reduction of 61,500 referrals for the most deprived and 62,600 or the least: without adjusting for age, the most deprive quintile had a smaller proportion of the pre-pandemic urgent cancer referrals, with 350,000 referrals compared to 410,000 for the least deprived quintile from April 2019 to January 2020.

At the same time, rates of new treatment for cancer for the people living in the most deprived 20% of England experienced a 15.8% (14.6 to 17.0; 95% CI) reduction between 01 April 2020 and 31 January 2021 compared with the same period 12 months before (6,610 missing first treatments). The reduction for the least deprived was 12.6% (11.5 to 13.7; 95% CI) which equates to 6,880 missing first treatments.

Despite having more access to primary care for patients in more deprived areas (9.7% reduction for most deprived compared to 12.5% for the least deprived), urgent cancer referrals and newly diagnosed cancers have been disrupted by the pandemic more for people living in poorer areas.

DISCUSSION

The coronavirus SARS-CoV-2 (COVID-19) pandemic has had a profound impact on the management of patients with cancer(21). The first national lockdown in March 2020 created a ripple of non-pharmaceutical interventions, including 'stay at home' orders, diminished healthcare service provision and redistribution of healthcare to COVID-19 related care that has had a profound impact on cancer services(1,22).

There are also new potential barriers to the pathway that have resulted and may exacerbate these findings. For example, decreases in health seeking behaviour due to fear of acquiring covid-19 infection through interactions with health care settings, increasing use of remote consultations(23), changes in routine referral guidelines(24), as well as changes in the capacity of acute care. The backlog for routine diagnostic services is a particular concern given that approximately 40% of cancer are diagnosed through this route(25).

Similar issues have also been identified within other high-income country health systems. Primary care providers in eight European countries experienced similar issues in how to rapidly transform services in the wake to COVID-19(26). A study in Sweden found an almost identical percentage reduction in primary care consultations (12%) as a results of the pandemic(27). Our results don't appear to be unique to England: while different countries can have different routes to diagnosis(28), different countries with different systems also observed disruptions to cancer pathways(29–32).

Whilst it was already known that there had been a substantial reduction in the number of overall cancer-related referrals(30,31) the quantification of this had been missing. Our findings, that primary care consultations in English NHS fell by 12.4% between January 2020 and January 2021 with urgent cancer referrals even more suppressed (20.2%), reflect how profound the pathway disruptions were for cancer patients. Furthermore, many cancers are picked up through the course of routine referrals from general practice for non-specific symptoms. The drop in routine referrals that we found (4.3 million, over this period) will inevitably translate into late-stage presentation and a substantial

[xiii] They do not age-standardise their results

For peer review only

reduction in outcomes. This will include wider economic costs due to more expensive, late-stage treatment and productivity losses due to morbidity and premature mortality. However, the trajectory of the declines reflect not just changes to national policy in terms of NPI, but also knock-on effects around public behaviour, primary care staffing, downstream reductions in diagnostics and an overall increase in friction across all cancer pathways and systems.

This reduction in cancer pathways through primary care needs to be put in the context of wider disruptions. The suspension of national cancer screening programmes meant that around 2 million people were not screened for cancer through national programs(32,33). Moreover, delays in cancer diagnoses and treatments have consistently been associated with poorer outcomes(1,2). The COVID-19 pandemic has also exacerbated the worst 62-day cancer waiting time targets in the last decade where 1 of 4 patients urgently referred from primary care between April 2020 and January 2021 did not receive treatment within 62 days(31).

In our analysis of urgent cancer referrals by site in relation to reductions seen in first treatments significant differences were seen. Urological cancers (testis, renal, prostate and urothelial) have been particularly impacted with greater than 25% decrease both in urgent referrals and first treatments. This suggests that outcomes will be particularly impacted in this group. Lung, skin and lower GI (colon and rectal) cancer also experienced significant declines in urgent referrals. Breast cancer was the least impacted of all in terms of urgent referrals but experienced a 25% reduction in first treatments. This highlights how much breast cancer diagnosis relies on screening programmes, which have suffered badly as a result of the pandemic(31). Although it is likely that some cancer patients have already been 'lost' to the system i.e. died of COVID-19 or other non-COVID-19 comorbidities, a significant number will now present with later stage disease.

Our findings also reflect socioeconomic inequalities with more profound decrease in urgent cancer referrals and first treatments for the most deprived populations, despite relatively better preservation of consultation rates. This is unexpected and extremely worrying, indicating greater disruption to the diagnostic pathway for patients living in more deprived areas, whose cancer outcomes were typically worse than their less deprived counterparts pre-pandemic(34,35). Resilience in primary care is key for the cancer diagnosis pathway and must be developed. We know that there are challenges associated with resourcing health services in poorer areas (the inverse care law(36)), resulting in fewer resources per head of sick patient(10) and shorter consultation times(37). Further research should focus on understanding to what extent complex morbidity, which is greater in poorer areas(8,38), contributes to the disruption of the cancer diagnostic pathway. Greater understanding would help health systems better prepare for the kind of disruption we have seen as a result of COVID-19.

Limitations

This study uses multiple data sets to analyse a complex and disjointed pathway. We include a primary care data set that uses a relatively small (500,000) patient sample. However, the CPRD data produces results that closely mirror the rates of consultation per patient (and their reduction) produced in NHS Digital's appointments data(39). In addition, the estimated reduction in urgent cancer referrals is close to those presented in the NCRAS's analysis of their cancer registry data (Table 1a & Table 1b). It is not yet possible to link these data on a patient basis due to delays in data access and once possible further research would be illuminating.

Conclusions

Our data reflects a disruption to a complex interaction of several systemic issues that place a great deal of impetus on the role of primary care in ensuring early diagnosis of cancer. Primary care was already under strain pre-pandemic, with low levels of investment and workforce deficits(40). Particularly in areas of high deprivation, general practice is under-funded and under staffed relative to need(7,8,10).

Early cancer diagnosis requires concordance of each participant and mechanism - including patients' awareness and ability to present with cancer symptoms, the ability of GPs to detect and urgently refer possible cancer cases and sufficient diagnostic capacity (in terms of both workforce and equipment) to enable swift referrals and minimise delays to diagnosis and treatment. Every one of these nodes on the pathway to early diagnosis has been affected by the

For peer review only

pandemic and the national policy response. However, further work is required as there is currently little understanding and even less evidence about how much each disruption is ultimately impacting cancer pathways.

The impact of the pandemic on cancer diagnosis and time to treatment shown here is very serious. However, what is more concerning is the unequal and inequitable impact on those worst off. Cancer as a disease area "*magnifies what we know to be true about the totality of the health care system. It exposes all its strengths and weaknesses*"(41). Our results further evidence the strain on primary care, the presence of the inverse care law(36), and the dire need to address the inequalities so sharply brought into focus by the pandemic. We need to address the disconnect between the importance we place on the role of primary care in cancer care and the resources we devote to it.

For peer review only

Figure legends:

Figure 1: Observed vs expected primary care activity, 01 Jan 2019 – 30 January 2021 (per 100,000 patient-months) (CPRD Aurum data) Panels: a. Consultations, b. Urgent cancer (2 week wait) referrals from primary care, c. Routine referrals from primary care

Figure 2: Percentage difference between observed and expected first treatments for new cancer and urgent cancer referrals by tumour location from NCRAS Cancer equity data pack (%, 01 Apr 2020 to 31 Jan 2021)

Figure 3: Percentage difference between observed and expected urgent cancer referrals and first treatments for cancer by IMD quintile (01 Apr 2020 – 31 Jan 2021)

Contributors: Data cleaning and analysis was conducted by TW. TW conceived the study with input from the coauthors and wrote the first draft. All the authors provided critical scholarly feedback on the manuscript. All the co-authors approved of the final version of the manuscript. TW and RS are the guarantors. The corresponding author attests that all listed authors meet authorship criteria and that no others meeting the criteria have been omitted.

Competing interests: None

Funding: This publication is funded through the UK Research and Innovation GCRF grant ES/P010962/1.

Data Availability statement:

The primary care activity data may be obtained from a third party and are not publicly available. We used de-identified primary care data from the Clinical Practice Research Datalink (CPRD). For more information please visit: https://www.cprd.com/Data-access, enquiries can be emailed to enquiries@cprd.gov.uk. Scientific approval for this study was given by the CPRD Independent Scientific Advisory Committee (ISAC). The study was approved by the Independent Scientific Advisory Committee for CPRD research (20_143). The data is provided by patients and collected by the NHS as part of their care and support.

Other data sources are available in a public, open access repository: Cancer Wait Times at https://www.england.nhs.uk/statistics/statistical-work-areas/cancer-waiting-times/ and the NCRAS Cancer data equity pack is available at http://www.ncin.org.uk/local_cancer_intelligence/cadeas.

For peer review only

References

1. Maringe C, Spicer J, Morris M, Purushotham A, Nolte E, Sullivan R, et al. The impact of the COVID-19 pandemic on cancer deaths due to delays in diagnosis in England, UK: a national, population-based, modelling study. Lancet Oncol. 2020;21(8).

2. Hanna TP, King WD, Thibodeau S, Jalink M, Paulin GA, Harvey-Jones E, et al. Mortality due to cancer treatment delay: systematic review and meta-analysis. BMJ. 2020;371.

3. Purushotham A, Roberts G, Haire K, Dodkins J, Harvey-Jones E, Han L, et al. The impact of national non-pharmaceutical interventions ('lockdowns') on the presentation of cancer patients. Ecancermedicalscience. 2021;15.

4. Health at a Glance: Europe 2020 [Internet]. OECD; 2020 [cited 2021 Oct 14]. (Health at a Glance: Europe). Available from: https://www.oecd-ilibrary.org/social-issues-migration-health/health-at-a-glance-europe-2020_82129230-en

5. Meiklejohn JA, Mimery A, Martin JH, Bailie R, Garvey G, Walpole ET, et al. The role of the GP in follow-up cancer care: a systematic literature review. Vol. 10, Journal of Cancer Survivorship. 2016.

6. PHE. Routes to Diagnosis [Internet]. National Cancer Registration and Analysis Service. [cited 2021 Oct 14]. Available from: http://www.ncin.org.uk/publications/routes_to_diagnosis

7. Parisi R, Lau YS, Bower P, Checkland K, Rubery J, Sutton M, et al. Rates of turnover among general practitioners: A retrospective study of all English general practices between 2007 and 2019. Vol. 11, BMJ Open. 2021.

8. Head A, Fleming K, Kypridemos C, Schofield P, Pearson-Stuttard J, O'Flaherty M. Inequalities in incident and prevalent multimorbidity in England, 2004–19: a population-based, descriptive study. Lancet Heal Longev. 2021;2(8).

9. DHSC, ONS. Direct and Indirect health impacts of COVID-19 in England [Internet]. London; 2021 Sep [cited 2021 Oct 14]. Available from: https://assets.publishing.service.gov.uk/government/uploads/system/uploads/attachment_data/file/1018698/S1373_Direct_and_Indirect_Health_Impacts_of_C19_Detailed_Paper_.pdf

10. Fisher R, Dunn P, Asaria M, Thorlby R. Level or not? Comparing general practice in areas of high and low socioeconomic deprivation in England. London; 2020 Sep.

11. NHS. NHS Long Term Plan Summary. NHS Improv. 2019;

12. Moscrop A. Health inequalities in primary care: Time to face justice. Vol. 62, British Journal of General Practice. 2012.

13. Cookson R, Mondor L, Asaria M, Kringos DS, Klazinga NS, Wodchis WP. Primary care and health inequality: Difference-in-difference study comparing England and Ontario. PLoS One. 2017;12(11).

14. IFG. Timeline of UK coronavirus lockdowns, March 2020 to March 2021 [Internet]. Institute for Government . 2021 [cited 2021 Oct 14]. Available from: https://www.instituteforgovernment.org.uk/sites/default/files/timeline-lockdown-web.pdf

15. Health and Social Care Committee & Science and Technology Committee. Coronavirus: lessons learned to date [Internet]. London; 2021 Oct [cited 2021 Oct 14]. Available from: https://committees.parliament.uk/work/657/coronavirus-lessons-learnt/news/157991/coronavirus-lessons-learned-to-date-report-published/

16. Carr MJ, Steeg S, Webb RT, Kapur N, Chew-Graham CA, Abel KM, et al. Effects of the COVID-19 pandemic on primary care-recorded mental illness and self-harm episodes in the UK: a population-based cohort study. Lancet Public Heal. 2021;6(2).

For peer review only

17. Herrett E, Gallagher AM, Bhaskaran K, Forbes H, Mathur R, Staa T van, et al. Data Resource Profile: Clinical Practice Research Datalink (CPRD). Int J Epidemiol. 2015;44(3).

18. Padmanabhan S, Carty L, Cameron E, Ghosh RE, Williams R, Strongman H. Approach to record linkage of primary care data from Clinical Practice Research Datalink to other health-related patient data: overview and implications. Eur J Epidemiol. 2019;34(1).

19. NHS, PHE. The Cancer Alliance Data, Evaluation and Analysis Service [Internet]. National Cancer Registration and Analysis Service: Local Cancer Intelligence. 2021 [cited 2021 Oct 14]. Available from: http://www.ncin.org.uk/local_cancer_intelligence/cadeas#covid-19

20. Breslow NE, Day NE. Statistical methods in cancer research. Volume II--The design and analysis of cohort studies. IARC scientific publications. 1987.

21. Raymond E, Thieblemont C, Alran S, Faivre S. Impact of the COVID-19 Outbreak on the Management of Patients with Cancer. Vol. 15, Targeted Oncology. 2020.

22. UK Government. Staying at home and away from others (social distancing) [Internet]. Cabinet Office. 2020 [cited 2021 Oct 29]. Available from: https://www.gov.uk/government/publications/full-guidance-on-staying-at-home-and-away-from-others

23. Wanat M, Hoste M, Gobat N, Anastasaki M, Böhmer F, Chlabicz S, et al. Transformation of primary care during the COVID-19 pandemic: Experiences of healthcare professionals in eight European countries. Br J Gen Pract. 2021;71(709).

24. Ekman B, Arvidsson E, Thulesius H, Wilkens J, Cronberg O. Impact of the Covid-19 pandemic on primary care utilization: evidence from Sweden using national register data. BMC Res Notes. 2021;14(1).

25. Arnold M, Rutherford MJ, Bardot A, Ferlay J, Andersson TML, Myklebust TÅ, et al. Progress in cancer survival, mortality, and incidence in seven high-income countries 1995–2014 (ICBP SURVMARK-2): a population-based study. Lancet Oncol. 2019;20(11).

26. Riera R, Bagattini ÂM, Pacheco RL, Pachito DV, Roitberg F, Ilbawi A. Delays and Disruptions in Cancer Health Care Due to COVID-19 Pandemic: Systematic Review. JCO Glob Oncol. 2021;(7).

27. Gurney JK, Millar E, Dunn A, Pirie R, Mako M, Manderson J, et al. The impact of the COVID-19 pandemic on cancer diagnosis and service access in New Zealand–a country pursuing COVID-19 elimination. Lancet Reg Heal - West Pacific. 2021;10.

28. Priou S, Lamé G, Chatellier G, Tournigand C, Kempf E. Effect of the COVID-19 pandemic on colorectal cancer care in France. Vol. 6, The Lancet Gastroenterology and Hepatology. 2021.

29. Jacob L, Loosen SH, Kalder M, Luedde T, Roderburg C, Kostev K. Impact of the COVID-19 pandemic on cancer diagnoses in general and specialized practices in Germany. Cancers (Basel). 2021;13(3).

30. Morris EJA, Goldacre R, Spata E, Mafham M, Finan PJ, Shelton J, et al. Impact of the COVID-19 pandemic on the detection and management of colorectal cancer in England: a population-based study. Lancet Gastroenterol Hepatol. 2021;6(3).

31. Cancer Waiting Times Team: NHS England and NHS Improvement. Cancer Wait Times Statistics [Internet]. NHS England. 2021 [cited 2021 Oct 25]. Available from: https://www.england.nhs.uk/statistics/statistical-work-areas/cancer-waiting-times/

32. Sud A, Torr B, Jones ME, Broggio J, Scott S, Loveday C, et al. Effect of delays in the 2-week-wait cancer referral pathway during the COVID-19 pandemic on cancer survival in the UK: a modelling study. Lancet Oncol. 2020;21(8).

33. Greenwood E, Swanton C. Consequences of COVID-19 for cancer care — a CRUK perspective. Vol. 18, Nature Reviews Clinical Oncology. 2021.

For peer review only

34. Coleman MP, Rachet B, Woods LM, Mitry E, Riga M, Cooper N, et al. Trends and socioeconomic inequalities in cancer survival in England and Wales up to 2001. Br J Cancer. 2004;90(7).

35. Cancer Research UK. Cancer incidence for all cancers combined: cancer incidence by deprivation [Internet]. 2020 [cited 2021 Oct 14]. Available from: https://www.cancerresearchuk.org/health-professional/cancer-statistics/incidence/all-cancers-combined#heading-Three

36. Tudor Hart J. THE INVERSE CARE LAW. Lancet. 1971;297(7696).

37. Gopfert A, Deeny SR, Fisher R, Stafford M. Primary care consultation length by deprivation and multimorbidity in England: An observational study using electronic patient records. Br J Gen Pract. 2021;71(704).

38. Ingram E, Ledden S, Beardon S, Gomes M, Hogarth S, Mcdonald H, et al. Household and area-level social determinants of multimorbidity: a systematic review. Vol. 75, Journal of Epidemiology and Community Health. 2021.

39. NHS. Appointments in General Practice - September 2021 [Internet]. NHS Digital. 2021 [cited 2021 Nov 16]. Available from: https://digital.nhs.uk/data-and-information/publications/statistical/appointments-in-general-practice

40. Roland M, Everington S. Tackling the crisis in general practice. BMJ. 2016;

41. Levit LA, Balogh EP, Nass SJ, Ganz PA. Delivering high-quality cancer care: Charting a new course for a system in crisis. Delivering High-Quality Cancer Care: Charting a New Course for a System in Crisis. 2014.

Key messages

- Primary care is key part of the pathway for early cancer diagnosis through both routine and 2ww referrals
- Cancer diagnosis rates have experienced a sustained fall since the start of the COVID-19 pandemic and introduction of non-pharmaceutical interventions (NPIs) 'lockdowns'.
- The fall in urgent cancer referral is larger than the fall in primary care contacts, implying that the content of consultations has shifted away from potential cancer diagnosis.
- Despite having a smaller reduction in primary care contact through the pandemic, patients living in poorer areas have had larger reductions in urgent cancer referrals and first treatments for new cancer.
- Government, patients and primary care staff must work together to catch up on missing diagnosis.
- Resilience in primary care is key for the cancer diagnosis pathway and must be developed for future disruptions, particularly in poorer areas where care is more complex.

For peer review only

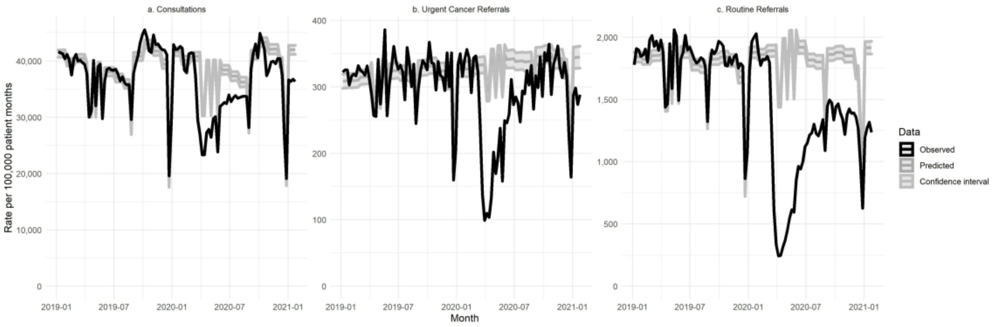

Figure 1: Observed vs expected primary care activity, 01 Jan 2019 – 30 January 2021 (per 100,000 patient-months) (CPRD Aurum data) Panels: a. Consultations, b. Urgent cancer (2 week wait) referrals from primary care, c. Routine referrals from primary care

381x127mm (300 x 300 DPI)

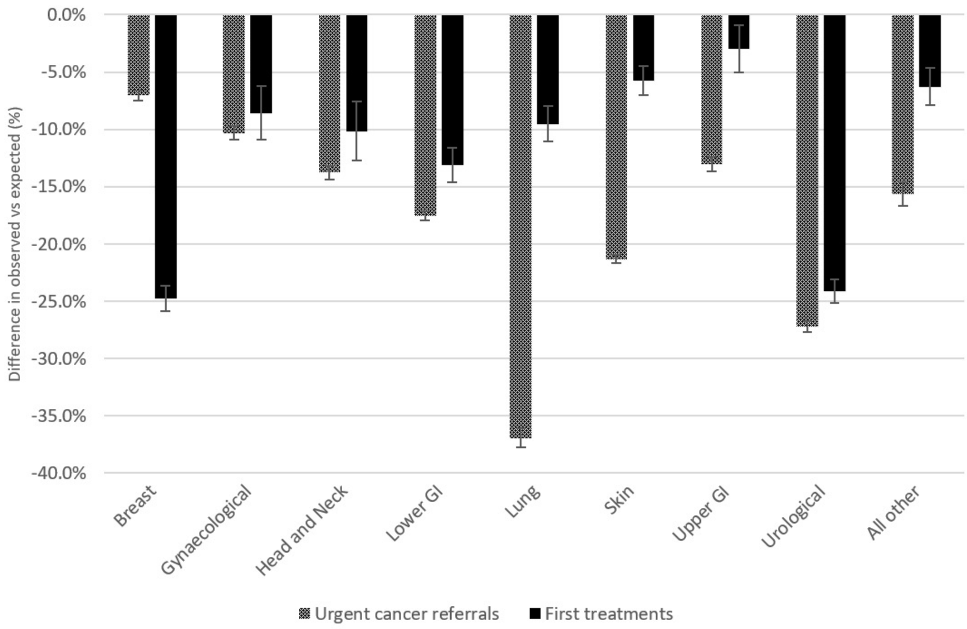

Figure 2: Percentage difference between observed and expected first treatments for new cancer and urgent cancer referrals by tumour location from NCRAS Cancer equity data pack (%, 01 Apr 2020 to 31 Jan 2021)

233x150mm (120 x 120 DPI)

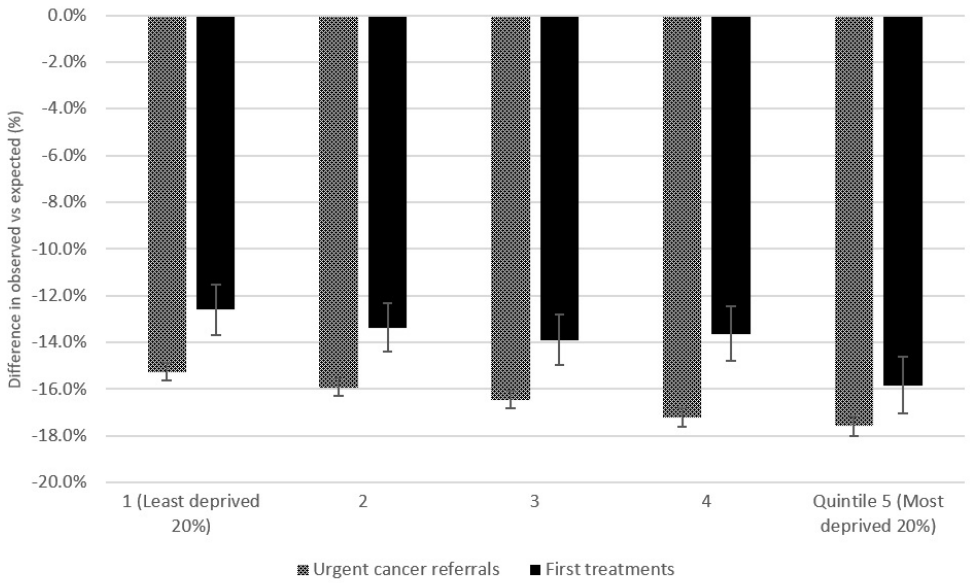

Figure 3: Percentage difference between observed and expected urgent cancer referrals and first treatments for cancer by IMD quintile (01 Apr 2020 – 31 Jan 2021)

205x122mm (120 x 120 DPI)

Annex 1: NCRAS data equity pack, technical notes

CADEAS and NCRAS have produced two equity data packs presenting the latest national data on the number of urgent suspected two-week wait referrals and first definitive treatments for cancer. These data packs are produced on the basis of the Cancer Wait Times data, with analysis from Hospital Episode Statistics (HES) and other sources outlined in their technical notes.

“Any differences between treatment volumes in the published official statistics and the volumes presented in this pack are because:

- Data was extracted from the CWT system at a slightly different time.
- Data included here is based on England residents only.

Additional logic has been applied to remove treatments where some of the information required for this equity analysis is missing or there are potential data quality issues, for example cases with a mismatch between the suspected cancer referral type and sex (eg. gynaecological cancer treatments for men, testicular cancer treatments for women), and suspected cancer referral type and age (eg. suspected children's cancer for patients aged 20 and over)." – NCRAS Cancer data equity pack technical notes, final tab within the downloaded spreadsheet. Available under “Links to data”: http://www.ncin.org.uk/local_cancer_intelligence/cadeas as at 26/01/2022

Annex 2: Consultation definition

CPRD Aurum data dictionary sets out the structure of the data. Within the consultation file there are two variables one can use to identify whether a primary care contact, rather than an administrative note (“EMIS® consultation source identifier” and “Consultation source code identifier”).

The EMIS consultation source identifier is the primary variable used. We include the following observations of this variable:

Acute visit, Casualty attendance, Clinic, Emergency appointment, Emergency consultation, Enterprise consultation, Face to face consultation, Follow-up/routine visit, Gp surgery, Home visit, Home visit note, Main surgery, Nursing home, Nursing home visit note, Online services message, Other, Residential home, Residential home visit note, Same day appointment, Surgery consultation, Telephone encounter, Urgent consultation, Walk-in centre, Walk-in clinic

We also include instances where EMIS consultation source identifier is “awaiting review” and the Consultation source code identifier is in the following list:

Consultation, visit, seen in gp unit, seen in private clinic, seen in rapid access clinic at gp surgery, seen in urgent care centre, online communication.

We then further exclude records on the basis of the category of staff responsible for the record. The “Job category” variable from the staff file, linked by the consultation id is used. We only include as a consultation records filled out by GPs, doctors, nurses and other health care professionals as defined in CPRD’s numerical codes listed below:

GP – 4, 5, 15, 24, 31, 181, 183

Dr – 1, 41, 91, 116, 119, 121, 126, 173, 177, 197

Nurse – 8, 9, 27, 33, 47, 48, 50, 55, 59, 60, 61, 111

Other healthcare professional - 2, 3, 6, 7, 10:14, 16, 17, 34:37, 42, 43, 52, 54, 58, 62:65, 68, 72, 73, 77, 80, 82, 83, 86:89, 94, 95, 97, 100:102, 105, 106, 112:114, 118, 122, 125, 127, 131, 135, 136, 138, 141, 142, 145, 148, 149, 154, 156, 158, 168, 185, 186, 188, 189, 204, 208

In Table A2.1 we present the CPRD Aurum Staff Category list.

In Table A2.2 we present the total number of consultations identified from 01 January 2016 to 31 January 2021, the count with each combination of staff category, “EMIS® consultation source identifier” and “Consultation source code identifier” in Table A2.2 we show the count of records that were excluded, highlighting those that were excluded on the basis of staff category, not the consultation file description variables.

For peer review only

Table A2.1: CPRD Aurum Staff Job Categories

1 Consultant	51 Helper/Assistant
2 Hospital Practitioner	52 Community Mental Health Nurse
3 Clinical Medical Officer	53 Senior Administrator
4 General Medical Practitioner	54 Technician - Healthcare Scientists
5 Salaried General Practitioner	55 Associate Practitioner - Nurse
6 Midwife - Sister/Charge Nurse	56 Senior Manager
7 Midwife	57 Community Administrator
8 Community Practitioner	58 Associate Specialist
9 Community Nurse	59 Student Practice Nurse
10 Chiropodist/Podiatrist	60 Nurse Manager
11 Dietitian	61 Sister/Charge Nurse
12 Pharmacist	62 Psychotherapist
13 Clinical Psychologist	63 Osteopath
14 Health Care Support Worker	64 Social Care Support Worker
15 Associate Practitioner - General Practitioner	65 Assistant Psychologist
16 Counsellor	66 Officer
17 Phlebotomist	67 Technician - Admin & Clerical
18 Clerical Worker	68 Psychiatrist
19 Manager	69 Health Records Clerk
20 Analyst	70 Desktop Support Technician
21 System Administrator	71 Dispenser
22 Desktop Support Administrator	72 Clinical Assistant
23 System Worker	73 Practitioner
24 GP Registrar	74 Information Officer
25 Medical Student	75 Network Administrator
26 Other Community Health Service - Admin Clerk	76 Chaplain
27 Specialist Nurse Practitioner	77 Student Physiotherapist
28 Receptionist	78 Paramedic Specialist Practitioner
29 Secretary	79 Clinical Team Manager
30 Medical Secretary	80 Physiotherapist Specialist Practitioner
31 Sessional GP	81 Helpdesk Technician
32 Clinical Application Administrator	82 Radiographer
33 Nurse Consultant	83 Other Community Health Service
34 Physiotherapist	84 Call Operator
35 Specialist Practitioner	85 Community Worker (children)
36 Healthcare Assistant	86 Paramedic Consultant
37 Medical Technical Officer - Pharmacy	87 Associate Practitioner
38 Health Records Administrator	88 Modern Matron
39 Helpdesk Administrator	89 Asst. Clinical Medical Officer
40 Appointments Clerk	90 Community Team Manager
41 Senior House Officer	91 Specialist Registrar
42 Social Worker	92 Chiropodist/Podiatrist Manager
43 Trainee Practitioner	93 Radiographer - Therapeutic, Manager
44 Network Technician	94 Optometrist
45 Clinical Coder	95 Assistant Practitioner
46 Medical Records Clerk	96 Community Learning Disabilities Nurse
47 Staff Nurse	97 Technician - Additional Clinical Services
48 Enrolled Nurse	98 Student Health Visitor
49 Multi Therapist	99 Interpreter
50 Nursery Nurse	100 Medical Technical Officer

For peer review only

101 Midwife - Specialist Practitioner
102 Occupational Therapist
103 Chief Executive
104 Audit Manager
105 Paramedic
106 Physiotherapist Consultant
107 Availability Monitor
108 Medical Laboratory Assistant
109 Gateway Worker
110 Medical Records Manager
111 Student Nurse - Adult Branch
112 Audiologist
113 Radiographer - Diagnostic
114 Therapist
115 Student District Nurse
116 House Officer - Post Registration
117 Speech & Language Therapist
118 Dietitian Specialist Practitioner
119 Trust Grade Doctor - SHO level
120 Director of Public Health
121 Staff Grade
122 Patient Welfare Officer
123 Occupational Therapy Specialist Practitioner
124 Technician - PS&T
125 Chiropodist/Podiatrist Consultant
126 Trust Grade Doctor - Career Grade level
127 Student Community Practitioner
128 Healthcare Scientist
129 Waiting List Clerk
130 Clinical Director
131 Pre-reg Pharmacist
132 Mental Health Act Administrator
133 Ward Clerk
134 Support, Time, Recovery Worker
135 Art Therapist Specialist Practitioner
136 Physiotherapist Manager
137 Healthcare Cadet
138 Dietitian Consultant
139 Orthoptist Manager
140 Social work assistant (mental health)
141 Chiropodist/Podiatrist Specialist Practitioner
142 Student Technician
143 Complaints Investigator
144 Trainee Scientist
145 Radiographer - Diagnostic, Manager
146 Social services care manager (mental health)
147 Dietitian Manager
148 Midwife - Consultant
149 Art Therapist Consultant
150 Paramedic Manager
151 Finance Director
152 Senior social worker (adults)
153 Student Midwife
154 Radiologist
155 Ward Manager
156 Midwife - Manager
157 Waiting List Manager
158 Radiographer - Diagnostic, Specialist Practitioner
159 Biomedical Scientist
160 Board Level Director
161 Non Executive Director
162 Nursing Cadet
163 Porter
164 Social services care manager (adults)
165 Student Psychotherapist
166 Orthoptist
167 Clinical Director - Medical
168 Approved Social Worker
169 Student Community Mental Health Nurse
170 Other Executive Director
171 Student Orthoptist
172 Childcare Co-ordinator
173 House Officer - Pre Registration
174 SODP
175 Outpatient Manager
176 Medical Director
177 Trust Grade Doctor - Specialist Registrar level
178 Senior Clinical Medical Officer
179 Consultant Healthcare Scientist
180 Reporting Radiographer
181 Locum GP
182 Researcher
183 Assistant GP
184 Special salary scale in Public Health Medicine
185 Advanced Practitioner
186 Health Visitor
187 Dental Assistant Clinical Director
188 Other Community Health Service - Social Care Worker
189 Physician Assistant
190 Deputising Doctor
191 Student Occupational Health Nurse
192 Senior social worker (mental health)
193 Regional Dental Officer
194 Trainer
195 Cytoscreener
196 Chair
197 Trust Grade Doctor - House Officer level
198 Art Therapist
199 Multi Therapist Specialist Practitioner
200 Drama Therapist
201 Healthcare Science Assistant
202 Social work assistant (adults)
203 Social work team manager (adults)

For peer review only

204 Intermediate Care worker
205 Student Occupational Therapist
206 Student Dietitian
207 Healthcare Science Associate
208 Child Protection worker
209 Professor
210 General Dental Practitioner
211 Student School Nurse
212 Occupational Therapist Consultant
213 Intermediate Care staff
214 Home help
215 Art, Music & Drama Student
216 Specialist Healthcare Scientist
217 Social Services information manager

For peer review only

Table A2.2: Number of observations by EMIS® consultation source identifier, Consultation source code identifier and Staff Job Category, with an indicator for whether it was included as a consultation: Include: 1 = Include, 0 = Exclude, “Excl. job title” = Excluded on the basis of job title.

Include	Staff Job Category	Consultation source code identifier	EMIS consultation source identifier	Count
1	gp	gp surgery	gp surgery	3,119,080
1	nurse	gp surgery	gp surgery	1,692,606
1	gp	telephone consultation	telephone consultation	1,471,946
1	other care provider	gp surgery	gp surgery	977,067
1	nurse	telephone consultation	telephone consultation	196,665
1	gp	telephone call to a patient	telephone call to a patient	108,025
1	gp	home visit note	home visit note	83,600
1	other care provider	telephone consultation	telephone consultation	81,980
1	nurse	telephone call to a patient	telephone call to a patient	32,423
1	gp	face to face consultation	face to face consultation	25,435
1	nurse	home visit note	home visit note	24,174
1	other care provider	telephone call to a patient	telephone call to a patient	23,194
1	gp	gp surgery	surgery consultation	22,756

1	gp	nursing home visit note	nursing home visit note	21,357
1	nurse	face to face consultation	face to face consultation	17,580
1	gp	enterprise consultation	enterprise consultation	14,904
1	gp	telephone call from a patient	telephone call from a patient	13,062
1	gp	routine consultation	surgery consultation	11,668
1	other care provider	home visit note	home visit note	10,853
1	gp	provision of general practitioner intermediate care	gp surgery	10,441
1	gp	emergency consultation	emergency consultation	10,351
1	gp	residential home visit note	residential home visit note	9,579
1	other care provider	face to face consultation	face to face consultation	9,350
1	gp	emergency appointment	emergency appointment	8,687
1	gp	urgent consultation	urgent consultation	8,155
1	gp	walk-in clinic	walk-in clinic	7,908
1	dr	gp surgery	gp surgery	7,654
1	gp	other note	other	7,520
1	gp	face to face consultation	surgery consultation	6,932
1	nurse	gp surgery	surgery consultation	5,318
1	gp	seen in gp unit	surgery consultation	4,687
1	gp	consultation via video conference	awaiting review	4,653
1	nurse	enterprise consultation	enterprise consultation	4,460
1	other care provider	provision of general practitioner intermediate care	gp surgery	4,369
1	gp	clinic note	surgery consultation	3,823
1	nurse	residential home visit note	residential home visit note	3,612
1	nurse	clinic note	clinic	3,585
1	nurse	nursing home visit note	nursing home visit note	3,528
1	nurse	face to face consultation	surgery consultation	3,442
1	gp	online communication	awaiting review	3,410
1	other care provider	other note	other	3,406
1	other care provider	seen in gp unit	gp surgery	2,781
1	gp	e-mail consultation	awaiting review	2,523
1	nurse	other note	other	2,449
1	other care provider	gp surgery	surgery consultation	2,334
1	other care provider	enterprise consultation	enterprise consultation	2,318
1	other care provider	telephone call from a patient	telephone call from a patient	2,211
1	nurse	telephone call from a patient	telephone call from a patient	2,183
1	gp	routine consultation	awaiting review	2,117
1	nurse	emergency appointment	emergency appointment	2,041
1	gp	home visit note	home visit	2,021
1	gp	seen in gp unit	gp surgery	1,896
1	nurse	provision of general practitioner intermediate care	gp surgery	1,762

For peer review only

1	other care provider	clinic note	clinic	1,699
1	nurse	clinic note	surgery consultation	1,628
1	gp	clinic note	clinic	1,623
1	nurse	routine consultation	surgery consultation	1,578
1	nurse	seen in gp unit	surgery consultation	1,426
1	nurse	walk-in clinic	walk-in clinic	1,412
1	nurse	gp surgery	clinic	1,355
1	other care provider	routine consultation	other	1,303
1	other care provider	clinic note	surgery consultation	1,297
1	gp	face to face consultation	emergency consultation	1,292
1	other care provider	walk-in clinic	walk-in clinic	1,216
1	gp	telephone encounter	telephone encounter	1,184
1	gp	online communication	online services message	1,139
1	gp	other consultation medium used	awaiting review	1,134
1	other care provider	residential home visit note	residential home visit note	1,113
1	nurse	seen in gp unit	gp surgery	1,103
1	other care provider	nursing home visit note	nursing home visit note	1,081
1	other care provider	face to face consultation	surgery consultation	1,045
1	other care provider	seen in gp unit	surgery consultation	1,043
1	nurse	emergency consultation	emergency consultation	1,024
1	nurse	urgent consultation	urgent consultation	959
1	gp	extended hours consultation	awaiting review	924
1	gp	routine consultation	other	922
1	gp	home visit note	other	835
1	gp	gp surgery	face to face consultation	808
1	other care provider	gp surgery	clinic	746
1	other care provider	routine consultation	surgery consultation	738
1	gp	consultation via multimedia	awaiting review	734
1	gp	face to face consultation with relative/carer	awaiting review	669
1	nurse	e-mail consultation	awaiting review	638
1	nurse	routine consultation	awaiting review	574
1	nurse	consultation via video conference	awaiting review	505
1	nurse	routine consultation	other	478
1	other care provider	online communication	awaiting review	473
1	nurse	same day appointment	same day appointment	468
1	gp	face to face consultation	gp surgery	461
1	gp	same day appointment	same day appointment	457
1	nurse	gp surgery	face to face consultation	455
1	gp	telephone encounter	telephone consultation	429
1	nurse	face to face consultation	emergency consultation	420

For peer review only

1	gp	group consultation	awaiting review	402
1	nurse	home visit note	other	402
1	nurse	seen in urgent care centre	awaiting review	344
1	gp	face to face consultation	emergency appointment	330
1	gp	seen in urgent care centre	awaiting review	322
1	nurse	telephone encounter	telephone encounter	314
1	other care provider	consultation via video conference	awaiting review	288
1	gp	telephone consultation	telephone call to a patient	287
1	dr	telephone consultation	telephone consultation	279
1	nurse	online communication	awaiting review	279
1	other care provider	face to face consultation	awaiting review	272
1	other care provider	home visit note	home visit	262
1	other care provider	routine consultation	awaiting review	260
1	nurse	home visit note	home visit	246
1	gp	consultation via sms text message	awaiting review	244
1	other care provider	online communication	online services message	241
1	other care provider	emergency consultation	emergency consultation	235
1	other care provider	emergency appointment	emergency appointment	231
1	gp	telephone encounter	telephone call to a patient	226
1	gp	residential home visit note	residential home	225
1	gp	face to face consultation	awaiting review	224
1	nurse	face to face consultation	gp surgery	221
1	other care provider	gp surgery	face to face consultation	209
1	nurse	extended hours consultation	awaiting review	186
1	gp	seen in rapid access clinic at gp surgery	awaiting review	182
1	gp	school visit note	awaiting review	181
1	other care provider	telephone consultation	telephone call to a patient	179
1	nurse	face to face consultation	awaiting review	166
1	gp	home visit note	acute visit	153
1	nurse	online communication	online services message	145
1	other care provider	group consultation	awaiting review	133
1	gp	seen in gp unit	awaiting review	129
1	dr	clinic note	surgery consultation	125
1	other care provider	telephone encounter	telephone encounter	119
1	other care provider	extended hours consultation	awaiting review	116
1	nurse	face to face consultation with relative/carer	awaiting review	115
1	nurse	seen in gp unit	awaiting review	109
1	gp	night visit note	awaiting review	108

For peer review only

1	nurse	school visit note	awaiting review	107
1	nurse	group consultation	awaiting review	102
1	gp	other consultation medium used	other	99
1	gp	children's home visit note	awaiting review	95
1	gp	telephone consultation	telephone call from a patient	94
1	gp	administration note	other	91
1	nurse	telephone encounter	telephone call to a patient	86
1	other care provider	e-mail consultation	awaiting review	71
1	nurse	consultation via multimedia	awaiting review	65
1	gp	gp surgery	clinic	63
1	nurse	telephone consultation	telephone call to a patient	63
1	other care provider	face to face consultation with relative/carer	awaiting review	62
1	gp	consultation via telemedicine web camera	awaiting review	61
1	dr	telephone call to a patient	telephone call to a patient	60
1	nurse	laboratory result	clinic	55
1	other care provider	other consultation medium used	awaiting review	53
1	nurse	other consultation medium used	awaiting review	51
1	nurse	district nurse visit	awaiting review	48
1	gp	clinic note	gp surgery	47
1	other care provider	clinic note	gp surgery	45
1	other care provider	urgent consultation	urgent consultation	42
1	other care provider	home visit note	other	41
1	other care provider	laboratory result	clinic	40
1	gp	laboratory result	acute visit	39
1	dr	home visit note	home visit note	38
1	other care provider	telephone encounter	telephone call to a patient	38
1	nurse	telephone encounter	telephone consultation	33
1	other care provider	consultation via sms text message	awaiting review	28
1	gp	walk-in clinic	walk-in centre	27
1	nurse	walk-in clinic	walk-in centre	27
1	other care provider	walk-in clinic	clinic	27
1	nurse	children's home visit note	awaiting review	25
1	gp	gp surgery	main surgery	24
1	nurse	residential home visit note	residential home	22
1	other care provider	seen in urgent care centre	awaiting review	22
1	nurse	night visit note	awaiting review	20
1	other care provider	children's home visit note	awaiting review	19
1	other care provider	consultation via multimedia	awaiting review	19

For peer review only

1	gp	home visit note	nursing home visit note	17
1	gp	nursing home visit note	nursing home	17
1	gp	residential home visit note	nursing home	15
1	other care provider	face to face consultation	gp surgery	14
1	other care provider	night visit note	awaiting review	14
1	other care provider	other consultation medium used	other	14
1	nurse	clinic note	gp surgery	13
1	nurse	walk-in clinic	clinic	13
1	nurse	home visit note	acute visit	10
1	nurse	consultation via sms text message	awaiting review	< 10
1	nurse	consultation via telemedicine web camera	awaiting review	< 10
1	dr	consultation via video conference	awaiting review	< 10
1	dr	nursing home visit note	nursing home visit note	< 10
1	other care provider	home visit note	nursing home visit note	< 10
1	dr	other note	other	< 10
1	gp	telephone encounter	telephone call from a patient	< 10
1	gp	twilight visit note	awaiting review	< 10
1	other care provider	laboratory result	acute visit	< 10
1	dr	face to face consultation	face to face consultation	< 10
1	gp	home visit note	follow-up/routine visit	< 10
1	gp	other consultation medium used	casualty attendance	< 10
1	other care provider	home visit note	residential home visit note	< 10
1	other care provider	other note	gp surgery	< 10
1	gp	home visit note	awaiting review	< 10
1	gp	non-consultation medication data	awaiting review	< 10
1	gp	remote consultation	awaiting review	< 10
1	gp	third party consultation	casualty attendance	< 10
1	nurse	home visit note	follow-up/routine visit	< 10
1	nurse	telephone encounter	telephone call from a patient	< 10
1	nurse	third party consultation	casualty attendance	< 10
1	dr	enterprise consultation	enterprise consultation	< 10
1	dr	telephone call from a patient	telephone call from a patient	< 10
1	gp	district nurse visit	awaiting review	< 10
1	gp	e-mail received from patient	acute visit	< 10
1	gp	hospital outpatient report	casualty attendance	< 10
1	gp	joint consultation	awaiting review	< 10
1	gp	pharmacy consultation	awaiting review	< 10
1	gp	telephone call to a patient	telephone consultation	< 10
1	nurse	administration note	other	< 10
1	nurse	face to face consultation	emergency appointment	< 10
1	other care provider	consultation via telemedicine web camera	awaiting review	< 10

For peer review only

1	other care provider	face to face consultation	emergency appointment	< 10
1	other care provider	face to face consultation	emergency consultation	< 10
1	other care provider	home visit note	acute visit	< 10
1	other care provider	pharmacy consultation	awaiting review	< 10
1	other care provider	residential home visit note	residential home	< 10
1	other care provider	telephone encounter	telephone consultation	< 10
1	dr	group consultation	awaiting review	< 10
1	dr	home visit note	acute visit	< 10
1	gp	clinic note	follow-up/routine visit	< 10
1	gp	emergency consultation	casualty attendance	< 10
1	gp	home visit note	nursing home	< 10
1	nurse	seen in rapid access clinic at gp surgery	awaiting review	< 10
1	nurse	telephone consultation	telephone call from a patient	< 10
1	nurse	twilight visit note	awaiting review	< 10
1	other care provider	administration note	other	< 10
1	other care provider	children's home visit note	clinic	< 10
1	other care provider	home visit note	awaiting review	< 10
1	other care provider	twilight visit note	awaiting review	< 10
excl. job cat		gp surgery	gp surgery	875,291
excl. job cat		other note	other	31,332
excl. job cat		telephone consultation	telephone consultation	29,455
excl. job cat		online communication	online services message	14,055
excl. job cat		telephone call to a patient	telephone call to a patient	13,935
excl. job cat		provision of general practitioner intermediate care	gp surgery	9,791
excl. job cat		telephone call from a patient	telephone call from a patient	7,098
excl. job cat		seen in gp unit	gp surgery	6,240
excl. job cat		home visit note	home visit note	4,776
excl. job cat		routine consultation	other	4,248
excl. job cat		gp surgery	surgery consultation	3,047
excl. job cat		home visit note	other	2,664
excl. job cat		face to face consultation	face to face consultation	2,071
excl. job cat		face to face consultation	surgery consultation	1,177
excl. job cat		online communication	awaiting review	1,098
excl. job cat		nursing home visit note	nursing home visit note	708
excl. job cat		routine consultation	surgery consultation	509
excl. job cat		consultation via video conference	awaiting review	494
excl. job cat		routine consultation	awaiting review	453
excl. job cat		enterprise consultation	enterprise consultation	398
excl. job cat		administration note	other	376
excl. job cat		telephone encounter	telephone encounter	376

For peer review only

excl. job cat		home visit note	awaiting review	371
excl. job cat		clinic note	clinic	337
excl. job cat		clinic note	surgery consultation	329
excl. job cat		residential home visit note	residential home visit note	327
excl. job cat		face to face consultation	gp surgery	305
excl. job cat		face to face consultation	awaiting review	293
excl. job cat		home visit note	home visit	250
excl. job cat		urgent consultation	urgent consultation	240
excl. job cat		walk-in clinic	walk-in clinic	196
excl. job cat		group consultation	awaiting review	170
excl. job cat		seen in gp unit	surgery consultation	148
excl. job cat		e-mail consultation	awaiting review	145
excl. job cat		emergency consultation	emergency consultation	121
excl. job cat		face to face consultation with relative/carer	awaiting review	116
excl. job cat		consultation via multimedia	awaiting review	75
excl. job cat		seen in gp unit	awaiting review	75
excl. job cat		children's home visit note	awaiting review	63
excl. job cat		other note	gp surgery	54
excl. job cat		other consultation medium used	other	44
excl. job cat		other consultation medium used	awaiting review	42
excl. job cat		extended hours consultation	awaiting review	37
excl. job cat		gp surgery	face to face consultation	36
excl. job cat		emergency appointment	emergency appointment	35
excl. job cat		gp surgery	clinic	32
excl. job cat		face to face consultation	emergency consultation	25
excl. job cat		residential home visit note	residential home	23
excl. job cat		telephone encounter	telephone call to a patient	23
excl. job cat		night visit note	awaiting review	14
excl. job cat		home visit note	acute visit	13
excl. job cat		walk-in clinic	walk-in centre	11
excl. job cat		district nurse visit	awaiting review	< 10
excl. job cat		seen in urgent care centre	awaiting review	< 10
excl. job cat		twilight visit note	awaiting review	< 10
excl. job cat		clinic note	gp surgery	< 10
excl. job cat		laboratory result	acute visit	< 10
excl. job cat		telephone consultation	telephone call to a patient	< 10
excl. job cat		walk-in clinic	clinic	< 10
excl. job cat		telephone encounter	telephone call from a patient	< 10
excl. job cat		emergency consultation	casualty attendance	< 10
excl. job cat		pharmacy consultation	awaiting review	< 10
excl. job cat		third party consultation	casualty attendance	< 10
excl. job cat		case conference	gp surgery	< 10
excl. job cat		emergency consultation	awaiting review	< 10
excl. job cat		gp surgery	main surgery	< 10
excl. job cat		home visit note	follow-up/routine visit	< 10
excl. job cat		non-consultation medication data	casualty attendance	< 10
excl. job cat		nursing home visit note	nursing home	< 10

For peer review only

excl. job cat		remote consultation	awaiting review	< 10
excl. job cat		same day appointment	same day appointment	< 10
excl. job cat		school visit note	awaiting review	< 10
excl. job cat		telephone consultation	awaiting review	< 10
excl. job cat		telephone encounter	telephone consultation	< 10
excl. job cat		third party consultation	awaiting review	< 10
0	gp	externally entered note	externally entered	3,467,397
0		scanned document	docman	3,183,781
0		administration note	administration note	968,767
0				737,843
0		scanned document	scanned document	727,269
0	gp	administration note	administration note	725,612
0		inbound document	inbound document	402,647
0		awaiting clinical code migration to emis web	awaiting review	385,598
0	nurse	externally entered note	externally entered	303,830
0	gp	awaiting clinical code migration to emis web	awaiting review	261,627
0		administration note	administration	256,604
0	other care provider	scanned document	docman	255,022
0		externally entered note	externally entered	252,167
0	gp			208,462
0	gp	outbound referral	outbound referral	197,534
0	gp	awaiting clinical code migration to emis web	gp surgery	162,048
0	other care provider	administration note	administration note	160,918
0		awaiting clinical code migration to emis web	third party consultation	147,374
0	gp	scanned document	scanned document	145,355
0		awaiting clinical code migration to emis web	gp surgery	127,041
0	gp	awaiting clinical code migration to emis web	results recording	122,118
0		awaiting clinical code migration to emis web	other	121,401
0	gp	awaiting clinical code migration to emis web	surgery consultation	107,304
0		awaiting clinical code migration to emis web		104,693
0	gp	inbound document	inbound document	102,534
0	nurse	awaiting clinical code migration to emis web	awaiting review	94,158
0	other care provider	awaiting clinical code migration to emis web	gp surgery	86,398
0	other care provider	awaiting clinical code migration to emis web	awaiting review	85,745
0	nurse	administration note	administration note	84,051
0	gp	awaiting clinical code migration to emis web		77,585
0	nurse	awaiting clinical code migration to emis web	gp surgery	75,552

For peer review only

0	other care provider			66,366
0	other care provider	externally entered note	externally entered	62,810
0		externally entered note	externally entered note	61,848
0		awaiting clinical code migration to emis web	repeat issue	61,683
0	nurse			58,019
0	gp	telephone triage encounter	telephone triage encounter	50,662
0		third party consultation	third party consultation	44,609
0	other care provider	scanned document	scanned document	38,341
0	nurse	awaiting clinical code migration to emis web		35,376
0	nurse	awaiting clinical code migration to emis web	surgery consultation	27,699
0	gp	administration note	administration	27,390
0	gp	awaiting clinical code migration to emis web	telephone consultation	26,214
0	gp	awaiting clinical code migration to emis web	other	26,204
0		awaiting clinical code migration to emis web	scanned document	25,627
0		awaiting clinical code migration to emis web	administration note	24,458
0		gp surgery	awaiting review	24,337
0		awaiting clinical code migration to emis web	touchscreen	24,311
0	gp	third party consultation	third party consultation	23,952
0		awaiting clinical code migration to emis web	patientchase insert	22,515
0	gp	laboratory result	results recording	22,066
0	gp	externally entered note	externally entered note	20,864
0	gp	awaiting clinical code migration to emis web	administration note	20,729
0	other care provider	inbound document	inbound document	20,159
0	gp	telephone call to relative/carer	telephone call to relative/carer	19,234
0	other care provider	awaiting clinical code migration to emis web		17,081
0		awaiting clinical code migration to emis web	surgery consultation	15,972
0	nurse	awaiting clinical code migration to emis web	clinic	15,892
0	gp	other note	other note	15,816
0	gp	gp surgery	awaiting review	15,810
0	gp	discussion with colleague	discussion with colleague	12,973
0	gp	awaiting clinical code migration to emis web	telephone call to a patient	11,782
0		repeat prescription	repeat issue	11,542
0	other care provider	administration note	administration	11,037
0	nurse	awaiting clinical code migration to emis web	other	11,008

For peer review only

0		hospital outpatient report	hospital outpatient report	10,033
0	nurse	inbound document	inbound document	9,304
0	nurse	scanned document	scanned document	7,878
0	other care provider	awaiting clinical code migration to emis web	repeat issue	7,745
0	gp	awaiting clinical code migration to emis web	outbound referral	7,402
0	other care provider	externally entered note	externally entered note	6,736
0	gp	telephone call from relative/carer	telephone call from relative/carer	6,626
0	other care provider	awaiting clinical code migration to emis web	surgery consultation	6,514
0		awaiting clinical code migration to emis web	inbound document	6,495
0	nurse	gp surgery	awaiting review	6,475
0	other care provider	awaiting clinical code migration to emis web	other	6,315
0	nurse	administration note	administration	6,285
0		outbound referral	outbound referral	6,194
0	nurse	telephone triage encounter	telephone triage encounter	6,074
0	gp	clinic note	clinic note	5,834
0	gp	awaiting clinical code migration to emis web	repeat issue	5,803
0	gp	non-consultation data	non-consultation data	5,552
0	other care provider	third party consultation	third party consultation	5,505
0	other care provider	gp surgery	awaiting review	5,332
0	gp	awaiting clinical code migration to emis web	telephone call from a patient	5,282
0	nurse	externally entered note	externally entered note	5,251
0	gp	face to face consultation	triage	5,196
0	nurse	telephone call to relative/carer	telephone call to relative/carer	5,191
0		awaiting clinical code migration to emis web	mail to patient	5,178
0		non-consultation data	non-consultation data	4,933
0	gp	e-mail received from patient	e-mail received from patient	4,877
0	nurse	outbound referral	outbound referral	4,787
0		medication requested	repeat issue	4,639
0	nurse	third party consultation	third party consultation	4,605
0	nurse	awaiting clinical code migration to emis web	results recording	4,405
0	gp	awaiting clinical code migration to emis web	scanned document	4,384
0		other note	other note	4,375
0		awaiting clinical code migration to emis web	mjog	4,221
0	other care provider	other note	other note	4,071
0		mail to patient	mail to patient	3,924
0	other care provider	awaiting clinical code migration to emis web	clinic	3,859
0		e-mail received from patient	e-mail received from patient	3,632

For peer review only

0		awaiting clinical code migration to emis web	out of hours, non practice	3,607
0	other care provider	clinic note	clinic note	3,371
0	gp	awaiting clinical code migration to emis web	home visit note	3,370
0	gp	hospital outpatient report	hospital outpatient report	3,359
0	nurse	nurse telephone triage	nurse telephone triage	3,274
0	nurse	clinic note	clinic note	3,252
0	gp	awaiting clinical code migration to emis web	clinic	3,245
0	other care provider	awaiting clinical code migration to emis web	administration note	3,044
0	gp	awaiting clinical code migration to emis web	third party consultation	3,038
0	other care provider	awaiting clinical code migration to emis web	medicine management	2,829
0	other care provider	telephone call to relative/carer	telephone call to relative/carer	2,807
0	nurse	awaiting clinical code migration to emis web	telephone consultation	2,782
0		awaiting clinical code migration to emis web	clinic	2,775
0		laboratory result	laboratory result	2,727
0	gp	awaiting clinical code migration to emis web	inbound document	2,605
0		telephone call from relative/carer	telephone call from relative/carer	2,594
0		clinic note	clinic note	2,579
0	gp	laboratory result	laboratory result	2,511
0	other care provider	repeat prescription	repeat issue	2,511
0		awaiting clinical code migration to emis web	home of patient	2,454
0	gp	awaiting clinical code migration to emis web	out of hours, non practice	2,365
0	gp	awaiting clinical code migration to emis web	nhs direct report	2,334
0		awaiting clinical code migration to emis web	results recording	2,185
0	dr	third party consultation	third party consultation	2,146
0	other care provider	awaiting clinical code migration to emis web	third party consultation	2,095
0	nurse	awaiting clinical code migration to emis web	administration note	2,075
0		administration note	patientchase insert	1,855
0		clinic note	awaiting review	1,790
0		hospital inpatient report	hospital inpatient report	1,760
0		e-mail received from patient	docman	1,753
0	nurse	other note	other note	1,708
0	nurse	non-consultation data	non-consultation data	1,681
0	gp	awaiting clinical code migration to emis web	referral letter	1,660
0		laboratory result	results recording	1,624

For peer review only

0		awaiting clinical code migration to emis web	non-consultation data	1,537
0	gp	awaiting clinical code migration to emis web	medicine management	1,510
0	nurse	laboratory result	results recording	1,464
0	nurse	telephone call from relative/carer	telephone call from relative/carer	1,401
0		administration note	scanned document	1,373
0		telephone triage encounter	telephone triage encounter	1,353
0	gp	medication requested	awaiting review	1,347
0	nurse	discussion with colleague	discussion with colleague	1,329
0	gp	awaiting clinical code migration to emis web	telephone triage encounter	1,313
0	nurse	awaiting clinical code migration to emis web	telephone call to a patient	1,243
0	other care provider	non-consultation data	non-consultation data	1,239
0	gp	case conference	awaiting review	1,225
0	gp	awaiting clinical code migration to emis web	externally entered note	1,206
0		telephone call to relative/carer	telephone call to relative/carer	1,191
0	nurse	awaiting clinical code migration to emis web	third party consultation	1,170
0	gp	e-mail sent to patient	awaiting review	1,162
0	dr	administration note	administration	1,159
0		other note		1,156
0		administration note		1,120
0	dr	externally entered note	externally entered	1,113
0	gp	awaiting clinical code migration to emis web	face to face consultation	1,112
0	other care provider	telephone triage encounter	telephone triage encounter	1,107
0	nurse	laboratory result	laboratory result	1,064
0	other care provider	outbound referral	outbound referral	1,052
0	gp	clinic note	awaiting review	1,030
0	other care provider	mail to patient	patientchase insert	1,019
0	gp	repeat prescription	awaiting review	1,003
0	gp	awaiting clinical code migration to emis web	data transferred from other system	983
0		awaiting clinical code migration to emis web	non-consultation medication data	980
0	gp	awaiting clinical code migration to emis web	nursing home visit note	971
0	gp	repeat prescription	repeat issue	964
0		awaiting clinical code migration to emis web	telephone call to a patient	901
0	dr			885
0	nurse	awaiting clinical code migration to emis web	face to face consultation	853
0	other care provider	hospital outpatient report	hospital outpatient report	828
0		clinic note	community clinic	823

For peer review only

0		awaiting clinical code migration to emis web	gp2gp import	822
0		awaiting clinical code migration to emis web	letter from outpatients	809
0	other care provider	mail to patient	mail to patient	801
0	other care provider	telephone call from relative/carer	telephone call from relative/carer	789
0		awaiting clinical code migration to emis web	externally entered note	769
0		awaiting clinical code migration to emis web	referral letter	751
0	other care provider	awaiting clinical code migration to emis web	inbound document	719
0		e-mail sent to patient	awaiting review	711
0	gp	hospital inpatient report	hospital inpatient report	710
0	nurse	awaiting clinical code migration to emis web	repeat issue	694
0	gp	awaiting clinical code migration to emis web	acute visit	693
0	gp	awaiting clinical code migration to emis web	telephone call to relative/carer	680
0		non-consultation medication data	repeat issue	679
0	nurse	awaiting clinical code migration to emis web	home visit note	672
0	other care provider	discussion with colleague	discussion with colleague	665
0		administration note	docman	665
0	gp	other note		661
0		awaiting clinical code migration to emis web	telephone consultation	656
0	other care provider	awaiting clinical code migration to emis web	scanned document	642
0		ooh report	third party consultation	637
0	gp	scanned document	externally entered	619
0	gp	medication requested	repeat issue	606
0	nurse	awaiting clinical code migration to emis web	telephone triage encounter	602
0	other care provider	awaiting clinical code migration to emis web	telephone call to a patient	541
0	other care provider	awaiting clinical code migration to emis web	face to face consultation	534
0	dr	administration note	administration note	525
0	gp	discussion with other professional	awaiting review	514
0	gp	administration note	awaiting review	509
0	other care provider	awaiting clinical code migration to emis web	mail to patient	507
0		ooh report	awaiting review	504
0	dr	awaiting clinical code migration to emis web	gp surgery	501
0		walk-in clinic		495
0		administration note	inbound document	493
0		routine consultation	repeat issue	488

For peer review only

0		awaiting clinical code migration to emis web	telephone call from a patient	486
0	gp	ooh report	out of hours, non practice	478
0	gp	mail from patient	mail from patient	473
0		other note	awaiting review	472
0	gp	awaiting clinical code migration to emis web	enterprise consultation	458
0	other care provider	repeat prescription	awaiting review	455
0	gp	awaiting clinical code migration to emis web	discussion with colleague	451
0		awaiting clinical code migration to emis web	outbound referral	450
0	gp	awaiting clinical code migration to emis web	nursing home	447
0	dr	awaiting clinical code migration to emis web	awaiting review	444
0	nurse	e-mail received from patient	e-mail received from patient	443
0		awaiting clinical code migration to emis web	data transferred from other system	442
0	nurse	ooh report	awaiting review	439
0		administration note	awaiting review	436
0	other care provider	clinic note	awaiting review	431
0	gp	administration note		415
0	gp	administration note	scanned document	414
0	gp	awaiting clinical code migration to emis web	urgent consultation	404
0	other care provider	awaiting clinical code migration to emis web	externally entered note	385
0		awaiting clinical code migration to emis web	school	384
0	nurse	hospital outpatient report	hospital outpatient report	380
0		mail to patient	patientchase insert	377
0		awaiting clinical code migration to emis web	telephone encounter	376
0	gp	administration note	results recording	372
0		discussion with colleague	discussion with colleague	368
0	other care provider	awaiting clinical code migration to emis web	results recording	365
0		case conference	awaiting review	361
0	other care provider	awaiting clinical code migration to emis web	telephone consultation	350
0	other care provider	awaiting clinical code migration to emis web	patientchase insert	345
0		sms text message sent to patient	patientchase insert	345
0	nurse	mail to patient	mail to patient	342
0		awaiting clinical code migration to emis web	hospital outpatient report	336
0		gp surgery		315
0	gp	ooh report	nhs direct report	314
0	gp	administration note	referral letter	313
0	dr	awaiting clinical code migration to emis web		310

For peer review only

0	gp	awaiting clinical code migration to emis web	telephone call from relative/carer	310
0	gp	awaiting clinical code migration to emis web	non-consultation data	309
0	nurse	awaiting clinical code migration to emis web	telephone call from a patient	309
0	other care provider	awaiting clinical code migration to emis web	non-consultation data	306
0	other care provider	e-mail received from patient	e-mail received from patient	292
0	other care provider	awaiting clinical code migration to emis web	non-consultation medication data	289
0	nurse	clinic note	awaiting review	286
0	dr	scanned document	scanned document	285
0		ooh report	out of hours, non practice	283
0	other care provider	laboratory result	laboratory result	282
0	gp	awaiting clinical code migration to emis web	discharge details	281
0		home visit note	home of patient	277
0	nurse	awaiting clinical code migration to emis web	inbound document	276
0	other care provider	hospital inpatient report	hospital inpatient report	275
0	gp	mail to patient	mail to patient	270
0	gp	awaiting clinical code migration to emis web	residential home visit note	266
0	other care provider	administration note	awaiting review	265
0		awaiting clinical code migration to emis web	acute visit	263
0		walk-in clinic	awaiting review	260
0	dr	hospital outpatient report	hospital outpatient report	256
0	gp	awaiting clinical code migration to emis web	mail to patient	249
0		administration note	mjog	248
0		mail from patient	mail from patient	248
0	gp	awaiting clinical code migration to emis web	letter from outpatients	247
0		awaiting clinical code migration to emis web	discharge details	244
0	nurse	awaiting clinical code migration to emis web	mail to patient	243
0	other care provider	awaiting clinical code migration to emis web	home visit note	236
0	gp	home visit note	results recording	235
0	dr	inbound document	inbound document	234
0	gp	awaiting clinical code migration to emis web	other note	230
0	gp	awaiting clinical code migration to emis web	patientchase insert	229
0	dr	awaiting clinical code migration to emis web	administration note	225
0	gp	awaiting clinical code migration to emis web	open door surgery	222

For peer review only

0		awaiting clinical code migration to emis web	nhs direct report	222
0	other care provider	awaiting clinical code migration to emis web	touchscreen	216
0	gp	scanned document	docman	213
0	nurse	awaiting clinical code migration to emis web	nursing home visit note	210
0	nurse	administration note		209
0	nurse	awaiting clinical code migration to emis web	externally entered note	209
0		awaiting clinical code migration to emis web	laboratory result	199
0		hospital outpatient report	hospital	197
0	other care provider	non-consultation medication data	repeat issue	196
0	other care provider	awaiting clinical code migration to emis web	referral letter	195
0	nurse	e-mail sent to patient	awaiting review	191
0	nurse	awaiting clinical code migration to emis web	referral letter	190
0	dr	awaiting clinical code migration to emis web	telephone consultation	186
0	nurse	case conference	awaiting review	185
0	other care provider	awaiting clinical code migration to emis web	data transferred from other system	184
0	gp	multidisciplinary team meeting without patient	awaiting review	179
0	gp	awaiting clinical code migration to emis web	clinic note	176
0	other care provider	awaiting clinical code migration to emis web	mjog	175
0		awaiting clinical code migration to emis web	hospital inpatient report	168
0		awaiting clinical code migration to emis web	home visit note	164
0		awaiting clinical code migration to emis web	online services message	163
0	other care provider	administration note		162
0	nurse	awaiting clinical code migration to emis web	outbound referral	159
0	other care provider	ooh report	awaiting review	156
0	gp	awaiting clinical code migration to emis web	non-consultation medication data	152
0	nurse	awaiting clinical code migration to emis web	telephone call to relative/carer	152
0	other care provider	other note	awaiting review	149
0	dr	awaiting clinical code migration to emis web	surgery consultation	148
0	nurse	awaiting clinical code migration to emis web	scanned document	146
0	gp	administration note	repeat issue	145
0		awaiting clinical code migration to emis web	open door surgery	144

For peer review only

0	other care provider	awaiting clinical code migration to emis web	clinic note	140
0		inbound referral	awaiting review	138
0		awaiting clinical code migration to emis web	clinic note	133
0		awaiting clinical code migration to emis web	face to face consultation	129
0	other care provider	non-consultation medication data	non-consultation medication data	124
0	nurse	awaiting clinical code migration to emis web	residential home visit note	122
0	nurse	awaiting clinical code migration to emis web	data transferred from other system	121
0	other care provider	e-mail sent to patient	awaiting review	121
0	gp	administration note	outbound referral	118
0	nurse	other note		117
0	nurse	awaiting clinical code migration to emis web	nurse telephone triage	116
0		awaiting clinical code migration to emis web	accident & emergency	116
0	gp	other consultation medium used	data transferred from other system	109
0		non-consultation medication data	non-consultation medication data	108
0	nurse	face to face consultation	triage	104
0	other care provider	awaiting clinical code migration to emis web	acute visit	102
0	nurse	awaiting clinical code migration to emis web	telephone call from relative/carer	100
0		extended hours consultation	out of hours, non practice	100
0	nurse	mail from patient	mail from patient	98
0	other care provider	hospital outpatient report	hospital	97
0	dr	awaiting clinical code migration to emis web	nursing home visit note	96
0		nurse telephone triage	nurse telephone triage	94
0	nurse	hospital inpatient report	hospital inpatient report	93
0	other care provider	case conference	awaiting review	92
0	gp	provision of general practitioner intermediate care	awaiting review	91
0	gp	walk-in clinic		91
0		hospital outpatient report	letter from outpatients	91
0	other care provider	other note		89
0	gp	administration note	other note	87
0	other care provider	medication requested	awaiting review	85
0	dr	laboratory result	results recording	84
0		awaiting clinical code migration to emis web	mail from patient	84
0		awaiting clinical code migration to emis web	health centre	80
0	gp	administration note	inbound document	76
0	gp	other note	awaiting review	76

For peer review only

0	nurse	walk-in clinic		76
0		other note	non-consultation medication data	76
0	other care provider	awaiting clinical code migration to emis web	nhs direct report	73
0		administration note	repeat issue	73
0	gp	ooh report	awaiting review	70
0	gp	weekly care home ward round	awaiting review	70
0		awaiting clinical code migration to emis web	medicine management	70
0	other care provider	awaiting clinical code migration to emis web	open door surgery	69
0	other care provider	awaiting clinical code migration to emis web	telephone encounter	69
0		medication requested	awaiting review	68
0	other care provider	awaiting clinical code migration to emis web	telephone call to relative/carer	65
0	dr	awaiting clinical code migration to emis web	repeat issue	64
0	gp	non-consultation medication data	repeat issue	64
0	other care provider	awaiting clinical code migration to emis web	telephone call from a patient	64
0	gp	clinic note	nhs direct report	62
0	gp	non-consultation medication data	medicine management	61
0	other care provider	awaiting clinical code migration to emis web	letter from outpatients	61
0	gp	hospital outpatient report	letter from outpatients	60
0	gp	non-consultation data	data transferred from other system	57
0	nurse	awaiting clinical code migration to emis web	enterprise consultation	57
0	gp	outbound referral	referral letter	55
0	other care provider	repeat prescription	medicine management	55
0	nurse	seen in influenza vaccination clinic	awaiting review	54
0		administration note	results recording	54
0		awaiting clinical code migration to emis web	telephone call from relative/carer	54
0	gp	awaiting clinical code migration to emis web	hospital outpatient report	53
0	gp	gp surgery		53
0	gp	third party consultation	out of hours, non practice	51
0		awaiting clinical code migration to emis web	telephone call to relative/carer	51
0		clinic note	out of hours, non practice	51
0		face to face consultation	primary care centre	51
0	gp	awaiting clinical code migration to emis web	laboratory result	50
0	nurse	non-consultation medication data	repeat issue	48
0		awaiting clinical code migration to emis web	other note	48
0		face to face consultation	triage	48
0	gp	progress report	nhs direct report	47
0	other care provider	ooh report	nhs direct report	47

For peer review only

0	nurse	awaiting clinical code migration to emis web	non-consultation data	46
0		awaiting clinical code migration to emis web	nursing home	46
0	gp	awaiting clinical code migration to emis web	mail from patient	45
0	nurse	awaiting clinical code migration to emis web	discussion with colleague	45
0		awaiting clinical code migration to emis web	e-mail received from patient	45
0	nurse	awaiting clinical code migration to emis web	medicine management	43
0	nurse	medication requested	repeat issue	42
0	dr	mail to patient	mail to patient	40
0	gp	awaiting clinical code migration to emis web	encompass message	39
0	gp	other note	data transferred from other system	39
0	nurse	awaiting clinical code migration to emis web	clinic note	39
0	nurse	awaiting clinical code migration to emis web	laboratory result	39
0	other care provider	awaiting clinical code migration to emis web	other note	39
0	other care provider	medication requested	repeat issue	38
0	gp	administration note	non-consultation data	37
0	other care provider	mail from patient	mail from patient	37
0	dr	awaiting clinical code migration to emis web	results recording	36
0	gp	awaiting clinical code migration to emis web	e-mail received from patient	36
0	nurse	awaiting clinical code migration to emis web	walk-in centre	36
0	other care provider	awaiting clinical code migration to emis web	gp2gp import	36
0		administration note	mail to patient	36
0	gp	awaiting clinical code migration to emis web	home of patient	35
0	gp	awaiting clinical code migration to emis web	casualty attendance	34
0	gp	inbound document	letter from outpatients	34
0	other care provider	awaiting clinical code migration to emis web	outbound referral	34
0	other care provider	extended hours consultation	out of hours, non practice	34
0	gp	awaiting clinical code migration to emis web	telephone encounter	33
0	gp	seen in hospital ward	awaiting review	33
0		other note	non-consultation data	33
0	nurse	other note	awaiting review	32
0	other care provider	awaiting clinical code migration to emis web	discussion with colleague	32
0		discussion with other professional	awaiting review	32

For peer review only

0		multidisciplinary team meeting without patient	awaiting review	32
0		children's home visit note		31
0	other care provider	laboratory result	results recording	30
0		awaiting clinical code migration to emis web	casualty attendance	30
0		extended hours consultation	nhs direct report	30
0	nurse	administration note	awaiting review	29
0	nurse	awaiting clinical code migration to emis web	letter from outpatients	29
0	nurse	awaiting clinical code migration to emis web	open door surgery	29
0	nurse	discussion with other professional	awaiting review	29
0	nurse	ooh report	out of hours, non practice	29
0		face to face consultation	treatment room	29
0	gp	email received from carer	awaiting review	28
0	other care provider	awaiting clinical code migration to emis web	telephone triage encounter	28
0		administration note	touchscreen	28
0	nurse	awaiting clinical code migration to emis web	encompass message	27
0	nurse	awaiting clinical code migration to emis web	nursing home	27
0		third party consultation	out of hours, non practice	27
0	gp	awaiting clinical code migration to emis web	follow-up/routine visit	26
0	other care provider	emergency consultation	accident & emergency	26
0	gp	hospital outpatient report	nhs direct report	25
0	other care provider	awaiting clinical code migration to emis web	telephone call from relative/carer	23
0	other care provider	outbound referral	referral letter	23
0		externally entered note	scanned document	23
0	nurse	multidisciplinary team meeting without patient	awaiting review	22
0		other consultation medium used	data transferred from other system	22
0	dr	awaiting clinical code migration to emis web	outbound referral	21
0	gp	seen in influenza vaccination clinic	awaiting review	21
0	nurse	awaiting clinical code migration to emis web	other note	21
0		ooh report	nhs direct report	21
0	dr	awaiting clinical code migration to emis web	inbound document	20
0	gp	awaiting clinical code migration to emis web	day case report	20
0	other care provider	seen by general practitioner with special interest in ear nose and throat disorders	data transferred from other system	20
0		emergency consultation	accident & emergency	20
0	gp	awaiting clinical code migration to emis web	online services message	19

For peer review only

0	gp	hospital outpatient report	third party consultation	19
0	nurse	repeat prescription	awaiting review	19
0		awaiting clinical code migration to emis web	walk in centre	19
0	dr	hospital inpatient report	hospital inpatient report	18
0	other care provider	awaiting clinical code migration to emis web	residential home visit note	18
0	other care provider	walk-in clinic		18
0		awaiting clinical code migration to emis web	residential home visit note	18
0		awaiting clinical code migration to emis web	telephone triage encounter	18
0	dr	outbound referral	outbound referral	17
0	gp	ooh report	third party consultation	17
0	gp	other consultation medium used	nhs direct report	17
0	gp	radiology result	awaiting review	17
0	gp	telephone consultation	telephone call from relative/carer	17
0	other care provider	awaiting clinical code migration to emis web	enterprise consultation	17
0	other care provider	walk-in clinic	walk in centre	17
0		administration note	referral letter	17
0	dr	other note	other note	16
0	other care provider	awaiting clinical code migration to emis web	nursing home visit note	16
0	dr	medication requested	repeat issue	15
0	other care provider	administration note	inbound document	15
0	other care provider	awaiting clinical code migration to emis web	out of hours, non practice	15
0		non-consultation data	data transferred from other system	15
0	other care provider	face to face consultation	triage	15
0	dr	awaiting clinical code migration to emis web	telephone call to a patient	14
0	dr	externally entered note	externally entered note	14
0	gp	hospital inpatient report	discharge details	14
0	gp	seen by general practitioner with special interest in ear nose and throat disorders	data transferred from other system	14
0	other care provider	administration note	scanned document	14
0	other care provider	discussion with other professional	awaiting review	14
0		awaiting clinical code migration to emis web	out of hours, practice	14
0		externally entered note		14
0	dr	telephone call to relative/carer	telephone call to relative/carer	13
0	nurse	awaiting clinical code migration to emis web	nhs direct report	13
0	nurse	awaiting clinical code migration to emis web	out of hours, non practice	13
0		hospital inpatient note	awaiting review	13

0		repeat prescription	awaiting review	13
0	gp	other note	encompass message	12
0	nurse	awaiting clinical code migration to emis web	hospital outpatient report	12
0	other care provider	awaiting clinical code migration to emis web	accident & emergency	12
0	nurse	other note	encompass message	11
0	nurse	repeat prescription	repeat issue	11
0	other care provider	seen in influenza vaccination clinic	awaiting review	11
0		awaiting clinical code migration to emis web	discussion with colleague	11
0		awaiting clinical code migration to emis web	hospital outpatient consultation	11
0		awaiting clinical code migration to emis web	walk-in clinic	11
0		non-consultation medication data	medicine management	11
0		provision of general practitioner intermediate care	awaiting review	10
0	dr	clinic note	awaiting review	< 10
0	other care provider	ooh report	out of hours centre	< 10
0		administration note	non-consultation data	< 10
0		clinic note	nhs direct report	< 10
0		telephone follow-up	awaiting review	< 10
0	gp	administration note	clinic note	< 10
0	gp	progress report	awaiting review	< 10
0	nurse	awaiting clinical code migration to emis web	acute visit	< 10
0	other care provider	multidisciplinary team meeting without patient	awaiting review	< 10
0		administration note	other note	< 10
0	dr	awaiting clinical code migration to emis web	home visit note	< 10
0	gp	hospital inpatient note	day case report	< 10
0	gp	inbound document	discharge details	< 10
0	nurse	awaiting clinical code migration to emis web	follow-up/routine visit	< 10
0	other care provider	awaiting clinical code migration to emis web	discharge details	< 10
0		awaiting clinical code migration to emis web	enterprise consultation	< 10
0		awaiting clinical code migration to emis web	nursing home visit note	< 10
0		email received from carer	awaiting review	< 10
0		ooh report	out of hours, practice	< 10
0		other note	data transferred from other system	< 10
0		outbound referral	referral letter	< 10
0		radiology result	awaiting review	< 10
0	dr	gp surgery	awaiting review	< 10
0	gp	routine consultation	repeat issue	< 10
0	nurse	email received from carer	awaiting review	< 10
0	nurse	non-consultation data	data transferred from other system	< 10

For peer review only

0	nurse	scanned document	docman	< 10
0	other care provider	awaiting clinical code migration to emis web	home of patient	< 10
0	other care provider	hospital outpatient report	letter from outpatients	< 10
0	dr	discussion with colleague	discussion with colleague	< 10
0	gp	administration note	discussion with colleague	< 10
0	gp	awaiting clinical code migration to emis web	out of hours, practice	< 10
0	gp	clinic note	out of hours, non practice	< 10
0	gp	ooh report	out of hours, practice	< 10
0	gp	seen in diabetic clinic	awaiting review	< 10
0	gp	sms text message sent to patient	awaiting review	< 10
0	nurse	administration note	discussion with colleague	< 10
0	other care provider	awaiting clinical code migration to emis web	nursing home	< 10
0	other care provider	awaiting clinical code migration to emis web	online services message	< 10
0		awaiting clinical code migration to emis web	bulk operation	< 10
0		non-consultation data	touchscreen	< 10
0	dr	awaiting clinical code migration to emis web	hospital outpatient report	< 10
0	dr	awaiting clinical code migration to emis web	other	< 10
0	dr	awaiting clinical code migration to emis web	third party consultation	< 10
0	dr	clinic note	clinic note	< 10
0	dr	non-consultation data	non-consultation data	< 10
0	gp	awaiting clinical code migration to emis web	hospital inpatient report	< 10
0	gp	clinic note	community clinic	< 10
0	nurse	administration note	non-consultation data	< 10
0	nurse	awaiting clinical code migration to emis web	non-consultation medication data	< 10
0	other care provider	externally entered note	data transferred from other system	< 10
0	other care provider	gp surgery		< 10
0	other care provider	other note	referral letter	< 10
0		awaiting clinical code migration to emis web	nurseries/playgroup	< 10
0		hospital outpatient report	third party consultation	< 10
0	gp	nurse telephone triage	nurse telephone triage	< 10
0	dr	awaiting clinical code migration to emis web	discussion with colleague	< 10
0	dr	awaiting clinical code migration to emis web	medicine management	< 10
0	dr	awaiting clinical code migration to emis web	residential home visit note	< 10
0	dr	e-mail received from patient	e-mail received from patient	< 10
0	dr	telephone call from relative/carer	telephone call from relative/carer	< 10

For peer review only

0	gp	awaiting clinical code migration to emis web	walk-in centre	< 10
0	gp	extended hours consultation	out of hours, non practice	< 10
0	gp	externally entered note		< 10
0	gp	other consultation medium used	other note	< 10
0	gp	telephone consultation	telephone call to relative/carer	< 10
0	nurse	awaiting clinical code migration to emis web	telephone encounter	< 10
0	nurse	medication requested	awaiting review	< 10
0	nurse	telephone follow-up	awaiting review	< 10
0	other care provider	awaiting clinical code migration to emis web	e-mail received from patient	< 10
0	other care provider	awaiting clinical code migration to emis web	mail from patient	< 10
0	other care provider	awaiting clinical code migration to emis web	out of hours, practice	< 10
0	other care provider	other note	data transferred from other system	< 10
0		awaiting clinical code migration to emis web	day case report	< 10
0		awaiting clinical code migration to emis web	encompass message	< 10
0		e-mail encounter to carer	awaiting review	< 10
0		walk-in clinic	walk in centre	< 10
0	dr	awaiting clinical code migration to emis web	non-consultation data	< 10
0	dr	awaiting clinical code migration to emis web	other note	< 10
0	dr	e-mail sent to patient	awaiting review	< 10
0	gp	email received from third party	awaiting review	< 10
0	gp	hospital inpatient note	awaiting review	< 10
0	gp	night visit note	night visit	< 10
0	gp	other note	third party consultation	< 10
0	gp	telephone encounter	telephone call to relative/carer	< 10
0	gp	telephone follow-up	awaiting review	< 10
0	nurse	administration note	referral letter	< 10
0	nurse	awaiting clinical code migration to emis web	urgent consultation	< 10
0	nurse	seen in asthma clinic	awaiting review	< 10
0	nurse	seen in hospital ward	awaiting review	< 10
0	nurse	telephone consultation	telephone call to relative/carer	< 10
0	other care provider	administration note	other note	< 10
0	other care provider	administration note	referral letter	< 10
0	other care provider	administration note	repeat issue	< 10
0	other care provider	administration note	results recording	< 10
0	other care provider	awaiting clinical code migration to emis web	casualty attendance	< 10
0	other care provider	awaiting clinical code migration to emis web	diabetic clinic	< 10

For peer review only

0	other care provider	awaiting clinical code migration to emis web	encompass message	< 10
0	other care provider	clinic note	nhs direct report	< 10
0	other care provider	seen in diabetic clinic	awaiting review	< 10
0		awaiting clinical code migration to emis web	other report	< 10
0		child in need meeting	awaiting review	< 10
0		hospital inpatient report	hospital inpatient	< 10
0		progress report	nhs direct report	< 10
0		repeat prescription	non-consultation medication data	< 10
0		weekly care home ward round	awaiting review	< 10
0	dr	awaiting clinical code migration to emis web	non-consultation medication data	< 10
0	dr	awaiting clinical code migration to emis web	telephone call from a patient	< 10
0	dr	awaiting clinical code migration to emis web	telephone call from relative/carer	< 10
0	dr	awaiting clinical code migration to emis web	telephone call to relative/carer	< 10
0	gp	awaiting clinical code migration to emis web	night visit , practice	< 10
0	gp	awaiting clinical code migration to emis web	walk-in clinic	< 10
0	gp	non-consultation medication data	non-consultation medication data	< 10
0	gp	other consultation medium used	referral letter	< 10
0	gp	other note	non-consultation data	< 10
0	gp	other note	non-consultation medication data	< 10
0	gp	seen in baby clinic	awaiting review	< 10
0	gp	telephone encounter	telephone call from relative/carer	< 10
0	nurse	administration note	clinic note	< 10
0	nurse	administration note	laboratory result	< 10
0	nurse	awaiting clinical code migration to emis web	casualty attendance	< 10
0	nurse	awaiting clinical code migration to emis web	patientchase insert	< 10
0	nurse	clinic note	community clinic	< 10
0	nurse	extended hours consultation	out of hours, non practice	< 10
0	nurse	externally entered note	data transferred from other system	< 10
0	nurse	first attendance face to face	awaiting review	< 10
0	nurse	hospital inpatient note	awaiting review	< 10
0	nurse	ooh report	out of hours, practice	< 10
0	nurse	other consultation medium used	data transferred from other system	< 10
0	nurse	other note	data transferred from other system	< 10
0	nurse	other note	referral letter	< 10
0	nurse	outbound referral	referral letter	< 10
0	nurse	patient initiated enc. nos	awaiting review	< 10
0	nurse	telephone consultation	telephone call from relative/carer	< 10
0	nurse	telephone encounter	telephone call to relative/carer	< 10
0	nurse	weekly care home ward round	awaiting review	< 10

For peer review only

0	other care provider	administration note	mail to patient	< 10
0	other care provider	awaiting clinical code migration to emis web	hospital inpatient report	< 10
0	other care provider	awaiting clinical code migration to emis web	hospital outpatient report	< 10
0	other care provider	awaiting clinical code migration to emis web	laboratory result	< 10
0	other care provider	clinic note	community clinic	< 10
0	other care provider	first attendance face to face	awaiting review	< 10
0	other care provider	inbound referral	awaiting review	< 10
0	other care provider	non-consultation data	data transferred from other system	< 10
0	other care provider	ooh report	out of hours, non practice	< 10
0	other care provider	telephone encounter	awaiting review	< 10
0		awaiting clinical code migration to emis web	follow-up/routine visit	< 10
0		awaiting clinical code migration to emis web	letter	< 10
0		awaiting clinical code migration to emis web	minor injuries unit	< 10
0		awaiting clinical code migration to emis web	secretary	< 10
0		email received from third party	awaiting review	< 10
0		gp surgery	non-consultation data	< 10
0		hospital inpatient report	awaiting review	< 10
0		hospital inpatient report	discharge details	< 10
0		multidisciplinary team meeting with patient	awaiting review	< 10
0		night visit note	night visit, local rota	< 10
0		provision of general practitioner intermediate care	gp2gp import	< 10
0		telephone triage encounter	nhs direct report	< 10
0		third party consultation	third party	< 10
0	other care provider	nurse telephone triage	nurse telephone triage	< 10

For peer review only

Annex 3: *NHS targets for cancer wait times*

			Operational standard
Maximum two weeks from	Receipt of urgent referral for suspected cancer to first outpatient attendance		93%
	Receipt of referral of any patient with breast symptoms (where cancer not suspected) to first hospital assessment		93%
Maximum 28 days from	Receipt of two week wait referral for suspected cancer, receipt of urgent referral from a cancer screening programme (breast, bowel, cervical), and receipt of two week wait referral of any patient with breast symptoms (where cancer not suspected), to the date the patient is informed of a diagnosis or ruling out of cancer		75%
Maximum one month (31 days) from:	Decision to treat to first definitive treatment		96%
	Decision to treat/earliest clinically appropriate date to start of second or subsequent treatment(s) for all cancer patients including those diagnosed with a recurrence where the subsequent treatment is:	surgery	94%
		drug treatment	98%
		radiotherapy	94%
Maximum two months (62 days) from:	Urgent referral for suspected cancer to first treatment (62-day classic)		85%
	Urgent referral from a NHS Cancer Screening Programme (breast, cervical or bowel) for suspected cancer to first treatment		90%

Annex 4: Comparing Cancer Wait Times counts for referrals and first treatments with the NCRAS data equity pack

Table A4.1: Comparing Cancer Wait Times counts for referrals and first treatments with the NCRAS data equity pack

	01 Apr 2020 - 31 Jan 2021					
	First treatments for new cancer			Urgent cancer referrals		
	Observed	Expected	Percentage change (95% CI)	Observed	Expected	Percentage change (95% CI)
Cancer wait times data						
All	224,323	267,946	-16.3% (-16.6, -15.9)	1,673,775	2,071,967	-19.2% (-19.3, -19.1)
NCRAS data equity pack						
All	219,410	254,436	-13.8% (-14.3, -13.3)	1,658,309	1,984,489	-16.4% (-16.6, -16.3)
Breast	30,488	40,530	-24.8% (-25.9, -23.6)	337,582	363,139	-7% (-7.5, -6.6)
Gynaecological	11,281	12,344	-8.6% (-10.9, -6.2)	158,723	176,985	-10.3% (-10.9, -9.7)
Head and Neck	8,892	9,901	-10.2% (-12.7, -7.6)	163,668	189,837	-13.8% (-14.4, -13.2)
Lower GI	23,507	27,056	-13.1% (-14.6, -11.6)	302,369	366,677	-17.5% (-17.9, -17.1)
Lung	24,796	27,409	-9.5% (-11.1, -8.0)	33,830	53,641	-36.9% (-37.8, -36.1)
Skin	40,977	43,475	-5.7% (-7.0, -4.5)	338,172	429,802	-21.3% (-21.7, -21.0)
Upper GI	17,059	17,586	-3% (-5.0, -0.9)	141,720	163,013	-13.1% (-13.7, -12.4)
Urological	37,970	50,056	-24.1% (-25.2, -23.1)	134,389	184,642	-27.2% (-27.7, -26.7)
All other	24,441	26,080	-6.3% (-7.9, -4.6)	47,856	56,753	-15.7% (-16.7, -14.6)

For peer review only

Annex 5: Patient demographics and person-time and total numbers of observed activity in CPRD Aurum sample

Table A5.1: Patient demographics in CPRD Aurum sample as at 22 March 2020

	Patient count as at 22 March 2020	
	n	%
All	375,501	
Female	187,509	49.9%
Male	187,992	50.1%
Under 11	39,611	10.5%
11 to 19	43,406	11.6%
20 to 49	157,962	42.1%
50 to 69	87,482	23.3%
70 and older	47,040	12.5%
IMD Quintile - 1	78,759	21.0%
IMD Quintile - 2	73,046	19.5%
IMD Quintile - 3	71,840	19.1%
IMD Quintile - 4	77,545	20.7%
IMD Quintile - 5	74,020	19.7%
IMD not recorded	291	0.1%

Table A5.2: Person time (weeks) and total primary care activity analysed - CPRD Aurum

	3 January 2016 - 21March 2020*				22 March 2020 - 30 January 2021			
	Patient-time (weeks)	100,000 person-months	Activity count	Observed rate per 100,000 person-months	Patient-time (weeks)	100,000 person-months	Activity count	Observed rate per 100,000 person-months
Consultations excl flu vaccinations	78,868,977	181.44	6,912,079	38,095	16,701,707	38.19	1,306,206	34,201
Routine Referrals	78,868,977	181.44	312,422	1,722	16,701,707	38.19	40,744	1,067
2 Week Wait Referrals	78,868,977	181.44	38,905	214	16,701,707	38.19	10,235	268

**The pre-pandemic period consists of 220 weeks or 51.61 months, the post pandemic period is 45 weeks or 10.29 months*

For peer review only

Annex 6: Observed vs expected appointment and cancer diagnosis counts from CWT data from 01 Jan 2019 (per person-month)

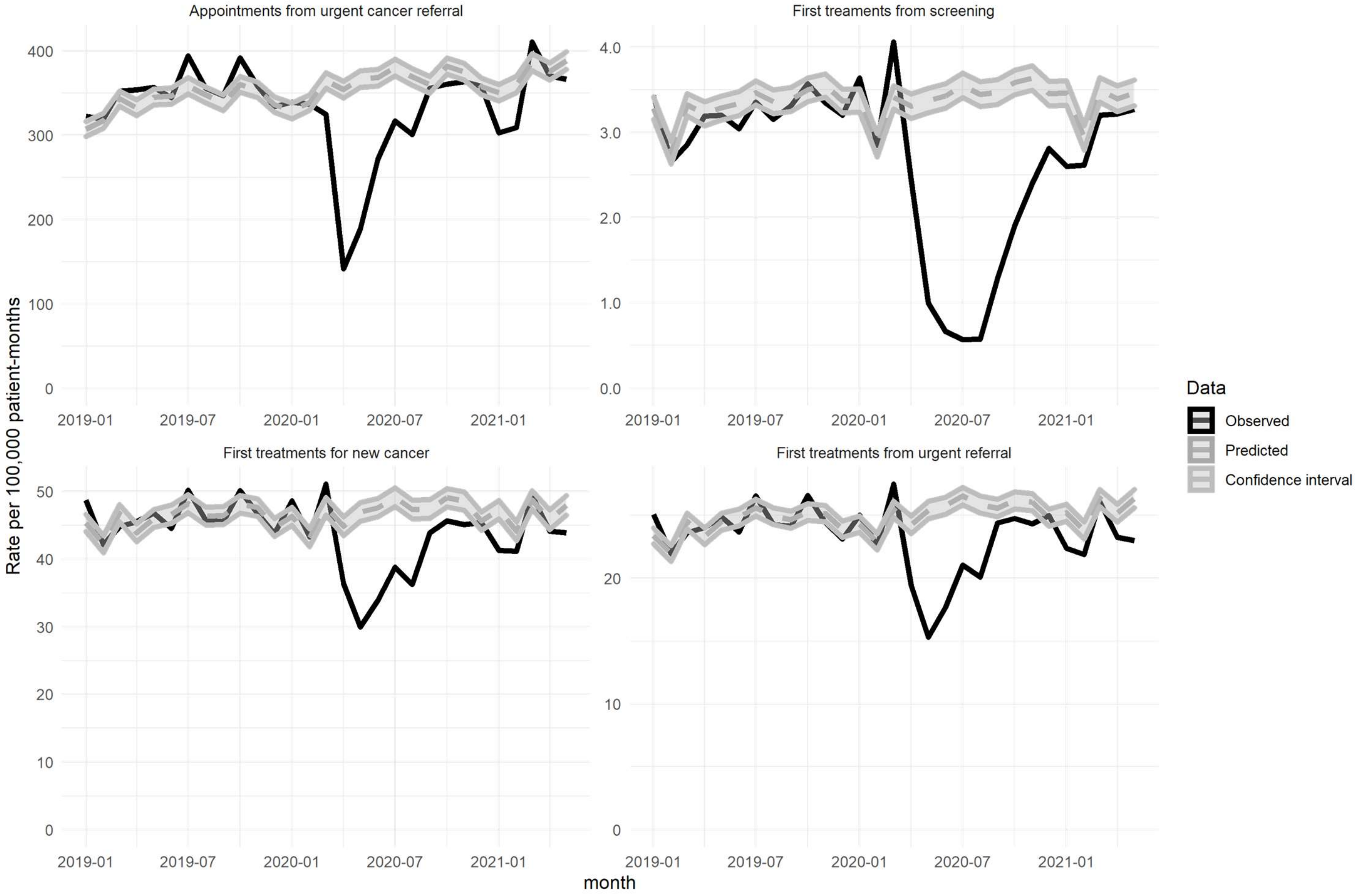

For peer review only

Annex 7: Observed vs expected consultations by IMD quintile

Figure A7: Observed vs expected consultations per person per week by IMD quintile, age-standardised (01 Jan 2019 – 30 Jan 2021)

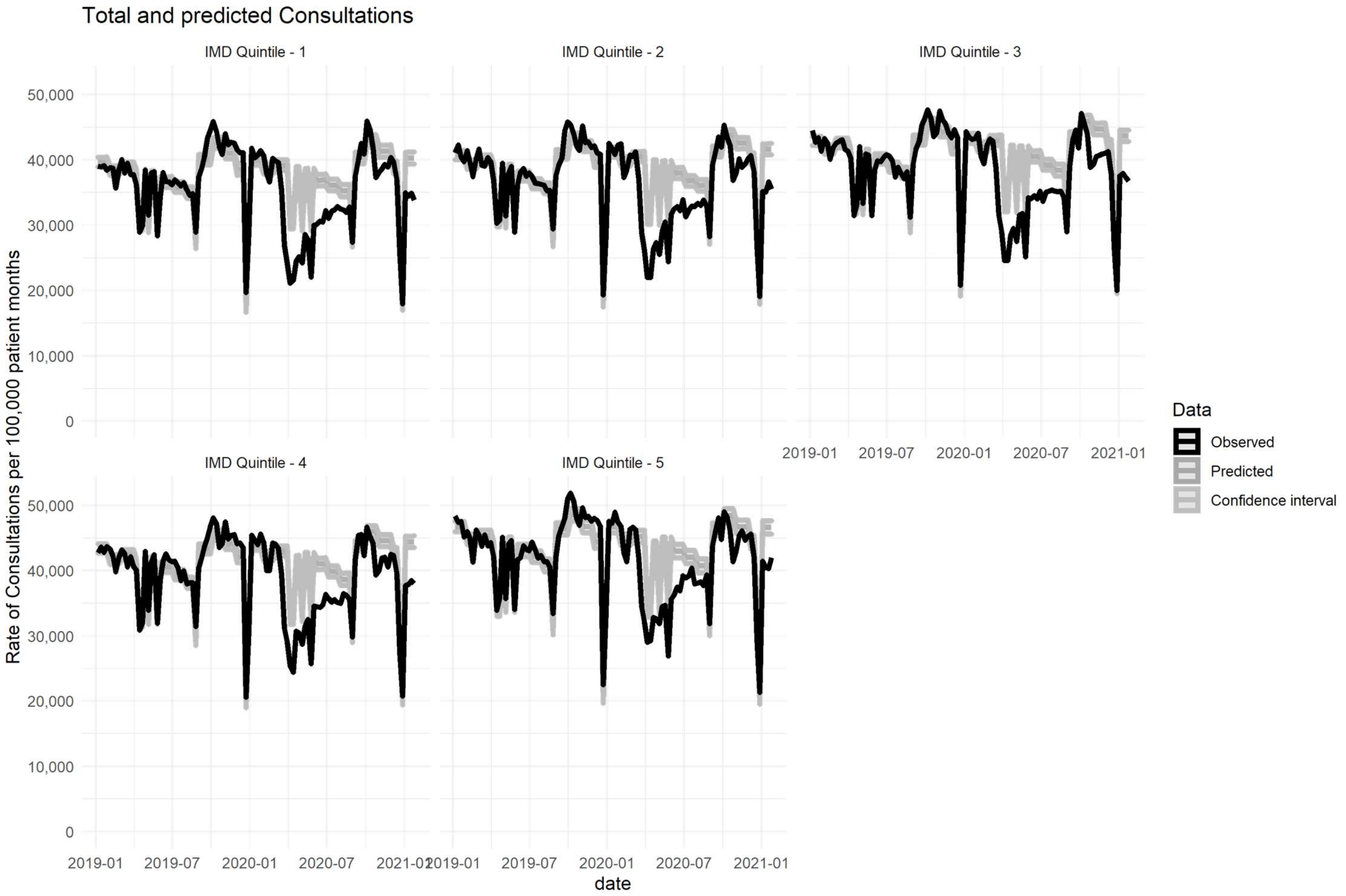

STROBE Statement—checklist of items that should be included in reports of observational studies

	Item No	Recommendation	Page No
**Title and abstract**	1	(*a*) Indicate the study's design with a commonly used term in the title or the abstract	2
		(*b*) Provide in the abstract an informative and balanced summary of what was done and what was found	2
**Introduction**			
Background/rationale	2	Explain the scientific background and rationale for the investigation being reported	3
Objectives	3	State specific objectives, including any prespecified hypotheses	3
**Methods**			
Study design	4	Present key elements of study design early in the paper	4
Setting	5	Describe the setting, locations, and relevant dates, including periods of recruitment, exposure, follow-up, and data collection	4
Participants	6	(*a*) *Cohort study*—Give the eligibility criteria, and the sources and methods of selection of participants. Describe methods of follow-up<br>*Case-control study*—Give the eligibility criteria, and the sources and methods of case ascertainment and control selection. Give the rationale for the choice of cases and controls<br>*Cross-sectional study*—Give the eligibility criteria, and the sources and methods of selection of participants	4
		(*b*) *Cohort study*—For matched studies, give matching criteria and number of exposed and unexposed<br>*Case-control study*—For matched studies, give matching criteria and the number of controls per case	
Variables	7	Clearly define all outcomes, exposures, predictors, potential confounders, and effect modifiers. Give diagnostic criteria, if applicable	4/5
Data sources/ measurement	8*	For each variable of interest, give sources of data and details of methods of assessment (measurement). Describe comparability of assessment methods if there is more than one group	*4/5*
Bias	9	Describe any efforts to address potential sources of bias	5/6
Study size	10	Explain how the study size was arrived at	4
Quantitative variables	11	Explain how quantitative variables were handled in the analyses. If applicable, describe which groupings were chosen and why	4-6
Statistical methods	12	(*a*) Describe all statistical methods, including those used to control for confounding	5/6
		(*b*) Describe any methods used to examine subgroups and interactions	5/6
		(*c*) Explain how missing data were addressed	4-6
		(*d*) *Cohort study*—If applicable, explain how loss to follow-up was addressed<br>*Case-control study*—If applicable, explain how matching of cases and controls was addressed<br>*Cross-sectional study*—If applicable, describe analytical methods taking account of sampling strategy	N/A
		(*e*) Describe any sensitivity analyses	4-6

Continued on next page

For peer review only - http://bmjopen.bmj.com/site/about/guidelines.xhtml

**Results**			
Participants	13*	(a) Report numbers of individuals at each stage of study—eg numbers potentially eligible, examined for eligibility, confirmed eligible, included in the study, completing follow-up, and analysed	4/5
		(b) Give reasons for non-participation at each stage	N/A
		(c) Consider use of a flow diagram	N/A
Descriptive data	14*	(a) Give characteristics of study participants (eg demographic, clinical, social) and information on exposures and potential confounders	6-8
		(b) Indicate number of participants with missing data for each variable of interest	6-8
		(c) *Cohort study*—Summarise follow-up time (eg, average and total amount)	N/A
Outcome data	15*	*Cohort study*—Report numbers of outcome events or summary measures over time	*6/7*
		Case-control study—Report numbers in each exposure category, or summary measures of exposure	*N/A*
		Cross-sectional study—Report numbers of outcome events or summary measures	*N/A*
Main results	16	(*a*) Give unadjusted estimates and, if applicable, confounder-adjusted estimates and their precision (eg, 95% confidence interval). Make clear which confounders were adjusted for and why they were included	6-8
		(*b*) Report category boundaries when continuous variables were categorized	N/A
		(*c*) If relevant, consider translating estimates of relative risk into absolute risk for a meaningful time period	N/A
Other analyses	17	Report other analyses done—eg analyses of subgroups and interactions, and sensitivity analyses	6-8
**Discussion**			
Key results	18	Summarise key results with reference to study objectives	9
Limitations	19	Discuss limitations of the study, taking into account sources of potential bias or imprecision. Discuss both direction and magnitude of any potential bias	9/10
Interpretation	20	Give a cautious overall interpretation of results considering objectives, limitations, multiplicity of analyses, results from similar studies, and other relevant evidence	10
Generalisability	21	Discuss the generalisability (external validity) of the study results	9/10
**Other information**			
Funding	22	Give the source of funding and the role of the funders for the present study and, if applicable, for the original study on which the present article is based	1

*Give information separately for cases and controls in case-control studies and, if applicable, for exposed and unexposed groups in cohort and cross-sectional studies.

Note: An Explanation and Elaboration article discusses each checklist item and gives methodological background and published examples of transparent reporting. The STROBE checklist is best used in conjunction with this article (freely available on the Web sites of PLoS Medicine at http://www.plosmedicine.org/, Annals of Internal Medicine at http://www.annals.org/, and Epidemiology at http://www.epidem.com/). Information on the STROBE Initiative is available at www.strobe-statement.org.

For peer review only

BMJ Open

Primary Care and Cancer: an analysis of the impact and inequalities of the COVID-19 pandemic on patient pathways

Journal:	*BMJ Open*
Manuscript ID	bmjopen-2021-059374.R2
Article Type:	Original research
Date Submitted by the Author:	18-Feb-2022
Complete List of Authors:	Watt, Toby; The Health Foundation, REAL Centre<br>Sullivan, Richard; King's College London<br>Aggarwal, Ajay; London School of Hygiene and Tropical Medicine Faculty of Public Health and Policy, Health Services Research & Policy
**Primary Subject Heading**:	Oncology
Secondary Subject Heading:	General practice / Family practice
Keywords:	ONCOLOGY, PRIMARY CARE, COVID-19

SCHOLARONE™
Manuscripts

I, the Submitting Author has the right to grant and does grant on behalf of all authors of the Work (as defined in the below author licence), an exclusive licence and/or a non-exclusive licence for contributions from authors who are: i) UK Crown employees; ii) where BMJ has agreed a CC-BY licence shall apply, and/or iii) in accordance with the terms applicable for US Federal Government officers or employees acting as part of their official duties; on a worldwide, perpetual, irrevocable, royalty-free basis to BMJ Publishing Group Ltd ("BMJ") its licensees and where the relevant Journal is co-owned by BMJ to the co-owners of the Journal, to publish the Work in this journal and any other BMJ products and to exploit all rights, as set out in our licence.

The Submitting Author accepts and understands that any supply made under these terms is made by BMJ to the Submitting Author unless you are acting as an employee on behalf of your employer or a postgraduate student of an affiliated institution which is paying any applicable article publishing charge ("APC") for Open Access articles. Where the Submitting Author wishes to make the Work available on an Open Access basis (and intends to pay the relevant APC), the terms of reuse of such Open Access shall be governed by a Creative Commons licence – details of these licences and which Creative Commons licence will apply to this Work are set out in our licence referred to above.

Other than as permitted in any relevant BMJ Author's Self Archiving Policies, I confirm this Work has not been accepted for publication elsewhere, is not being considered for publication elsewhere and does not duplicate material already published. I confirm all authors consent to publication of this Work and authorise the granting of this licence.

For peer review only

Primary Care and Cancer: an analysis of the impact and inequalities of the COVID-19 pandemic on patient pathways

Authors: Toby Watt[1,2], Richard Sullivan[3] and Ajay Aggarwal[4]

1. The Health Foundation, 8 Salisbury Sq., London, UK,
2. Department of Public Health, Environments and Society, London School of Hygiene and Tropical Medicine, 15-17 Tavistock Place, London, UK
3, School of Cancer and Pharmaceutical Sciences, King's College London, London, UK; Institute of Cancer Policy, King's College London, London, UK; Department of Oncology, Guy's and St Thomas' NHS Foundation Trust, London, UK.
4. Department of Health Services Research and Policy, London School of Hygiene & Tropical Medicine, London, UK; Institute of Cancer Policy, King's College London, London, UK; Department of Oncology, Guy's and St Thomas' NHS Foundation Trust, London, UK.

Corresponding author: Toby Watt, toby.watt@health.org.uk

Word count: 4,817

For peer review only

Abstract

Objectives

We explore the routes to cancer diagnosis to further undertanding of the inequality in the reduction in detection of new cancers since the start of the pandemic. We use different data sets to assess stages in the cancer pathway: primary care data for primary care consultations, routine and urgent referrals and published analysis of cancer registry data for appointments and first treatments.

Setting

Primary and cancer care

Participants

In this study we combine multiple data sets to perform a population-based cohort study on different areas of the cancer pathway. For primary care analysis, we use a random sample of 500,000 patients from the Clinical Practice Research Datalink (CPRD). Post-referral we perform a secondary data analysis on the Cancer Wait Times data and the National Cancer Registry Analysis Service (NCRAS) COVID-19 data equity pack.

Outcome measures:

Primary care: consultation, urgent cancer referral and routine referral rates, then appointments following an urgent cancer referral, and first treatments for new cancer, for all and by quintile of patient's local area Index of Multiple Deprivation.

Results

Primary care contacts and urgent cancer referrals fell by 12.4% (12.3 to 12.6; 95% CI) and 20.2% (18.1 to 22.3; 95% CI) respectively, while routine referrals have not recovered to pre-pandemic levels. Reductions in first treatments for newly diagnosed cancers are down 16.3% (15.9 to 16.6; 95% CI). The reduction in the number of two week wait referrals and first treatments for all cancer has been largest for those living in poorer areas, despite having a smaller reduction in primary care contact.

Conclusions

Our results further evidence the strain on primary care and the presence of the inverse care law, and the dire need to address the inequalities so sharply brought into focus by the pandemic. We need to address the disconnect between the importance we place on the role of primary care and the resources we devote to it.

Key Words: Oncology, Primary Care, COVID-19

Strengths and Limitations

- This study draws from multiple data sets along the complex, multidisciplinary cancer pathway.
- We use a rich primary care data set containing patient level primary care activity linked to patients' local area socioeconomic indicator.
- Our primary care patient sample is relatively small (500,000 active patients from January 2016 to January 2021), however the data produces results that closely mirror the rates of consultation and urgent cancer referral per patient produced in publicly available national data sets.

For peer review only

INTRODUCTION

The COVID-19 pandemic has had a profound impact on UK's health system. Each part of the UK's National Health Service has been impacted in different ways, and we are still feeling many of the consequences of both the COVID-19 pandemic and the public health measures put in place to manage it (non-pharmaceutical interventions; NPI). Cancer is one of the most complicated diseases that the UK health system must manage, being responsible for over one in four UK deaths in 2019. Cancer outcomes are acutely sensitive to changes in social determinants, patient pathways and service provision. Delays in both diagnosis and treatment have significant impacts on patient outcomes(1,2). Pandemic related diagnostic delays, lack of capacity and downstream stage progression (to more advanced disease) are already being seen(3). In addition, the impact of the pandemic needs to be seen in the context of an already overstretched UK cancer care system pre-pandemic that was 'burning hot' even in normal times(4).

Primary care sits at the heart of the cancer patient pathway and is the most crucial interface for early diagnosis and referral to hospital-based care, in addition to their wider support of cancer patient undergoing and post treatment. As models of cancer care have evolved in light of both technical advances and an ageing co-morbid population primary care has become an increasingly important aspect of integrated cancer care and an expansion of General Practitioner (GP) roles in cancer care(5). On average 22.5% of patients diagnosed with cancer are referred to oncology diagnostic services from primary care, but this reflects wide site-specific variation from as little as 8.3% of breast cancer to 42% for bladder cancer(6).

It is important to reflect that prior to the start of the COVID-19 pandemic primary care had seen significant declines in overall resourcing relative to the funding of the rest of the NHS and compared to growing levels of disease burden that is managed in primary care. In addition, there is growing evidence that primary care has been under greater pressure in more deprived areas, with higher levels of staff turnover(7), higher levels of complex multimorbidity(8), higher numbers of consultations(9) and lower levels of funding and fewer GPs per capita once levels of ill health are taken into account(10). These pressures on primary care, and a desire to correct them, have been recognised in the NHS Long Term Plan(11).

Thus, to understand the COVID-19's impact on primary care and the downstream impact on cancer outcomes we need to see that the pandemic arrived when the system that was already struggling to cope. Prior to COVID-19, the central role of primary care as agents of change in reducing inequalities had been the subject of much debate yet could do little in the face of political avoidance of health equity(12). Primary care had become a mirror on inequalities but also subject to significant pressures from these growing inequalities that had put practices in deprived populations under significant stress. Yet despite this, equity-oriented primary care reform in England in the mid-to-late 2000s may have helped to reduce socioeconomic inequality in health(13).

[Box]

COVID-19 was officially declared a pandemic by the WHO on 11 March 2020, and the UK announced its first full lockdown on 23rd March. In the following months UK NPI were eased, schools reopened in phases, non-essential shops reopened and in August the population were encouraged to eat out. Some restrictions were re-imposed in September and October, on the 5th of November 2020 a second brief national locked lasted until 2nd December. On the 6th of January a third national lockdown was introduced(14).

[End box]

It is now clear that the UK experience of the pandemic was one of the worst in the world, both in terms of excess mortality (both COVID-19 and non-COVID-19) and the impact of NPI (lockdowns) on both the ability of health services to continue provide care and the impact of messaging (stay at home) on patients' timely presentation for care(15). However, the overwhelming focus of impact studies on cancer care has been on hospital-based services, including diagnostics. Given primary care's central role in pathways to diagnosis and integrated cancer care, including survivorship there has been little insight around how overall changes in consultation rates impacted both routine and two week wait referrals as well as how this varied both in terms of site-specific cancers and as a consequence of socio-economic

For peer review only - http://bmjopen.bmj.com/site/about/guidelines.xhtml

inequalities. This study aimed to analyse the socioeconomic inequalities in the impact of NPI measures taken in response to COVID-19 on consultations and routine and urgent cancer referrals in primary care and cancer diagnosis in secondary care.

METHODS

Study design, data sources and participants

We perform a population-based cohort study using the following three separate sources.

Primary care data – CPRD Aurum

Primary care electronic health records were obtained from the Clinical Practice Research Datalink Aurum database (henceforth CPRD). We included patient records from 01 January 2016 to 31 January 2021. Pre-pandemic data were included to establish long-term trends and patterns of seasonality in primary care use and referrals to secondary care. Similar to recent analysis of the COVID-19 pandemic(16), our analysis focusses on comparing observed levels of activity to the expected following the introduction of NPI in England in March 2020.

CPRD contains anonymised patient primary care data from approximately 7% of the UK population and is broadly representative in terms of age, sex, and ethnicity(17). The patient records include information on consultations, patient demographic information, diagnoses, medication prescriptions, and referrals to secondary care.

The period of eligibility for study inclusion, which starts on the latest of the study start date (01/01/2016) or the patient's registration to their practice. A patient's period of eligibility ends on the earliest of leaving their practice, the end of data collection from their practice or their death. Primary care records from CPRD were linked to the deciled index of multiple deprivation (IMD) from 2015[i](18) of each patient's lower layer super output area (LSOA[ii]). 500,000 patients were randomly sampled from the CPRD population in England who were eligible for linkage within the defined study period.

Cancer wait times data

Cancer waiting times (CWT) measure performance against the NHS Constitution Standards, recording the number of patients screened, referred to oncology specialists, diagnosed and treated for cancer. These measures are used by local and national organisations to monitor the timely delivery of services to patients, they are published quarterly by NHS Digital[iii].

Cancer diagnosis by socioeconomic status - NCRAS Cancer equity data

Data on cancer diagnosis by socioeconomic group was drawn from CADEAS and National Cancer Registry Analysis Service (NCRAS) which have two published data sets(19), presenting the latest national data on:

i. The number of urgent suspected two-week wait referrals[iv] and,
ii. First definitive treatments for cancer[v].

---

[i] https://www.gov.uk/government/statistics/english-indices-of-deprivation-2015
[ii] Geographic areas in England and Wales that are built from groups of contiguous Output Areas and have been automatically generated to be as consistent in population size as possible, and typically contain from four to six Output Areas. The Minimum population is 1000 and the mean is 1500. For more details visit: https://datadictionary.nhs.uk/nhs_business_definitions/lower_layer_super_output_area.html#:~:text=Lower%20Layer%20Super%20Output%20Areas,statistics%20in%20England%20and%20Wales.
[iii] https://www.england.nhs.uk/statistics/statistical-work-areas/cancer-waiting-times/
[iv] http://www.ncin.org.uk/view?rid=4346 (accessed on 24 January 2022)
[v] http://www.ncin.org.uk/view?rid=4347 (accessed on 24 January 2022)

For peer review only

These data packs are produced based on the CWT data, with analysis from Hospital Episode Statistics (HES) and other sources outlined in their technical notes (further details in Annex 1).

Study Outcomes

Primary care consultations

We define consultations in CPRD data by a set of rules developed based on two variables in the consultations file[vi] ("EMIS® consultation source identifier" and "Consultation source code identifier")[vii]. In line with the approach taken by Carey et al 2012 for CPRD Gold data, we use a combination of the consultation code and the category of the record to identify consultations (details in Annex 2).

Using the observation file in CPRD Aurum, we were also able to identify where patients had influenza vaccinations. We look to exclude flu vaccines from our analysis on the basis that the programme was expanded in 2020/21 to achieve maximum uptake[viii]. To help with the comparability of consultations in the two periods, we removed primary care appointments that included a flu vaccine.

Referrals from primary care: routine and urgent cancer

Referrals in CPRD are categorized into routine and "urgent cancer". Referrals from the 'referral file' are linked to patients, no additional data cleaning steps were taken in the analysis of referrals.

First appointment following an urgent referral

The CWT data present monthly counts of patients in England who have been recorded as receiving a first appointment following an urgent referral from primary care. The CWT data record this because the NHS have a 2-week performance target (Annex 3).

The NCRAS cancer equity data contain monthly counts in England of appointments following an urgent cancer referral broken down by tumour type and by deprivation according to patient's place of residence.

First treatment following a cancer diagnosis

The CWT data present monthly counts of patients in England who have been recorded as receiving a first treatment for a new cancer diagnosis. The CWT data record this because the NHS have a 31-day performance target (Annex 3).

The NCRAS cancer equity data contain monthly counts in England of first treatments for new cancer broken down by tumour type and by deprivation according to patient's place of residence.

Patient and public involvement

No patients involved

Data analysis

CPRD & CWT

For both CPRD and CWT we separate the data into two, before and after the introduction of the first NPI.

[vi] https://cprd.com/primary-care

[vii] These variables contain strings that categorise the patient record input and are selected by the staff member completing the record.

[viii] https://www.england.nhs.uk/wp-content/uploads/2020/05/Letter_AnnualFlu_2020-21_20200805.pdf

For peer review only

Our analysis of CPRD primary care is conducted weekly and split into two periods before and after the introduction of NPI on March 23rd 2020 (pre-NPI data is from 03 January 2016 to 21 March 2020, our post-NPI onset data is 22 March 2020 to 30 January 2021).

CWT data is reported monthly, our pre-NPI data is therefore from 01 October 2009 to 31 March 2020, our post-NPI onset period is 01 April 2020 to 31 January 2021.

We perform a linear regression of consultations, urgent and routine referrals from CPRD data and appointments following an urgent cancer referral and first treatments from CWT data over time to estimate expected values for the post-NPI onset period, based on predicted values from the data pre-NPI. To account for seasonality and time trends we include months as a categorical variable and time as a continuous variable, the approach taken by Carr et al.(16). In the case of weekly primary care data, we observe large dips in activity in weeks that include bank holidays and include a categorical variable on the basis of the number of bank holidays in each week (in the winter holidays in England there is always one week with two bank holidays). Our primary care activity rates are presented per 100,000 patient-months[ix]. When analysing primary care consultation rates by socioeconomics we adjust for population age. We do so when calculating the consultation rates by IMD quintile and weighting the sample according to the European Standard Population[x] (ESP).

NCRAS equity data

The analysis presented in the equity data pack compares new instances of first treatments in months during the pandemic (01 April 2020 – 31 Jan 2021) compared with the same months in 2019/20. The analysis includes a 95% confidence interval for the changes, based on rate ratios under an assumption that the population is the same in the pre-COVID-19 baseline and COVID-19 months. This is calculated using the exact method described in Breslow & Day 1987, pp 93-95(20). The NCRAS equity data pack shows the high levels of heterogeneity in the impact of the COVID-19 pandemic on different tumour locations. The NCRAS data equity pack is different in its count and analysis of "all tumours" compared with the Cancer Wait Times Data, this is because the data are slightly different (cleaned and analysed by NCRAS – details in Annex 1). Results of our analysis with each data set are compared in Annex 4. Our presentation of these data follows the same method but presents the cumulative difference for the period from April 2020 to the end of January compared with the previous 12 months.

RESULTS

Overall impact of the pandemic

In the calendar year of 2019, before the COVID-19 pandemic and the associated NPI, there was an average of 39,127 primary care consultations per 100,000 patient-months. This equates to 4.70 attended appointments per registered patient, or an estimated 266 million appointments in primary care nationally in 2019[xi].

Primary care consultations (Figure 1 a) dropped rapidly to a low of 26,919consultations per 100,000 patient-months in the week following 29 March 2020, this was 66.0% lower than the predicted rate. Rates slowly recovered over the next 24 weeks and by 05 September 2020 were up to 99% of the baseline. In total there were an estimated 19.7 million (19.5 to 20.0; 95% CI) fewer primary care consultations in the English NHS during this period. Primary care consultations again fell to below 90% of predicted levels in the third wave NPI starting on 06 January 2021, by the end of January 2021 there were a further 6.4 million fewer consultations than expected. Between the start of the first NPI in

---

[ix] We adjust the weekly rates per active patient in our sample to 100,000 patient-months: Weekly rate per registered patient in sample x 100,000 x (52/12)

[x] https://www.causesofdeath.org/docs/standard.pdf

[xi] For comparison the NHS national appointments data recorded 272 million attended appointments in primary care in 2019. Found here: https://digital.nhs.uk/data-and-information/publications/statistical/appointments-in-general-practice/march-2021

For peer review only - http://bmjopen.bmj.com/site/about/guidelines.xhtml

March 2020 and the end of January 2021 there were an estimated 26.1 million (25.7 to 26.5; 95% CI) fewer appointments than expected (Table 1a).

In 2019, the average rate of urgent cancer (two week wait) referral was 314 per 100,000 patient-months, equating to an estimated 2.12 million for the NHS in England. Following the first NPI, urgent cancer referrals from primary care (Figure 1 b) fell to a nadir of 86 per 100,000 patient-months by 29 March 2020 (29.7% of the predicted level). Urgent cancer referrals did not return to pre-pandemic baseline until the week following 23 August 2020 equating to 317,000 (280,000 to 356,000; 95% CI) estimated lost urgent cancer referrals over this period. There was a second fall in urgent cancer referrals from primary care in the winter to below 90% of the baseline following the third lockdown (164referrals per 100,000 patient-months in the week beginning 27 December 2021). This resulted in a further estimated 91,705 fewer urgent cancer referrals than expected. Between the start of the first NPI in March 2020 and the end of January 2021 there were 395,000 (344,000 to 446,000; 95% CI) fewer urgent cancer referrals than expected (Table 1a).

Routine referrals however have shown a different trajectory in that their rates did not recover to pre-pandemic levels (Figure 1 c). As a share of predicted levels routine referrals had the greatest fall, dropping to 16.1% of predicted rates in the week from 19 April 2020. From then to the end of January the closest it came to predicted levels was 80.3% in the week flowing 13 September 2020. For the four weeks in January 2021, it had fallen back down to 60-70% of predicted rates. In 2019 there were an average of 1,801 routine referrals per 100,000 patient-months from primary care, equivalent to an estimated 12.2 million for the NHS in England. Between the start of the first NPI in March 2020 and the end of January 2021 there were 4.33 million (4.21 to 4.46; 95% CI) fewer routine referrals than expected (Table 1a).

Patient demographics and patient-time and total numbers of observed consultations and routine and urgent referrals in our CPRD sample are presented in Annex 5.

Table 1a: Observed post COVID-19 primary care activity (CPRD Aurum) 22 March 2020 – 30 January 2021

	Observed rate	Expected rate	Percentage reduction (95% CI)	Estimated # missing from England population, to 3 significant digits (95% CI)
Event rate per 100,000 patient-months				
Consultations	34,201	38,684	11.6% (11.4, 11.7)	26,100,000 (25,700,000, 26,500,000)
Routine Referrals	1,067	1,812	41.1% (40.4, 41.8)	4,330,000 (4,210,000, 4,460,000)
2 Week Wait Referrals	268	336	20.2% (18.1, 22.3)	395,000 (344,000, 446,000)

Table 1b: Observed post COVID-19 cancer diagnostic activity (Cancer Wait Times), 01 April 2020 - 31 January 2021

	Observed rate	Expected rate	Percentage reduction (95% CI)	Estimated # missing from England population, to 3 significant digits (95% CI)
Event rate per 100,000 patient-months				
First consultant appointments following urgent referral from primary care	296	366	19.2% (19.1, 19.3)	398,000 (395,000, 401,000)
Incidence rate per 100,000 patient-months				
First treatments for new cancer from the urgent primary care referral pathway	21.4	25.5	16.1% (15.5, 16.8)	23,300 (22,200, 24,400)
First treatments for new cancer from the national screening pathway	1.63	3.47	53.2% (52, 54.3)	10,400 (10,000, 10,900)
First treatments for new cancer	39.7	47.4	16.3% (15.9, 16.6)	43,600 (42,500, 44,700)

For peer review only

For peer review only - http://bmjopen.bmj.com/site/about/guidelines.xhtml

Table 1a summarises the missing appointments and referrals for the post-pandemic period. Since the start of the pandemic in March we have observed consultations rates that are 11.6% (11.4 to 11.7; 95% CI) lower than predicted by previous data. The number of referrals to secondary care per consultation has also fallen, with urgent cancer referrals 20.2% (18.1 to 22.3; 95% CI) and routine referrals 41.1% (40.4 to 41.8; 95% CI) lower than expected.

The knock-on effect of the reductions in patients' primary care appointments and referrals can be observed in the national CWT data. The number of first appointments with a cancer specialist following an urgent cancer referral has fallen by approximately the same amount as estimated for the referrals themselves: 19.2% (19.1 to 19.3; 95% CI). The number of cancer first treatments (following a diagnosis and decision to treat) was 16.3% (15.9 to 16.6; 95% CI) lower than expected, or 43,600 (42,500 to 44,700; 95% CI) missing first treatments from 01 April 2020 - 31 January 2021[xii] (graphs of observed compared with expected are presented in Annex 6).

Urgent cancer referrals by site specific cancer from 01 April 2020 until 31 January 2021 showed significant heterogeneity from moderate reductions in urgent referrals for suspected breast (7.0%; 95% CI 6.6 to 7.5) and gynaecological cancers (10.3%; 95% CI 9.7 to 10.9) and greater reductions for lung (36.9%; 95% CI36.1 to 37.8) and urological (27.2%; 95% CI 26.7 to 27.7) cancers (Figure 2, further details in Annex 4, Table A4.1). To show how pathway delays interface with reductions in cancer referrals we also examined reductions in first treatments for the same site-specific cancers over this period (Figure 2). Breast and urological cancers observed the greatest reduction in new first treatments: Breast fell by 24.8% (23.6 to 25.9; 95% CI) which equates to 10,000 missing treatments and urological by 24.1% (23.2 to 25.2; 95% CI) which equates to 12,100 missing treatments. Taken together these data reflect substantial delays in both diagnostic and treatment phases of the patient pathway.

Inequalities in cancer diagnosis outcomes in the pandemic

There are inequalities in primary care use in England, with the people who live in the poorest areas have higher rates of consultation than those in richer areas once we adjust for age. The most deprived quintile was expected to have 43,184 consultations per 100,000 patient-months (Table 2), 15% more than the least deprived.

Table 2: Observed post COVID-19 primary care activity (CPRD Aurum) by IMD quintile, actual and age-standardised

	22 Mar 2020 - 30 Jan 2021 (Weekly)		
	Observed rate	Expected rate	Percentage reduction (95% CI)
Consultations per 100,000 patient-months			
IMD Quintile - 1 (least deprived)	33,813	38,601	12.4% (12.1, 12.7)
IMD Quintile - 2	34,169	38,793	11.9% (11.6, 12.3)
IMD Quintile - 3	35,069	40,127	12.6% (12.3, 12.9)
IMD Quintile - 4	33,494	37,793	11.4% (11, 11.7)
IMD Quintile - 5 (most deprived)	34,561	38,212	9.6% (9.2, 9.9)
Consultations per 100,000 patient-months (Age-standardised*)			
IMD Quintile - 1 (least deprived)	32,927	37,636	12.5% (12.2, 12.8)
IMD Quintile - 2	33,916	38,647	12.2% (11.9, 12.6)
IMD Quintile - 3	35,535	40,870	13.1% (12.7, 13.4)
IMD Quintile - 4	36,271	41,148	11.9% (11.5, 12.2)
IMD Quintile - 5 (most deprived)	38,997	43,184	9.7% (9.4, 10)

**Age-standardisation is performed according to the European Standard Population (ESP)*

The reduction of consultations over the period 22 March 2020-30 January 2021 was smallest for those in most deprived areas. Their reduction in consultations for the non-age-standardised figures was 9.6% (9.2 to 9.9), while for the least

[xii] Dates for the CWT and NCRAS analysis do not line up with the CPRD analysis because the latter is conducted weekly, not monthly.

For peer review only

deprived the reduction was 12.4% (13.2 to 13.9; 95% CI) (Table 2). Weekly levels of age-standardised consultations per 100,000 patient-months by IMD quintile are presented in Annex 7.

Despite a smaller reduction in primary care contacts, we observe the largest reduction in both urgent cancer referrals and first treatments for cancer for patients living in the most deprived areas. The NCRAS data equity pack presents the number of urgent cancer referrals and first cancer treatments by IMD quintile[xiii]. Figure 3 shows the reduction in urgent cancer referrals and first treatments for newly diagnosed cancer by IMD quintile.

There was a greater percentage reduction in urgent cancer referrals for those living in the most deprived areas in England, who experienced a 17.6% (17.2 to 18.0; 95% CI) reduction between 01 April 2020 and 31 January 2021 compared with the same period 12 months before, while referrals for the least deprived quintile fell by proportionately less: 15.3% (14.9 to 15.6; 95% CI). This equates to a reduction of 61,500 referrals for the most deprived and 62,600 or the least: without adjusting for age, the most deprive quintile had a smaller proportion of the pre-pandemic urgent cancer referrals, with 350,000 referrals compared to 410,000 for the least deprived quintile from April 2019 to January 2020.

At the same time, rates of new treatment for cancer for the people living in the most deprived 20% of England experienced a 15.8% (14.6 to 17.0; 95% CI) reduction between 01 April 2020 and 31 January 2021 compared with the same period 12 months before (6,610 missing first treatments). The reduction for the least deprived was 12.6% (11.5 to 13.7; 95% CI) which equates to 6,880 missing first treatments.

Despite having more access to primary care for patients in more deprived areas (9.7% reduction for most deprived compared to 12.5% for the least deprived), urgent cancer referrals and newly diagnosed cancers have been disrupted by the pandemic more for people living in poorer areas.

DISCUSSION

The coronavirus SARS-CoV-2 (COVID-19) pandemic has had a profound impact on the management of patients with cancer(21). The first national lockdown in March 2020 created a ripple of non-pharmaceutical interventions, including 'stay at home' orders, diminished healthcare service provision and redistribution of healthcare to COVID-19 related care that has had a profound impact on cancer services(1,22).

There are also new potential barriers to the pathway that have resulted and may exacerbate these findings. For example, decreases in health seeking behaviour due to fear of acquiring COVID-19 infection through interactions with health care settings, increasing use of remote consultations(23), changes in routine referral guidelines(24), as well as changes in the capacity of acute care. The backlog for routine diagnostic services is a particular concern given that approximately 40% of cancer are diagnosed through this route(25).

Similar issues have also been identified within the health systems of other high-income countries. Primary care providers in eight European countries experienced similar issues in how to rapidly transform services in the wake to COVID-19(26). A study in Sweden found an almost identical percentage reduction in primary care consultations (12%) as a results of the pandemic(27), in Norway there was a 24% reduction in cancer referrals(28), the Netherlands had a 26% reduction in non-skin cancer diagnoses(29) and in Belgium there was a 44% reduction in diagnosis of invasive tumours in the first wave of the pandemic(30). Our results don't appear to be unique to England: while different countries can have different routes to diagnosis(31), many countries also observed disruptions to cancer pathways(32–35).

Whilst it was already known that there had been a substantial reduction in the number of overall cancer-related referrals(36,37) the quantification of this had been missing. Our findings, that primary care consultations in English NHS fell by 12.4% between January 2020 and January 2021 with urgent cancer referrals even more suppressed (20.2%), reflect how profound the pathway disruptions were for cancer patients. Furthermore, many cancers are picked up

---

[xiii] They do not age-standardise their results

For peer review only - http://bmjopen.bmj.com/site/about/guidelines.xhtml

through the course of routine referrals from general practice for non-specific symptoms. The drop in routine referrals that we found (4.3 million, over this period) will inevitably translate into late-stage presentation and a substantial reduction in outcomes. This will include wider economic costs due to more expensive, late-stage treatment and productivity losses due to morbidity and premature mortality. However, the trajectory of the declines reflect not just changes to national policy in terms of NPI, but also knock-on effects around public behaviour, primary care staffing, downstream reductions in diagnostics and an overall increase in friction across all cancer pathways and systems.

This reduction in cancer pathways through primary care needs to be put in the context of wider disruptions. The suspension of national cancer screening programmes meant that around 2 million people were not screened for cancer through national programs(38,39). Moreover, delays in cancer diagnoses and treatments have consistently been associated with poorer outcomes(1,2). The COVID-19 pandemic has also exacerbated the worst 62-day cancer waiting time targets in the last decade where 1 of 4 patients urgently referred from primary care between April 2020 and January 2021 did not receive treatment within 62 days(37).

In our analysis of urgent cancer referrals by site in relation to reductions seen in first treatments significant differences were seen, which is also reflected in the international evidence. Urological cancers (testis, renal, prostate and urothelial) have been particularly impacted with greater than 25% decrease both in urgent referrals and first treatments. This suggests that outcomes will be particularly impacted in this group. Lung, skin and lower GI (colon and rectal) cancer also experienced significant declines in urgent referrals, in the Netherlands there was a 60% reduction in skin cancer diagnosis during the first wave(29).

Breast cancer was the least impacted of all in terms of urgent referrals but experienced a 25% reduction in first treatments. This highlights how much breast cancer diagnosisrelies on screening programmes which have suffered badly as a result of the pandemic, in England(37) and internationally(40). In England, head and neck cancers (HNC) in saw a 10.2% (7.6 to 12.7; 95% CI) reduction in diagnosis, while studies in other geographies showed wide differences in the measures impacts of the pandemic on HNC: a study in Ontario, Canada found no evidence of a reduction in head and neck cancer diagnosis following an initial drop in the 6 weeks following lockdown(41), a clinic in Italy had just a 3.7% reduction in HNC(42), a 14% reduction in Belgium(30), a clinic in California showed a 22% reduction(43) and a Cancer Centre in the North of England reported a 33% reduction in NHC cases(44). There is further international evidence of the impact of COVID-19 on interventions down the pathway, with reductions in radical cancer surgeries in two major cancer hubs in England and Italy(45).

Differences in systems, populations and NPI from the pandemic present high levels of complexity in tackling the recovery at both a national and local level. Although it is possible that, in many countries, some cancer patients have already been ‘lost’ to the system i.e. died of COVID-19 or other non-COVID-19 comorbidities, a significant number will now present with later stage disease, creating further pressure on acute cancer care.

Our findings also reflect socioeconomic inequalities, with more profound decrease in urgent cancer referrals and first treatments for the most deprived populations despite relatively better preservation of consultation rates. This is unexpected and extremely worrying, indicating greater disruption to the diagnostic pathway for patients living in more deprived areas, whose cancer outcomes were typically worse than their less deprived counterparts pre-pandemic(46,47). Resilience in primary care is key for the cancer diagnosis pathway and must be developed. We know that there are challenges associated with resourcing health services in poorer areas (the inverse care law(48)), resulting in fewer resources per head of sick patient(10) and shorter consultation times(49). Further research should focus on understanding to what extent complex morbidity, which is greater in poorer areas(8,50), contributes to the disruption of the cancer diagnostic pathway. Greater understanding would help health systems better prepare for the kind of disruption we have seen as a result of COVID-19.

Limitations

This study uses multiple data sets to analyse a complex and disjointed pathway. We include a primary care data set that uses a relatively small (500,000) patient sample. However, the CPRD data produces results that closely mirror the rates

For peer review only

of consultation per patient (and their reduction) produced in NHS Digital's appointments data(51). In addition, the estimated reduction in urgent cancer referrals is close to those presented in the NCRAS's analysis of their cancer registry data (Table 1a & Table 1b). It is not yet possible to link these data on a patient basis due to delays in data access and once possible further research would be illuminating.

Conclusions

Our data reflects a disruption to a complex interaction of several systemic issues that place a great deal of impetus on the role of primary care in ensuring early diagnosis of cancer. Primary care was already under strain pre-pandemic, with low levels of investment and workforce deficits(52). Particularly in areas of high deprivation, general practice is under-funded and under staffed relative to need(7,8,10).

Early cancer diagnosis requires concordance of each participant and mechanism - including patients' awareness and ability to present with cancer symptoms, the ability of GPs to detect and urgently refer possible cancer cases and sufficient diagnostic capacity (in terms of both workforce and equipment) to enable swift referrals and minimise delays to diagnosis and treatment. Every one of these nodes on the pathway to early diagnosis has been affected by the pandemic and the national policy response. However, further work is required as there is currently little understanding and even less evidence about how much each disruption is ultimately impacting cancer pathways.

The impact of the pandemic on cancer diagnosis and time to treatment shown here is very serious. However, what is more concerning is the unequal and inequitable impact on those worst off. Cancer as a disease area "*magnifies what we know to be true about the totality of the health care system. It exposes all its strengths and weaknesses*"(53). Our results further evidence the strain on primary care, the presence of the inverse care law(48), and the dire need to address the inequalities so sharply brought into focus by the pandemic. We need to address the disconnect between the importance we place on the role of primary care in cancer care and the resources we devote to it.

For peer review only

Figure legends:

Figure 1: Observed vs expected primary care activity, 01 Jan 2019 – 30 January 2021 (per 100,000 patient-months) (CPRD Aurum data) Panels: a. Consultations, b. Urgent cancer (2 week wait) referrals from primary care, c. Routine referrals from primary care

Figure 2: Percentage difference between observed and expected first treatments for new cancer and urgent cancer referrals by tumour location from NCRAS Cancer equity data pack (%, 01 Apr 2020 to 31 Jan 2021)

Figure 3: Percentage difference between observed and expected urgent cancer referrals and first treatments for cancer by IMD quintile (01 Apr 2020 – 31 Jan 2021)

Contributors: TW, RS and AA designed the study. Data acquisition, cleaning and analysis was conducted by TW on the Health Foundation's secure date environment. TW wrote the manuscript in the first instance. TW, RS and AA interpreted the data and substantially reviewed the draft manuscript. All authors approved the final version of the manuscript. TW and RS are the guarantors. The corresponding author attests that all listed authors meet authorship criteria and that no others meeting the criteria have been omitted.

Competing interests: None

Funding: This publication is funded through the UK Research and Innovation GCRF grant ES/P010962/1.

Ethics approval: CPRD collect data for research use. We did not require ethical approval however, scientific approval for this study was given by the CPRD Independent Scientific Advisory Committee (ISAC). The study was approved by the Independent Scientific Advisory Committee for CPRD research (20_143). No additional ethics approval was required for this study.

Data Availability statement:

The primary care activity data may be obtained from a third party and are not publicly available. We used de-identified primary care data from the Clinical Practice Research Datalink (CPRD). For more information please visit: https://www.cprd.com/Data-access, enquiries can be emailed to enquiries@cprd.gov.uk. Scientific approval for this study was given by the CPRD Independent Scientific Advisory Committee (ISAC). The study was approved by the Independent Scientific Advisory Committee for CPRD research (20_143). The data is provided by patients and collected by the NHS as part of their care and support.

Other data sources are available in a public, open access repository: Cancer Wait Times at https://www.england.nhs.uk/statistics/statistical-work-areas/cancer-waiting-times/ and the NCRAS Cancer data equity pack is available at http://www.ncin.org.uk/local_cancer_intelligence/cadeas.

For peer review only

References

1. Maringe C, Spicer J, Morris M, Purushotham A, Nolte E, Sullivan R, et al. The impact of the COVID-19 pandemic on cancer deaths due to delays in diagnosis in England, UK: a national, population-based, modelling study. Lancet Oncol. 2020;21(8).

2. Hanna TP, King WD, Thibodeau S, Jalink M, Paulin GA, Harvey-Jones E, et al. Mortality due to cancer treatment delay: systematic review and meta-analysis. BMJ. 2020;371.

3. Purushotham A, Roberts G, Haire K, Dodkins J, Harvey-Jones E, Han L, et al. The impact of national non-pharmaceutical interventions ('lockdowns') on the presentation of cancer patients. Ecancermedicalscience. 2021;15.

4. Health at a Glance: Europe 2020 [Internet]. OECD; 2020 [cited 2021 Oct 14]. (Health at a Glance: Europe). Available from: https://www.oecd-ilibrary.org/social-issues-migration-health/health-at-a-glance-europe-2020_82129230-en

5. Meiklejohn JA, Mimery A, Martin JH, Bailie R, Garvey G, Walpole ET, et al. The role of the GP in follow-up cancer care: a systematic literature review. Vol. 10, Journal of Cancer Survivorship. 2016.

6. PHE. Routes to Diagnosis [Internet]. National Cancer Registration and Analysis Service. [cited 2021 Oct 14]. Available from: http://www.ncin.org.uk/publications/routes_to_diagnosis

7. Parisi R, Lau YS, Bower P, Checkland K, Rubery J, Sutton M, et al. Rates of turnover among general practitioners: A retrospective study of all English general practices between 2007 and 2019. Vol. 11, BMJ Open. 2021.

8. Head A, Fleming K, Kypridemos C, Schofield P, Pearson-Stuttard J, O'Flaherty M. Inequalities in incident and prevalent multimorbidity in England, 2004–19: a population-based, descriptive study. Lancet Heal Longev. 2021;2(8).

9. DHSC, ONS. Direct and Indirect health impacts of COVID-19 in England [Internet]. London; 2021 Sep [cited 2021 Oct 14]. Available from: https://assets.publishing.service.gov.uk/government/uploads/system/uploads/attachment_data/file/1018698/S1373_Direct_and_Indirect_Health_Impacts_of_C19_Detailed_Paper_.pdf

10. Fisher R, Dunn P, Asaria M, Thorlby R. Level or not? Comparing general practice in areas of high and low socioeconomic deprivation in England. London; 2020 Sep.

11. NHS. NHS Long Term Plan Summary. NHS Improv. 2019;

12. Moscrop A. Health inequalities in primary care: Time to face justice. Vol. 62, British Journal of General Practice. 2012.

13. Cookson R, Mondor L, Asaria M, Kringos DS, Klazinga NS, Wodchis WP. Primary care and health inequality: Difference-in-difference study comparing England and Ontario. PLoS One. 2017;12(11).

14. IFG. Timeline of UK coronavirus lockdowns, March 2020 to March 2021 [Internet]. Institute for Government . 2021 [cited 2021 Oct 14]. Available from: https://www.instituteforgovernment.org.uk/sites/default/files/timeline-lockdown-web.pdf

15. Health and Social Care Committee & Science and Technology Committee. Coronavirus: lessons learned to date [Internet]. London; 2021 Oct [cited 2021 Oct 14]. Available from: https://committees.parliament.uk/work/657/coronavirus-lessons-learnt/news/157991/coronavirus-lessons-learned-to-date-report-published/

16. Carr MJ, Steeg S, Webb RT, Kapur N, Chew-Graham CA, Abel KM, et al. Effects of the COVID-19 pandemic on primary care-recorded mental illness and self-harm episodes in the UK: a population-based cohort study. Lancet Public Heal. 2021;6(2).

For peer review only

17. Herrett E, Gallagher AM, Bhaskaran K, Forbes H, Mathur R, Staa T van, et al. Data Resource Profile: Clinical Practice Research Datalink (CPRD). Int J Epidemiol. 2015;44(3).

18. Padmanabhan S, Carty L, Cameron E, Ghosh RE, Williams R, Strongman H. Approach to record linkage of primary care data from Clinical Practice Research Datalink to other health-related patient data: overview and implications. Eur J Epidemiol. 2019;34(1).

19. NHS, PHE. The Cancer Alliance Data, Evaluation and Analysis Service [Internet]. National Cancer Registration and Analysis Service: Local Cancer Intelligence. 2021 [cited 2021 Oct 14]. Available from: http://www.ncin.org.uk/local_cancer_intelligence/cadeas#covid-19

20. Breslow NE, Day NE. Statistical methods in cancer research. Volume II--The design and analysis of cohort studies. IARC scientific publications. 1987.

21. Raymond E, Thieblemont C, Alran S, Faivre S. Impact of the COVID-19 Outbreak on the Management of Patients with Cancer. Vol. 15, Targeted Oncology. 2020.

22. UK Government. Staying at home and away from others (social distancing) [Internet]. Cabinet Office. 2020 [cited 2021 Oct 29]. Available from: https://www.gov.uk/government/publications/full-guidance-on-staying-at-home-and-away-from-others

23. Green MA, McKee M, Katikireddi SV. Remote general practitioner consultations during COVID-19. Lancet Digit Heal. 2022 Jan;4(1):e7.

24. Dunn P, Allen L, Cameron G, Malhotra AM, Alderwick H. COVID-19 policy tracker 2020: A timeline of national policy and health system responses to COVID-19 in England [Internet]. The Health Foundation. 2021 [cited 2022 Jan 25]. Available from: https://www.health.org.uk/news-and-comment/charts-and-infographics/covid-19-policy-tracker

25. Elliss-Brookes L, McPhail S, Ives A, Greenslade M, Shelton J, Hiom S, et al. Routes to diagnosis for cancer - Determining the patient journey using multiple routine data sets. Br J Cancer. 2012;107(8).

26. Wanat M, Hoste M, Gobat N, Anastasaki M, Böhmer F, Chlabicz S, et al. Transformation of primary care during the COVID-19 pandemic: Experiences of healthcare professionals in eight European countries. Br J Gen Pract. 2021;71(709).

27. Ekman B, Arvidsson E, Thulesius H, Wilkens J, Cronberg O. Impact of the Covid-19 pandemic on primary care utilization: evidence from Sweden using national register data. BMC Res Notes. 2021;14(1).

28. Sagan A, Webb E, Azzopardi-Muscat N, de la Mata I, McKee M, Figueras J. Health systems resilience during COVID-19: Lessons for building back better [Internet]. 2021 Nov [cited 2022 Feb 17]. Available from: https://eurohealthobservatory.who.int/publications/i/health-systems-resilience-during-covid-19-lessons-for-building-back-better

29. Dinmohamed AG, Visser O, Verhoeven RHA, Louwman MWJ, van Nederveen FH, Willems SM, et al. Fewer cancer diagnoses during the COVID-19 epidemic in the Netherlands. Lancet Oncol. 2020 Jun;21(6):750–1.

30. Peacock HM, Tambuyzer T, Verdoodt F, Calay F, Poirel HA, De Schutter H, et al. Decline and incomplete recovery in cancer diagnoses during the COVID-19 pandemic in Belgium: a year-long, population-level analysis. ESMO Open. 2021 Aug;6(4):100197.

31. Arnold M, Rutherford MJ, Bardot A, Ferlay J, Andersson TML, Myklebust TÅ, et al. Progress in cancer survival, mortality, and incidence in seven high-income countries 1995–2014 (ICBP SURVMARK-2): a population-based study. Lancet Oncol. 2019;20(11).

32. Riera R, Bagattini ÂM, Pacheco RL, Pachito DV, Roitberg F, Ilbawi A. Delays and Disruptions in Cancer Health Care Due to COVID-19 Pandemic: Systematic Review. JCO Glob Oncol. 2021;(7).

33. Gurney JK, Millar E, Dunn A, Pirie R, Mako M, Manderson J, et al. The impact of the COVID-19 pandemic on cancer diagnosis and service access in New Zealand–a country pursuing COVID-19 elimination. Lancet Reg Heal -

For peer review only

West Pacific. 2021;10.

34. Priou S, Lamé G, Chatellier G, Tournigand C, Kempf E. Effect of the COVID-19 pandemic on colorectal cancer care in France. Vol. 6, The Lancet Gastroenterology and Hepatology. 2021.

35. Jacob L, Loosen SH, Kalder M, Luedde T, Roderburg C, Kostev K. Impact of the COVID-19 pandemic on cancer diagnoses in general and specialized practices in Germany. Cancers (Basel). 2021;13(3).

36. Morris EJA, Goldacre R, Spata E, Mafham M, Finan PJ, Shelton J, et al. Impact of the COVID-19 pandemic on the detection and management of colorectal cancer in England: a population-based study. Lancet Gastroenterol Hepatol. 2021;6(3).

37. Cancer Waiting Times Team: NHS England and NHS Improvement. Cancer Wait Times Statistics [Internet]. NHS England. 2021 [cited 2021 Oct 25]. Available from: https://www.england.nhs.uk/statistics/statistical-work-areas/cancer-waiting-times/

38. Sud A, Torr B, Jones ME, Broggio J, Scott S, Loveday C, et al. Effect of delays in the 2-week-wait cancer referral pathway during the COVID-19 pandemic on cancer survival in the UK: a modelling study. Lancet Oncol. 2020;21(8).

39. Greenwood E, Swanton C. Consequences of COVID-19 for cancer care — a CRUK perspective. Vol. 18, Nature Reviews Clinical Oncology. 2021.

40. Figueroa JD, Gray E, Pashayan N, Deandrea S, Karch A, Vale DB, et al. The impact of the Covid-19 pandemic on breast cancer early detection and screening. Prev Med (Baltim). 2021 Oct;151:106585.

41. Noel CW, Li Q, Sutradhar R, Eskander A. Total Laryngectomy Volume During the COVID-19 Pandemic. JAMA Otolaryngol Neck Surg. 2021 Oct 1;147(10):909.

42. Longo F, Trecca EMC, D'Ecclesia A, Copelli C, Tewfik K, Manfuso A, et al. Managing head and neck cancer patients during the COVID-19 pandemic: the experience of a tertiary referral center in southern Italy. Vol. 16, Infectious Agents and Cancer. 2021.

43. Solis RN, Mehrzad M, Faiq S, Frusciante RP, Sekhon HK, Abouyared M, et al. The Impact of COVID-19 on Head and Neck Cancer Treatment: Before and During the Pandemic. OTO Open. 2021 Oct 23;5(4):2473974X2110680.

44. Iqbal MS, Uzzaman L, Fox H, Munro S, Kelly C. The impact of COVID-19 on the presentation, stage and management of head and neck cancer patients: A real-time assessment. Clin Otolaryngol. 2022 Jan 26;47(1):251–3.

45. Monroy-Iglesias MJ, Tagliabue M, Dickinson H, Roberts G, De Berardinis R, Russell B, et al. Continuity of cancer care: The surgical experience of two large cancer hubs in london and milan. Cancers (Basel). 2021;13(7).

46. Coleman MP, Rachet B, Woods LM, Mitry E, Riga M, Cooper N, et al. Trends and socioeconomic inequalities in cancer survival in England and Wales up to 2001. Br J Cancer. 2004;90(7).

47. Cancer Research UK. Cancer incidence for all cancers combined: cancer incidence by deprivation [Internet]. 2020 [cited 2021 Oct 14]. Available from: https://www.cancerresearchuk.org/health-professional/cancer-statistics/incidence/all-cancers-combined#heading-Three

48. Tudor Hart J. THE INVERSE CARE LAW. Lancet. 1971;297(7696).

49. Gopfert A, Deeny SR, Fisher R, Stafford M. Primary care consultation length by deprivation and multimorbidity in England: An observational study using electronic patient records. Br J Gen Pract. 2021;71(704).

50. Ingram E, Ledden S, Beardon S, Gomes M, Hogarth S, Mcdonald H, et al. Household and area-level social determinants of multimorbidity: a systematic review. Vol. 75, Journal of Epidemiology and Community Health. 2021.

51. NHS. Appointments in General Practice - September 2021 [Internet]. NHS Digital. 2021 [cited 2021 Nov 16].

For peer review only

Available from: https://digital.nhs.uk/data-and-information/publications/statistical/appointments-in-general-practice

52. Roland M, Everington S. Tackling the crisis in general practice. BMJ. 2016;

53. Levit LA, Balogh EP, Nass SJ, Ganz PA. Delivering high-quality cancer care: Charting a new course for a system in crisis. Delivering High-Quality Cancer Care: Charting a New Course for a System in Crisis. 2014.

Key messages

- Primary care is key part of the pathway for early cancer diagnosis through both routine and 2ww referrals
- Cancer diagnosis rates have experienced a sustained fall since the start of the COVID-19 pandemic and introduction of non-pharmaceutical interventions (NPIs) ‘lockdowns’.
- The fall in urgent cancer referral is larger than the fall in primary care contacts, implying that the content of consultations has shifted away from potential cancer diagnosis.
- Despite having a smaller reduction in primary care contact through the pandemic, patients living in poorer areas have had larger reductions in urgent cancer referrals and first treatments for new cancer.
- Government, patients and primary care staff must work together to catch up on missing diagnosis.
- Resilience in primary care is key for the cancer diagnosis pathway and must be developed for future disruptions, particularly in poorer areas where care is more complex.

For peer review only

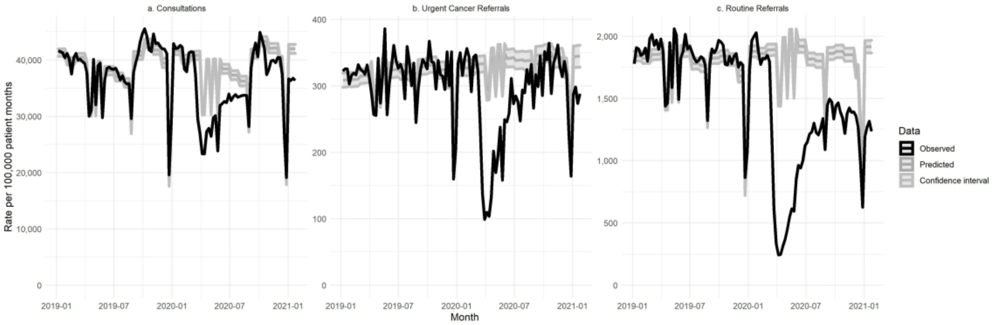

Figure 1: Observed vs expected primary care activity, 01 Jan 2019 – 30 January 2021 (per 100,000 patient-months) (CPRD Aurum data) Panels: a. Consultations, b. Urgent cancer (2 week wait) referrals from primary care, c. Routine referrals from primary care

381x127mm (300 x 300 DPI)

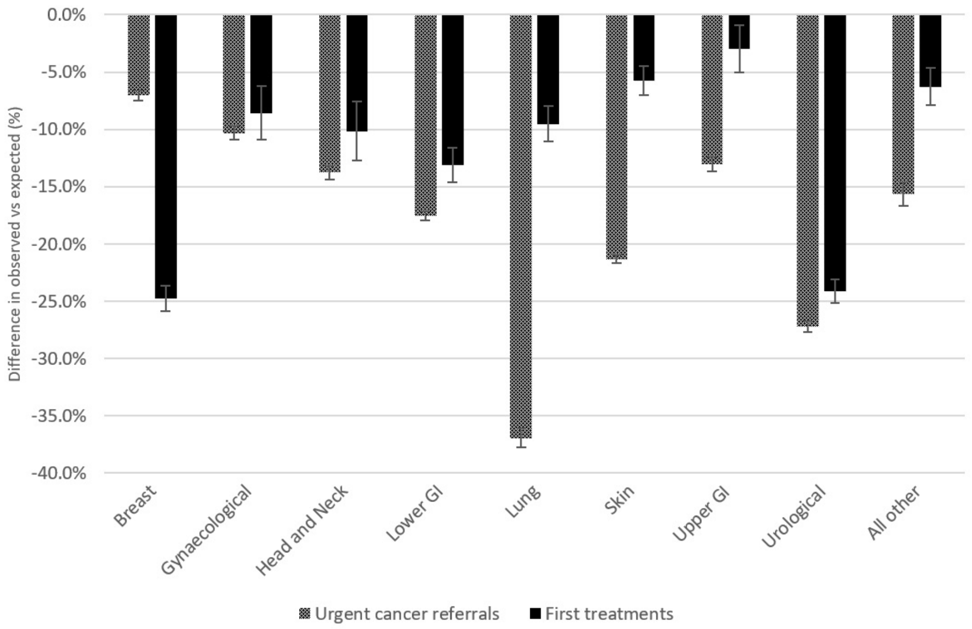

Figure 2: Percentage difference between observed and expected first treatments for new cancer and urgent cancer referrals by tumour location from NCRAS Cancer equity data pack (%, 01 Apr 2020 to 31 Jan 2021)

233x150mm (120 x 120 DPI)

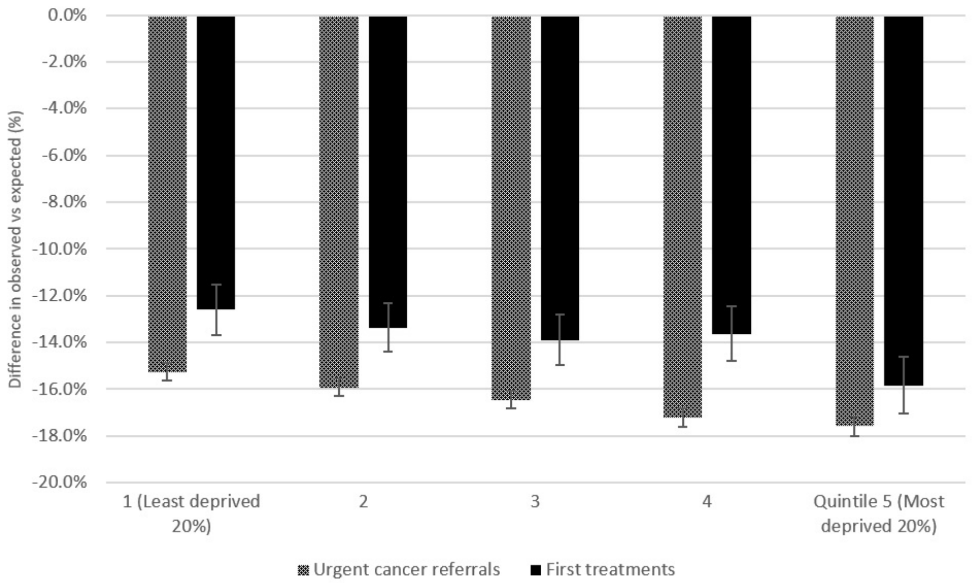

Figure 3: Percentage difference between observed and expected urgent cancer referrals and first treatments for cancer by IMD quintile (01 Apr 2020 – 31 Jan 2021)

205x122mm (120 x 120 DPI)

Annex 1: NCRAS data equity pack, technical notes

CADEAS and NCRAS have produced two equity data packs presenting the latest national data on the number of urgent suspected two-week wait referrals and first definitive treatments for cancer. These data packs are produced on the basis of the Cancer Wait Times data, with analysis from Hospital Episode Statistics (HES) and other sources outlined in their technical notes.

"Any differences between treatment volumes in the published official statistics and the volumes presented in this pack are because:

- Data was extracted from the CWT system at a slightly different time.
- Data included here is based on England residents only.

Additional logic has been applied to remove treatments where some of the information required for this equity analysis is missing or there are potential data quality issues, for example cases with a mismatch between the suspected cancer referral type and sex (eg. gynaecological cancer treatments for men, testicular cancer treatments for women), and suspected cancer referral type and age (eg. suspected children's cancer for patients aged 20 and over)." – NCRAS Cancer data equity pack technical notes, final tab within the downloaded spreadsheet. Available under "Links to data": http://www.ncin.org.uk/local_cancer_intelligence/cadeas as at 26/01/2022

Annex 2: Consultation definition

CPRD Aurum data dictionary sets out the structure of the data. Within the consultation file there are two variables one can use to identify whether a primary care contact, rather than an administrative note ("EMIS® consultation source identifier" and "Consultation source code identifier").

The EMIS consultation source identifier is the primary variable used. We include the following observations of this variable:

Acute visit, Casualty attendance, Clinic, Emergency appointment, Emergency consultation, Enterprise consultation, Face to face consultation, Follow-up/routine visit, Gp surgery, Home visit, Home visit note, Main surgery, Nursing home, Nursing home visit note, Online services message, Other, Residential home, Residential home visit note, Same day appointment, Surgery consultation, Telephone encounter, Urgent consultation, Walk-in centre, Walk-in clinic

We also include instances where EMIS consultation source identifier is "awaiting review" and the Consultation source code identifier is in the following list:

Consultation, visit, seen in gp unit, seen in private clinic, seen in rapid access clinic at gp surgery, seen in urgent care centre, online communication.

We then further exclude records on the basis of the category of staff responsible for the record. The "Job category" variable from the staff file, linked by the consultation id is used. We only include as a consultation records filled out by GPs, doctors, nurses and other health care professionals as defined in CPRD's numerical codes listed below:

GP – 4, 5, 15, 24, 31, 181, 183

Dr – 1, 41, 91, 116, 119, 121, 126, 173, 177, 197

Nurse – 8, 9, 27, 33, 47, 48, 50, 55, 59, 60, 61, 111

Other healthcare professional - 2, 3, 6, 7, 10:14, 16, 17, 34:37, 42, 43, 52, 54, 58, 62:65, 68, 72, 73, 77, 80, 82, 83, 86:89, 94, 95, 97, 100:102, 105, 106, 112:114, 118, 122, 125, 127, 131, 135, 136, 138, 141, 142, 145, 148, 149, 154, 156, 158, 168, 185, 186, 188, 189, 204, 208

In Table A2.1 we present the CPRD Aurum Staff Category list.

In Table A2.2 we present the total number of consultations identified from 01 January 2016 to 31 January 2021, the count with each combination of staff category, "EMIS® consultation source identifier" and "Consultation source code identifier" in Table A2.2 we show the count of records that were excluded, highlighting those that were excluded on the basis of staff category, not the consultation file description variables.

For peer review only

Table A2.1: CPRD Aurum Staff Job Categories

1 Consultant
2 Hospital Practitioner
3 Clinical Medical Officer
4 General Medical Practitioner
5 Salaried General Practitioner
6 Midwife - Sister/Charge Nurse
7 Midwife
8 Community Practitioner
9 Community Nurse
10 Chiropodist/Podiatrist
11 Dietitian
12 Pharmacist
13 Clinical Psychologist
14 Health Care Support Worker
15 Associate Practitioner - General Practitioner
16 Counsellor
17 Phlebotomist
18 Clerical Worker
19 Manager
20 Analyst
21 System Administrator
22 Desktop Support Administrator
23 System Worker
24 GP Registrar
25 Medical Student
26 Other Community Health Service - Admin Clerk
27 Specialist Nurse Practitioner
28 Receptionist
29 Secretary
30 Medical Secretary
31 Sessional GP
32 Clinical Application Administrator
33 Nurse Consultant
34 Physiotherapist
35 Specialist Practitioner
36 Healthcare Assistant
37 Medical Technical Officer - Pharmacy
38 Health Records Administrator
39 Helpdesk Administrator
40 Appointments Clerk
41 Senior House Officer
42 Social Worker
43 Trainee Practitioner
44 Network Technician
45 Clinical Coder
46 Medical Records Clerk
47 Staff Nurse
48 Enrolled Nurse
49 Multi Therapist
50 Nursery Nurse
51 Helper/Assistant
52 Community Mental Health Nurse
53 Senior Administrator
54 Technician - Healthcare Scientists
55 Associate Practitioner - Nurse
56 Senior Manager
57 Community Administrator
58 Associate Specialist
59 Student Practice Nurse
60 Nurse Manager
61 Sister/Charge Nurse
62 Psychotherapist
63 Osteopath
64 Social Care Support Worker
65 Assistant Psychologist
66 Officer
67 Technician - Admin & Clerical
68 Psychiatrist
69 Health Records Clerk
70 Desktop Support Technician
71 Dispenser
72 Clinical Assistant
73 Practitioner
74 Information Officer
75 Network Administrator
76 Chaplain
77 Student Physiotherapist
78 Paramedic Specialist Practitioner
79 Clinical Team Manager
80 Physiotherapist Specialist Practitioner
81 Helpdesk Technician
82 Radiographer
83 Other Community Health Service
84 Call Operator
85 Community Worker (children)
86 Paramedic Consultant
87 Associate Practitioner
88 Modern Matron
89 Asst. Clinical Medical Officer
90 Community Team Manager
91 Specialist Registrar
92 Chiropodist/Podiatrist Manager
93 Radiographer - Therapeutic, Manager
94 Optometrist
95 Assistant Practitioner
96 Community Learning Disabilities Nurse
97 Technician - Additional Clinical Services
98 Student Health Visitor
99 Interpreter
100 Medical Technical Officer

For peer review only

For peer review only - http://bmjopen.bmj.com/site/about/guidelines.xhtml

101 Midwife - Specialist Practitioner
102 Occupational Therapist
103 Chief Executive
104 Audit Manager
105 Paramedic
106 Physiotherapist Consultant
107 Availability Monitor
108 Medical Laboratory Assistant
109 Gateway Worker
110 Medical Records Manager
111 Student Nurse - Adult Branch
112 Audiologist
113 Radiographer - Diagnostic
114 Therapist
115 Student District Nurse
116 House Officer - Post Registration
117 Speech & Language Therapist
118 Dietitian Specialist Practitioner
119 Trust Grade Doctor - SHO level
120 Director of Public Health
121 Staff Grade
122 Patient Welfare Officer
123 Occupational Therapy Specialist Practitioner
124 Technician - PS&T
125 Chiropodist/Podiatrist Consultant
126 Trust Grade Doctor - Career Grade level
127 Student Community Practitioner
128 Healthcare Scientist
129 Waiting List Clerk
130 Clinical Director
131 Pre-reg Pharmacist
132 Mental Health Act Administrator
133 Ward Clerk
134 Support, Time, Recovery Worker
135 Art Therapist Specialist Practitioner
136 Physiotherapist Manager
137 Healthcare Cadet
138 Dietitian Consultant
139 Orthoptist Manager
140 Social work assistant (mental health)
141 Chiropodist/Podiatrist Specialist Practitioner
142 Student Technician
143 Complaints Investigator
144 Trainee Scientist
145 Radiographer - Diagnostic, Manager
146 Social services care manager (mental health)
147 Dietitian Manager
148 Midwife - Consultant
149 Art Therapist Consultant
150 Paramedic Manager
151 Finance Director
152 Senior social worker (adults)
153 Student Midwife
154 Radiologist
155 Ward Manager
156 Midwife - Manager
157 Waiting List Manager
158 Radiographer - Diagnostic, Specialist Practitioner
159 Biomedical Scientist
160 Board Level Director
161 Non Executive Director
162 Nursing Cadet
163 Porter
164 Social services care manager (adults)
165 Student Psychotherapist
166 Orthoptist
167 Clinical Director - Medical
168 Approved Social Worker
169 Student Community Mental Health Nurse
170 Other Executive Director
171 Student Orthoptist
172 Childcare Co-ordinator
173 House Officer - Pre Registration
174 SODP
175 Outpatient Manager
176 Medical Director
177 Trust Grade Doctor - Specialist Registrar level
178 Senior Clinical Medical Officer
179 Consultant Healthcare Scientist
180 Reporting Radiographer
181 Locum GP
182 Researcher
183 Assistant GP
184 Special salary scale in Public Health Medicine
185 Advanced Practitioner
186 Health Visitor
187 Dental Assistant Clinical Director
188 Other Community Health Service - Social Care Worker
189 Physician Assistant
190 Deputising Doctor
191 Student Occupational Health Nurse
192 Senior social worker (mental health)
193 Regional Dental Officer
194 Trainer
195 Cytoscreener
196 Chair
197 Trust Grade Doctor - House Officer level
198 Art Therapist
199 Multi Therapist Specialist Practitioner
200 Drama Therapist
201 Healthcare Science Assistant
202 Social work assistant (adults)
203 Social work team manager (adults)

For peer review only

204 Intermediate Care worker
205 Student Occupational Therapist
206 Student Dietitian
207 Healthcare Science Associate
208 Child Protection worker
209 Professor
210 General Dental Practitioner
211 Student School Nurse
212 Occupational Therapist Consultant
213 Intermediate Care staff
214 Home help
215 Art, Music & Drama Student
216 Specialist Healthcare Scientist
217 Social Services information manager

For peer review only

Table A2.2: Number of observations by EMIS® consultation source identifier, Consultation source code identifier and Staff Job Category, with an indicator for whether it was included as a consultation: Include: 1 = Include, 0 = Exclude, "Excl. job title" = Excluded on the basis of job title.

Include	Staff Job Category	Consultation source code identifier	EMIS consultation source identifier	Count
1	gp	gp surgery	gp surgery	3,119,080
1	nurse	gp surgery	gp surgery	1,692,606
1	gp	telephone consultation	telephone consultation	1,471,946
1	other care provider	gp surgery	gp surgery	977,067
1	nurse	telephone consultation	telephone consultation	196,665
1	gp	telephone call to a patient	telephone call to a patient	108,025
1	gp	home visit note	home visit note	83,600
1	other care provider	telephone consultation	telephone consultation	81,980
1	nurse	telephone call to a patient	telephone call to a patient	32,423
1	gp	face to face consultation	face to face consultation	25,435
1	nurse	home visit note	home visit note	24,174
1	other care provider	telephone call to a patient	telephone call to a patient	23,194
1	gp	gp surgery	surgery consultation	22,756

1	gp	nursing home visit note	nursing home visit note	21,357
1	nurse	face to face consultation	face to face consultation	17,580
1	gp	enterprise consultation	enterprise consultation	14,904
1	gp	telephone call from a patient	telephone call from a patient	13,062
1	gp	routine consultation	surgery consultation	11,668
1	other care provider	home visit note	home visit note	10,853
1	gp	provision of general practitioner intermediate care	gp surgery	10,441
1	gp	emergency consultation	emergency consultation	10,351
1	gp	residential home visit note	residential home visit note	9,579
1	other care provider	face to face consultation	face to face consultation	9,350
1	gp	emergency appointment	emergency appointment	8,687
1	gp	urgent consultation	urgent consultation	8,155
1	gp	walk-in clinic	walk-in clinic	7,908
1	dr	gp surgery	gp surgery	7,654
1	gp	other note	other	7,520
1	gp	face to face consultation	surgery consultation	6,932
1	nurse	gp surgery	surgery consultation	5,318
1	gp	seen in gp unit	surgery consultation	4,687
1	gp	consultation via video conference	awaiting review	4,653
1	nurse	enterprise consultation	enterprise consultation	4,460
1	other care provider	provision of general practitioner intermediate care	gp surgery	4,369
1	gp	clinic note	surgery consultation	3,823
1	nurse	residential home visit note	residential home visit note	3,612
1	nurse	clinic note	clinic	3,585
1	nurse	nursing home visit note	nursing home visit note	3,528
1	nurse	face to face consultation	surgery consultation	3,442
1	gp	online communication	awaiting review	3,410
1	other care provider	other note	other	3,406
1	other care provider	seen in gp unit	gp surgery	2,781
1	gp	e-mail consultation	awaiting review	2,523
1	nurse	other note	other	2,449
1	other care provider	gp surgery	surgery consultation	2,334
1	other care provider	enterprise consultation	enterprise consultation	2,318
1	other care provider	telephone call from a patient	telephone call from a patient	2,211
1	nurse	telephone call from a patient	telephone call from a patient	2,183
1	gp	routine consultation	awaiting review	2,117
1	nurse	emergency appointment	emergency appointment	2,041
1	gp	home visit note	home visit	2,021
1	gp	seen in gp unit	gp surgery	1,896
1	nurse	provision of general practitioner intermediate care	gp surgery	1,762

For peer review only

1	other care provider	clinic note	clinic	1,699
1	nurse	clinic note	surgery consultation	1,628
1	gp	clinic note	clinic	1,623
1	nurse	routine consultation	surgery consultation	1,578
1	nurse	seen in gp unit	surgery consultation	1,426
1	nurse	walk-in clinic	walk-in clinic	1,412
1	nurse	gp surgery	clinic	1,355
1	other care provider	routine consultation	other	1,303
1	other care provider	clinic note	surgery consultation	1,297
1	gp	face to face consultation	emergency consultation	1,292
1	other care provider	walk-in clinic	walk-in clinic	1,216
1	gp	telephone encounter	telephone encounter	1,184
1	gp	online communication	online services message	1,139
1	gp	other consultation medium used	awaiting review	1,134
1	other care provider	residential home visit note	residential home visit note	1,113
1	nurse	seen in gp unit	gp surgery	1,103
1	other care provider	nursing home visit note	nursing home visit note	1,081
1	other care provider	face to face consultation	surgery consultation	1,045
1	other care provider	seen in gp unit	surgery consultation	1,043
1	nurse	emergency consultation	emergency consultation	1,024
1	nurse	urgent consultation	urgent consultation	959
1	gp	extended hours consultation	awaiting review	924
1	gp	routine consultation	other	922
1	gp	home visit note	other	835
1	gp	gp surgery	face to face consultation	808
1	other care provider	gp surgery	clinic	746
1	other care provider	routine consultation	surgery consultation	738
1	gp	consultation via multimedia	awaiting review	734
1	gp	face to face consultation with relative/carer	awaiting review	669
1	nurse	e-mail consultation	awaiting review	638
1	nurse	routine consultation	awaiting review	574
1	nurse	consultation via video conference	awaiting review	505
1	nurse	routine consultation	other	478
1	other care provider	online communication	awaiting review	473
1	nurse	same day appointment	same day appointment	468
1	gp	face to face consultation	gp surgery	461
1	gp	same day appointment	same day appointment	457
1	nurse	gp surgery	face to face consultation	455
1	gp	telephone encounter	telephone consultation	429
1	nurse	face to face consultation	emergency consultation	420

For peer review only

1	gp	group consultation	awaiting review	402
1	nurse	home visit note	other	402
1	nurse	seen in urgent care centre	awaiting review	344
1	gp	face to face consultation	emergency appointment	330
1	gp	seen in urgent care centre	awaiting review	322
1	nurse	telephone encounter	telephone encounter	314
1	other care provider	consultation via video conference	awaiting review	288
1	gp	telephone consultation	telephone call to a patient	287
1	dr	telephone consultation	telephone consultation	279
1	nurse	online communication	awaiting review	279
1	other care provider	face to face consultation	awaiting review	272
1	other care provider	home visit note	home visit	262
1	other care provider	routine consultation	awaiting review	260
1	nurse	home visit note	home visit	246
1	gp	consultation via sms text message	awaiting review	244
1	other care provider	online communication	online services message	241
1	other care provider	emergency consultation	emergency consultation	235
1	other care provider	emergency appointment	emergency appointment	231
1	gp	telephone encounter	telephone call to a patient	226
1	gp	residential home visit note	residential home	225
1	gp	face to face consultation	awaiting review	224
1	nurse	face to face consultation	gp surgery	221
1	other care provider	gp surgery	face to face consultation	209
1	nurse	extended hours consultation	awaiting review	186
1	gp	seen in rapid access clinic at gp surgery	awaiting review	182
1	gp	school visit note	awaiting review	181
1	other care provider	telephone consultation	telephone call to a patient	179
1	nurse	face to face consultation	awaiting review	166
1	gp	home visit note	acute visit	153
1	nurse	online communication	online services message	145
1	other care provider	group consultation	awaiting review	133
1	gp	seen in gp unit	awaiting review	129
1	dr	clinic note	surgery consultation	125
1	other care provider	telephone encounter	telephone encounter	119
1	other care provider	extended hours consultation	awaiting review	116
1	nurse	face to face consultation with relative/carer	awaiting review	115
1	nurse	seen in gp unit	awaiting review	109
1	gp	night visit note	awaiting review	108

For peer review only

1	nurse	school visit note	awaiting review	107
1	nurse	group consultation	awaiting review	102
1	gp	other consultation medium used	other	99
1	gp	children's home visit note	awaiting review	95
1	gp	telephone consultation	telephone call from a patient	94
1	gp	administration note	other	91
1	nurse	telephone encounter	telephone call to a patient	86
1	other care provider	e-mail consultation	awaiting review	71
1	nurse	consultation via multimedia	awaiting review	65
1	gp	gp surgery	clinic	63
1	nurse	telephone consultation	telephone call to a patient	63
1	other care provider	face to face consultation with relative/carer	awaiting review	62
1	gp	consultation via telemedicine web camera	awaiting review	61
1	dr	telephone call to a patient	telephone call to a patient	60
1	nurse	laboratory result	clinic	55
1	other care provider	other consultation medium used	awaiting review	53
1	nurse	other consultation medium used	awaiting review	51
1	nurse	district nurse visit	awaiting review	48
1	gp	clinic note	gp surgery	47
1	other care provider	clinic note	gp surgery	45
1	other care provider	urgent consultation	urgent consultation	42
1	other care provider	home visit note	other	41
1	other care provider	laboratory result	clinic	40
1	gp	laboratory result	acute visit	39
1	dr	home visit note	home visit note	38
1	other care provider	telephone encounter	telephone call to a patient	38
1	nurse	telephone encounter	telephone consultation	33
1	other care provider	consultation via sms text message	awaiting review	28
1	gp	walk-in clinic	walk-in centre	27
1	nurse	walk-in clinic	walk-in centre	27
1	other care provider	walk-in clinic	clinic	27
1	nurse	children's home visit note	awaiting review	25
1	gp	gp surgery	main surgery	24
1	nurse	residential home visit note	residential home	22
1	other care provider	seen in urgent care centre	awaiting review	22
1	nurse	night visit note	awaiting review	20
1	other care provider	children's home visit note	awaiting review	19
1	other care provider	consultation via multimedia	awaiting review	19

For peer review only

1	gp	home visit note	nursing home visit note	17
1	gp	nursing home visit note	nursing home	17
1	gp	residential home visit note	nursing home	15
1	other care provider	face to face consultation	gp surgery	14
1	other care provider	night visit note	awaiting review	14
1	other care provider	other consultation medium used	other	14
1	nurse	clinic note	gp surgery	13
1	nurse	walk-in clinic	clinic	13
1	nurse	home visit note	acute visit	10
1	nurse	consultation via sms text message	awaiting review	< 10
1	nurse	consultation via telemedicine web camera	awaiting review	< 10
1	dr	consultation via video conference	awaiting review	< 10
1	dr	nursing home visit note	nursing home visit note	< 10
1	other care provider	home visit note	nursing home visit note	< 10
1	dr	other note	other	< 10
1	gp	telephone encounter	telephone call from a patient	< 10
1	gp	twilight visit note	awaiting review	< 10
1	other care provider	laboratory result	acute visit	< 10
1	dr	face to face consultation	face to face consultation	< 10
1	gp	home visit note	follow-up/routine visit	< 10
1	gp	other consultation medium used	casualty attendance	< 10
1	other care provider	home visit note	residential home visit note	< 10
1	other care provider	other note	gp surgery	< 10
1	gp	home visit note	awaiting review	< 10
1	gp	non-consultation medication data	awaiting review	< 10
1	gp	remote consultation	awaiting review	< 10
1	gp	third party consultation	casualty attendance	< 10
1	nurse	home visit note	follow-up/routine visit	< 10
1	nurse	telephone encounter	telephone call from a patient	< 10
1	nurse	third party consultation	casualty attendance	< 10
1	dr	enterprise consultation	enterprise consultation	< 10
1	dr	telephone call from a patient	telephone call from a patient	< 10
1	gp	district nurse visit	awaiting review	< 10
1	gp	e-mail received from patient	acute visit	< 10
1	gp	hospital outpatient report	casualty attendance	< 10
1	gp	joint consultation	awaiting review	< 10
1	gp	pharmacy consultation	awaiting review	< 10
1	gp	telephone call to a patient	telephone consultation	< 10
1	nurse	administration note	other	< 10
1	nurse	face to face consultation	emergency appointment	< 10
1	other care provider	consultation via telemedicine web camera	awaiting review	< 10

For peer review only

1	other care provider	face to face consultation	emergency appointment	< 10
1	other care provider	face to face consultation	emergency consultation	< 10
1	other care provider	home visit note	acute visit	< 10
1	other care provider	pharmacy consultation	awaiting review	< 10
1	other care provider	residential home visit note	residential home	< 10
1	other care provider	telephone encounter	telephone consultation	< 10
1	dr	group consultation	awaiting review	< 10
1	dr	home visit note	acute visit	< 10
1	gp	clinic note	follow-up/routine visit	< 10
1	gp	emergency consultation	casualty attendance	< 10
1	gp	home visit note	nursing home	< 10
1	nurse	seen in rapid access clinic at gp surgery	awaiting review	< 10
1	nurse	telephone consultation	telephone call from a patient	< 10
1	nurse	twilight visit note	awaiting review	< 10
1	other care provider	administration note	other	< 10
1	other care provider	children's home visit note	clinic	< 10
1	other care provider	home visit note	awaiting review	< 10
1	other care provider	twilight visit note	awaiting review	< 10
excl. job cat		gp surgery	gp surgery	875,291
excl. job cat		other note	other	31,332
excl. job cat		telephone consultation	telephone consultation	29,455
excl. job cat		online communication	online services message	14,055
excl. job cat		telephone call to a patient	telephone call to a patient	13,935
excl. job cat		provision of general practitioner intermediate care	gp surgery	9,791
excl. job cat		telephone call from a patient	telephone call from a patient	7,098
excl. job cat		seen in gp unit	gp surgery	6,240
excl. job cat		home visit note	home visit note	4,776
excl. job cat		routine consultation	other	4,248
excl. job cat		gp surgery	surgery consultation	3,047
excl. job cat		home visit note	other	2,664
excl. job cat		face to face consultation	face to face consultation	2,071
excl. job cat		face to face consultation	surgery consultation	1,177
excl. job cat		online communication	awaiting review	1,098
excl. job cat		nursing home visit note	nursing home visit note	708
excl. job cat		routine consultation	surgery consultation	509
excl. job cat		consultation via video conference	awaiting review	494
excl. job cat		routine consultation	awaiting review	453
excl. job cat		enterprise consultation	enterprise consultation	398
excl. job cat		administration note	other	376
excl. job cat		telephone encounter	telephone encounter	376

For peer review only

excl. job cat		home visit note	awaiting review	371
excl. job cat		clinic note	clinic	337
excl. job cat		clinic note	surgery consultation	329
excl. job cat		residential home visit note	residential home visit note	327
excl. job cat		face to face consultation	gp surgery	305
excl. job cat		face to face consultation	awaiting review	293
excl. job cat		home visit note	home visit	250
excl. job cat		urgent consultation	urgent consultation	240
excl. job cat		walk-in clinic	walk-in clinic	196
excl. job cat		group consultation	awaiting review	170
excl. job cat		seen in gp unit	surgery consultation	148
excl. job cat		e-mail consultation	awaiting review	145
excl. job cat		emergency consultation	emergency consultation	121
excl. job cat		face to face consultation with relative/carer	awaiting review	116
excl. job cat		consultation via multimedia	awaiting review	75
excl. job cat		seen in gp unit	awaiting review	75
excl. job cat		children's home visit note	awaiting review	63
excl. job cat		other note	gp surgery	54
excl. job cat		other consultation medium used	other	44
excl. job cat		other consultation medium used	awaiting review	42
excl. job cat		extended hours consultation	awaiting review	37
excl. job cat		gp surgery	face to face consultation	36
excl. job cat		emergency appointment	emergency appointment	35
excl. job cat		gp surgery	clinic	32
excl. job cat		face to face consultation	emergency consultation	25
excl. job cat		residential home visit note	residential home	23
excl. job cat		telephone encounter	telephone call to a patient	23
excl. job cat		night visit note	awaiting review	14
excl. job cat		home visit note	acute visit	13
excl. job cat		walk-in clinic	walk-in centre	11
excl. job cat		district nurse visit	awaiting review	< 10
excl. job cat		seen in urgent care centre	awaiting review	< 10
excl. job cat		twilight visit note	awaiting review	< 10
excl. job cat		clinic note	gp surgery	< 10
excl. job cat		laboratory result	acute visit	< 10
excl. job cat		telephone consultation	telephone call to a patient	< 10
excl. job cat		walk-in clinic	clinic	< 10
excl. job cat		telephone encounter	telephone call from a patient	< 10
excl. job cat		emergency consultation	casualty attendance	< 10
excl. job cat		pharmacy consultation	awaiting review	< 10
excl. job cat		third party consultation	casualty attendance	< 10
excl. job cat		case conference	gp surgery	< 10
excl. job cat		emergency consultation	awaiting review	< 10
excl. job cat		gp surgery	main surgery	< 10
excl. job cat		home visit note	follow-up/routine visit	< 10
excl. job cat		non-consultation medication data	casualty attendance	< 10
excl. job cat		nursing home visit note	nursing home	< 10

For peer review only

excl. job cat		remote consultation	awaiting review	< 10
excl. job cat		same day appointment	same day appointment	< 10
excl. job cat		school visit note	awaiting review	< 10
excl. job cat		telephone consultation	awaiting review	< 10
excl. job cat		telephone encounter	telephone consultation	< 10
excl. job cat		third party consultation	awaiting review	< 10
0	gp	externally entered note	externally entered	3,467,397
0		scanned document	docman	3,183,781
0		administration note	administration note	968,767
0				737,843
0		scanned document	scanned document	727,269
0	gp	administration note	administration note	725,612
0		inbound document	inbound document	402,647
0		awaiting clinical code migration to emis web	awaiting review	385,598
0	nurse	externally entered note	externally entered	303,830
0	gp	awaiting clinical code migration to emis web	awaiting review	261,627
0		administration note	administration	256,604
0	other care provider	scanned document	docman	255,022
0		externally entered note	externally entered	252,167
0	gp			208,462
0	gp	outbound referral	outbound referral	197,534
0	gp	awaiting clinical code migration to emis web	gp surgery	162,048
0	other care provider	administration note	administration note	160,918
0		awaiting clinical code migration to emis web	third party consultation	147,374
0	gp	scanned document	scanned document	145,355
0		awaiting clinical code migration to emis web	gp surgery	127,041
0	gp	awaiting clinical code migration to emis web	results recording	122,118
0		awaiting clinical code migration to emis web	other	121,401
0	gp	awaiting clinical code migration to emis web	surgery consultation	107,304
0		awaiting clinical code migration to emis web		104,693
0	gp	inbound document	inbound document	102,534
0	nurse	awaiting clinical code migration to emis web	awaiting review	94,158
0	other care provider	awaiting clinical code migration to emis web	gp surgery	86,398
0	other care provider	awaiting clinical code migration to emis web	awaiting review	85,745
0	nurse	administration note	administration note	84,051
0	gp	awaiting clinical code migration to emis web		77,585
0	nurse	awaiting clinical code migration to emis web	gp surgery	75,552

For peer review only

0	other care provider			66,366
0	other care provider	externally entered note	externally entered	62,810
0		externally entered note	externally entered note	61,848
0		awaiting clinical code migration to emis web	repeat issue	61,683
0	nurse			58,019
0	gp	telephone triage encounter	telephone triage encounter	50,662
0		third party consultation	third party consultation	44,609
0	other care provider	scanned document	scanned document	38,341
0	nurse	awaiting clinical code migration to emis web		35,376
0	nurse	awaiting clinical code migration to emis web	surgery consultation	27,699
0	gp	administration note	administration	27,390
0	gp	awaiting clinical code migration to emis web	telephone consultation	26,214
0	gp	awaiting clinical code migration to emis web	other	26,204
0		awaiting clinical code migration to emis web	scanned document	25,627
0		awaiting clinical code migration to emis web	administration note	24,458
0		gp surgery	awaiting review	24,337
0		awaiting clinical code migration to emis web	touchscreen	24,311
0	gp	third party consultation	third party consultation	23,952
0		awaiting clinical code migration to emis web	patientchase insert	22,515
0	gp	laboratory result	results recording	22,066
0	gp	externally entered note	externally entered note	20,864
0	gp	awaiting clinical code migration to emis web	administration note	20,729
0	other care provider	inbound document	inbound document	20,159
0	gp	telephone call to relative/carer	telephone call to relative/carer	19,234
0	other care provider	awaiting clinical code migration to emis web		17,081
0		awaiting clinical code migration to emis web	surgery consultation	15,972
0	nurse	awaiting clinical code migration to emis web	clinic	15,892
0	gp	other note	other note	15,816
0	gp	gp surgery	awaiting review	15,810
0	gp	discussion with colleague	discussion with colleague	12,973
0	gp	awaiting clinical code migration to emis web	telephone call to a patient	11,782
0		repeat prescription	repeat issue	11,542
0	other care provider	administration note	administration	11,037
0	nurse	awaiting clinical code migration to emis web	other	11,008

For peer review only

0		hospital outpatient report	hospital outpatient report	10,033
0	nurse	inbound document	inbound document	9,304
0	nurse	scanned document	scanned document	7,878
0	other care provider	awaiting clinical code migration to emis web	repeat issue	7,745
0	gp	awaiting clinical code migration to emis web	outbound referral	7,402
0	other care provider	externally entered note	externally entered note	6,736
0	gp	telephone call from relative/carer	telephone call from relative/carer	6,626
0	other care provider	awaiting clinical code migration to emis web	surgery consultation	6,514
0		awaiting clinical code migration to emis web	inbound document	6,495
0	nurse	gp surgery	awaiting review	6,475
0	other care provider	awaiting clinical code migration to emis web	other	6,315
0	nurse	administration note	administration	6,285
0		outbound referral	outbound referral	6,194
0	nurse	telephone triage encounter	telephone triage encounter	6,074
0	gp	clinic note	clinic note	5,834
0	gp	awaiting clinical code migration to emis web	repeat issue	5,803
0	gp	non-consultation data	non-consultation data	5,552
0	other care provider	third party consultation	third party consultation	5,505
0	other care provider	gp surgery	awaiting review	5,332
0	gp	awaiting clinical code migration to emis web	telephone call from a patient	5,282
0	nurse	externally entered note	externally entered note	5,251
0	gp	face to face consultation	triage	5,196
0	nurse	telephone call to relative/carer	telephone call to relative/carer	5,191
0		awaiting clinical code migration to emis web	mail to patient	5,178
0		non-consultation data	non-consultation data	4,933
0	gp	e-mail received from patient	e-mail received from patient	4,877
0	nurse	outbound referral	outbound referral	4,787
0		medication requested	repeat issue	4,639
0	nurse	third party consultation	third party consultation	4,605
0	nurse	awaiting clinical code migration to emis web	results recording	4,405
0	gp	awaiting clinical code migration to emis web	scanned document	4,384
0		other note	other note	4,375
0		awaiting clinical code migration to emis web	mjog	4,221
0	other care provider	other note	other note	4,071
0		mail to patient	mail to patient	3,924
0	other care provider	awaiting clinical code migration to emis web	clinic	3,859
0		e-mail received from patient	e-mail received from patient	3,632

For peer review only

0		awaiting clinical code migration to emis web	out of hours, non practice	3,607
0	other care provider	clinic note	clinic note	3,371
0	gp	awaiting clinical code migration to emis web	home visit note	3,370
0	gp	hospital outpatient report	hospital outpatient report	3,359
0	nurse	nurse telephone triage	nurse telephone triage	3,274
0	nurse	clinic note	clinic note	3,252
0	gp	awaiting clinical code migration to emis web	clinic	3,245
0	other care provider	awaiting clinical code migration to emis web	administration note	3,044
0	gp	awaiting clinical code migration to emis web	third party consultation	3,038
0	other care provider	awaiting clinical code migration to emis web	medicine management	2,829
0	other care provider	telephone call to relative/carer	telephone call to relative/carer	2,807
0	nurse	awaiting clinical code migration to emis web	telephone consultation	2,782
0		awaiting clinical code migration to emis web	clinic	2,775
0		laboratory result	laboratory result	2,727
0	gp	awaiting clinical code migration to emis web	inbound document	2,605
0		telephone call from relative/carer	telephone call from relative/carer	2,594
0		clinic note	clinic note	2,579
0	gp	laboratory result	laboratory result	2,511
0	other care provider	repeat prescription	repeat issue	2,511
0		awaiting clinical code migration to emis web	home of patient	2,454
0	gp	awaiting clinical code migration to emis web	out of hours, non practice	2,365
0	gp	awaiting clinical code migration to emis web	nhs direct report	2,334
0		awaiting clinical code migration to emis web	results recording	2,185
0	dr	third party consultation	third party consultation	2,146
0	other care provider	awaiting clinical code migration to emis web	third party consultation	2,095
0	nurse	awaiting clinical code migration to emis web	administration note	2,075
0		administration note	patientchase insert	1,855
0		clinic note	awaiting review	1,790
0		hospital inpatient report	hospital inpatient report	1,760
0		e-mail received from patient	docman	1,753
0	nurse	other note	other note	1,708
0	nurse	non-consultation data	non-consultation data	1,681
0	gp	awaiting clinical code migration to emis web	referral letter	1,660
0		laboratory result	results recording	1,624

For peer review only

0		awaiting clinical code migration to emis web	non-consultation data	1,537
0	gp	awaiting clinical code migration to emis web	medicine management	1,510
0	nurse	laboratory result	results recording	1,464
0	nurse	telephone call from relative/carer	telephone call from relative/carer	1,401
0		administration note	scanned document	1,373
0		telephone triage encounter	telephone triage encounter	1,353
0	gp	medication requested	awaiting review	1,347
0	nurse	discussion with colleague	discussion with colleague	1,329
0	gp	awaiting clinical code migration to emis web	telephone triage encounter	1,313
0	nurse	awaiting clinical code migration to emis web	telephone call to a patient	1,243
0	other care provider	non-consultation data	non-consultation data	1,239
0	gp	case conference	awaiting review	1,225
0	gp	awaiting clinical code migration to emis web	externally entered note	1,206
0		telephone call to relative/carer	telephone call to relative/carer	1,191
0	nurse	awaiting clinical code migration to emis web	third party consultation	1,170
0	gp	e-mail sent to patient	awaiting review	1,162
0	dr	administration note	administration	1,159
0		other note		1,156
0		administration note		1,120
0	dr	externally entered note	externally entered	1,113
0	gp	awaiting clinical code migration to emis web	face to face consultation	1,112
0	other care provider	telephone triage encounter	telephone triage encounter	1,107
0	nurse	laboratory result	laboratory result	1,064
0	other care provider	outbound referral	outbound referral	1,052
0	gp	clinic note	awaiting review	1,030
0	other care provider	mail to patient	patientchase insert	1,019
0	gp	repeat prescription	awaiting review	1,003
0	gp	awaiting clinical code migration to emis web	data transferred from other system	983
0		awaiting clinical code migration to emis web	non-consultation medication data	980
0	gp	awaiting clinical code migration to emis web	nursing home visit note	971
0	gp	repeat prescription	repeat issue	964
0		awaiting clinical code migration to emis web	telephone call to a patient	901
0	dr			885
0	nurse	awaiting clinical code migration to emis web	face to face consultation	853
0	other care provider	hospital outpatient report	hospital outpatient report	828
0		clinic note	community clinic	823

For peer review only

0		awaiting clinical code migration to emis web	gp2gp import	822
0		awaiting clinical code migration to emis web	letter from outpatients	809
0	other care provider	mail to patient	mail to patient	801
0	other care provider	telephone call from relative/carer	telephone call from relative/carer	789
0		awaiting clinical code migration to emis web	externally entered note	769
0		awaiting clinical code migration to emis web	referral letter	751
0	other care provider	awaiting clinical code migration to emis web	inbound document	719
0		e-mail sent to patient	awaiting review	711
0	gp	hospital inpatient report	hospital inpatient report	710
0	nurse	awaiting clinical code migration to emis web	repeat issue	694
0	gp	awaiting clinical code migration to emis web	acute visit	693
0	gp	awaiting clinical code migration to emis web	telephone call to relative/carer	680
0		non-consultation medication data	repeat issue	679
0	nurse	awaiting clinical code migration to emis web	home visit note	672
0	other care provider	discussion with colleague	discussion with colleague	665
0		administration note	docman	665
0	gp	other note		661
0		awaiting clinical code migration to emis web	telephone consultation	656
0	other care provider	awaiting clinical code migration to emis web	scanned document	642
0		ooh report	third party consultation	637
0	gp	scanned document	externally entered	619
0	gp	medication requested	repeat issue	606
0	nurse	awaiting clinical code migration to emis web	telephone triage encounter	602
0	other care provider	awaiting clinical code migration to emis web	telephone call to a patient	541
0	other care provider	awaiting clinical code migration to emis web	face to face consultation	534
0	dr	administration note	administration note	525
0	gp	discussion with other professional	awaiting review	514
0	gp	administration note	awaiting review	509
0	other care provider	awaiting clinical code migration to emis web	mail to patient	507
0		ooh report	awaiting review	504
0	dr	awaiting clinical code migration to emis web	gp surgery	501
0		walk-in clinic		495
0		administration note	inbound document	493
0		routine consultation	repeat issue	488

0		awaiting clinical code migration to emis web	telephone call from a patient	486
0	gp	ooh report	out of hours, non practice	478
0	gp	mail from patient	mail from patient	473
0		other note	awaiting review	472
0	gp	awaiting clinical code migration to emis web	enterprise consultation	458
0	other care provider	repeat prescription	awaiting review	455
0	gp	awaiting clinical code migration to emis web	discussion with colleague	451
0		awaiting clinical code migration to emis web	outbound referral	450
0	gp	awaiting clinical code migration to emis web	nursing home	447
0	dr	awaiting clinical code migration to emis web	awaiting review	444
0	nurse	e-mail received from patient	e-mail received from patient	443
0		awaiting clinical code migration to emis web	data transferred from other system	442
0	nurse	ooh report	awaiting review	439
0		administration note	awaiting review	436
0	other care provider	clinic note	awaiting review	431
0	gp	administration note		415
0	gp	administration note	scanned document	414
0	gp	awaiting clinical code migration to emis web	urgent consultation	404
0	other care provider	awaiting clinical code migration to emis web	externally entered note	385
0		awaiting clinical code migration to emis web	school	384
0	nurse	hospital outpatient report	hospital outpatient report	380
0		mail to patient	patientchase insert	377
0		awaiting clinical code migration to emis web	telephone encounter	376
0	gp	administration note	results recording	372
0		discussion with colleague	discussion with colleague	368
0	other care provider	awaiting clinical code migration to emis web	results recording	365
0		case conference	awaiting review	361
0	other care provider	awaiting clinical code migration to emis web	telephone consultation	350
0	other care provider	awaiting clinical code migration to emis web	patientchase insert	345
0		sms text message sent to patient	patientchase insert	345
0	nurse	mail to patient	mail to patient	342
0		awaiting clinical code migration to emis web	hospital outpatient report	336
0		gp surgery		315
0	gp	ooh report	nhs direct report	314
0	gp	administration note	referral letter	313
0	dr	awaiting clinical code migration to emis web		310

For peer review only

0	gp	awaiting clinical code migration to emis web	telephone call from relative/carer	310
0	gp	awaiting clinical code migration to emis web	non-consultation data	309
0	nurse	awaiting clinical code migration to emis web	telephone call from a patient	309
0	other care provider	awaiting clinical code migration to emis web	non-consultation data	306
0	other care provider	e-mail received from patient	e-mail received from patient	292
0	other care provider	awaiting clinical code migration to emis web	non-consultation medication data	289
0	nurse	clinic note	awaiting review	286
0	dr	scanned document	scanned document	285
0		ooh report	out of hours, non practice	283
0	other care provider	laboratory result	laboratory result	282
0	gp	awaiting clinical code migration to emis web	discharge details	281
0		home visit note	home of patient	277
0	nurse	awaiting clinical code migration to emis web	inbound document	276
0	other care provider	hospital inpatient report	hospital inpatient report	275
0	gp	mail to patient	mail to patient	270
0	gp	awaiting clinical code migration to emis web	residential home visit note	266
0	other care provider	administration note	awaiting review	265
0		awaiting clinical code migration to emis web	acute visit	263
0		walk-in clinic	awaiting review	260
0	dr	hospital outpatient report	hospital outpatient report	256
0	gp	awaiting clinical code migration to emis web	mail to patient	249
0		administration note	mjog	248
0		mail from patient	mail from patient	248
0	gp	awaiting clinical code migration to emis web	letter from outpatients	247
0		awaiting clinical code migration to emis web	discharge details	244
0	nurse	awaiting clinical code migration to emis web	mail to patient	243
0	other care provider	awaiting clinical code migration to emis web	home visit note	236
0	gp	home visit note	results recording	235
0	dr	inbound document	inbound document	234
0	gp	awaiting clinical code migration to emis web	other note	230
0	gp	awaiting clinical code migration to emis web	patientchase insert	229
0	dr	awaiting clinical code migration to emis web	administration note	225
0	gp	awaiting clinical code migration to emis web	open door surgery	222

For peer review only

0		awaiting clinical code migration to emis web	nhs direct report	222
0	other care provider	awaiting clinical code migration to emis web	touchscreen	216
0	gp	scanned document	docman	213
0	nurse	awaiting clinical code migration to emis web	nursing home visit note	210
0	nurse	administration note		209
0	nurse	awaiting clinical code migration to emis web	externally entered note	209
0		awaiting clinical code migration to emis web	laboratory result	199
0		hospital outpatient report	hospital	197
0	other care provider	non-consultation medication data	repeat issue	196
0	other care provider	awaiting clinical code migration to emis web	referral letter	195
0	nurse	e-mail sent to patient	awaiting review	191
0	nurse	awaiting clinical code migration to emis web	referral letter	190
0	dr	awaiting clinical code migration to emis web	telephone consultation	186
0	nurse	case conference	awaiting review	185
0	other care provider	awaiting clinical code migration to emis web	data transferred from other system	184
0	gp	multidisciplinary team meeting without patient	awaiting review	179
0	gp	awaiting clinical code migration to emis web	clinic note	176
0	other care provider	awaiting clinical code migration to emis web	mjog	175
0		awaiting clinical code migration to emis web	hospital inpatient report	168
0		awaiting clinical code migration to emis web	home visit note	164
0		awaiting clinical code migration to emis web	online services message	163
0	other care provider	administration note		162
0	nurse	awaiting clinical code migration to emis web	outbound referral	159
0	other care provider	ooh report	awaiting review	156
0	gp	awaiting clinical code migration to emis web	non-consultation medication data	152
0	nurse	awaiting clinical code migration to emis web	telephone call to relative/carer	152
0	other care provider	other note	awaiting review	149
0	dr	awaiting clinical code migration to emis web	surgery consultation	148
0	nurse	awaiting clinical code migration to emis web	scanned document	146
0	gp	administration note	repeat issue	145
0		awaiting clinical code migration to emis web	open door surgery	144

For peer review only

0	other care provider	awaiting clinical code migration to emis web	clinic note	140
0		inbound referral	awaiting review	138
0		awaiting clinical code migration to emis web	clinic note	133
0		awaiting clinical code migration to emis web	face to face consultation	129
0	other care provider	non-consultation medication data	non-consultation medication data	124
0	nurse	awaiting clinical code migration to emis web	residential home visit note	122
0	nurse	awaiting clinical code migration to emis web	data transferred from other system	121
0	other care provider	e-mail sent to patient	awaiting review	121
0	gp	administration note	outbound referral	118
0	nurse	other note		117
0	nurse	awaiting clinical code migration to emis web	nurse telephone triage	116
0		awaiting clinical code migration to emis web	accident & emergency	116
0	gp	other consultation medium used	data transferred from other system	109
0		non-consultation medication data	non-consultation medication data	108
0	nurse	face to face consultation	triage	104
0	other care provider	awaiting clinical code migration to emis web	acute visit	102
0	nurse	awaiting clinical code migration to emis web	telephone call from relative/carer	100
0		extended hours consultation	out of hours, non practice	100
0	nurse	mail from patient	mail from patient	98
0	other care provider	hospital outpatient report	hospital	97
0	dr	awaiting clinical code migration to emis web	nursing home visit note	96
0		nurse telephone triage	nurse telephone triage	94
0	nurse	hospital inpatient report	hospital inpatient report	93
0	other care provider	case conference	awaiting review	92
0	gp	provision of general practitioner intermediate care	awaiting review	91
0	gp	walk-in clinic		91
0		hospital outpatient report	letter from outpatients	91
0	other care provider	other note		89
0	gp	administration note	other note	87
0	other care provider	medication requested	awaiting review	85
0	dr	laboratory result	results recording	84
0		awaiting clinical code migration to emis web	mail from patient	84
0		awaiting clinical code migration to emis web	health centre	80
0	gp	administration note	inbound document	76
0	gp	other note	awaiting review	76

For peer review only

0	nurse	walk-in clinic		76
0		other note	non-consultation medication data	76
0	other care provider	awaiting clinical code migration to emis web	nhs direct report	73
0		administration note	repeat issue	73
0	gp	ooh report	awaiting review	70
0	gp	weekly care home ward round	awaiting review	70
0		awaiting clinical code migration to emis web	medicine management	70
0	other care provider	awaiting clinical code migration to emis web	open door surgery	69
0	other care provider	awaiting clinical code migration to emis web	telephone encounter	69
0		medication requested	awaiting review	68
0	other care provider	awaiting clinical code migration to emis web	telephone call to relative/carer	65
0	dr	awaiting clinical code migration to emis web	repeat issue	64
0	gp	non-consultation medication data	repeat issue	64
0	other care provider	awaiting clinical code migration to emis web	telephone call from a patient	64
0	gp	clinic note	nhs direct report	62
0	gp	non-consultation medication data	medicine management	61
0	other care provider	awaiting clinical code migration to emis web	letter from outpatients	61
0	gp	hospital outpatient report	letter from outpatients	60
0	gp	non-consultation data	data transferred from other system	57
0	nurse	awaiting clinical code migration to emis web	enterprise consultation	57
0	gp	outbound referral	referral letter	55
0	other care provider	repeat prescription	medicine management	55
0	nurse	seen in influenza vaccination clinic	awaiting review	54
0		administration note	results recording	54
0		awaiting clinical code migration to emis web	telephone call from relative/carer	54
0	gp	awaiting clinical code migration to emis web	hospital outpatient report	53
0	gp	gp surgery		53
0	gp	third party consultation	out of hours, non practice	51
0		awaiting clinical code migration to emis web	telephone call to relative/carer	51
0		clinic note	out of hours, non practice	51
0		face to face consultation	primary care centre	51
0	gp	awaiting clinical code migration to emis web	laboratory result	50
0	nurse	non-consultation medication data	repeat issue	48
0		awaiting clinical code migration to emis web	other note	48
0		face to face consultation	triage	48
0	gp	progress report	nhs direct report	47
0	other care provider	ooh report	nhs direct report	47

For peer review only

0	nurse	awaiting clinical code migration to emis web	non-consultation data	46
0		awaiting clinical code migration to emis web	nursing home	46
0	gp	awaiting clinical code migration to emis web	mail from patient	45
0	nurse	awaiting clinical code migration to emis web	discussion with colleague	45
0		awaiting clinical code migration to emis web	e-mail received from patient	45
0	nurse	awaiting clinical code migration to emis web	medicine management	43
0	nurse	medication requested	repeat issue	42
0	dr	mail to patient	mail to patient	40
0	gp	awaiting clinical code migration to emis web	encompass message	39
0	gp	other note	data transferred from other system	39
0	nurse	awaiting clinical code migration to emis web	clinic note	39
0	nurse	awaiting clinical code migration to emis web	laboratory result	39
0	other care provider	awaiting clinical code migration to emis web	other note	39
0	other care provider	medication requested	repeat issue	38
0	gp	administration note	non-consultation data	37
0	other care provider	mail from patient	mail from patient	37
0	dr	awaiting clinical code migration to emis web	results recording	36
0	gp	awaiting clinical code migration to emis web	e-mail received from patient	36
0	nurse	awaiting clinical code migration to emis web	walk-in centre	36
0	other care provider	awaiting clinical code migration to emis web	gp2gp import	36
0		administration note	mail to patient	36
0	gp	awaiting clinical code migration to emis web	home of patient	35
0	gp	awaiting clinical code migration to emis web	casualty attendance	34
0	gp	inbound document	letter from outpatients	34
0	other care provider	awaiting clinical code migration to emis web	outbound referral	34
0	other care provider	extended hours consultation	out of hours, non practice	34
0	gp	awaiting clinical code migration to emis web	telephone encounter	33
0	gp	seen in hospital ward	awaiting review	33
0		other note	non-consultation data	33
0	nurse	other note	awaiting review	32
0	other care provider	awaiting clinical code migration to emis web	discussion with colleague	32
0		discussion with other professional	awaiting review	32

For peer review only

0		multidisciplinary team meeting without patient	awaiting review	32
0		children's home visit note		31
0	other care provider	laboratory result	results recording	30
0		awaiting clinical code migration to emis web	casualty attendance	30
0		extended hours consultation	nhs direct report	30
0	nurse	administration note	awaiting review	29
0	nurse	awaiting clinical code migration to emis web	letter from outpatients	29
0	nurse	awaiting clinical code migration to emis web	open door surgery	29
0	nurse	discussion with other professional	awaiting review	29
0	nurse	ooh report	out of hours, non practice	29
0		face to face consultation	treatment room	29
0	gp	email received from carer	awaiting review	28
0	other care provider	awaiting clinical code migration to emis web	telephone triage encounter	28
0		administration note	touchscreen	28
0	nurse	awaiting clinical code migration to emis web	encompass message	27
0	nurse	awaiting clinical code migration to emis web	nursing home	27
0		third party consultation	out of hours, non practice	27
0	gp	awaiting clinical code migration to emis web	follow-up/routine visit	26
0	other care provider	emergency consultation	accident & emergency	26
0	gp	hospital outpatient report	nhs direct report	25
0	other care provider	awaiting clinical code migration to emis web	telephone call from relative/carer	23
0	other care provider	outbound referral	referral letter	23
0		externally entered note	scanned document	23
0	nurse	multidisciplinary team meeting without patient	awaiting review	22
0		other consultation medium used	data transferred from other system	22
0	dr	awaiting clinical code migration to emis web	outbound referral	21
0	gp	seen in influenza vaccination clinic	awaiting review	21
0	nurse	awaiting clinical code migration to emis web	other note	21
0		ooh report	nhs direct report	21
0	dr	awaiting clinical code migration to emis web	inbound document	20
0	gp	awaiting clinical code migration to emis web	day case report	20
0	other care provider	seen by general practitioner with special interest in ear nose and throat disorders	data transferred from other system	20
0		emergency consultation	accident & emergency	20
0	gp	awaiting clinical code migration to emis web	online services message	19

For peer review only

0	gp	hospital outpatient report	third party consultation	19
0	nurse	repeat prescription	awaiting review	19
0		awaiting clinical code migration to emis web	walk in centre	19
0	dr	hospital inpatient report	hospital inpatient report	18
0	other care provider	awaiting clinical code migration to emis web	residential home visit note	18
0	other care provider	walk-in clinic		18
0		awaiting clinical code migration to emis web	residential home visit note	18
0		awaiting clinical code migration to emis web	telephone triage encounter	18
0	dr	outbound referral	outbound referral	17
0	gp	ooh report	third party consultation	17
0	gp	other consultation medium used	nhs direct report	17
0	gp	radiology result	awaiting review	17
0	gp	telephone consultation	telephone call from relative/carer	17
0	other care provider	awaiting clinical code migration to emis web	enterprise consultation	17
0	other care provider	walk-in clinic	walk in centre	17
0		administration note	referral letter	17
0	dr	other note	other note	16
0	other care provider	awaiting clinical code migration to emis web	nursing home visit note	16
0	dr	medication requested	repeat issue	15
0	other care provider	administration note	inbound document	15
0	other care provider	awaiting clinical code migration to emis web	out of hours, non practice	15
0		non-consultation data	data transferred from other system	15
0	other care provider	face to face consultation	triage	15
0	dr	awaiting clinical code migration to emis web	telephone call to a patient	14
0	dr	externally entered note	externally entered note	14
0	gp	hospital inpatient report	discharge details	14
0	gp	seen by general practitioner with special interest in ear nose and throat disorders	data transferred from other system	14
0	other care provider	administration note	scanned document	14
0	other care provider	discussion with other professional	awaiting review	14
0		awaiting clinical code migration to emis web	out of hours, practice	14
0		externally entered note		14
0	dr	telephone call to relative/carer	telephone call to relative/carer	13
0	nurse	awaiting clinical code migration to emis web	nhs direct report	13
0	nurse	awaiting clinical code migration to emis web	out of hours, non practice	13
0		hospital inpatient note	awaiting review	13

For peer review only

0		repeat prescription	awaiting review	13
0	gp	other note	encompass message	12
0	nurse	awaiting clinical code migration to emis web	hospital outpatient report	12
0	other care provider	awaiting clinical code migration to emis web	accident & emergency	12
0	nurse	other note	encompass message	11
0	nurse	repeat prescription	repeat issue	11
0	other care provider	seen in influenza vaccination clinic	awaiting review	11
0		awaiting clinical code migration to emis web	discussion with colleague	11
0		awaiting clinical code migration to emis web	hospital outpatient consultation	11
0		awaiting clinical code migration to emis web	walk-in clinic	11
0		non-consultation medication data	medicine management	11
0		provision of general practitioner intermediate care	awaiting review	10
0	dr	clinic note	awaiting review	< 10
0	other care provider	ooh report	out of hours centre	< 10
0		administration note	non-consultation data	< 10
0		clinic note	nhs direct report	< 10
0		telephone follow-up	awaiting review	< 10
0	gp	administration note	clinic note	< 10
0	gp	progress report	awaiting review	< 10
0	nurse	awaiting clinical code migration to emis web	acute visit	< 10
0	other care provider	multidisciplinary team meeting without patient	awaiting review	< 10
0		administration note	other note	< 10
0	dr	awaiting clinical code migration to emis web	home visit note	< 10
0	gp	hospital inpatient note	day case report	< 10
0	gp	inbound document	discharge details	< 10
0	nurse	awaiting clinical code migration to emis web	follow-up/routine visit	< 10
0	other care provider	awaiting clinical code migration to emis web	discharge details	< 10
0		awaiting clinical code migration to emis web	enterprise consultation	< 10
0		awaiting clinical code migration to emis web	nursing home visit note	< 10
0		email received from carer	awaiting review	< 10
0		ooh report	out of hours, practice	< 10
0		other note	data transferred from other system	< 10
0		outbound referral	referral letter	< 10
0		radiology result	awaiting review	< 10
0	dr	gp surgery	awaiting review	< 10
0	gp	routine consultation	repeat issue	< 10
0	nurse	email received from carer	awaiting review	< 10
0	nurse	non-consultation data	data transferred from other system	< 10

For peer review only

0	nurse	scanned document	docman	< 10
0	other care provider	awaiting clinical code migration to emis web	home of patient	< 10
0	other care provider	hospital outpatient report	letter from outpatients	< 10
0	dr	discussion with colleague	discussion with colleague	< 10
0	gp	administration note	discussion with colleague	< 10
0	gp	awaiting clinical code migration to emis web	out of hours, practice	< 10
0	gp	clinic note	out of hours, non practice	< 10
0	gp	ooh report	out of hours, practice	< 10
0	gp	seen in diabetic clinic	awaiting review	< 10
0	gp	sms text message sent to patient	awaiting review	< 10
0	nurse	administration note	discussion with colleague	< 10
0	other care provider	awaiting clinical code migration to emis web	nursing home	< 10
0	other care provider	awaiting clinical code migration to emis web	online services message	< 10
0		awaiting clinical code migration to emis web	bulk operation	< 10
0		non-consultation data	touchscreen	< 10
0	dr	awaiting clinical code migration to emis web	hospital outpatient report	< 10
0	dr	awaiting clinical code migration to emis web	other	< 10
0	dr	awaiting clinical code migration to emis web	third party consultation	< 10
0	dr	clinic note	clinic note	< 10
0	dr	non-consultation data	non-consultation data	< 10
0	gp	awaiting clinical code migration to emis web	hospital inpatient report	< 10
0	gp	clinic note	community clinic	< 10
0	nurse	administration note	non-consultation data	< 10
0	nurse	awaiting clinical code migration to emis web	non-consultation medication data	< 10
0	other care provider	externally entered note	data transferred from other system	< 10
0	other care provider	gp surgery		< 10
0	other care provider	other note	referral letter	< 10
0		awaiting clinical code migration to emis web	nurseries/playgroup	< 10
0		hospital outpatient report	third party consultation	< 10
0	gp	nurse telephone triage	nurse telephone triage	< 10
0	dr	awaiting clinical code migration to emis web	discussion with colleague	< 10
0	dr	awaiting clinical code migration to emis web	medicine management	< 10
0	dr	awaiting clinical code migration to emis web	residential home visit note	< 10
0	dr	e-mail received from patient	e-mail received from patient	< 10
0	dr	telephone call from relative/carer	telephone call from relative/carer	< 10

For peer review only

0	gp	awaiting clinical code migration to emis web	walk-in centre	< 10
0	gp	extended hours consultation	out of hours, non practice	< 10
0	gp	externally entered note		< 10
0	gp	other consultation medium used	other note	< 10
0	gp	telephone consultation	telephone call to relative/carer	< 10
0	nurse	awaiting clinical code migration to emis web	telephone encounter	< 10
0	nurse	medication requested	awaiting review	< 10
0	nurse	telephone follow-up	awaiting review	< 10
0	other care provider	awaiting clinical code migration to emis web	e-mail received from patient	< 10
0	other care provider	awaiting clinical code migration to emis web	mail from patient	< 10
0	other care provider	awaiting clinical code migration to emis web	out of hours, practice	< 10
0	other care provider	other note	data transferred from other system	< 10
0		awaiting clinical code migration to emis web	day case report	< 10
0		awaiting clinical code migration to emis web	encompass message	< 10
0		e-mail encounter to carer	awaiting review	< 10
0		walk-in clinic	walk in centre	< 10
0	dr	awaiting clinical code migration to emis web	non-consultation data	< 10
0	dr	awaiting clinical code migration to emis web	other note	< 10
0	dr	e-mail sent to patient	awaiting review	< 10
0	gp	email received from third party	awaiting review	< 10
0	gp	hospital inpatient note	awaiting review	< 10
0	gp	night visit note	night visit	< 10
0	gp	other note	third party consultation	< 10
0	gp	telephone encounter	telephone call to relative/carer	< 10
0	gp	telephone follow-up	awaiting review	< 10
0	nurse	administration note	referral letter	< 10
0	nurse	awaiting clinical code migration to emis web	urgent consultation	< 10
0	nurse	seen in asthma clinic	awaiting review	< 10
0	nurse	seen in hospital ward	awaiting review	< 10
0	nurse	telephone consultation	telephone call to relative/carer	< 10
0	other care provider	administration note	other note	< 10
0	other care provider	administration note	referral letter	< 10
0	other care provider	administration note	repeat issue	< 10
0	other care provider	administration note	results recording	< 10
0	other care provider	awaiting clinical code migration to emis web	casualty attendance	< 10
0	other care provider	awaiting clinical code migration to emis web	diabetic clinic	< 10

For peer review only

0	other care provider	awaiting clinical code migration to emis web	encompass message	< 10
0	other care provider	clinic note	nhs direct report	< 10
0	other care provider	seen in diabetic clinic	awaiting review	< 10
0		awaiting clinical code migration to emis web	other report	< 10
0		child in need meeting	awaiting review	< 10
0		hospital inpatient report	hospital inpatient	< 10
0		progress report	nhs direct report	< 10
0		repeat prescription	non-consultation medication data	< 10
0		weekly care home ward round	awaiting review	< 10
0	dr	awaiting clinical code migration to emis web	non-consultation medication data	< 10
0	dr	awaiting clinical code migration to emis web	telephone call from a patient	< 10
0	dr	awaiting clinical code migration to emis web	telephone call from relative/carer	< 10
0	dr	awaiting clinical code migration to emis web	telephone call to relative/carer	< 10
0	gp	awaiting clinical code migration to emis web	night visit , practice	< 10
0	gp	awaiting clinical code migration to emis web	walk-in clinic	< 10
0	gp	non-consultation medication data	non-consultation medication data	< 10
0	gp	other consultation medium used	referral letter	< 10
0	gp	other note	non-consultation data	< 10
0	gp	other note	non-consultation medication data	< 10
0	gp	seen in baby clinic	awaiting review	< 10
0	gp	telephone encounter	telephone call from relative/carer	< 10
0	nurse	administration note	clinic note	< 10
0	nurse	administration note	laboratory result	< 10
0	nurse	awaiting clinical code migration to emis web	casualty attendance	< 10
0	nurse	awaiting clinical code migration to emis web	patientchase insert	< 10
0	nurse	clinic note	community clinic	< 10
0	nurse	extended hours consultation	out of hours, non practice	< 10
0	nurse	externally entered note	data transferred from other system	< 10
0	nurse	first attendance face to face	awaiting review	< 10
0	nurse	hospital inpatient note	awaiting review	< 10
0	nurse	ooh report	out of hours, practice	< 10
0	nurse	other consultation medium used	data transferred from other system	< 10
0	nurse	other note	data transferred from other system	< 10
0	nurse	other note	referral letter	< 10
0	nurse	outbound referral	referral letter	< 10
0	nurse	patient initiated enc. nos	awaiting review	< 10
0	nurse	telephone consultation	telephone call from relative/carer	< 10
0	nurse	telephone encounter	telephone call to relative/carer	< 10
0	nurse	weekly care home ward round	awaiting review	< 10

For peer review only

0	other care provider	administration note	mail to patient	< 10
0	other care provider	awaiting clinical code migration to emis web	hospital inpatient report	< 10
0	other care provider	awaiting clinical code migration to emis web	hospital outpatient report	< 10
0	other care provider	awaiting clinical code migration to emis web	laboratory result	< 10
0	other care provider	clinic note	community clinic	< 10
0	other care provider	first attendance face to face	awaiting review	< 10
0	other care provider	inbound referral	awaiting review	< 10
0	other care provider	non-consultation data	data transferred from other system	< 10
0	other care provider	ooh report	out of hours, non practice	< 10
0	other care provider	telephone encounter	awaiting review	< 10
0		awaiting clinical code migration to emis web	follow-up/routine visit	< 10
0		awaiting clinical code migration to emis web	letter	< 10
0		awaiting clinical code migration to emis web	minor injuries unit	< 10
0		awaiting clinical code migration to emis web	secretary	< 10
0		email received from third party	awaiting review	< 10
0		gp surgery	non-consultation data	< 10
0		hospital inpatient report	awaiting review	< 10
0		hospital inpatient report	discharge details	< 10
0		multidisciplinary team meeting with patient	awaiting review	< 10
0		night visit note	night visit, local rota	< 10
0		provision of general practitioner intermediate care	gp2gp import	< 10
0		telephone triage encounter	nhs direct report	< 10
0		third party consultation	third party	< 10
0	other care provider	nurse telephone triage	nurse telephone triage	< 10

For peer review only

Annex 3: *NHS targets for cancer wait times*

			Operational standard
Maximum two weeks from	Receipt of urgent referral for suspected cancer to first outpatient attendance		93%
	Receipt of referral of any patient with breast symptoms (where cancer not suspected) to first hospital assessment		93%
Maximum 28 days from	Receipt of two week wait referral for suspected cancer, receipt of urgent referral from a cancer screening programme (breast, bowel, cervical), and receipt of two week wait referral of any patient with breast symptoms (where cancer not suspected), to the date the patient is informed of a diagnosis or ruling out of cancer		75%
Maximum one month (31 days) from:	Decision to treat to first definitive treatment		96%
	Decision to treat/earliest clinically appropriate date to start of second or subsequent treatment(s) for all cancer patients including those diagnosed with a recurrence where the subsequent treatment is:	surgery	94%
		drug treatment	98%
		radiotherapy	94%
Maximum two months (62 days) from:	Urgent referral for suspected cancer to first treatment (62-day classic)		85%
	Urgent referral from a NHS Cancer Screening Programme (breast, cervical or bowel) for suspected cancer to first treatment		90%

Annex 4: Comparing Cancer Wait Times counts for referrals and first treatments with the NCRAS data equity pack

Table A4.1: Comparing Cancer Wait Times counts for referrals and first treatments with the NCRAS data equity pack

	01 Apr 2020 - 31 Jan 2021					
	First treatments for new cancer			Urgent cancer referrals		
	Observed	Expected	Percentage change (95% CI)	Observed	Expected	Percentage change (95% CI)
Cancer wait times data						
All	224,323	267,946	-16.3% (-16.6, -15.9)	1,673,775	2,071,967	-19.2% (-19.3, -19.1)
NCRAS data equity pack						
All	219,410	254,436	-13.8% (-14.3, -13.3)	1,658,309	1,984,489	-16.4% (-16.6, -16.3)
Breast	30,488	40,530	-24.8% (-25.9, -23.6)	337,582	363,139	-7% (-7.5, -6.6)
Gynaecological	11,281	12,344	-8.6% (-10.9, -6.2)	158,723	176,985	-10.3% (-10.9, -9.7)
Head and Neck	8,892	9,901	-10.2% (-12.7, -7.6)	163,668	189,837	-13.8% (-14.4, -13.2)
Lower GI	23,507	27,056	-13.1% (-14.6, -11.6)	302,369	366,677	-17.5% (-17.9, -17.1)
Lung	24,796	27,409	-9.5% (-11.1, -8.0)	33,830	53,641	-36.9% (-37.8, -36.1)
Skin	40,977	43,475	-5.7% (-7.0, -4.5)	338,172	429,802	-21.3% (-21.7, -21.0)
Upper GI	17,059	17,586	-3% (-5.0, -0.9)	141,720	163,013	-13.1% (-13.7, -12.4)
Urological	37,970	50,056	-24.1% (-25.2, -23.1)	134,389	184,642	-27.2% (-27.7, -26.7)
All other	24,441	26,080	-6.3% (-7.9, -4.6)	47,856	56,753	-15.7% (-16.7, -14.6)

For peer review only

Annex 5: Patient demographics and person-time and total numbers of observed activity in CPRD Aurum sample

Table A5.1: Patient demographics in CPRD Aurum sample as at 22 March 2020

	Patient count as at 22 March 2020	
	n	%
All	375,501	
Female	187,509	49.9%
Male	187,992	50.1%
Under 11	39,611	10.5%
11 to 19	43,406	11.6%
20 to 49	157,962	42.1%
50 to 69	87,482	23.3%
70 and older	47,040	12.5%
IMD Quintile - 1	78,759	21.0%
IMD Quintile - 2	73,046	19.5%
IMD Quintile - 3	71,840	19.1%
IMD Quintile - 4	77,545	20.7%
IMD Quintile - 5	74,020	19.7%
IMD not recorded	291	0.1%

Table A5.2: Person time (weeks) and total primary care activity analysed - CPRD Aurum

	3 January 2016 - 21March 2020*				22 March 2020 - 30 January 2021			
	Patient-time (weeks)	100,000 person-months	Activity count	Observed rate per 100,000 person-months	Patient-time (weeks)	100,000 person-months	Activity count	Observed rate per 100,000 person-months
Consultations excl flu vaccinations	78,868,977	181.44	6,912,079	38,095	16,701,707	38.19	1,306,206	34,201
Routine Referrals	78,868,977	181.44	312,422	1,722	16,701,707	38.19	40,744	1,067
2 Week Wait Referrals	78,868,977	181.44	38,905	214	16,701,707	38.19	10,235	268

**The pre-pandemic period consists of 220 weeks or 51.61 months, the post pandemic period is 45 weeks or 10.29 months*

For peer review only

Annex 6: Observed vs expected appointment and cancer diagnosis counts from CWT data from 01 Jan 2019 (per person-month)

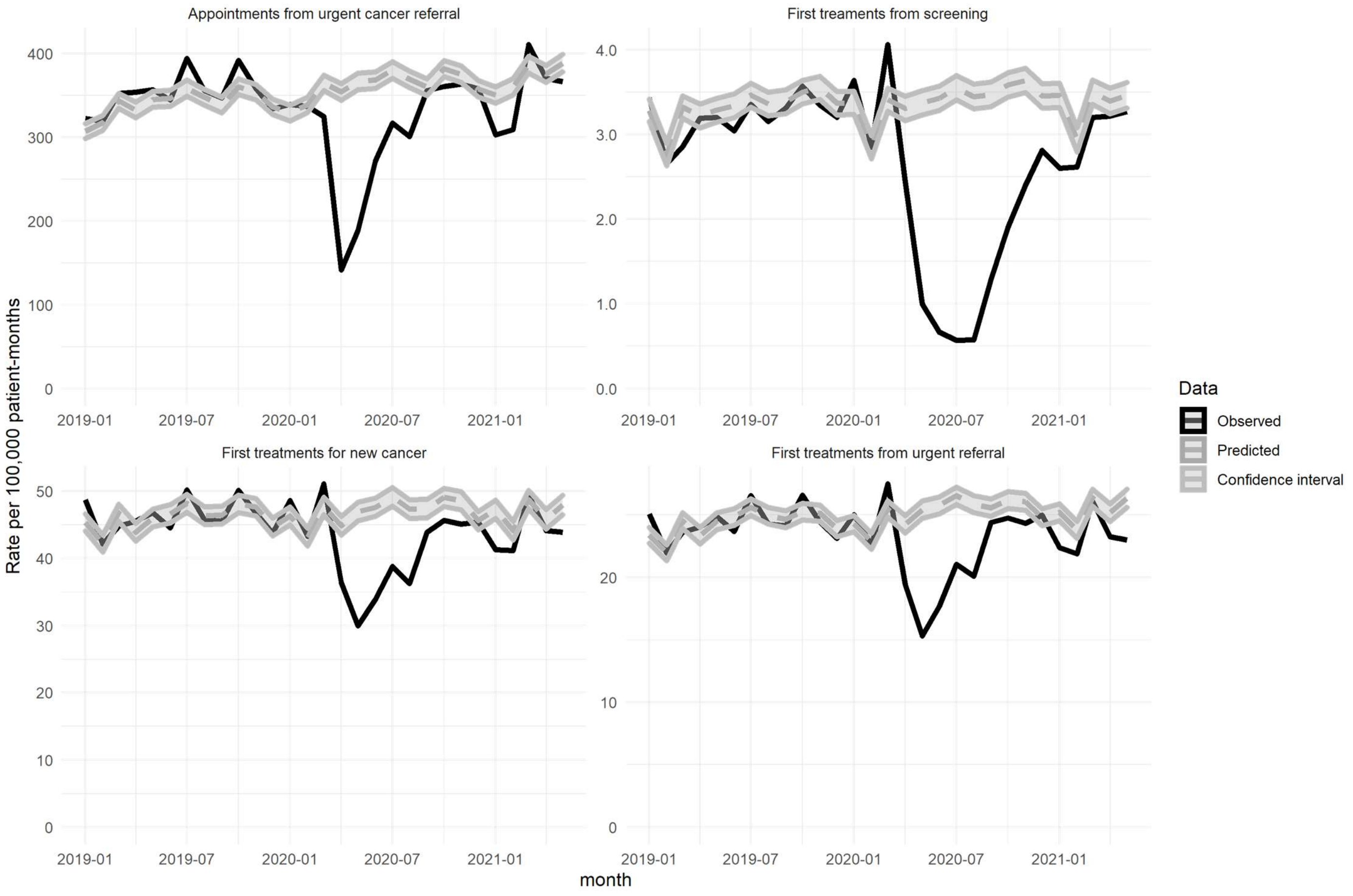

For peer review only - http://bmjopen.bmj.com/site/about/guidelines.xhtml

Annex 7: Observed vs expected consultations by IMD quintile

Figure A7: Observed vs expected consultations per person per week by IMD quintile, age-standardised (01 Jan 2019 – 30 Jan 2021)

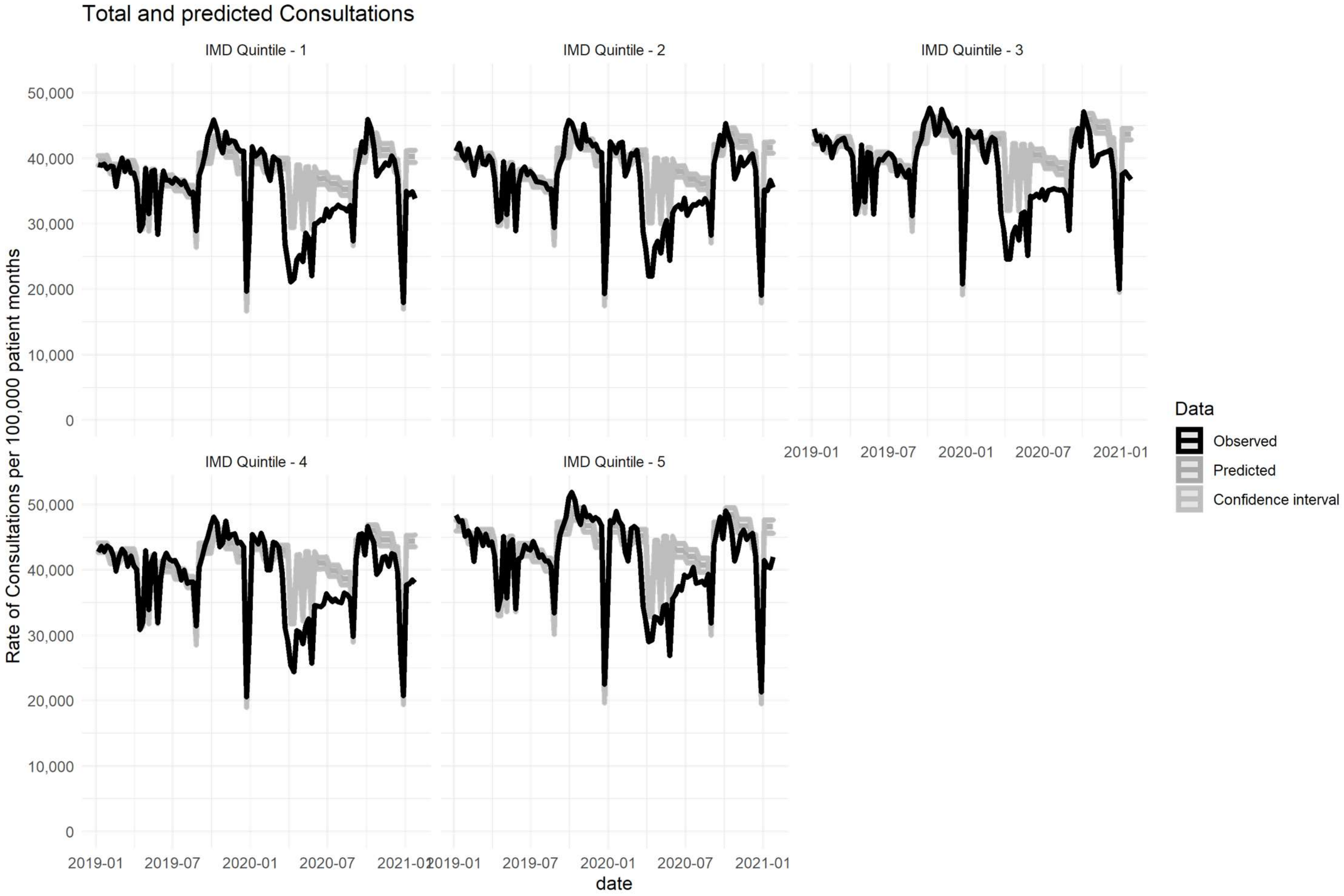

w only

STROBE Statement—checklist of items that should be included in reports of observational studies

	Item No	Recommendation	Page No
**Title and abstract**	1	(*a*) Indicate the study's design with a commonly used term in the title or the abstract	2
		(*b*) Provide in the abstract an informative and balanced summary of what was done and what was found	2
**Introduction**			
Background/rationale	2	Explain the scientific background and rationale for the investigation being reported	3
Objectives	3	State specific objectives, including any prespecified hypotheses	3
**Methods**			
Study design	4	Present key elements of study design early in the paper	4
Setting	5	Describe the setting, locations, and relevant dates, including periods of recruitment, exposure, follow-up, and data collection	4
Participants	6	(*a*) *Cohort study*—Give the eligibility criteria, and the sources and methods of selection of participants. Describe methods of follow-up<br>*Case-control study*—Give the eligibility criteria, and the sources and methods of case ascertainment and control selection. Give the rationale for the choice of cases and controls<br>*Cross-sectional study*—Give the eligibility criteria, and the sources and methods of selection of participants	4
		(*b*) *Cohort study*—For matched studies, give matching criteria and number of exposed and unexposed<br>*Case-control study*—For matched studies, give matching criteria and the number of controls per case	
Variables	7	Clearly define all outcomes, exposures, predictors, potential confounders, and effect modifiers. Give diagnostic criteria, if applicable	4/5
Data sources/ measurement	8*	For each variable of interest, give sources of data and details of methods of assessment (measurement). Describe comparability of assessment methods if there is more than one group	*4/5*
Bias	9	Describe any efforts to address potential sources of bias	5/6
Study size	10	Explain how the study size was arrived at	4
Quantitative variables	11	Explain how quantitative variables were handled in the analyses. If applicable, describe which groupings were chosen and why	4-6
Statistical methods	12	(*a*) Describe all statistical methods, including those used to control for confounding	5/6
		(*b*) Describe any methods used to examine subgroups and interactions	5/6
		(*c*) Explain how missing data were addressed	4-6
		(*d*) *Cohort study*—If applicable, explain how loss to follow-up was addressed<br>*Case-control study*—If applicable, explain how matching of cases and controls was addressed<br>*Cross-sectional study*—If applicable, describe analytical methods taking account of sampling strategy	N/A
		(*e*) Describe any sensitivity analyses	4-6

Continued on next page

For peer review only - http://bmjopen.bmj.com/site/about/guidelines.xhtml

**Results**			
Participants	13*	(a) Report numbers of individuals at each stage of study—eg numbers potentially eligible, examined for eligibility, confirmed eligible, included in the study, completing follow-up, and analysed	4/5
		(b) Give reasons for non-participation at each stage	N/A
		(c) Consider use of a flow diagram	N/A
Descriptive data	14*	(a) Give characteristics of study participants (eg demographic, clinical, social) and information on exposures and potential confounders	6-8
		(b) Indicate number of participants with missing data for each variable of interest	6-8
		(c) *Cohort study*—Summarise follow-up time (eg, average and total amount)	N/A
Outcome data	15*	*Cohort study*—Report numbers of outcome events or summary measures over time	*6/7*
		Case-control study—Report numbers in each exposure category, or summary measures of exposure	*N/A*
		Cross-sectional study—Report numbers of outcome events or summary measures	*N/A*
Main results	16	(*a*) Give unadjusted estimates and, if applicable, confounder-adjusted estimates and their precision (eg, 95% confidence interval). Make clear which confounders were adjusted for and why they were included	6-8
		(*b*) Report category boundaries when continuous variables were categorized	N/A
		(*c*) If relevant, consider translating estimates of relative risk into absolute risk for a meaningful time period	N/A
Other analyses	17	Report other analyses done—eg analyses of subgroups and interactions, and sensitivity analyses	6-8
**Discussion**			
Key results	18	Summarise key results with reference to study objectives	9
Limitations	19	Discuss limitations of the study, taking into account sources of potential bias or imprecision. Discuss both direction and magnitude of any potential bias	9/10
Interpretation	20	Give a cautious overall interpretation of results considering objectives, limitations, multiplicity of analyses, results from similar studies, and other relevant evidence	10
Generalisability	21	Discuss the generalisability (external validity) of the study results	9/10
**Other information**			
Funding	22	Give the source of funding and the role of the funders for the present study and, if applicable, for the original study on which the present article is based	1

*Give information separately for cases and controls in case-control studies and, if applicable, for exposed and unexposed groups in cohort and cross-sectional studies.

Note: An Explanation and Elaboration article discusses each checklist item and gives methodological background and published examples of transparent reporting. The STROBE checklist is best used in conjunction with this article (freely available on the Web sites of PLoS Medicine at http://www.plosmedicine.org/, Annals of Internal Medicine at http://www.annals.org/, and Epidemiology at http://www.epidem.com/). Information on the STROBE Initiative is available at www.strobe-statement.org.

For peer review only
